# Supplementary material for: Alteration in the Morphological and Transcriptomic Profiles of Acinetobacter baumannii after Exposure to Colistin
Source: Microorganisms. 2024 Aug 11;12(8):1644. doi: 10.3390/microorganisms12081644 (PMC11356899; doi:10.3390/microorganisms12081644)
Supplement: Supplementary file 1 [file microorganisms-12-01644-s001.zip › microorganisms-3143059-supplementary.pdf]

Supplementary data

Supplementary Table S1. Transcriptional level of the colistin susceptible and its colistin-resistant isotype mutant

Gene or locus_tag	Z0317AB0082			Z0317AB0082-R					Z0317AB0082-R /Z0317AB0082
	**NT**	**colistin 2 ug/ml**	**R-transc. level by treatment of the inh. conc. of colistin**	**NT**	**colistin 2 ug/ml**	**colistin 64 ug/ml**	**R-transc. level by treatment of the sub-inh. conc. of colistin**	**R-transc. level by treatment of the inh. conc. of colistin**	
dnaA	1.1	1.2	1.1	1.3	2.6	2.5	2.0	2.0	**1.1**
dnaN	3.5	4.7	1.4	5.9	8.9	10.3	1.5	1.7	**1.7**
recF	0.3	0.6	2.1	0.4	0.9	1.1	2.1	2.6	**1.5**
gyrB	3.7	5.6	1.5	6.3	8.2	9.6	1.3	1.5	**1.7**
cybC	0.4	1.0	2.3	1.9	2.3	2.7	1.2	1.5	**4.3**
F3P16_RS00030	0.0	0.0	2.1	0.0	0.1	0.2	3.0	3.5	**7.9**
F3P16_RS00035	0.5	0.6	1.1	0.6	0.7	1.1	1.2	1.9	**1.0**
F3P16_RS00040	0.0	0.0	1.6	0.0	0.0	0.2	3.3	19.4	**1.5**
F3P16_RS00045	0.1	0.3	4.1	0.3	0.1	0.4	0.4	1.4	**3.7**
F3P16_RS00050	0.1	0.1	1.8	0.1	0.0	0.3	0.4	2.3	**2.2**
erpA	0.0	0.0	2.1	0.3	0.2	0.8	0.8	2.6	**20.2**
F3P16_RS00060	0.0	0.1	1.7	0.0	0.0	0.7	1.0	16.0	**1.0**
F3P16_RS00065	0.2	0.3	1.5	0.3	0.3	1.0	1.0	3.2	**1.7**
tyrS	0.2	0.2	1.2	0.2	0.3	0.6	1.1	2.7	**1.6**
F3P16_RS00080	10000.8	10000.0	1.0	10000.2	9999.1	9999.8	1.0	1.0	**1.0**
F3P16_RS00085	2.0	1.5	0.7	1.7	2.4	7.6	1.4	4.4	**0.9**
F3P16_RS00090	0.5	1.0	2.1	1.0	0.8	5.2	0.8	5.3	**2.1**

F3P16_RS00095	13853.6	12855.4	0.9	12098.6	10115.2	11101.7	0.8	0.9	**0.9**
rrf]	1.4	1.5	1.0	2.6	2.6	5.2	1.0	2.0	**1.9**
F3P16_RS00105	0.0	0.0	1.2	0.1	0.0	0.1	0.9	2.0	**3.0**
F3P16_RS00110	0.0	0.2	3.9	0.3	0.4	0.6	1.4	2.1	**6.7**
F3P16_RS00115	0.1	0.2	2.5	0.1	0.1	0.2	1.0	1.6	**1.6**
F3P16_RS00120	0.0	0.0	UC	0.1	0.1	0.2	0.9	1.9	**UC**
rsmB	0.4	0.7	1.6	0.8	1.1	1.4	1.4	1.9	**1.7**
fmt]	0.6	0.9	1.6	0.6	1.2	1.6	1.9	2.6	**1.1**
ilvD	0.8	1.6	1.9	1.5	1.8	2.8	1.2	1.8	**1.9**
F3P16_RS00140	0.2	0.4	1.8	0.3	0.4	0.5	1.2	1.5	**1.6**
F3P16_RS00145	0.1	0.1	1.2	0.1	0.1	0.2	0.6	1.3	**1.4**
F3P16_RS00150	0.0	0.1	2.0	0.1	0.1	0.2	1.2	2.1	**2.7**
F3P16_RS00155	0.0	0.1	1.9	0.1	0.1	0.2	1.4	3.2	**1.7**
ppc]	0.6	0.9	1.7	0.5	0.5	1.2	1.0	2.4	**0.9**
F3P16_RS00165	0.1	0.2	2.9	0.2	0.1	0.2	0.7	1.2	**3.1**
F3P16_RS00170	0.1	0.2	3.4	0.2	0.2	0.3	1.1	1.3	**3.3**
F3P16_RS00175	0.2	0.5	3.0	0.6	0.5	0.5	0.8	0.8	**3.9**
F3P16_RS00180	0.1	0.1	1.1	0.2	0.3	0.3	1.2	1.2	**1.8**
dnaJ	1.7	2.0	1.2	2.6	2.8	7.3	1.1	2.8	**1.5**
F3P16_RS00190	0.0	0.0	UC	0.1	0.0	0.1	0.3	0.8	**UC**
dapB	1.2	1.6	1.4	1.7	1.8	2.9	1.0	1.7	**1.5**
F3P16_RS00200	0.3	0.6	2.1	0.5	0.7	1.1	1.4	2.2	**1.8**
F3P16_RS00205	1.2	0.9	0.7	0.6	1.9	2.5	3.1	4.1	**0.5**
dkgB	0.2	0.2	0.9	0.1	0.4	0.7	4.4	7.3	**0.5**
F3P16_RS00215	0.0	0.0	3.3	0.0	0.1	0.1	1.3	3.0	**3.1**
F3P16_RS00220	0.0	0.0	UC	0.0	0.0	0.1	1.0	9.1	**UC**
F3P16_RS00225	0.0	0.0	UC	0.0	0.0	0.1	UC	UC	**UC**
F3P16_RS00230	0.1	0.2	2.0	0.1	0.1	0.2	0.9	1.4	**1.5**
F3P16_RS00235	0.5	0.7	1.3	0.7	0.7	1.3	1.0	1.8	**1.4**
F3P16_RS00240	0.3	0.4	1.2	0.4	0.7	0.8	1.7	1.9	**1.2**

F3P16_RS00245	0.5	0.7	1.5	0.5	0.9	1.4	1.7	2.7	**1.1**
F3P16_RS00250	0.5	0.5	1.0	0.6	0.8	1.3	1.4	2.3	**1.1**
mutY	0.5	0.6	1.1	0.6	0.6	0.9	1.0	1.5	**1.2**
F3P16_RS00260	0.2	0.2	1.4	0.3	0.4	0.5	1.2	1.6	**2.0**
F3P16_RS00265	0.4	0.8	2.0	1.0	0.5	1.3	0.6	1.3	**2.3**
F3P16_RS00270	0.0	0.0	UC	0.0	0.1	0.1	UC	UC	**UC**
F3P16_RS00275	0.0	0.1	2.7	0.1	0.2	0.3	3.8	5.4	**2.4**
F3P16_RS00280	0.0	0.1	3.1	0.1	0.1	0.2	0.8	2.1	**2.4**
F3P16_RS00285	0.5	0.8	1.8	0.7	0.8	1.6	1.1	2.2	**1.5**
F3P16_RS00290	0.1	0.1	1.9	0.1	0.1	0.7	0.7	4.5	**2.9**
F3P16_RS00295	0.0	0.0	UC	0.0	0.0	0.1	UC	UC	**UC**
F3P16_RS00300	1.1	1.5	1.4	3.1	3.3	5.9	1.1	1.9	**2.7**
F3P16_RS00305	3.5	6.2	1.8	7.1	8.7	10.5	1.2	1.5	**2.1**
dapA	6.9	13.5	2.0	14.2	17.3	19.3	1.2	1.4	**2.1**
pncA	0.1	0.1	2.1	0.3	0.3	0.2	1.0	0.9	**3.8**
F3P16_RS00320	0.1	0.1	1.7	0.1	0.1	0.3	0.7	2.7	**1.8**
F3P16_RS00325	0.0	0.0	1.4	0.0	0.0	0.2	1.3	7.6	**2.0**
F3P16_RS00330	0.0	0.1	6.9	0.0	0.0	0.3	0.5	5.2	**3.5**
hppD	1.4	10.4	7.2	11.7	3.8	4.0	0.3	0.3	**8.1**
F3P16_RS00340	0.1	0.5	4.4	0.7	0.5	0.7	0.7	1.0	**5.5**
F3P16_RS00345	0.1	1.5	10.3	1.9	0.6	0.3	0.3	0.1	**13.3**
maiA	1.5	16.5	10.7	18.8	5.5	1.7	0.3	0.1	**12.2**
fahA	0.9	9.7	10.3	7.6	2.1	0.8	0.3	0.1	**8.0**
F3P16_RS00360	0.1	0.5	9.0	0.5	0.2	0.5	0.4	1.0	**9.1**
F3P16_RS00365	0.0	0.1	UC	0.1	0.2	0.4	1.8	3.6	**UC**
zigA	0.0	0.1	4.7	0.7	1.0	0.8	1.5	1.2	**55.6**
F3P16_RS00375	0.1	0.2	2.9	0.2	0.2	0.4	1.0	1.7	**3.4**
hutC	0.5	0.8	1.5	0.8	0.5	0.6	0.6	0.7	**1.5**
F3P16_RS00385	0.2	0.4	1.9	0.4	0.1	0.3	0.3	0.8	**2.1**
hutU	4.6	19.1	4.1	17.5	3.8	3.1	0.2	0.2	**3.8**

hutH	2.0	8.5	4.3	9.1	2.7	1.0	0.3	0.1	**4.6**
F3P16_RS00400	1.4	5.9	4.3	6.6	2.7	1.3	0.4	0.2	**4.7**
hutI	0.9	2.8	3.2	3.4	1.7	1.0	0.5	0.3	**3.8**
hutG	0.3	0.8	2.9	1.0	0.6	0.4	0.6	0.4	**3.6**
F3P16_RS00415	0.0	0.0	1.2	0.0	0.0	0.0	4.3	4.7	**0.6**
F3P16_RS00420	0.2	0.1	0.7	0.1	0.1	0.2	1.7	3.1	**0.5**
murI	0.2	0.4	1.8	0.5	0.7	0.7	1.4	1.4	**2.4**
glmS	1.1	3.3	3.0	4.3	6.1	8.3	1.4	1.9	**3.9**
glmU	2.8	8.0	2.8	6.7	9.2	11.9	1.4	1.8	**2.4**
F3P16_RS00440	0.5	1.3	2.9	1.0	2.1	2.2	2.0	2.2	**2.3**
thiL	1.7	2.1	1.3	2.2	3.4	3.9	1.6	1.8	**1.3**
nusB	7.5	10.2	1.4	9.8	13.9	18.1	1.4	1.8	**1.3**
ribE	9.0	13.9	1.5	14.0	19.8	28.5	1.4	2.0	**1.6**
F3P16_RS00460	2.2	3.0	1.4	2.8	3.9	6.1	1.4	2.2	**1.3**
F3P16_RS00465	0.6	1.1	1.7	1.2	1.5	2.1	1.3	1.7	**1.9**
serB	0.4	0.5	1.1	0.7	0.6	1.3	0.9	1.8	**1.7**
F3P16_RS00480	0.8	0.5	0.6	0.6	0.8	2.4	1.2	3.9	**0.8**
F3P16_RS00485	0.6	1.4	2.4	2.3	2.7	4.5	1.2	1.9	**3.8**
dtd]	0.0	0.0	1.6	0.1	0.1	0.2	1.3	2.3	**2.7**
pncB	0.2	0.3	2.1	0.4	0.4	0.6	1.1	1.5	**2.2**
F3P16_RS00500	0.3	0.5	1.6	0.7	0.7	0.8	1.0	1.2	**2.0**
asd]	0.4	0.7	2.0	0.8	0.9	1.2	1.2	1.5	**2.2**
F3P16_RS00510	0.0	0.0	1.1	0.0	0.0	0.1	1.4	5.7	**0.6**
F3P16_RS00515	0.0	0.0	UC	0.0	0.0	0.0	UC	UC	**UC**
F3P16_RS00520	0.0	0.0	0.0	0.0	0.0	0.0	0.0	2.3	**0.7**
F3P16_RS00525	0.2	0.1	0.7	0.1	0.1	0.2	1.4	2.0	**0.5**
phoR	0.2	0.5	2.3	1.0	1.6	1.2	1.7	1.3	**4.0**
phoB	0.3	0.7	2.8	1.3	1.9	2.0	1.5	1.5	**5.0**
F3P16_RS00540	0.0	0.0	0.5	0.0	0.0	0.2	3.3	10.8	**0.3**
F3P16_RS00545	0.1	0.2	1.3	0.2	0.2	0.6	1.2	3.0	**1.7**

F3P16_RS00550	0.1	0.7	4.9	0.5	0.5	1.2	0.9	2.2	**3.8**
F3P16_RS00555	0.6	1.7	2.6	1.3	1.7	1.9	1.3	1.5	**2.1**
F3P16_RS00560	0.8	2.5	3.2	2.1	2.5	2.7	1.2	1.3	**2.7**
F3P16_RS00565	0.7	1.9	2.8	1.1	1.2	2.0	1.1	1.8	**1.7**
F3P16_RS00570	0.1	0.4	3.6	0.4	0.5	1.0	1.0	2.3	**3.6**
gshA	0.2	0.6	2.5	0.6	0.8	1.5	1.3	2.5	**2.4**
F3P16_RS00580	0.5	1.0	1.9	1.3	1.5	3.4	1.1	2.6	**2.5**
F3P16_RS00585	0.2	0.3	1.1	0.1	0.3	0.5	2.3	4.2	**0.5**
F3P16_RS00590	0.0	0.0	UC	0.0	0.0	0.0	UC	UC	**UC**
F3P16_RS00595	0.0	0.0	UC	0.0	0.0	0.0	UC	UC	**UC**
F3P16_RS00600	0.0	0.0	UC	0.0	0.0	0.0	UC	UC	**UC**
F3P16_RS00605	0.1	0.8	9.8	0.6	0.6	0.7	1.0	1.2	**7.3**
F3P16_RS00610	0.1	0.3	2.5	0.3	0.1	0.5	0.5	1.9	**2.2**
parE	0.5	0.8	1.8	0.7	0.8	1.4	1.2	2.1	**1.4**
F3P16_RS00620	0.1	0.1	1.5	0.1	0.2	0.4	2.2	3.6	**1.2**
alc]	0.0	0.1	3.7	0.1	0.1	0.3	1.0	2.3	**6.0**
hpxO	0.0	0.0	0.6	0.0	0.0	0.1	1.9	4.7	**0.6**
F3P16_RS00635	0.1	0.1	0.8	0.1	0.1	0.3	0.9	3.5	**1.3**
F3P16_RS00640	0.0	0.1	1.5	0.0	0.1	0.3	1.8	11.6	**0.7**
uraH	0.0	0.1	10.6	0.0	0.0	0.2	4.0	17.1	**2.0**
uraD	0.0	0.0	0.6	0.0	0.0	0.1	0.8	2.7	**1.0**
puuE	0.1	0.1	1.8	0.1	0.1	0.4	1.3	7.2	**0.9**
F3P16_RS00660	0.9	1.6	1.7	2.2	2.4	3.3	1.1	1.5	**2.3**
F3P16_RS00665	0.7	1.3	1.8	1.5	2.0	2.2	1.3	1.4	**2.1**
F3P16_RS00670	0.8	1.6	2.0	2.2	2.7	3.1	1.2	1.4	**2.8**
dsbD	0.6	1.3	2.1	1.4	2.0	1.9	1.5	1.4	**2.2**
F3P16_RS00680	0.0	0.0	0.7	0.0	0.1	0.1	4.7	2.4	**0.6**
F3P16_RS00685	0.0	0.1	5.7	0.1	0.2	0.2	1.6	1.2	**5.2**
F3P16_RS00690	0.2	0.6	3.8	1.1	1.1	1.8	1.0	1.6	**7.0**
F3P16_RS00695	0.0	0.1	1.9	0.0	0.1	0.1	2.3	3.2	**1.5**

xth]	0.2	0.4	2.2	0.6	0.5	1.3	0.8	2.2	**2.9**
pyrE	0.3	0.5	1.6	0.6	0.7	1.0	1.2	1.7	**1.7**
F3P16_RS00710	0.1	0.0	0.4	0.0	0.0	0.1	2.5	6.0	**0.3**
F3P16_RS00715	0.0	0.0	UC	0.0	0.0	0.0	UC	UC	**UC**
gshB	0.1	0.3	3.3	0.4	0.4	0.6	1.0	1.7	**4.2**
murG	0.9	1.8	2.0	2.5	2.8	4.8	1.1	1.9	**2.9**
murC	2.6	5.1	2.0	6.4	7.5	8.8	1.2	1.4	**2.5**
F3P16_RS00735	2.0	3.7	1.8	4.7	5.7	6.3	1.2	1.3	**2.3**
F3P16_RS00740	0.8	1.5	1.9	1.8	2.5	2.8	1.4	1.5	**2.3**
ftsA	2.3	4.6	2.0	3.9	5.3	6.0	1.4	1.5	**1.7**
ftsZ	1.7	2.7	1.6	3.0	3.3	5.9	1.1	2.0	**1.8**
lpxC	0.5	0.8	1.6	0.1	0.2	0.3	1.4	2.3	**0.3**
F3P16_RS00760	0.3	0.5	1.9	0.5	0.6	0.8	1.1	1.5	**2.0**
F3P16_RS00765	0.4	1.3	3.1	1.2	1.2	1.9	1.1	1.6	**2.8**
aceE	10.1	18.7	1.9	20.3	23.5	36.3	1.2	1.8	**2.0**
F3P16_RS00775	5.6	8.6	1.5	9.8	12.8	23.3	1.3	2.4	**1.8**
F3P16_RS00780	0.0	0.0	UC	0.0	0.1	0.0	5.0	4.0	**UC**
F3P16_RS00785	0.0	0.0	0.8	0.1	0.1	0.2	1.0	2.8	**2.8**
F3P16_RS00790	0.0	0.1	2.3	0.1	0.1	0.2	1.6	3.9	**2.5**
F3P16_RS00795	0.0	0.0	1.3	0.0	0.0	0.1	1.6	3.4	**1.2**
F3P16_RS00800	0.1	0.1	1.0	0.1	0.2	0.5	1.6	5.6	**1.0**
guaB	6.3	6.6	1.1	8.8	10.3	14.1	1.2	1.6	**1.4**
glmM	2.0	5.8	2.9	5.7	6.7	7.7	1.2	1.4	**2.8**
pdxH	1.1	3.1	2.9	2.6	3.3	3.7	1.3	1.4	**2.4**
recJ	0.5	1.2	2.5	1.1	1.5	1.6	1.4	1.4	**2.3**
F3P16_RS00825	1.7	19.7	11.4	47.8	44.9	78.5	0.9	1.6	**27.6**
prfB	1.2	1.4	1.1	1.8	2.6	3.1	1.4	1.7	**1.5**
F3P16_RS00835	1.1	1.2	1.1	1.6	2.5	2.7	1.6	1.7	**1.5**
F3P16_RS00840	0.5	0.6	1.2	0.7	1.0	1.3	1.3	1.8	**1.5**
F3P16_RS00845	0.4	1.1	2.7	1.2	1.4	2.1	1.1	1.7	**3.0**

F3P16_RS00850	0.1	0.2	2.7	0.3	0.4	0.7	1.3	2.5	**3.9**
F3P16_RS00855	0.1	0.2	3.4	0.2	0.4	0.7	1.6	3.0	**3.3**
F3P16_RS00860	0.0	0.1	2.6	0.1	0.1	0.2	0.9	2.2	**2.7**
acs]	0.5	1.1	2.3	1.0	0.6	2.0	0.6	2.0	**2.2**
F3P16_RS00870	0.0	0.0	2.1	0.0	0.1	0.1	2.9	3.3	**3.5**
F3P16_RS00875	0.0	0.0	1.1	0.0	0.0	0.1	5.5	6.8	**0.3**
sfnG	0.1	0.1	1.0	0.0	0.0	0.1	1.0	2.6	**0.7**
msuE	0.0	0.0	1.1	0.0	0.0	0.1	0.8	3.8	**0.9**
F3P16_RS00890	0.4	0.6	1.4	0.6	0.7	1.4	1.3	2.3	**1.4**
F3P16_RS00895	0.6	2.8	4.5	0.4	0.3	0.6	0.8	1.3	**0.7**
F3P16_RS00900	0.4	0.7	1.6	0.5	0.6	0.8	1.1	1.5	**1.2**
F3P16_RS00905	0.3	0.8	2.8	0.4	0.3	0.3	0.6	0.8	**1.5**
F3P16_RS00910	0.8	2.1	2.5	1.2	0.8	1.1	0.6	0.9	**1.4**
F3P16_RS00915	0.0	0.0	11.6	0.0	0.0	0.1	0.6	6.2	**6.9**
betT	1.1	0.7	0.6	1.7	2.2	3.2	1.3	2.0	**1.5**
omp3	5.9	6.7	1.1	19.7	28.4	80.3	1.4	4.1	**3.4**
F3P16_RS00930	0.0	0.0	1.7	0.0	0.0	0.1	2.9	4.3	**1.4**
uvrA	0.8	1.6	1.9	1.5	1.9	3.9	1.3	2.5	**1.8**
F3P16_RS00940	0.1	0.2	2.1	0.5	0.4	0.7	0.9	1.6	**3.9**
F3P16_RS00945	0.2	0.3	1.7	0.4	0.3	0.7	0.8	1.6	**2.2**
F3P16_RS00950	0.0	0.1	1.5	0.1	0.0	0.1	0.4	0.8	**2.5**
F3P16_RS00955	0.0	0.0	UC	0.0	0.0	0.1	1.1	1.4	**UC**
tenA	0.1	0.1	1.8	0.1	0.1	0.2	2.4	3.4	**1.0**
F3P16_RS00965	0.2	0.3	1.2	0.6	0.7	1.5	1.3	2.6	**2.6**
F3P16_RS00970	0.5	0.7	1.4	0.8	1.0	1.3	1.2	1.7	**1.6**
ssb]	1.3	1.4	1.1	1.7	1.9	5.2	1.1	3.0	**1.4**
F3P16_RS00980	0.1	0.2	1.3	0.2	0.2	1.0	1.5	6.3	**1.4**
F3P16_RS00985	0.1	0.1	0.7	0.1	0.0	0.3	0.5	3.5	**1.2**
F3P16_RS00990	0.2	0.3	1.4	0.2	0.3	1.0	1.7	5.1	**0.8**
F3P16_RS00995	0.0	0.0	1.0	0.0	0.0	0.1	0.3	3.5	**1.3**

F3P16_RS01000	0.1	0.1	2.0	0.1	0.1	0.4	1.3	4.6	**1.5**
gabT	0.0	0.1	4.0	0.1	0.1	0.1	1.9	2.1	**3.0**
F3P16_RS01010	0.7	5.2	7.2	4.9	4.6	8.2	0.9	1.7	**6.8**
F3P16_RS01015	0.1	0.4	2.9	0.3	0.4	0.4	1.1	1.2	**2.5**
F3P16_RS01020	0.3	1.2	3.4	0.8	0.8	0.9	1.0	1.1	**2.4**
F3P16_RS01025	0.1	0.1	1.5	0.1	0.1	0.4	1.6	7.0	**1.0**
F3P16_RS01030	0.5	0.8	1.6	0.7	0.9	1.2	1.2	1.7	**1.5**
F3P16_RS01035	0.3	0.5	1.5	0.5	0.5	0.9	1.0	1.9	**1.6**
F3P16_RS01040	0.1	0.2	1.5	0.3	0.3	0.6	0.8	1.7	**3.0**
F3P16_RS01045	6.3	7.6	1.2	12.5	13.0	30.2	1.0	2.4	**2.0**
F3P16_RS01050	0.1	0.3	1.8	0.2	0.3	0.2	1.2	0.8	**1.6**
F3P16_RS01055	0.0	0.1	6.8	0.0	0.0	0.1	1.3	4.8	**3.0**
F3P16_RS01060	0.0	0.0	1.5	0.0	0.0	0.2	6.0	20.0	**0.4**
F3P16_RS01065	0.1	0.2	2.3	0.1	0.1	0.3	0.8	2.3	**1.9**
F3P16_RS01070	0.0	0.1	3.7	0.0	0.1	0.2	2.1	4.3	**1.1**
F3P16_RS01075	0.1	0.1	1.9	0.1	0.1	0.2	0.9	1.5	**2.2**
F3P16_RS01080	0.0	0.0	13.2	0.1	0.1	0.1	0.7	1.4	**18.9**
F3P16_RS01090	0.0	0.0	UC	0.0	0.0	0.0	UC	UC	**UC**
F3P16_RS01095	0.0	0.0	UC	0.0	0.0	0.0	UC	UC	**UC**
F3P16_RS01100	0.0	0.0	UC	0.0	0.0	0.0	UC	UC	**UC**
F3P16_RS01105	0.0	0.0	UC	0.0	0.0	0.0	UC	UC	**UC**
F3P16_RS01110	0.0	0.0	UC	0.0	0.0	0.1	0.0	8.0	**UC**
F3P16_RS01115	0.0	0.0	2.1	0.1	0.1	0.2	1.1	1.8	**10.4**
F3P16_RS01120	0.0	0.0	UC	0.0	0.0	0.1	0.7	1.6	**UC**
F3P16_RS01125	0.0	0.0	UC	0.0	0.0	0.0	3.0	0.6	**UC**
F3P16_RS01130	0.0	0.0	4.2	0.0	0.0	0.0	0.6	0.3	**3.5**
F3P16_RS01135	0.0	0.0	UC	0.0	0.0	0.0	UC	UC	**UC**
F3P16_RS01140	0.6	0.7	1.1	1.2	0.9	2.3	0.7	1.9	**2.0**
F3P16_RS01145	1.7	3.5	2.1	3.1	4.5	5.0	1.5	1.6	**1.9**
F3P16_RS01150	0.4	5.3	12.6	8.6	5.1	23.0	0.6	2.7	**20.7**

F3P16_RS01155	0.1	0.1	1.3	0.1	0.2	0.5	1.6	5.0	**0.9**
F3P16_RS01160	0.0	0.0	4.2	0.0	0.0	0.1	3.0	10.3	**2.0**
F3P16_RS01165	0.0	0.0	1.1	0.0	0.0	0.1	0.8	2.9	**2.0**
F3P16_RS01170	0.0	0.0	UC	0.0	0.0	0.0	UC	UC	**UC**
aqpZ	0.0	0.1	3.5	0.1	0.1	0.3	1.1	4.7	**1.7**
F3P16_RS01180	0.0	0.0	0.6	0.0	0.1	0.1	1.5	2.2	**1.0**
F3P16_RS01185	0.0	0.0	1.6	0.0	0.1	0.1	1.5	3.2	**1.5**
hisF	0.2	0.1	0.9	0.2	0.2	0.5	0.8	2.2	**1.3**
F3P16_RS01195	0.3	0.3	1.2	0.4	0.4	0.9	1.1	2.2	**1.5**
F3P16_RS01200	0.2	0.2	1.5	0.2	0.3	0.5	1.3	2.4	**1.5**
F3P16_RS01205	0.1	0.3	3.3	0.3	0.2	0.5	0.8	1.8	**3.0**
F3P16_RS01210	0.2	0.2	1.4	0.2	0.1	0.5	0.8	3.3	**0.9**
F3P16_RS01215	0.0	0.1	2.8	0.0	0.0	0.1	0.1	2.4	**2.3**
hisA	0.3	0.4	1.7	0.5	0.6	1.2	1.2	2.5	**2.0**
F3P16_RS01225	0.0	0.1	1.7	0.0	0.1	0.1	2.2	4.1	**0.8**
F3P16_RS01230	0.2	0.2	0.9	0.1	0.1	0.1	1.2	1.3	**0.4**
hisH	0.8	1.1	1.4	1.0	1.2	2.8	1.2	2.8	**1.2**
hisB	1.2	1.5	1.3	1.7	2.3	3.2	1.3	1.8	**1.5**
F3P16_RS01245	0.1	0.1	1.0	0.2	0.2	0.6	0.9	2.6	**2.2**
F3P16_RS01250	0.0	0.1	UC	0.0	0.1	0.2	1.5	4.6	**UC**
F3P16_RS01255	0.0	0.1	UC	0.0	0.1	0.2	1.5	4.6	**UC**
F3P16_RS01260	0.1	0.2	4.3	0.2	0.1	0.1	0.7	0.9	**3.2**
F3P16_RS01265	1.3	3.6	2.8	4.1	3.0	3.8	0.7	0.9	**3.2**
F3P16_RS01270	1.2	2.7	2.2	2.2	2.3	5.1	1.0	2.3	**1.8**
ompR	4.6	10.7	2.3	9.8	8.7	21.3	0.9	2.2	**2.1**
F3P16_RS01280	1.4	3.1	2.3	2.8	3.7	6.0	1.3	2.2	**2.0**
F3P16_RS01285	0.0	0.3	15.0	0.3	0.3	0.7	0.9	2.3	**19.1**
F3P16_RS01290	0.2	0.3	1.9	0.3	0.9	1.0	2.7	3.1	**1.9**
F3P16_RS01295	0.2	0.3	1.4	0.2	0.2	0.2	0.9	1.1	**0.7**
ettA	2.3	3.0	1.3	4.8	6.2	11.5	1.3	2.4	**2.1**

F3P16_RS01305	0.1	0.1	2.4	0.1	0.1	0.3	2.3	5.7	**1.0**
F3P16_RS01310	0.0	0.0	0.4	0.0	0.0	0.1	0.5	2.4	**1.6**
F3P16_RS01315	0.0	0.0	0.5	0.0	0.0	0.0	1.0	2.9	**1.0**
F3P16_RS01320	0.0	0.0	1.3	0.1	0.0	0.2	0.7	3.0	**1.5**
F3P16_RS01325	0.0	0.0	1.1	0.0	0.0	0.0	7.0	5.7	**1.0**
F3P16_RS01330	0.0	0.0	UC	0.0	0.0	0.0	UC	UC	**UC**
F3P16_RS01335	0.2	0.1	0.6	0.2	0.1	0.3	0.7	1.9	**1.0**
F3P16_RS01340	0.0	0.0	0.7	0.0	0.0	0.1	5.4	7.4	**0.6**
F3P16_RS01345	0.1	0.1	1.0	0.0	0.1	0.1	4.8	6.7	**0.2**
F3P16_RS01350	0.2	0.2	1.4	0.2	0.3	0.3	1.8	1.9	**0.9**
F3P16_RS01355	0.1	0.2	1.7	0.2	0.4	0.5	1.7	2.3	**1.6**
F3P16_RS01360	0.1	0.4	3.7	0.4	0.6	0.8	1.4	1.9	**3.9**
F3P16_RS01365	0.1	0.2	3.4	0.2	0.2	0.4	1.1	2.1	**3.2**
F3P16_RS01370	0.4	0.2	0.6	0.3	0.4	1.1	1.5	3.7	**0.8**
F3P16_RS01375	0.4	0.2	0.6	0.3	0.4	1.1	1.5	3.7	**0.8**
F3P16_RS01380	0.0	0.0	UC	0.0	0.0	0.1	2.0	47.9	**UC**
F3P16_RS01385	0.4	0.4	1.0	0.4	0.7	1.4	1.5	3.1	**1.0**
F3P16_RS01390	0.4	0.2	0.6	0.3	0.4	1.1	1.5	3.7	**0.8**
F3P16_RS01395	0.1	0.3	2.5	0.5	0.4	1.2	0.9	2.6	**4.2**
gltX	1.5	2.4	1.6	1.8	2.2	2.6	1.2	1.4	**1.3**
F3P16_RS01405	0.3	0.7	2.7	0.5	0.9	0.8	1.7	1.5	**2.1**
F3P16_RS01410	0.0	0.0	0.4	0.0	0.0	0.1	UC	UC	**0.0**
rsmH	0.8	1.4	1.8	1.3	1.8	2.4	1.4	1.8	**1.6**
ftsL	0.5	1.3	2.7	0.7	1.5	2.2	2.3	3.2	**1.4**
ftsI	1.0	2.3	2.4	2.5	3.8	4.6	1.5	1.8	**2.6**
F3P16_RS01430	0.8	1.9	2.5	2.0	3.4	4.0	1.7	2.1	**2.5**
murF	0.9	1.7	1.9	1.5	2.6	4.4	1.8	2.9	**1.7**
mraY	0.7	1.5	2.3	1.4	2.2	4.3	1.5	3.0	**2.2**
F3P16_RS01445	0.4	0.7	1.9	0.6	1.0	1.1	1.8	2.0	**1.5**
F3P16_RS01450	0.6	0.8	1.3	0.7	1.0	1.8	1.4	2.5	**1.2**

ponA	0.1	0.2	3.2	0.2	0.3	0.5	1.8	2.8	**2.3**
F3P16_RS01460	0.1	0.1	0.5	0.0	0.0	0.2	1.6	7.2	**0.2**
F3P16_RS01465	0.0	0.1	3.5	0.0	0.0	0.1	2.8	5.1	**1.0**
F3P16_RS01470	0.0	0.0	1.6	0.0	0.0	0.0	0.6	1.0	**0.8**
F3P16_RS01475	0.0	0.0	0.5	0.0	0.0	0.1	0.0	37.6	**0.1**
F3P16_RS01480	0.0	0.0	1.3	0.0	0.0	0.1	1.8	6.6	**0.9**
aroK	0.3	0.3	1.0	0.2	0.4	0.9	2.2	4.9	**0.6**
aroB	0.5	0.8	1.4	0.6	0.8	1.4	1.2	2.2	**1.2**
F3P16_RS01495	0.2	0.5	2.4	0.5	0.7	1.0	1.5	2.1	**2.3**
gltB	4.1	4.6	1.1	1.9	5.2	3.7	2.7	1.9	**0.5**
F3P16_RS01505	1.5	1.8	1.2	0.9	2.2	2.0	2.3	2.1	**0.7**
F3P16_RS01510	0.0	0.1	14.3	0.0	0.0	0.1	0.6	2.7	**6.5**
F3P16_RS01515	0.2	0.5	3.0	0.5	0.5	0.8	0.9	1.5	**2.9**
F3P16_RS01520	0.4	1.3	3.3	1.0	1.1	1.7	1.1	1.7	**2.5**
F3P16_RS01525	0.1	0.2	4.3	0.3	0.3	0.4	0.9	1.4	**5.4**
F3P16_RS01530	0.0	0.0	UC	0.0	0.0	0.0	1.0	2.3	**UC**
F3P16_RS01535	0.1	0.1	1.3	0.2	0.3	0.5	1.7	2.7	**1.6**
bfr]	0.4	1.0	2.7	3.0	2.8	3.1	0.9	1.0	**8.4**
F3P16_RS01545	0.0	0.0	UC	0.0	0.0	0.1	UC	UC	**UC**
F3P16_RS01550	0.1	0.1	1.8	0.1	0.2	0.3	2.3	3.9	**1.3**
spoT	0.6	0.8	1.4	0.6	0.9	1.2	1.5	1.8	**1.1**
F3P16_RS01560	0.1	0.2	3.2	0.4	0.9	0.7	2.1	1.7	**5.6**
rpoZ	0.5	1.0	2.0	2.0	3.9	4.8	2.0	2.4	**3.9**
gmk	0.5	0.6	1.1	0.5	0.9	1.6	1.7	3.0	**1.0**
ispH	0.7	1.0	1.5	1.2	1.6	2.0	1.3	1.6	**1.8**
F3P16_RS01580	0.0	0.0	UC	0.0	0.0	0.1	UC	UC	**UC**
pilV	0.0	0.0	1.1	0.0	0.0	0.0	2.0	1.7	**1.0**
F3P16_RS01590	0.0	0.0	0.5	0.0	0.0	0.2	2.0	26.0	**0.2**
F3P16_RS01595	0.0	0.1	3.1	0.0	0.1	0.2	1.7	4.3	**2.1**
F3P16_RS01600	0.1	0.1	1.4	0.1	0.1	0.2	1.5	2.5	**1.4**

F3P16_RS01605	0.1	0.2	1.4	0.2	0.2	0.6	1.2	3.5	**1.2**
F3P16_RS01610	0.1	0.1	1.1	0.0	0.1	0.1	3.6	2.8	**0.7**
rpsP	14.3	15.1	1.1	20.6	35.1	48.0	1.7	2.3	**1.4**
rimM	31.9	39.8	1.2	55.1	85.5	120.6	1.6	2.2	**1.7**
trmD	15.0	20.8	1.4	32.2	58.1	83.5	1.8	2.6	**2.2**
rplS	7.2	9.0	1.3	16.2	29.5	51.8	1.8	3.2	**2.3**
F3P16_RS01635	0.4	0.4	1.2	0.5	1.0	0.9	2.0	1.8	**1.4**
F3P16_RS01640	0.0	0.0	0.8	0.0	0.0	0.0	1.6	1.9	**1.0**
truB	0.2	0.2	1.1	0.1	0.2	0.3	1.6	2.4	**0.9**
F3P16_RS01650	0.2	0.1	0.8	0.2	0.2	0.5	1.0	2.0	**1.2**
F3P16_RS01655	0.1	0.5	6.2	0.3	0.5	0.6	1.5	1.7	**4.1**
F3P16_RS01660	0.1	0.4	4.4	0.2	0.2	0.7	0.8	2.7	**2.7**
F3P16_RS01665	0.2	0.2	1.4	0.2	0.2	0.4	1.1	2.6	**1.1**
serA	1.8	2.5	1.4	3.0	4.7	8.2	1.5	2.7	**1.7**
F3P16_RS01675	0.5	0.7	1.4	1.0	1.5	1.6	1.5	1.6	**1.9**
F3P16_RS01680	0.0	0.1	2.4	0.1	0.3	0.3	2.8	2.9	**3.0**
F3P16_RS01685	0.6	0.4	0.8	0.5	1.1	1.3	2.0	2.6	**0.9**
F3P16_RS01690	0.4	0.4	1.1	0.3	0.6	0.7	2.0	2.4	**0.8**
F3P16_RS01695	0.0	0.0	0.0	0.0	0.0	0.1	UC	UC	**0.0**
F3P16_RS01700	0.8	1.2	1.4	1.8	3.0	5.7	1.6	3.1	**2.2**
yccS	0.0	0.0	1.4	0.0	0.1	0.1	1.4	1.8	**1.7**
F3P16_RS01710	0.6	1.2	1.8	1.5	1.2	2.0	0.8	1.3	**2.3**
F3P16_RS01715	0.6	1.0	1.6	0.7	1.1	1.1	1.5	1.5	**1.2**
F3P16_RS01720	0.1	0.2	1.7	0.2	0.5	0.4	2.3	2.1	**1.5**
F3P16_RS01725	0.1	0.1	2.0	0.0	0.1	0.3	2.6	7.6	**0.9**
F3P16_RS01730	0.0	0.4	36.2	0.6	0.7	1.1	1.1	1.7	**51.1**
F3P16_RS01735	0.1	0.3	5.2	0.3	0.4	0.6	1.2	1.9	**6.1**
F3P16_RS01740	0.1	0.2	3.6	0.2	0.3	0.5	1.9	3.1	**3.1**
F3P16_RS01745	0.1	0.1	1.5	0.2	0.3	0.5	1.5	2.5	**2.3**
F3P16_RS01750	0.1	0.2	1.6	0.2	0.2	0.6	1.0	3.4	**1.4**

F3P16_RS01755	5.7	19.0	3.3	18.7	25.2	37.0	1.3	2.0	**3.3**
F3P16_RS01760	0.9	4.4	4.9	3.5	5.3	4.6	1.5	1.3	**3.9**
astA	0.5	2.6	5.1	2.1	3.5	3.6	1.7	1.7	**4.2**
astD	1.1	5.0	4.7	3.7	6.2	7.2	1.7	2.0	**3.5**
astB	1.0	4.2	4.1	3.5	6.1	7.7	1.7	2.2	**3.4**
astE	1.1	5.4	4.8	4.3	9.1	12.9	2.1	3.0	**3.8**
F3P16_RS01785	0.7	26.2	39.5	15.5	16.4	28.0	1.1	1.8	**23.3**
F3P16_RS01790	0.0	0.0	UC	0.1	0.1	0.1	1.9	1.0	**UC**
F3P16_RS01795	0.1	0.5	3.4	0.6	0.9	0.8	1.4	1.3	**4.4**
F3P16_RS01800	0.1	0.4	6.6	0.5	0.5	1.5	0.9	2.7	**8.7**
F3P16_RS01805	0.0	0.3	9.8	0.3	0.4	1.0	1.3	3.0	**11.8**
F3P16_RS01810	0.0	0.0	7.9	0.0	0.0	0.1	3.0	6.6	**2.0**
F3P16_RS01815	0.0	0.0	UC	0.0	0.0	0.0	1.0	10.3	**UC**
F3P16_RS01820	0.0	0.0	UC	0.0	0.0	0.0	UC	UC	**UC**
F3P16_RS01825	0.0	0.0	UC	0.0	0.0	0.0	UC	UC	**UC**
glyS	1.0	1.7	1.7	2.0	3.4	5.0	1.7	2.6	**1.9**
F3P16_RS01835	0.0	0.0	UC	0.0	0.0	0.0	UC	UC	**UC**
glyQ	1.8	2.6	1.4	2.3	4.5	5.5	1.9	2.4	**1.3**
F3P16_RS01845	0.0	0.0	UC	0.0	0.0	0.0	UC	UC	**UC**
F3P16_RS01850	0.0	0.0	2.2	0.1	0.0	0.2	0.7	3.2	**3.4**
F3P16_RS01855	0.0	0.0	1.3	0.0	0.0	0.2	0.5	5.9	**1.6**
F3P16_RS01860	0.1	0.1	1.9	0.1	0.1	1.1	1.9	18.3	**1.1**
F3P16_RS01865	0.5	1.3	2.8	2.1	1.4	8.9	0.7	4.3	**4.5**
F3P16_RS01870	0.2	0.4	2.1	0.5	0.8	1.1	1.7	2.3	**2.6**
F3P16_RS01875	1.0	5.2	5.0	3.2	3.4	3.7	1.0	1.1	**3.1**
F3P16_RS01880	0.2	0.3	1.4	0.2	0.4	0.4	2.2	2.7	**0.7**
aroE	0.8	1.2	1.6	0.8	1.0	1.5	1.2	1.8	**1.0**
hemF	1.2	1.8	1.5	1.7	2.1	3.6	1.2	2.1	**1.5**
ribA	0.2	0.3	1.9	0.3	0.4	0.7	1.6	2.7	**1.5**
dxs]	1.0	1.2	1.3	1.3	1.8	2.8	1.3	2.1	**1.4**

F3P16_RS01905	2.3	2.1	0.9	3.4	5.2	5.2	1.5	1.5	**1.5**
F3P16_RS01910	82.3	147.2	1.8	147.8	287.1	315.9	1.9	2.1	**1.8**
F3P16_RS01915	0.0	0.4	9.8	0.5	0.8	0.5	1.6	1.1	**13.2**
F3P16_RS01920	1.9	4.6	2.5	3.6	5.7	7.0	1.6	1.9	**1.9**
mlaE	6.7	16.4	2.4	13.3	21.2	25.0	1.6	1.9	**2.0**
F3P16_RS01930	13.9	58.2	4.2	42.4	60.0	66.7	1.4	1.6	**3.0**
F3P16_RS01935	6.9	100.1	14.5	59.9	66.8	62.9	1.1	1.1	**8.7**
F3P16_RS01940	13.5	269.4	19.9	229.9	256.5	235.0	1.1	1.0	**17.0**
F3P16_RS01945	0.3	0.4	1.5	0.3	0.7	0.9	2.1	2.9	**1.1**
F3P16_RS01950	0.8	1.5	1.8	1.6	2.2	2.7	1.4	1.6	**2.0**
F3P16_RS01955	0.4	0.9	2.2	1.3	1.4	1.6	1.0	1.2	**3.3**
F3P16_RS01960	0.1	0.3	2.8	0.2	0.4	0.4	1.9	1.8	**2.2**
recG	0.1	0.2	2.0	0.2	0.3	0.4	1.4	1.6	**1.8**
F3P16_RS01970	0.1	0.4	3.1	0.5	0.5	1.1	1.0	2.2	**4.3**
adeT	0.0	0.1	4.1	0.1	0.1	0.2	1.1	2.7	**4.3**
plsB	1.0	2.9	2.8	2.4	4.5	5.8	1.9	2.4	**2.4**
F3P16_RS01985	0.1	0.4	2.5	0.4	0.5	0.8	1.3	1.9	**2.7**
F3P16_RS01990	0.4	1.9	4.4	1.4	1.9	2.2	1.4	1.6	**3.1**
F3P16_RS01995	0.5	0.8	1.6	0.8	1.3	1.5	1.6	1.9	**1.5**
yihA	0.6	1.1	1.8	0.9	1.7	1.9	1.9	2.1	**1.4**
F3P16_RS02005	0.1	0.4	2.4	0.4	0.5	0.5	1.1	1.2	**2.9**
F3P16_RS02010	0.1	0.2	1.7	0.1	0.3	0.2	2.3	1.9	**1.4**
F3P16_RS02015	0.4	0.8	2.3	0.6	1.0	1.0	1.7	1.7	**1.7**
F3P16_RS02020	0.6	1.1	1.7	0.6	1.1	1.1	1.9	1.8	**0.9**
F3P16_RS02025	0.0	0.0	2.1	0.0	0.0	0.2	UC	UC	**0.0**
rpsJ	31.0	62.4	2.0	54.7	145.9	161.8	2.7	3.0	**1.8**
rplC	90.0	181.0	2.0	173.4	406.9	482.9	2.3	2.8	**1.9**
rplD	91.3	161.7	1.8	159.7	351.6	443.4	2.2	2.8	**1.7**
rplW	30.3	57.4	1.9	61.2	149.1	169.7	2.4	2.8	**2.0**
rplB	76.5	159.0	2.1	141.3	362.0	460.4	2.6	3.3	**1.8**

rpsS	76.4	154.6	2.0	128.9	317.2	426.7	2.5	3.3	**1.7**
rplV	42.0	92.5	2.2	82.6	194.8	262.2	2.4	3.2	**2.0**
rpsC	136.8	259.5	1.9	202.0	454.9	630.8	2.3	3.1	**1.5**
rplP	224.4	353.8	1.6	293.5	582.5	924.0	2.0	3.1	**1.3**
rpmC	175.5	293.5	1.7	290.0	560.9	901.6	1.9	3.1	**1.7**
rpsQ	92.4	152.5	1.7	144.9	315.4	474.2	2.2	3.3	**1.6**
rplN	28.1	53.3	1.9	49.4	109.2	149.8	2.2	3.0	**1.8**
rplX	55.9	110.7	2.0	104.7	222.7	310.4	2.1	3.0	**1.9**
rplE	65.2	157.1	2.4	151.9	352.0	476.5	2.3	3.1	**2.3**
rpsN	44.9	104.7	2.3	103.0	243.7	283.6	2.4	2.8	**2.3**
rpsH	72.8	161.9	2.2	163.6	348.6	413.8	2.1	2.5	**2.2**
rplF	89.0	176.4	2.0	189.1	354.6	480.4	1.9	2.5	**2.1**
rplR	126.2	182.8	1.4	206.6	362.6	618.4	1.8	3.0	**1.6**
rpsE	42.1	93.9	2.2	103.7	227.2	334.4	2.2	3.2	**2.5**
rpmD	26.6	60.3	2.3	63.1	146.8	207.8	2.3	3.3	**2.4**
rplO	60.9	119.9	2.0	130.1	262.2	422.1	2.0	3.2	**2.1**
secY	20.2	43.1	2.1	45.7	108.6	155.3	2.4	3.4	**2.3**
rpmJ	32.2	66.7	2.1	70.4	169.5	255.6	2.4	3.6	**2.2**
rpsM	70.6	126.3	1.8	136.3	272.8	455.9	2.0	3.3	**1.9**
rpsK	80.0	125.3	1.6	135.2	258.6	459.5	1.9	3.4	**1.7**
rpsD	25.7	42.4	1.7	65.7	119.6	223.8	1.8	3.4	**2.6**
rpoA	19.3	25.5	1.3	51.6	80.2	196.5	1.6	3.8	**2.7**
rplQ	13.2	18.1	1.4	45.1	76.2	214.3	1.7	4.7	**3.4**
F3P16_RS02170	0.1	0.4	3.4	0.4	0.5	0.6	1.1	1.3	**4.2**
F3P16_RS02175	0.0	0.0	1.0	0.0	0.1	0.1	1.4	2.2	**1.6**
F3P16_RS02180	0.0	0.1	5.8	0.1	0.0	0.0	0.5	0.6	**4.0**
F3P16_RS02185	0.0	0.0	UC	0.0	0.0	0.0	UC	UC	**UC**
F3P16_RS02190	0.4	0.7	1.9	0.5	0.3	0.8	0.6	1.4	**1.4**
rrtA	0.1	0.1	2.4	0.1	0.2	0.1	1.2	0.9	**2.7**
F3P16_RS02200	0.2	0.4	2.2	0.3	0.4	0.4	1.1	1.1	**1.9**

F3P16_RS02205	0.1	0.8	6.9	0.6	0.6	1.6	0.9	2.7	**5.1**
F3P16_RS02210	1.6	6.7	4.2	4.0	3.5	9.5	0.9	2.4	**2.5**
F3P16_RS02215	1.0	4.3	4.1	2.1	1.7	5.1	0.8	2.5	**2.0**
rnr]	0.7	1.6	2.3	1.1	1.7	2.7	1.5	2.5	**1.5**
F3P16_RS02225	0.7	0.8	1.1	1.6	1.0	3.1	0.6	2.0	**2.2**
F3P16_RS02230	1.7	1.5	0.9	2.1	2.1	5.7	1.0	2.7	**1.3**
F3P16_RS02235	1.7	1.5	0.9	2.1	2.1	5.7	1.0	2.7	**1.2**
F3P16_RS02240	0.0	0.0	1.3	0.0	0.0	0.1	6.0	22.8	**0.2**
F3P16_RS02245	2.6	3.4	1.3	0.6	0.8	1.5	1.5	2.6	**0.2**
F3P16_RS02250	1.7	1.5	0.9	2.1	2.1	5.7	1.0	2.7	**1.2**
F3P16_RS02255	0.0	0.0	UC	0.0	0.0	0.1	1.5	5.7	**UC**
F3P16_RS02260	0.0	0.1	3.5	0.1	0.3	0.4	2.5	4.0	**2.5**
F3P16_RS02265	0.2	0.2	1.2	0.3	0.2	0.5	0.8	1.6	**1.6**
F3P16_RS02270	0.0	0.0	1.2	0.0	0.0	0.1	2.4	3.6	**1.0**
F3P16_RS02275	0.0	0.0	1.8	0.0	0.0	0.0	0.0	10.3	**0.5**
F3P16_RS02280	0.0	0.1	2.2	0.1	0.1	0.2	1.2	2.5	**1.9**
wrbA	0.0	0.0	3.2	0.1	0.1	0.2	2.5	3.2	**8.9**
F3P16_RS02290	1.0	20.7	20.5	12.1	14.5	13.9	1.2	1.1	**12.0**
F3P16_RS02295	0.2	13.5	56.6	8.3	10.3	9.0	1.2	1.1	**34.9**
F3P16_RS02300	0.0	0.2	5.1	0.1	0.1	0.2	1.4	2.0	**3.0**
F3P16_RS02305	0.0	0.0	1.0	0.0	0.1	0.1	1.3	2.2	**1.2**
ybeY	1.8	2.8	1.5	2.2	4.5	4.1	2.1	1.9	**1.2**
F3P16_RS02315	7.6	12.2	1.6	9.4	17.8	20.6	1.9	2.2	**1.2**
miaB	1.1	1.5	1.3	1.5	3.0	2.4	2.0	1.6	**1.3**
F3P16_RS02325	0.5	3.2	6.8	2.1	2.9	3.5	1.3	1.6	**4.6**
F3P16_RS02330	0.2	0.8	3.3	0.8	1.3	1.6	1.7	2.0	**3.2**
F3P16_RS02335	0.7	1.1	1.5	1.6	1.7	5.4	1.1	3.4	**2.2**
F3P16_RS02340	0.0	0.1	4.5	0.3	0.3	0.4	1.0	1.3	**11.2**
F3P16_RS02345	0.4	0.8	1.9	0.8	0.8	3.3	1.0	3.9	**1.9**
F3P16_RS02350	0.0	0.1	6.7	0.1	0.2	0.2	1.6	2.3	**6.3**

rlmJ	1.7	2.6	1.5	2.6	3.8	6.6	1.4	2.5	**1.5**
pssA	1.5	2.8	1.9	2.3	3.9	4.6	1.7	2.0	**1.5**
F3P16_RS02365	0.1	0.2	2.4	0.2	0.3	0.4	1.9	2.8	**2.0**
F3P16_RS02370	9999.6	10000.5	1.0	9999.9	10000.5	10000.1	1.0	1.0	**1.0**
F3P16_RS02375	2.0	1.5	0.7	1.7	2.4	7.6	1.4	4.4	**0.9**
F3P16_RS02380	0.5	1.0	2.1	1.0	0.8	5.2	0.8	5.3	**2.1**
F3P16_RS02385	13853.9	12855.1	0.9	12098.4	10116.1	11101.8	0.8	0.9	**0.9**
rrf]	1.4	1.5	1.0	2.6	2.6	5.2	1.0	2.0	**1.9**
F3P16_RS02395	0.0	0.0	UC	0.0	0.0	0.0	UC	UC	**UC**
F3P16_RS02400	0.0	0.0	2.5	0.0	0.0	0.0	3.3	3.4	**0.5**
F3P16_RS02405	0.0	0.1	4.9	0.0	0.1	0.2	2.7	5.5	**1.7**
F3P16_RS02410	0.0	0.0	1.0	0.0	0.1	0.1	1.3	1.9	**1.4**
F3P16_RS02415	0.0	0.1	4.5	0.1	0.1	0.2	1.0	1.8	**2.8**
F3P16_RS02420	0.0	0.1	2.4	0.0	0.1	0.1	1.2	2.4	**1.3**
F3P16_RS02425	0.1	0.2	2.9	0.2	0.2	0.2	1.1	1.2	**2.2**
F3P16_RS02430	0.0	0.2	3.4	0.1	0.1	0.1	0.9	0.7	**3.0**
F3P16_RS02435	0.1	0.1	1.7	0.1	0.2	0.2	1.8	1.4	**1.5**
F3P16_RS02440	0.1	0.2	3.1	0.2	0.1	0.2	0.9	1.4	**2.7**
F3P16_RS02445	0.1	0.2	1.7	0.3	0.5	0.6	1.6	2.1	**2.3**
F3P16_RS02450	0.2	0.3	2.0	0.3	0.5	1.0	1.8	3.3	**1.8**
F3P16_RS02455	0.1	0.2	2.2	0.2	0.2	0.4	1.0	1.9	**2.9**
rpsI	3.3	5.5	1.7	10.4	17.4	25.6	1.7	2.5	**3.1**
rplM	10.5	12.3	1.2	17.2	23.1	35.9	1.3	2.1	**1.6**
pdxA	0.5	1.1	2.2	0.9	1.1	1.5	1.2	1.7	**1.7**
rsmA	2.5	4.5	1.8	4.2	6.2	6.6	1.5	1.6	**1.7**
F3P16_RS02480	1.8	2.8	1.6	2.9	4.1	4.6	1.4	1.6	**1.6**
F3P16_RS02485	0.1	0.1	1.2	0.2	0.2	0.2	0.9	1.1	**1.8**
F3P16_RS02490	0.3	0.4	1.5	0.3	0.5	0.6	1.7	2.0	**1.2**
F3P16_RS02495	0.5	1.0	2.0	0.8	1.4	1.8	1.8	2.3	**1.6**
hflX	0.8	1.6	2.1	1.3	2.2	2.3	1.8	1.9	**1.6**

F3P16_RS02505	0.1	0.1	0.7	0.1	0.2	0.3	1.4	2.4	**1.9**
F3P16_RS02510	0.1	0.2	2.4	0.1	0.2	0.3	1.9	2.7	**1.5**
F3P16_RS02515	0.2	0.3	1.3	0.3	0.6	0.7	1.8	2.1	**1.7**
F3P16_RS02520	0.3	0.5	1.6	0.5	0.7	1.0	1.4	2.1	**1.6**
F3P16_RS02525	0.1	0.1	1.0	0.1	0.1	0.1	1.5	2.2	**1.1**
F3P16_RS02530	0.0	0.1	2.1	0.1	0.2	0.2	1.3	1.4	**4.1**
F3P16_RS02535	9999.6	10000.0	1.0	9999.5	10000.1	9999.0	1.0	1.0	**1.0**
F3P16_RS02540	2.0	1.5	0.7	1.7	2.4	7.6	1.4	4.4	**0.9**
F3P16_RS02545	0.5	1.0	2.1	1.0	0.8	5.2	0.8	5.3	**2.1**
F3P16_RS02550	13853.9	12854.7	0.9	12099.0	10115.3	11102.4	0.8	0.9	**0.9**
rrf]	1.4	1.5	1.0	2.6	2.6	5.2	1.0	2.0	**1.9**
F3P16_RS02560	0.1	0.3	4.7	0.3	0.3	0.2	1.1	0.7	**4.2**
F3P16_RS02565	0.1	0.2	1.4	0.2	0.1	0.2	0.7	1.0	**1.6**
F3P16_RS02570	0.0	0.0	1.6	0.1	0.1	0.1	1.5	1.1	**3.0**
F3P16_RS02575	0.1	0.2	1.4	0.1	0.2	0.2	1.5	1.8	**1.0**
trxC	0.6	1.5	2.7	1.2	1.8	1.8	1.5	1.6	**2.0**
F3P16_RS02585	1.0	2.6	2.6	2.6	3.5	3.6	1.3	1.4	**2.6**
F3P16_RS02590	0.7	1.8	2.5	1.6	2.8	2.8	1.8	1.8	**2.2**
F3P16_RS02595	0.1	0.6	4.3	0.7	1.2	1.2	1.8	1.8	**5.1**
F3P16_RS02600	0.1	0.2	2.9	0.1	0.1	0.4	1.0	3.0	**2.2**
mnmC	0.2	0.4	2.1	0.4	0.8	0.8	2.1	2.1	**1.9**
F3P16_RS02610	0.7	1.3	1.9	1.3	1.6	2.0	1.3	1.6	**1.8**
F3P16_RS02615	1.2	1.6	1.4	1.5	2.3	2.9	1.5	1.9	**1.3**
F3P16_RS02620	0.1	0.4	2.4	0.3	0.5	0.7	1.6	2.3	**2.2**
F3P16_RS02625	0.1	0.1	1.3	0.1	0.2	0.4	1.4	3.1	**1.2**
F3P16_RS02630	0.0	0.1	1.4	0.1	0.1	0.2	1.0	2.8	**1.7**
radC	0.0	0.0	UC	0.0	0.0	0.1	0.8	4.3	**UC**
coaB	0.6	1.0	1.7	0.6	1.0	1.6	1.6	2.6	**1.0**
secF	4.0	8.8	2.2	12.9	24.0	36.7	1.9	2.9	**3.2**
secD	3.3	7.3	2.2	8.8	15.8	21.8	1.8	2.5	**2.7**

yajC	4.3	8.3	1.9	10.8	16.2	19.4	1.5	1.8	**2.5**
tgt]	2.5	5.4	2.1	6.7	9.1	11.4	1.4	1.7	**2.6**
F3P16_RS02665	0.1	0.1	1.6	0.0	0.0	0.1	1.4	2.5	**0.6**
F3P16_RS02670	0.0	0.0	0.4	0.0	0.1	0.1	2.1	3.1	**0.8**
queA	0.2	0.3	1.4	0.3	0.4	0.7	1.5	2.7	**1.4**
F3P16_RS02680	0.0	0.0	UC	0.0	0.0	0.0	0.0	0.0	**UC**
F3P16_RS02685	0.0	0.0	UC	0.0	0.0	0.0	UC	UC	**UC**
F3P16_RS02690	0.0	0.0	UC	0.0	0.0	0.0	UC	UC	**UC**
F3P16_RS02695	0.0	0.0	UC	0.0	0.0	0.0	UC	UC	**UC**
F3P16_RS02700	0.0	0.0	UC	0.0	0.0	0.0	UC	UC	**UC**
F3P16_RS02705	0.0	0.0	UC	0.0	0.0	0.0	UC	UC	**UC**
F3P16_RS02710	0.1	0.2	1.4	0.1	0.2	0.2	3.4	3.2	**0.5**
glnE	0.3	0.6	1.7	0.4	0.5	0.9	1.3	2.2	**1.2**
F3P16_RS02720	1.5	2.9	1.9	2.7	3.2	5.2	1.2	1.9	**1.8**
F3P16_RS02725	0.2	0.5	2.3	0.4	0.8	0.6	1.8	1.4	**2.1**
F3P16_RS02730	0.3	0.4	1.4	0.3	0.7	0.6	2.0	1.8	**1.1**
F3P16_RS02735	0.5	0.8	1.6	0.7	1.1	0.9	1.6	1.4	**1.4**
F3P16_RS02740	0.2	0.3	1.6	0.4	0.8	0.6	2.2	1.7	**1.6**
F3P16_RS02745	0.2	0.5	2.3	0.5	0.7	0.8	1.6	1.8	**2.1**
F3P16_RS02750	0.1	0.1	1.2	0.2	0.3	0.3	1.7	2.0	**1.4**
F3P16_RS02755	0.0	0.0	1.7	0.1	0.1	0.1	1.0	1.7	**5.2**
F3P16_RS02760	0.0	0.1	6.3	0.1	0.1	0.1	1.3	1.4	**5.5**
F3P16_RS02765	0.0	0.0	UC	0.0	0.0	0.1	UC	UC	**UC**
F3P16_RS02770	0.0	0.1	UC	0.1	0.2	0.1	2.0	1.2	**UC**
aspS	21.0	35.8	1.7	30.4	45.3	77.8	1.5	2.6	**1.4**
F3P16_RS02780	0.0	0.1	18.5	0.0	0.1	0.3	2.6	8.3	**7.4**
znuD	0.2	1.4	8.8	2.8	5.8	3.2	2.1	1.1	**18.3**
F3P16_RS02790	0.1	0.3	2.6	0.4	0.4	0.7	1.1	1.8	**3.9**
F3P16_RS02795	0.2	0.2	0.9	0.1	0.2	0.4	3.9	7.0	**0.3**
F3P16_RS02800	0.2	13.1	83.1	8.4	7.4	7.9	0.9	0.9	**53.3**

F3P16_RS02805	0.0	0.1	UC	0.1	0.1	0.2	1.1	2.9	**UC**
F3P16_RS02810	0.4	2.2	6.0	0.9	0.7	1.2	0.8	1.4	**2.4**
F3P16_RS02815	1.9	13.5	7.1	6.3	6.3	5.1	1.0	0.8	**3.4**
F3P16_RS02820	0.3	78.1	280.7	67.2	79.1	26.8	1.2	0.4	**241.5**
baeS	0.2	4.2	18.0	1.8	2.4	1.4	1.3	0.8	**7.7**
F3P16_RS02830	0.6	7.1	11.8	3.7	4.0	2.7	1.1	0.7	**6.2**
F3P16_RS02835	0.3	1.4	4.7	1.1	1.2	1.2	1.1	1.1	**3.7**
F3P16_RS02840	0.9	1.5	1.6	1.4	1.8	2.7	1.3	1.9	**1.5**
F3P16_RS02845	0.1	0.2	2.0	0.2	0.4	0.5	1.8	2.7	**2.4**
F3P16_RS02850	0.1	0.3	2.4	0.3	0.3	0.5	1.1	2.0	**2.4**
F3P16_RS02855	0.3	0.8	2.6	0.5	1.0	1.3	2.2	2.8	**1.6**
F3P16_RS02860	0.1	0.1	1.0	0.2	0.2	0.3	1.3	2.2	**2.1**
F3P16_RS02865	0.7	1.3	2.0	0.9	1.6	1.7	1.8	1.9	**1.3**
trpB	1.7	2.7	1.6	2.0	3.4	3.5	1.7	1.7	**1.2**
F3P16_RS02875	0.7	0.8	1.1	0.7	1.1	1.2	1.5	1.7	**1.0**
F3P16_RS02880	0.0	0.0	1.4	0.1	0.1	0.2	1.0	2.5	**2.0**
F3P16_RS02885	0.0	0.0	6.3	0.0	0.1	0.2	3.1	9.4	**2.5**
F3P16_RS02890	0.0	0.1	2.3	0.0	0.1	0.1	1.4	3.0	**1.6**
trpA	9.7	14.1	1.4	16.8	21.4	30.6	1.3	1.8	**1.7**
accD	4.8	6.1	1.3	8.2	9.5	15.7	1.2	1.9	**1.7**
folC	1.7	2.8	1.7	2.2	3.3	4.4	1.5	2.0	**1.3**
F3P16_RS02910	0.9	1.7	1.8	1.2	1.9	3.3	1.5	2.7	**1.3**
F3P16_RS02915	0.0	0.0	2.5	0.1	0.1	0.1	1.2	1.9	**3.8**
F3P16_RS02920	0.0	0.1	10.6	0.0	0.1	0.1	1.5	2.8	**4.8**
F3P16_RS02925	0.1	0.8	10.4	0.7	0.7	1.2	0.9	1.7	**9.6**
secA	2.4	5.0	2.1	5.0	5.9	10.1	1.2	2.0	**2.0**
F3P16_RS02935	0.3	1.2	4.8	2.0	2.7	3.4	1.4	1.7	**7.7**
F3P16_RS02940	1.6	2.3	1.5	1.8	2.0	2.1	1.1	1.2	**1.1**
F3P16_RS02945	0.1	0.2	2.3	0.2	0.2	0.3	1.0	1.5	**2.3**
F3P16_RS02950	0.0	0.0	1.6	0.0	0.0	0.1	2.3	7.3	**0.7**

F3P16_RS02955	0.1	0.1	1.5	0.1	0.2	0.4	1.6	3.0	**2.0**
F3P16_RS02960	0.4	0.7	1.8	0.5	0.7	1.7	1.3	3.1	**1.5**
F3P16_RS02965	0.1	0.2	2.3	0.1	0.2	0.6	1.7	4.0	**1.6**
F3P16_RS02970	0.1	0.3	4.2	0.2	0.2	0.2	1.2	1.1	**2.5**
F3P16_RS02975	0.1	0.1	1.8	0.1	0.1	0.1	1.4	1.3	**1.3**
F3P16_RS02980	1.6	3.4	2.1	2.0	2.4	4.1	1.3	2.1	**1.2**
F3P16_RS02985	0.0	0.1	4.3	0.0	0.1	0.2	1.8	3.9	**1.7**
F3P16_RS02990	2.4	1.2	0.5	2.3	3.4	9.1	1.5	3.9	**1.0**
F3P16_RS02995	4.5	2.6	0.6	4.3	6.8	20.2	1.6	4.7	**1.0**
F3P16_RS03000	0.4	0.5	1.3	0.4	0.6	0.8	1.6	2.2	**0.9**
F3P16_RS03005	0.2	0.2	0.9	0.2	0.2	0.6	1.3	3.5	**0.8**
F3P16_RS03010	0.0	0.0	0.8	0.0	0.0	0.3	1.4	10.3	**1.0**
F3P16_RS03015	0.0	0.2	6.2	0.1	0.1	0.8	1.0	5.8	**4.2**
F3P16_RS03020	0.6	1.1	1.8	0.8	0.9	1.2	1.0	1.4	**1.4**
F3P16_RS03025	0.0	0.1	5.7	0.1	0.1	0.1	1.4	1.6	**5.6**
F3P16_RS03030	20.1	29.7	1.5	39.6	41.6	94.7	1.0	2.4	**2.0**
F3P16_RS18805	0.1	0.2	2.4	0.3	0.3	0.3	1.0	1.1	**3.1**
F3P16_RS03040	0.5	0.7	1.5	0.4	0.7	0.8	1.7	2.0	**0.8**
F3P16_RS03045	1.5	2.0	1.4	1.7	3.0	3.3	1.7	1.9	**1.2**
F3P16_RS03050	0.7	0.8	1.1	0.7	1.6	1.6	2.3	2.3	**0.9**
F3P16_RS03055	0.6	0.8	1.3	0.7	1.2	1.1	1.9	1.7	**1.0**
typA	7.9	8.6	1.1	12.3	18.6	27.1	1.5	2.2	**1.6**
mscL	0.7	1.3	2.0	1.4	2.2	3.6	1.6	2.6	**2.1**
F3P16_RS03070	0.1	0.2	1.8	0.2	0.2	0.3	0.9	1.2	**2.2**
F3P16_RS03075	0.2	0.6	2.4	0.5	0.7	0.8	1.5	1.7	**1.9**
mutM	0.3	0.6	1.8	0.5	0.6	0.9	1.2	1.7	**1.7**
F3P16_RS03085	0.1	0.3	2.8	0.1	0.1	0.3	1.6	3.5	**0.8**
F3P16_RS03090	0.1	0.1	1.4	0.1	0.1	0.3	1.4	2.6	**1.1**
F3P16_RS03095	0.2	1.1	5.0	1.3	0.9	2.3	0.7	1.8	**5.7**
F3P16_RS03100	0.0	0.3	7.7	0.2	0.3	0.3	1.1	1.3	**5.2**

F3P16_RS03105	0.2	1.4	8.4	1.0	1.2	1.6	1.2	1.5	**6.1**
F3P16_RS03110	0.1	0.4	6.5	0.3	0.4	0.6	1.5	2.0	**5.0**
F3P16_RS03115	0.2	0.3	1.5	0.3	0.3	0.4	1.2	1.6	**1.3**
F3P16_RS03120	0.0	0.5	23.2	0.2	0.5	0.4	1.9	1.7	**10.9**
F3P16_RS03125	0.0	0.0	UC	0.0	0.0	0.0	2.0	3.4	**UC**
F3P16_RS03130	0.0	0.0	0.5	0.0	0.0	0.0	1.0	0.0	**0.2**
F3P16_RS03135	0.0	0.1	6.0	0.1	0.2	0.3	2.0	2.3	**7.9**
F3P16_RS03140	2.2	3.9	1.8	3.7	5.0	6.1	1.3	1.6	**1.7**
F3P16_RS03145	0.2	0.4	1.9	0.3	0.6	0.5	1.9	1.7	**1.5**
F3P16_RS03150	0.0	0.1	2.2	0.1	0.1	0.2	0.9	1.7	**2.7**
F3P16_RS03155	0.0	0.0	UC	0.0	0.0	0.1	UC	UC	**UC**
pilG	0.0	0.0	0.4	0.0	0.0	0.0	0.3	2.9	**0.4**
F3P16_RS03165	0.1	0.1	0.7	0.0	0.1	0.2	1.5	6.1	**0.3**
F3P16_RS03170	0.0	0.1	1.3	0.0	0.0	0.2	10.0	58.2	**0.1**
F3P16_RS03175	0.0	0.1	2.5	0.0	0.1	0.1	2.1	4.8	**0.8**
F3P16_RS03180	0.0	0.1	1.9	0.0	0.1	0.1	1.8	2.5	**1.3**
F3P16_RS03185	0.0	0.1	4.6	0.1	0.1	0.1	1.1	1.0	**4.2**
dapE	0.2	0.4	1.7	0.4	0.7	0.7	1.6	1.7	**1.9**
F3P16_RS03195	0.1	1.2	8.8	0.7	0.6	0.7	0.9	1.0	**5.0**
F3P16_RS03200	0.0	0.0	1.4	0.0	0.1	0.1	1.8	4.2	**3.0**
F3P16_RS03205	0.3	0.6	2.0	0.5	0.6	1.8	1.1	3.3	**1.8**
F3P16_RS03210	0.1	0.3	2.3	0.3	0.3	1.0	0.9	3.2	**2.4**
F3P16_RS03215	0.9	1.5	1.7	0.8	1.2	3.2	1.4	3.9	**0.9**
F3P16_RS03220	1.7	2.1	1.2	0.8	1.2	3.6	1.6	4.6	**0.4**
F3P16_RS03225	1.7	2.1	1.2	0.8	1.2	3.6	1.6	4.6	**0.4**
F3P16_RS03230	0.9	1.5	1.7	0.8	1.2	3.2	1.4	3.9	**0.9**
F3P16_RS03235	1.7	2.1	1.2	0.8	1.2	3.6	1.6	4.6	**0.4**
F3P16_RS03240	0.9	1.5	1.7	0.8	1.2	3.2	1.4	3.9	**0.9**
F3P16_RS03245	0.0	0.1	2.2	0.1	0.1	0.6	0.7	4.1	**2.9**
F3P16_RS03250	0.0	0.2	4.7	0.1	0.1	0.2	2.1	3.4	**1.6**

F3P16_RS03255	0.3	0.6	2.3	0.3	0.9	1.0	2.8	3.1	**1.1**
yccS	0.4	2.2	5.1	1.7	2.9	2.5	1.7	1.5	**4.0**
F3P16_RS03265	0.3	0.9	2.8	0.7	1.0	1.6	1.5	2.3	**2.1**
F3P16_RS03270	0.3	0.7	2.7	0.5	0.5	0.8	1.1	1.6	**1.7**
F3P16_RS03275	0.7	0.2	0.3	0.6	1.3	2.5	2.1	4.1	**0.9**
F3P16_RS03280	0.6	0.5	0.8	1.1	1.7	2.7	1.6	2.5	**1.7**
F3P16_RS03285	0.7	0.7	1.0	1.2	2.0	4.1	1.7	3.3	**1.7**
F3P16_RS03290	0.1	0.3	3.2	0.3	0.3	0.7	0.9	2.0	**3.7**
F3P16_RS03295	0.2	0.4	1.7	0.3	0.4	0.7	1.5	2.9	**1.1**
sbmA	0.0	0.0	0.9	0.0	0.0	0.1	0.8	2.8	**2.1**
F3P16_RS03305	0.0	0.0	UC	0.0	0.0	0.0	UC	UC	**UC**
F3P16_RS03310	0.0	0.0	UC	0.0	0.0	0.0	2.7	3.2	**UC**
F3P16_RS03315	0.1	0.1	0.8	0.0	0.0	0.0	1.4	1.8	**0.4**
F3P16_RS03320	0.1	0.3	2.4	0.3	0.3	0.8	1.0	2.4	**2.9**
F3P16_RS03325	0.1	0.2	1.4	0.1	0.1	0.1	1.1	1.3	**0.8**
gatB	3.3	6.0	1.8	4.7	8.6	9.3	1.8	2.0	**1.4**
gatA	4.4	8.7	2.0	5.4	10.5	10.6	2.0	2.0	**1.2**
gatC	1.2	2.4	2.0	1.7	3.6	3.6	2.1	2.0	**1.4**
F3P16_RS03345	2.2	3.5	1.6	4.4	7.0	9.5	1.6	2.1	**2.0**
mreC	1.9	2.5	1.3	2.6	5.0	6.5	1.9	2.5	**1.4**
mreD	1.1	1.2	1.1	1.1	2.3	2.7	2.1	2.4	**1.0**
F3P16_RS03360	0.3	0.4	1.3	0.6	1.1	1.5	1.9	2.5	**1.7**
rng]	0.8	1.3	1.5	0.9	2.0	2.1	2.2	2.2	**1.1**
F3P16_RS03370	2.5	3.7	1.5	0.6	1.1	1.4	1.9	2.4	**0.2**
F3P16_RS03375	0.9	2.1	2.2	1.3	1.5	1.5	1.2	1.2	**1.3**
F3P16_RS03380	0.2	0.4	1.5	0.5	0.5	1.0	1.1	2.1	**1.9**
thrH	0.4	0.9	2.2	1.2	1.3	2.0	1.2	1.7	**2.8**
F3P16_RS03390	1.8	1.6	0.9	2.3	3.7	4.1	1.6	1.8	**1.3**
F3P16_RS03395	0.0	0.1	3.3	0.1	0.1	0.2	0.6	1.9	**5.0**
F3P16_RS03400	0.0	0.0	UC	0.0	0.1	0.1	4.1	5.9	**UC**

F3P16_RS03405	0.0	0.0	6.7	0.0	0.0	0.1	0.9	3.0	**6.0**
F3P16_RS03410	0.1	0.0	0.8	0.1	0.1	0.1	1.9	2.4	**1.0**
F3P16_RS03415	0.0	0.1	2.2	0.2	0.3	0.6	1.7	3.2	**3.5**
F3P16_RS03420	0.1	0.3	4.7	0.3	0.4	0.4	1.5	1.5	**4.3**
F3P16_RS03425	0.3	1.3	4.8	1.2	1.5	1.7	1.3	1.5	**4.1**
hchA	0.2	0.9	5.7	0.7	1.2	0.5	1.6	0.7	**4.5**
F3P16_RS03435	0.2	1.2	7.4	1.0	1.5	0.6	1.5	0.6	**6.4**
F3P16_RS03440	0.6	1.3	2.1	1.0	1.0	1.6	1.0	1.6	**1.6**
prpF	0.3	0.6	2.0	0.6	0.5	0.9	0.9	1.6	**1.9**
F3P16_RS03450	0.2	0.3	1.5	0.2	0.4	0.7	2.0	3.2	**1.2**
F3P16_RS03455	0.0	0.0	1.1	0.0	0.1	0.1	UC	UC	**0.0**
F3P16_RS03460	0.3	0.3	1.0	0.3	0.4	0.6	1.1	1.8	**1.2**
F3P16_RS03465	1.1	1.3	1.2	1.0	1.1	1.8	1.1	1.8	**0.9**
F3P16_RS03470	0.3	0.5	2.1	0.5	0.7	1.0	1.4	1.9	**1.9**
F3P16_RS03475	0.0	0.1	2.9	0.1	0.2	0.2	2.0	2.7	**2.9**
F3P16_RS03480	0.0	0.0	1.8	0.0	0.0	0.1	0.6	4.6	**1.5**
F3P16_RS03485	1.4	1.3	0.9	0.9	1.3	1.9	1.5	2.2	**0.6**
pmrC	0.0	0.1	1.8	0.1	0.2	0.2	2.2	2.1	**1.8**
pmrA	0.0	0.3	5.9	0.2	0.3	0.5	1.4	2.3	**4.7**
pmrB	0.0	0.1	4.0	0.1	0.1	0.2	1.4	2.0	**2.8**
F3P16_RS03505	0.0	0.0	1.3	0.0	0.0	0.1	UC	UC	**0.0**
F3P16_RS03510	0.1	0.1	1.4	0.0	0.1	0.2	1.3	3.8	**0.7**
F3P16_RS03520	0.0	0.0	3.0	0.0	0.0	0.1	3.8	4.6	**0.7**
F3P16_RS03525	0.0	0.0	UC	0.0	0.0	0.1	1.3	3.7	**UC**
F3P16_RS03530	0.0	0.0	2.1	0.0	0.0	0.0	0.0	1.7	**1.0**
F3P16_RS03535	0.0	0.0	UC	0.0	0.0	0.0	UC	UC	**UC**
F3P16_RS03540	1.6	2.6	1.6	2.8	4.0	4.5	1.5	1.6	**1.7**
F3P16_RS03545	0.0	0.1	5.5	0.1	0.0	0.1	0.7	1.7	**4.8**
F3P16_RS03550	0.4	0.6	1.6	0.4	0.6	0.6	1.6	1.5	**1.0**
F3P16_RS03555	0.2	0.3	1.8	0.2	0.3	0.5	2.0	2.8	**0.9**

F3P16_RS03560	0.2	0.4	2.0	0.2	0.4	0.6	1.9	2.8	**0.9**
F3P16_RS03565	0.0	0.1	1.3	0.1	0.2	0.2	1.8	1.8	**2.5**
adeK	2.4	9.1	3.8	10.7	18.7	29.3	1.7	2.7	**4.5**
adeJ	4.9	26.1	5.3	24.7	48.2	56.2	1.9	2.3	**5.0**
adeI	5.6	32.2	5.7	33.2	61.6	66.9	1.9	2.0	**5.9**
F3P16_RS03585	0.7	4.5	6.3	6.8	13.6	14.1	2.0	2.1	**9.5**
F3P16_RS03590	1.0	2.0	2.0	1.3	2.5	2.1	1.9	1.6	**1.3**
F3P16_RS03595	0.6	0.8	1.4	0.7	0.9	1.4	1.3	2.1	**1.2**
F3P16_RS03600	0.0	0.0	1.3	0.0	0.1	0.1	5.5	7.4	**0.5**
rplU	7.3	8.4	1.2	12.5	16.3	28.9	1.3	2.3	**1.7**
rpmA	0.9	1.1	1.1	2.1	3.3	7.8	1.6	3.7	**2.3**
lolA	0.3	18.4	65.6	10.3	8.3	10.8	0.8	1.0	**36.7**
F3P16_RS03620	0.1	0.4	3.4	0.4	0.5	0.6	1.4	1.6	**3.4**
serS	2.3	3.0	1.3	2.6	3.6	4.6	1.4	1.8	**1.1**
cysG	0.7	1.3	1.9	1.6	2.7	2.7	1.7	1.7	**2.3**
F3P16_RS03635	0.6	1.1	1.9	1.5	2.8	3.8	1.9	2.5	**2.5**
F3P16_RS03640	0.0	0.0	UC	0.0	0.0	0.0	UC	UC	**UC**
F3P16_RS03645	0.0	0.0	3.2	0.0	0.0	0.1	0.9	3.7	**6.5**
F3P16_RS03650	0.2	0.4	1.6	0.5	0.8	1.1	1.5	2.1	**2.1**
F3P16_RS03655	1.0	1.9	1.9	1.4	1.4	1.4	1.0	1.0	**1.4**
F3P16_RS03660	1.4	2.7	2.0	2.0	2.1	1.7	1.0	0.9	**1.4**
lipB	0.4	0.8	1.9	0.5	0.6	0.7	1.3	1.5	**1.1**
F3P16_RS03670	0.7	1.9	2.6	1.6	3.0	4.7	1.8	2.9	**2.2**
rpoD	3.8	6.1	1.6	6.2	8.3	11.9	1.3	1.9	**1.6**
F3P16_RS03680	0.1	0.0	0.3	0.1	0.1	0.5	1.2	4.6	**1.8**
F3P16_RS03685	0.3	0.7	2.2	1.4	2.1	3.4	1.6	2.5	**4.2**
F3P16_RS03690	0.3	0.4	1.7	0.4	0.7	1.1	1.7	2.4	**1.7**
F3P16_RS03695	0.0	0.1	1.4	0.1	0.1	0.4	0.5	3.5	**2.3**
gltA	6.4	8.4	1.3	5.7	5.8	11.4	1.0	2.0	**0.9**
sdhC	5.2	9.1	1.7	8.0	14.0	19.9	1.7	2.5	**1.5**

sdhD	16.8	33.6	2.0	35.1	60.8	80.4	1.7	2.3	**2.1**
sdhA	8.2	14.3	1.8	15.6	25.2	38.5	1.6	2.5	**1.9**
F3P16_RS03720	1.6	3.3	2.0	4.6	6.7	13.0	1.5	2.8	**2.8**
F3P16_RS03725	0.4	0.4	1.2	0.6	0.7	0.9	1.1	1.4	**1.7**
F3P16_RS03730	2.3	4.7	2.0	4.3	7.0	6.7	1.6	1.5	**1.9**
F3P16_RS03735	8.1	21.0	2.6	20.6	34.6	34.6	1.7	1.7	**2.6**
odhB	10.3	24.4	2.4	20.8	37.1	33.6	1.8	1.6	**2.0**
lpdA	10.6	23.4	2.2	23.1	41.8	39.7	1.8	1.7	**2.2**
sucC	6.2	13.5	2.2	14.5	29.1	33.1	2.0	2.3	**2.3**
sucD	9.4	15.8	1.7	17.1	30.0	49.1	1.8	2.9	**1.8**
trpS	0.5	0.8	1.6	0.9	1.1	2.0	1.2	2.3	**1.7**
F3P16_RS03765	2.0	4.4	2.2	2.9	3.7	4.3	1.3	1.5	**1.4**
F3P16_RS03770	1.3	2.3	1.8	1.4	2.0	2.6	1.4	1.8	**1.1**
F3P16_RS03775	0.7	1.6	2.3	1.1	1.7	1.3	1.5	1.2	**1.6**
F3P16_RS03780	1.6	2.3	1.4	0.8	1.2	1.8	1.5	2.3	**0.5**
F3P16_RS18995	0.7	1.0	1.5	0.4	0.6	0.5	1.8	1.4	**0.5**
F3P16_RS03835	0.0	0.0	6.6	0.0	0.0	0.0	1.3	1.0	**6.7**
F3P16_RS03840	0.0	0.1	1.8	0.0	0.1	0.1	1.6	1.5	**1.3**
F3P16_RS03845	0.0	0.0	2.8	0.0	0.0	0.0	1.8	1.6	**1.5**
F3P16_RS03850	0.1	0.0	0.8	0.0	0.1	0.1	3.2	3.0	**0.5**
F3P16_RS03855	0.1	0.1	0.7	0.1	0.2	0.2	1.5	1.5	**1.9**
F3P16_RS03860	0.1	0.1	1.0	0.0	0.2	0.1	4.0	2.2	**0.5**
F3P16_RS03865	0.0	0.0	UC	0.0	0.0	0.0	UC	UC	**UC**
F3P16_RS03870	0.0	0.0	UC	0.0	0.0	0.0	UC	UC	**UC**
greA	0.3	0.3	1.2	0.3	0.7	0.7	2.6	2.5	**1.0**
carB	6.5	10.1	1.6	9.8	20.2	17.7	2.1	1.8	**1.5**
carA	10.2	15.2	1.5	13.6	27.8	24.1	2.1	1.8	**1.3**
F3P16_RS03890	0.0	0.1	1.4	0.1	0.1	0.2	1.4	4.6	**1.1**
F3P16_RS03895	1.2	1.8	1.5	1.9	2.6	4.3	1.4	2.3	**1.6**
yhbY	1.1	1.4	1.3	1.7	2.4	3.6	1.4	2.0	**1.6**

rlmE	2.6	3.7	1.4	4.6	8.2	10.4	1.8	2.3	**1.8**
ftsH	12.3	27.1	2.2	30.4	39.2	51.3	1.3	1.7	**2.5**
folP	0.1	0.2	1.4	0.2	0.3	0.4	1.3	1.9	**1.5**
F3P16_RS03920	0.0	0.1	1.3	0.0	0.1	0.2	3.5	5.7	**0.7**
F3P16_RS03925	0.0	0.0	0.7	0.0	0.0	0.1	3.0	12.0	**0.7**
F3P16_RS03930	0.3	0.2	1.0	0.4	0.5	1.0	1.3	2.6	**1.5**
F3P16_RS03935	0.0	0.0	0.7	0.0	0.0	0.0	3.5	9.4	**0.2**
F3P16_RS03940	0.3	0.2	1.0	0.4	0.5	1.0	1.3	2.6	**1.5**
F3P16_RS03945	0.0	0.0	UC	0.1	0.0	0.1	0.5	0.6	**UC**
folD	0.7	1.3	2.0	0.9	1.7	1.8	2.0	2.0	**1.3**
F3P16_RS03955	0.3	0.3	1.2	0.4	0.7	0.7	1.8	1.7	**1.5**
F3P16_RS03960	0.1	0.2	2.4	0.3	0.7	1.0	2.0	2.9	**3.6**
F3P16_RS03965	0.3	1.0	2.9	0.5	0.7	0.9	1.3	1.8	**1.5**
glpK	0.1	0.2	1.8	0.1	0.1	0.3	1.3	2.5	**1.2**
glpD	0.0	0.1	2.3	0.1	0.1	0.2	1.4	1.6	**2.5**
F3P16_RS03980	8.7	14.5	1.7	14.9	26.6	31.4	1.8	2.1	**1.7**
F3P16_RS03985	0.0	0.1	3.4	0.1	0.1	0.1	1.0	1.8	**1.8**
F3P16_RS03990	0.0	0.1	16.9	0.0	0.1	0.1	1.6	2.2	**8.9**
blp1	0.1	0.2	2.1	0.1	0.2	0.4	1.7	3.0	**1.4**
F3P16_RS04000	1.5	2.4	1.6	2.6	5.0	8.5	2.0	3.3	**1.7**
groL	6.9	10.6	1.5	12.4	20.7	46.9	1.7	3.8	**1.8**
F3P16_RS04010	0.0	0.0	UC	0.0	0.0	0.1	1.8	6.0	**UC**
F3P16_RS04015	0.4	0.4	1.0	0.2	0.4	0.4	2.3	2.5	**0.4**
F3P16_RS04020	0.0	0.0	5.3	0.0	0.0	0.0	2.0	2.3	**1.0**
F3P16_RS04025	0.1	1.6	15.7	0.7	0.8	0.6	1.2	0.9	**6.6**
F3P16_RS04030	0.1	0.4	5.0	0.2	0.3	0.4	2.0	2.7	**2.3**
htpX	4.0	9.2	2.3	9.9	11.5	27.0	1.2	2.7	**2.5**
F3P16_RS04040	0.3	0.2	0.6	0.1	0.2	0.2	2.6	3.4	**0.2**
ttcA	0.4	0.7	1.8	0.9	1.2	1.5	1.4	1.7	**2.1**
F3P16_RS04055	0.4	1.5	3.8	2.0	3.0	4.2	1.5	2.1	**5.2**

F3P16_RS04060	0.7	19.2	26.2	10.4	16.0	9.6	1.5	0.9	**14.3**
greB	0.0	0.0	0.3	0.0	0.0	0.1	3.0	17.1	**0.2**
F3P16_RS04070	0.0	0.0	UC	0.0	0.0	0.1	UC	UC	**UC**
F3P16_RS04075	0.0	0.1	4.2	0.1	0.0	0.1	0.8	1.3	**4.5**
F3P16_RS04080	0.0	0.0	1.6	0.0	0.0	0.0	1.0	1.4	**4.7**
F3P16_RS04085	0.0	0.1	UC	0.0	0.0	0.0	0.8	0.9	**UC**
F3P16_RS04090	0.0	0.0	2.2	0.1	0.1	0.1	1.1	1.4	**2.4**
F3P16_RS04095	0.0	0.0	4.4	0.0	0.0	0.1	0.8	6.7	**2.6**
F3P16_RS04100	0.0	0.0	UC	0.0	0.0	0.0	UC	UC	**UC**
F3P16_RS04105	0.0	0.0	UC	0.0	0.0	0.0	UC	UC	**UC**
F3P16_RS04110	0.0	0.0	UC	0.0	0.0	0.0	UC	UC	**UC**
F3P16_RS04115	0.0	0.0	UC	0.0	0.0	0.0	UC	UC	**UC**
F3P16_RS04120	0.0	0.0	UC	0.0	0.0	0.0	UC	UC	**UC**
F3P16_RS04125	0.0	0.0	UC	0.0	0.0	0.0	UC	UC	**UC**
F3P16_RS04130	0.0	0.0	UC	0.0	0.0	0.0	UC	UC	**UC**
F3P16_RS04135	0.0	0.0	UC	0.0	0.0	0.0	UC	UC	**UC**
F3P16_RS04140	0.0	0.0	UC	0.0	0.0	0.0	UC	UC	**UC**
F3P16_RS18830	0.0	0.0	UC	0.0	0.0	0.0	UC	UC	**UC**
F3P16_RS04145	0.0	0.0	UC	0.0	0.0	0.0	UC	UC	**UC**
F3P16_RS04150	0.0	0.0	UC	0.0	0.0	0.0	UC	UC	**UC**
F3P16_RS04155	0.0	0.0	UC	0.0	0.0	0.0	UC	UC	**UC**
F3P16_RS04160	0.0	0.0	UC	0.0	0.0	0.0	UC	UC	**UC**
F3P16_RS04165	0.0	0.0	UC	0.0	0.0	0.0	UC	UC	**UC**
F3P16_RS04170	0.0	0.0	UC	0.0	0.0	0.0	UC	UC	**UC**
F3P16_RS19000	0.0	0.0	UC	0.0	0.0	0.0	UC	UC	**UC**
F3P16_RS04180	0.0	0.0	UC	0.0	0.0	0.0	UC	UC	**UC**
F3P16_RS04185	0.0	0.0	UC	0.0	0.0	0.0	UC	UC	**UC**
F3P16_RS04190	0.0	0.0	UC	0.0	0.0	0.0	UC	UC	**UC**
F3P16_RS04195	0.0	0.0	UC	0.0	0.0	0.0	UC	UC	**UC**
F3P16_RS04200	0.0	0.0	UC	0.0	0.0	0.0	UC	UC	**UC**

F3P16_RS04205	0.0	0.0	1.9	0.0	0.0	0.0	1.7	1.1	**1.6**
F3P16_RS04210	0.0	0.0	UC	0.0	0.0	0.0	UC	UC	**UC**
F3P16_RS04215	0.0	0.0	UC	0.0	0.0	0.0	UC	UC	**UC**
F3P16_RS19005	0.0	0.0	UC	0.0	0.0	0.0	UC	UC	**UC**
F3P16_RS04230	0.0	0.0	UC	0.0	0.0	0.0	UC	UC	**UC**
F3P16_RS04235	0.0	0.0	UC	0.0	0.0	0.0	UC	UC	**UC**
F3P16_RS04240	0.0	0.0	UC	0.0	0.0	0.0	UC	UC	**UC**
F3P16_RS04245	0.0	0.0	UC	0.0	0.0	0.0	UC	UC	**UC**
F3P16_RS04250	0.0	0.0	UC	0.0	0.0	0.0	UC	UC	**UC**
F3P16_RS04255	0.0	0.0	UC	0.0	0.0	0.0	UC	UC	**UC**
F3P16_RS04265	0.0	0.0	UC	0.0	0.0	0.0	UC	UC	**UC**
F3P16_RS04270	0.0	0.0	UC	0.0	0.0	0.0	UC	UC	**UC**
F3P16_RS04275	0.0	0.0	UC	0.0	0.0	0.0	UC	UC	**UC**
F3P16_RS04280	3.7	12.6	3.4	12.2	16.7	23.7	1.4	2.0	**3.3**
F3P16_RS04285	0.5	2.2	4.3	1.6	2.7	2.4	1.6	1.5	**3.2**
astA	0.0	0.0	UC	0.0	0.0	0.0	5.0	6.8	**UC**
astD	0.3	1.7	4.7	1.2	2.1	2.4	1.7	2.0	**3.5**
astE	0.0	0.0	UC	0.0	0.0	0.0	UC	UC	**UC**
F3P16_RS04305	0.0	0.0	UC	0.0	0.0	0.0	UC	UC	**UC**
F3P16_RS04310	0.0	0.0	UC	0.0	0.0	0.0	UC	UC	**UC**
F3P16_RS04315	0.0	0.0	UC	0.0	0.0	0.0	UC	UC	**UC**
F3P16_RS04320	0.0	0.0	UC	0.0	0.0	0.0	UC	UC	**UC**
F3P16_RS04325	0.0	0.0	UC	0.0	0.0	0.0	UC	UC	**UC**
F3P16_RS04330	0.0	0.0	UC	0.0	0.0	0.0	UC	UC	**UC**
F3P16_RS04335	0.0	0.0	UC	0.0	0.0	0.0	UC	UC	**UC**
F3P16_RS04340	0.0	0.0	UC	0.0	0.0	0.0	UC	UC	**UC**
F3P16_RS19010	0.0	0.0	UC	0.0	0.0	0.0	UC	UC	**UC**
F3P16_RS04350	0.0	0.0	UC	0.0	0.0	0.0	UC	UC	**UC**
F3P16_RS04355	0.0	0.0	UC	0.0	0.0	0.0	UC	UC	**UC**
F3P16_RS04360	0.0	0.0	UC	0.0	0.0	0.0	UC	UC	**UC**

F3P16_RS18835	0.0	0.0	UC	0.0	0.0	0.0	UC	UC	**UC**
F3P16_RS18840	0.0	0.0	UC	0.0	0.0	0.0	UC	UC	**UC**
F3P16_RS18845	0.0	0.0	UC	0.0	0.0	0.0	UC	UC	**UC**
F3P16_RS04370	0.0	0.0	UC	0.0	0.0	0.0	UC	UC	**UC**
F3P16_RS04375	0.0	0.0	UC	0.0	0.0	0.0	UC	UC	**UC**
F3P16_RS04380	0.0	0.0	UC	0.0	0.0	0.0	UC	UC	**UC**
F3P16_RS04385	0.0	0.0	UC	0.0	0.0	0.0	UC	UC	**UC**
F3P16_RS04390	0.0	0.0	UC	0.0	0.0	0.0	UC	UC	**UC**
F3P16_RS04395	0.0	0.0	UC	0.0	0.0	0.0	UC	UC	**UC**
F3P16_RS04400	0.0	0.0	UC	0.0	0.0	0.0	UC	UC	**UC**
F3P16_RS04405	0.0	0.1	7.7	0.0	0.0	0.2	1.0	4.0	**3.5**
F3P16_RS04410	0.0	0.1	2.9	0.0	0.1	0.1	1.3	3.3	**1.6**
F3P16_RS04415	0.0	0.0	UC	0.0	0.0	0.0	UC	UC	**UC**
F3P16_RS04420	0.0	0.0	UC	0.0	0.0	0.0	UC	UC	**UC**
F3P16_RS04425	0.0	0.0	UC	0.0	0.0	0.0	UC	UC	**UC**
F3P16_RS04430	0.0	0.0	UC	0.0	0.0	0.0	UC	UC	**UC**
F3P16_RS04435	0.0	0.0	UC	0.0	0.0	0.0	UC	UC	**UC**
F3P16_RS04440	0.0	0.0	UC	0.0	0.0	0.0	UC	UC	**UC**
F3P16_RS04445	0.0	0.0	UC	0.0	0.0	0.0	UC	UC	**UC**
F3P16_RS04450	0.0	0.0	UC	0.0	0.0	0.0	UC	UC	**UC**
F3P16_RS04455	0.0	0.0	UC	0.0	0.0	0.0	UC	UC	**UC**
F3P16_RS04460	0.0	0.0	UC	0.0	0.0	0.0	0.0	2.3	**UC**
F3P16_RS04465	0.4	0.7	1.9	0.7	0.9	1.6	1.2	2.2	**2.0**
F3P16_RS04470	0.0	0.2	5.7	0.2	0.1	0.3	0.9	2.0	**4.5**
F3P16_RS04475	0.0	0.0	1.9	0.1	0.0	0.1	0.5	0.9	**5.6**
F3P16_RS04480	0.0	0.1	1.9	0.0	0.1	0.2	3.2	8.5	**0.6**
F3P16_RS04485	0.7	2.1	3.1	1.9	2.0	1.2	1.1	0.7	**2.7**
fdhD	0.2	0.5	2.4	0.4	0.5	0.2	1.1	0.4	**2.1**
F3P16_RS04495	0.0	0.0	5.3	0.0	0.0	0.1	0.9	4.9	**6.9**
F3P16_RS04500	0.2	0.2	1.3	0.2	0.3	0.4	1.5	2.4	**1.0**

genX	0.3	0.3	1.2	0.3	0.5	0.6	1.5	1.9	**1.2**
F3P16_RS04510	5.5	16.8	3.1	12.5	16.5	22.0	1.3	1.8	**2.3**
radA	1.1	1.9	1.8	1.7	2.5	3.1	1.4	1.8	**1.6**
F3P16_RS04520	1.0	2.1	2.1	1.2	1.3	2.3	1.1	1.8	**1.3**
F3P16_RS04525	0.3	0.6	2.0	0.5	0.7	0.8	1.3	1.4	**2.0**
lysA	0.4	0.8	1.7	1.1	1.5	1.8	1.4	1.7	**2.4**
dapF	0.3	0.7	2.0	0.8	1.0	1.3	1.2	1.6	**2.5**
F3P16_RS04540	0.0	0.0	UC	0.0	0.0	0.0	UC	UC	**UC**
F3P16_RS04545	0.0	0.0	UC	0.0	0.0	0.0	UC	UC	**UC**
F3P16_RS04550	0.1	0.1	1.5	0.1	0.1	0.1	1.7	2.1	**1.0**
F3P16_RS04555	0.1	0.2	2.8	0.1	0.2	0.3	1.4	2.8	**2.3**
F3P16_RS04560	2.9	5.7	2.0	5.9	5.3	14.3	0.9	2.4	**2.1**
F3P16_RS04565	0.8	1.8	2.3	2.4	2.0	6.7	0.8	2.8	**3.1**
gyrA	2.9	5.5	1.9	5.9	8.6	9.2	1.5	1.6	**2.0**
F3P16_RS04575	0.0	0.0	3.0	0.0	0.0	0.0	0.9	2.3	**2.0**
F3P16_RS04580	0.0	0.0	UC	0.0	0.0	0.0	0.8	2.3	**UC**
F3P16_RS04585	0.0	0.0	UC	0.0	0.0	0.0	2.8	2.3	**UC**
F3P16_RS04590	0.0	0.0	UC	0.0	0.0	0.0	UC	UC	**UC**
F3P16_RS04595	0.0	0.1	2.4	0.1	0.1	0.1	1.4	1.3	**2.6**
F3P16_RS04600	0.0	0.2	5.4	0.1	0.2	0.2	1.4	1.3	**3.3**
F3P16_RS04605	0.2	0.5	2.7	0.3	0.4	0.4	1.7	1.7	**1.6**
F3P16_RS04610	0.0	0.2	3.9	0.1	0.2	0.3	2.2	3.7	**1.5**
serC	0.1	0.2	2.7	0.2	0.2	0.4	1.2	2.0	**3.1**
F3P16_RS18850	0.0	0.0	UC	0.0	0.0	0.1	0.7	2.7	**UC**
F3P16_RS04620	0.1	0.6	4.4	0.3	0.5	1.7	1.5	5.6	**2.4**
F3P16_RS04625	0.3	0.5	1.7	0.3	0.7	0.7	2.5	2.5	**0.9**
F3P16_RS04630	0.3	0.6	1.7	0.4	0.8	0.6	2.1	1.7	**1.1**
F3P16_RS04635	0.8	9.4	11.8	5.7	8.4	6.2	1.5	1.1	**7.1**
lolD	0.2	2.3	9.9	1.8	2.7	2.3	1.5	1.2	**8.1**
F3P16_RS04645	0.0	0.1	2.7	0.0	0.0	0.1	2.1	4.0	**1.0**

F3P16_RS04650	0.2	0.3	1.4	0.2	0.3	0.5	1.3	2.2	**1.1**
sppA	0.7	1.0	1.4	1.0	1.7	2.1	1.6	2.1	**1.4**
F3P16_RS04660	0.6	1.0	1.8	1.2	1.9	2.9	1.6	2.4	**2.2**
purN	2.7	2.9	1.1	3.9	6.3	9.2	1.6	2.3	**1.5**
purM	2.5	3.3	1.3	4.3	6.0	8.8	1.4	2.0	**1.7**
F3P16_RS04675	1.5	3.7	2.4	3.1	4.0	4.4	1.3	1.4	**2.1**
hda]	0.2	0.6	2.5	0.5	0.6	0.6	1.1	1.1	**2.3**
rbtA	0.1	0.1	1.7	0.1	0.1	0.1	1.1	1.4	**1.2**
F3P16_RS04690	0.0	0.0	UC	0.0	0.0	0.0	0.5	2.3	**UC**
F3P16_RS04695	0.2	0.8	3.6	0.7	0.9	0.9	1.4	1.4	**2.9**
F3P16_RS04700	0.6	2.1	3.7	1.7	1.7	2.6	1.0	1.6	**2.9**
F3P16_RS04705	0.5	2.3	4.3	1.6	1.6	2.4	1.0	1.5	**3.0**
F3P16_RS04710	0.3	0.9	3.6	0.7	1.5	1.2	2.2	1.8	**2.6**
F3P16_RS04715	1.9	4.8	2.5	4.0	6.6	6.9	1.7	1.7	**2.1**
F3P16_RS04720	0.0	0.2	UC	0.2	0.4	0.3	2.1	1.7	**UC**
pal]	5.7	24.2	4.3	22.1	27.4	31.4	1.2	1.4	**3.9**
tolB	0.9	5.0	5.6	3.4	5.2	6.2	1.5	1.8	**3.9**
tolA	0.5	2.3	4.6	2.1	2.9	4.2	1.4	2.0	**4.3**
tolR	1.4	4.0	2.9	3.9	4.3	7.4	1.1	1.9	**2.8**
tolQ	0.7	2.5	3.3	1.9	3.0	4.1	1.5	2.1	**2.6**
ybgC	0.3	0.6	1.8	0.6	0.8	1.0	1.3	1.5	**2.0**
F3P16_RS04755	0.0	0.0	5.6	0.0	0.0	0.1	0.5	2.3	**3.5**
ruvB	1.3	2.8	2.2	1.9	2.9	3.1	1.5	1.6	**1.5**
ruvA	1.3	3.4	2.7	2.4	3.7	3.4	1.6	1.4	**1.8**
F3P16_RS04770	1.4	3.8	2.8	1.8	2.8	2.5	1.6	1.4	**1.3**
purL	1.4	2.2	1.6	2.4	4.1	4.1	1.7	1.7	**1.7**
F3P16_RS04780	0.0	0.0	2.0	0.0	0.1	0.1	2.7	1.8	**2.4**
F3P16_RS04785	0.1	0.1	1.6	0.1	0.2	0.2	3.0	4.2	**0.6**
F3P16_RS04790	0.3	0.9	3.0	0.4	0.5	0.5	1.4	1.4	**1.3**
F3P16_RS04795	0.1	0.3	4.2	0.2	0.4	0.5	1.8	2.2	**3.4**

F3P16_RS04800	0.0	0.1	1.3	0.0	0.2	0.1	5.9	3.4	**0.6**
F3P16_RS04805	0.3	0.7	2.5	1.1	1.0	1.4	0.9	1.3	**3.7**
F3P16_RS04810	0.2	0.6	3.4	0.7	1.0	1.4	1.4	2.0	**4.1**
F3P16_RS04815	1.2	4.1	3.3	8.9	12.4	14.4	1.4	1.6	**7.3**
queC	4.0	6.3	1.6	6.9	12.7	14.2	1.8	2.1	**1.7**
queE	6.1	9.8	1.6	9.3	17.1	16.0	1.8	1.7	**1.5**
dapD	3.4	6.7	2.0	6.1	11.0	11.2	1.8	1.8	**1.8**
carO	2.6	10.9	4.3	14.6	16.5	11.2	1.1	0.8	**5.7**
F3P16_RS04840	0.6	1.1	1.8	0.7	1.4	1.1	2.0	1.6	**1.2**
F3P16_RS04845	0.3	0.3	0.9	0.2	0.4	0.4	2.2	2.0	**0.6**
cysW	0.2	0.2	0.9	0.1	0.2	0.1	2.6	1.9	**0.3**
cysT	0.1	0.1	1.0	0.1	0.1	0.2	1.2	2.9	**0.5**
F3P16_RS04860	0.7	0.4	0.6	0.2	0.2	0.2	1.0	1.0	**0.3**
F3P16_RS04865	0.3	0.2	0.8	0.1	0.1	0.2	2.1	2.6	**0.3**
pabC	0.0	0.1	2.7	0.0	0.1	0.2	2.2	3.9	**1.9**
mltG	0.3	0.4	1.7	0.4	0.6	0.6	1.6	1.6	**1.5**
tmk]	0.1	0.1	1.0	0.1	0.3	0.4	2.5	3.3	**1.2**
F3P16_RS04885	0.1	0.2	2.1	0.3	0.4	0.5	1.5	1.9	**2.4**
nadB	0.4	0.9	2.2	1.0	1.5	2.3	1.4	2.2	**2.5**
F3P16_RS04895	0.2	1.0	4.4	1.1	1.2	4.3	1.0	3.8	**5.0**
F3P16_RS04900	0.1	0.1	1.8	0.1	0.2	0.2	1.7	1.8	**1.8**
F3P16_RS04905	0.0	0.0	UC	0.0	0.0	0.0	UC	UC	**UC**
lepA	2.8	4.1	1.5	4.1	7.0	6.5	1.7	1.6	**1.5**
lepB	1.4	2.1	1.5	2.8	4.0	3.6	1.4	1.3	**2.0**
F3P16_RS04920	0.6	1.1	1.7	1.0	1.8	2.6	1.8	2.5	**1.6**
rnc]	0.4	0.8	1.9	0.7	1.7	2.2	2.3	3.1	**1.8**
era]	1.5	2.2	1.5	2.6	4.9	4.9	1.9	1.9	**1.7**
F3P16_RS04935	1.4	2.8	2.0	2.7	4.2	3.7	1.6	1.4	**1.9**
recO	0.9	1.5	1.7	1.4	3.4	2.3	2.4	1.6	**1.7**
pdxJ	1.8	2.4	1.3	2.7	3.8	4.8	1.4	1.8	**1.5**

F3P16_RS04950	0.3	0.5	1.5	0.4	0.8	1.1	2.0	2.5	**1.4**
F3P16_RS04955	0.1	0.1	1.7	0.1	0.2	0.3	2.0	2.5	**1.2**
F3P16_RS04960	0.1	0.1	2.1	0.2	0.1	0.7	0.8	3.9	**2.6**
F3P16_RS04965	0.0	0.0	UC	0.0	0.0	0.0	UC	UC	**UC**
F3P16_RS04970	0.0	0.1	6.3	0.1	0.1	0.2	2.2	2.9	**2.6**
F3P16_RS04975	0.1	0.3	4.8	0.2	0.2	0.3	1.1	1.7	**3.1**
F3P16_RS04980	0.0	0.0	0.0	0.0	0.0	0.0	UC	UC	**0.0**
F3P16_RS04985	0.0	0.0	UC	0.0	0.0	0.0	UC	UC	**UC**
F3P16_RS04990	0.0	0.0	UC	0.0	0.0	0.0	UC	UC	**UC**
F3P16_RS04995	0.5	1.2	2.4	1.0	1.8	2.8	1.8	2.8	**1.9**
F3P16_RS05000	0.0	0.1	UC	0.1	0.3	0.2	2.4	1.3	**UC**
F3P16_RS05005	0.0	0.1	1.8	0.1	0.1	0.1	1.0	1.6	**1.7**
F3P16_RS05010	0.1	0.1	2.3	0.1	0.2	0.3	1.3	2.4	**2.2**
uvrB	2.1	4.8	2.3	3.6	3.6	7.8	1.0	2.2	**1.7**
F3P16_RS05020	0.3	1.3	4.0	1.2	1.6	2.7	1.3	2.3	**3.5**
rarD	0.0	0.1	1.9	0.1	0.1	0.2	1.2	3.5	**1.1**
F3P16_RS05030	2.9	5.2	1.8	6.5	9.9	10.8	1.5	1.7	**2.2**
F3P16_RS05035	0.0	0.0	2.1	0.1	0.0	0.1	0.6	1.3	**6.4**
F3P16_RS05040	0.0	0.0	UC	0.1	0.1	0.1	1.0	1.5	**UC**
cgtA	12.9	14.6	1.1	12.5	18.4	20.0	1.5	1.6	**1.0**
proB	2.1	3.7	1.8	3.8	6.5	10.3	1.7	2.7	**1.8**
F3P16_RS05055	0.1	0.4	3.1	0.3	0.4	0.4	1.3	1.2	**2.4**
F3P16_RS05060	0.3	0.7	2.3	0.7	0.9	0.9	1.2	1.2	**2.6**
dusB	0.6	0.9	1.4	1.1	1.4	1.4	1.3	1.3	**1.7**
F3P16_RS05070	0.2	0.3	2.0	0.2	0.2	0.4	1.2	1.9	**1.3**
F3P16_RS05075	0.0	0.0	0.5	0.0	0.0	0.1	0.9	3.1	**1.1**
F3P16_RS05080	0.0	0.2	5.0	0.2	0.3	0.3	1.4	1.4	**6.2**
wecB	0.2	0.7	4.0	0.5	0.6	0.4	1.3	0.9	**2.9**
F3P16_RS05090	0.1	0.1	1.8	0.1	0.1	0.1	1.2	1.0	**1.9**
F3P16_RS05095	0.2	0.3	1.7	0.3	0.5	0.3	1.5	0.9	**1.7**

F3P16_RS05100	0.1	0.3	2.6	0.2	0.3	0.3	1.5	1.2	**2.2**
F3P16_RS05105	0.0	0.1	3.2	0.1	0.1	0.1	1.6	1.5	**2.9**
F3P16_RS05110	0.0	0.1	UC	0.1	0.1	0.1	1.0	2.7	**UC**
F3P16_RS05115	0.0	0.0	4.8	0.0	0.0	0.0	0.6	2.5	**3.5**
F3P16_RS05120	0.7	1.0	1.4	1.4	2.2	5.0	1.5	3.5	**2.0**
F3P16_RS05125	0.1	0.4	5.5	0.7	1.1	0.7	1.5	0.9	**11.2**
F3P16_RS05130	0.4	0.9	2.0	0.7	1.3	1.5	1.8	2.1	**1.6**
F3P16_RS05135	0.4	0.9	2.0	1.0	1.4	1.9	1.4	1.9	**2.3**
F3P16_RS05140	7.5	12.0	1.6	12.1	20.4	25.9	1.7	2.1	**1.6**
F3P16_RS05145	0.8	1.1	1.4	0.9	1.5	2.1	1.6	2.2	**1.2**
icd]	0.9	1.2	1.5	1.7	1.8	1.4	1.1	0.8	**2.0**
F3P16_RS05155	0.0	0.0	UC	0.0	0.0	0.0	0.0	13.7	**UC**
F3P16_RS05160	0.0	0.0	8.4	0.0	0.0	0.1	1.0	4.0	**4.0**
F3P16_RS05165	0.0	0.0	UC	0.0	0.0	0.0	UC	UC	**UC**
F3P16_RS05170	0.0	0.1	8.0	0.0	0.1	0.1	2.0	3.6	**3.8**
F3P16_RS05175	0.4	0.8	1.8	0.6	1.0	1.2	1.6	2.0	**1.4**
F3P16_RS05180	0.9	1.7	1.7	1.6	1.9	2.8	1.2	1.8	**1.7**
F3P16_RS05185	0.0	0.1	3.6	0.0	0.1	0.1	2.7	1.5	**2.2**
F3P16_RS05190	1.2	2.8	2.2	2.1	3.9	3.7	1.9	1.8	**1.6**
F3P16_RS05195	0.0	0.1	4.5	0.2	0.4	0.3	1.8	1.6	**7.1**
F3P16_RS05200	0.1	0.1	1.1	0.1	0.1	0.2	1.3	2.9	**1.1**
F3P16_RS05205	0.2	0.3	1.7	0.2	0.2	0.4	1.3	2.1	**0.9**
F3P16_RS05210	0.0	0.0	UC	0.0	0.1	0.2	1.3	3.9	**UC**
thiE	0.2	0.3	1.4	0.5	0.4	0.6	0.9	1.3	**2.3**
hemL	1.6	2.7	1.7	2.2	3.3	3.3	1.5	1.5	**1.4**
F3P16_RS05225	0.1	0.3	2.9	0.3	0.5	0.9	1.8	3.4	**2.4**
F3P16_RS05230	0.3	0.9	2.8	0.8	1.7	2.4	2.2	3.1	**2.4**
rapA	1.6	3.8	2.4	4.2	7.5	5.6	1.8	1.4	**2.6**
F3P16_RS05240	0.0	0.0	1.5	0.0	0.1	0.1	3.9	6.5	**0.7**
fabR	0.1	0.1	1.3	0.1	0.2	0.2	1.5	1.3	**1.1**

F3P16_RS05250	3.4	4.6	1.4	10.1	7.6	6.6	0.7	0.7	**3.0**
F3P16_RS05255	2.3	2.6	1.1	7.0	5.6	5.0	0.8	0.7	**3.0**
F3P16_RS05260	0.0	0.0	UC	0.0	0.0	0.0	UC	UC	**UC**
F3P16_RS05265	0.0	0.0	UC	0.0	0.0	0.0	UC	UC	**UC**
F3P16_RS05270	0.1	3.4	45.6	2.0	2.7	1.2	1.3	0.6	**27.1**
F3P16_RS05275	5.8	16.7	2.9	22.3	31.7	40.2	1.4	1.8	**3.8**
F3P16_RS05280	3.9	11.7	3.0	15.9	23.8	35.8	1.5	2.3	**4.1**
F3P16_RS05285	0.1	0.1	2.2	0.1	0.1	0.1	1.1	1.2	**1.9**
F3P16_RS05290	0.0	0.0	UC	0.0	0.0	0.0	UC	UC	**UC**
F3P16_RS05295	0.0	0.1	2.1	0.0	0.0	0.0	1.0	3.3	**0.3**
F3P16_RS05300	0.0	0.0	1.1	0.0	0.0	0.0	3.0	13.7	**0.1**
F3P16_RS05305	0.1	0.2	1.7	0.4	0.6	0.5	1.6	1.4	**3.4**
pstC	0.5	0.9	1.7	1.1	1.8	1.0	1.7	0.9	**2.2**
pstA	0.3	0.7	2.1	0.7	1.6	0.9	2.2	1.2	**2.2**
pstB	0.1	0.1	1.4	0.2	0.3	0.2	1.8	1.2	**3.4**
sohB	0.4	1.4	3.2	1.9	2.1	6.0	1.1	3.2	**4.3**
blp2	0.3	0.7	2.6	0.3	0.4	0.5	1.6	1.9	**1.0**
F3P16_RS05335	0.0	0.0	UC	0.0	0.0	0.0	2.8	0.8	**UC**
purB	0.8	1.1	1.5	1.3	2.5	3.5	1.9	2.6	**1.8**
hflD	0.5	0.7	1.4	0.9	1.7	1.1	1.9	1.2	**1.9**
mnmA	0.6	0.9	1.4	0.9	1.5	1.6	1.6	1.7	**1.5**
F3P16_RS05355	0.4	0.4	1.2	0.5	0.8	1.0	1.7	2.1	**1.2**
F3P16_RS05360	0.1	0.3	2.1	0.2	0.4	0.4	1.6	1.9	**1.8**
dacC	0.9	1.0	1.2	1.1	1.6	2.0	1.4	1.8	**1.3**
F3P16_RS05370	0.1	1.0	8.8	1.4	1.4	1.3	1.0	0.9	**13.0**
F3P16_RS05375	0.0	0.0	3.5	0.0	0.0	0.1	0.8	3.1	**3.3**
surE	0.2	0.4	1.7	0.3	0.4	0.6	1.2	2.0	**1.5**
F3P16_RS05385	0.8	2.4	3.1	2.1	3.1	3.4	1.5	1.6	**2.7**
F3P16_RS05390	2.2	2.9	1.3	2.1	3.9	5.0	1.9	2.4	**1.0**
F3P16_RS05395	3.2	8.9	2.8	4.3	5.6	4.4	1.3	1.0	**1.3**

F3P16_RS05400	0.3	1.0	2.9	1.1	1.6	1.2	1.5	1.1	**3.2**
gloA	0.2	0.4	2.3	0.4	0.7	0.7	1.7	1.7	**2.7**
F3P16_RS05410	0.1	0.2	1.7	0.2	0.2	0.3	0.9	1.5	**2.4**
rpmE	0.5	0.7	1.4	1.6	2.6	3.6	1.7	2.3	**3.2**
F3P16_RS05420	0.2	0.2	1.1	0.2	0.4	0.4	2.3	2.2	**1.2**
F3P16_RS05425	0.1	0.1	2.2	0.1	0.2	0.2	2.0	2.6	**1.4**
epmB	0.2	0.3	1.5	0.3	0.5	0.7	1.6	2.3	**1.3**
efp]	5.2	4.7	0.9	5.9	8.1	18.2	1.4	3.1	**1.1**
F3P16_RS05440	0.9	1.5	1.8	1.1	1.1	1.5	1.0	1.4	**1.2**
F3P16_RS05445	0.1	0.2	2.0	0.2	0.1	0.2	0.5	0.9	**2.8**
F3P16_RS05450	0.0	0.0	UC	0.0	0.0	0.0	UC	UC	**UC**
F3P16_RS05455	0.0	0.0	UC	0.0	0.0	0.0	UC	UC	**UC**
F3P16_RS05460	0.0	0.0	UC	0.0	0.0	0.0	UC	UC	**UC**
F3P16_RS05465	0.0	0.0	UC	0.0	0.0	0.0	UC	UC	**UC**
F3P16_RS05470	0.0	0.0	UC	0.0	0.0	0.0	UC	UC	**UC**
F3P16_RS05475	0.0	0.0	UC	0.0	0.0	0.0	UC	UC	**UC**
F3P16_RS05480	0.0	0.0	UC	0.0	0.0	0.0	UC	UC	**UC**
F3P16_RS05485	0.0	0.1	8.4	0.0	0.0	0.0	0.3	1.5	**2.0**
F3P16_RS05490	0.0	0.0	UC	0.0	0.0	0.0	UC	UC	**UC**
F3P16_RS05495	0.0	0.0	UC	0.0	0.0	0.0	UC	UC	**UC**
F3P16_RS05500	0.0	0.0	UC	0.0	0.0	0.0	UC	UC	**UC**
F3P16_RS05505	0.0	0.0	UC	0.0	0.0	0.0	UC	UC	**UC**
F3P16_RS05510	0.0	0.0	UC	0.0	0.0	0.0	UC	UC	**UC**
F3P16_RS05515	0.0	0.0	UC	0.0	0.0	0.0	UC	UC	**UC**
F3P16_RS05520	0.0	0.0	UC	0.0	0.0	0.0	UC	UC	**UC**
F3P16_RS05525	0.0	0.0	UC	0.0	0.0	0.0	UC	UC	**UC**
F3P16_RS05530	0.0	0.0	UC	0.0	0.0	0.0	UC	UC	**UC**
F3P16_RS19015	0.0	0.1	1.8	0.1	0.1	0.4	1.1	7.0	**2.0**
F3P16_RS05540	0.0	0.0	UC	0.0	0.0	0.0	UC	UC	**UC**
F3P16_RS18855	0.0	0.1	10.6	0.2	0.2	0.4	1.2	2.4	**12.4**

F3P16_RS05545	0.0	0.2	9.2	0.3	0.3	0.6	1.2	2.4	**10.8**
F3P16_RS05550	0.0	0.0	UC	0.0	0.0	0.1	1.0	26.2	**UC**
F3P16_RS05555	0.0	0.0	4.2	0.0	0.0	0.0	3.0	9.1	**1.0**
bauF	0.0	0.0	2.6	0.0	0.0	0.0	UC	UC	**0.0**
basA	0.0	0.0	16.9	0.0	0.0	0.0	2.3	2.9	**10.9**
basB	0.0	0.0	2.7	0.0	0.0	0.0	1.1	1.2	**2.7**
bauD	0.0	0.0	0.5	0.0	0.0	0.0	0.0	2.3	**3.0**
bauC	0.0	0.0	UC	0.0	0.0	0.0	0.7	5.3	**UC**
bauE	0.0	0.0	0.5	0.0	0.0	0.0	4.0	6.3	**0.1**
bauB	0.0	0.0	0.5	0.0	0.0	0.0	2.1	1.3	**0.7**
F3P16_RS05595	0.0	0.0	6.3	0.0	0.0	0.0	1.0	0.3	**4.0**
basC	0.0	0.0	0.5	0.0	0.0	0.0	0.8	1.1	**1.1**
basD	0.0	0.0	1.2	0.0	0.0	0.1	1.1	2.8	**1.2**
basE	0.0	0.0	6.3	0.0	0.0	0.0	1.5	11.1	**2.0**
basF	0.0	0.1	1.6	0.0	0.0	0.0	7.5	6.3	**0.1**
basG	0.0	0.0	1.6	0.0	0.0	0.0	18.1	30.8	**0.1**
barA	0.0	0.0	0.9	0.0	0.0	0.1	1.2	1.8	**0.9**
barB	0.1	0.1	1.4	0.1	0.2	0.2	1.6	1.5	**1.5**
basH	0.0	0.0	0.6	0.0	0.0	0.0	2.5	6.3	**0.3**
basI	0.0	0.0	0.0	0.0	0.0	0.0	UC	UC	**0.0**
basJ	0.0	0.0	1.8	0.0	0.0	0.0	4.3	6.8	**0.7**
F3P16_RS05655	1.5	4.4	3.0	4.2	5.1	7.1	1.2	1.7	**2.8**
hemE	0.4	0.5	1.4	0.6	0.7	0.9	1.1	1.5	**1.5**
F3P16_RS05665	0.0	0.1	2.4	0.1	0.2	0.1	2.1	1.4	**2.5**
F3P16_RS05670	0.5	1.1	2.1	0.4	0.6	0.8	1.5	1.9	**0.8**
F3P16_RS05675	0.7	1.0	1.3	0.5	0.9	0.6	1.6	1.1	**0.7**
F3P16_RS05680	4.5	8.3	1.9	9.2	13.9	13.6	1.5	1.5	**2.1**
folE	1.0	1.3	1.3	1.0	2.0	2.3	2.1	2.4	**1.0**
xdhA	0.0	0.1	2.6	0.0	0.1	0.1	2.1	2.6	**0.9**
xdhB	0.1	0.1	2.2	0.1	0.2	0.2	1.6	1.4	**1.7**

xdhC	0.1	0.1	1.2	0.1	0.1	0.2	1.1	2.3	**1.4**
F3P16_RS05705	0.4	0.7	1.8	0.8	1.6	2.0	2.1	2.5	**1.8**
trpC	0.9	1.2	1.3	1.6	2.8	2.4	1.8	1.5	**1.7**
trpD	2.1	3.3	1.5	2.4	5.1	3.1	2.2	1.3	**1.1**
F3P16_RS05720	0.0	0.0	1.7	0.0	0.0	0.1	0.9	2.7	**1.5**
F3P16_RS05725	0.3	0.6	1.9	0.4	0.7	0.5	2.0	1.3	**1.1**
F3P16_RS05730	0.7	1.6	2.2	1.4	1.8	1.9	1.3	1.3	**2.1**
F3P16_RS05735	0.4	1.4	3.7	1.2	1.2	1.8	1.0	1.5	**3.2**
F3P16_RS05740	0.0	0.3	8.4	0.2	0.2	0.3	0.8	1.4	**7.1**
glnA	35.6	47.1	1.3	26.9	45.2	50.7	1.7	1.9	**0.8**
F3P16_RS05750	0.2	0.1	0.8	0.1	0.2	0.3	2.0	3.2	**0.6**
ubiA	0.1	0.1	0.7	0.1	0.2	0.2	1.2	1.8	**1.1**
F3P16_RS05760	0.0	0.0	4.2	0.0	0.0	0.0	0.7	2.3	**3.0**
F3P16_RS05765	0.0	0.0	UC	0.0	0.0	0.1	UC	UC	**UC**
F3P16_RS05770	0.0	0.0	1.1	0.0	0.0	0.0	0.9	0.1	**2.5**
F3P16_RS05775	0.1	0.1	2.1	0.2	0.1	0.7	0.8	3.9	**2.6**
F3P16_RS05780	0.1	0.1	2.1	0.2	0.1	0.5	0.8	2.7	**2.6**
F3P16_RS05785	0.4	1.2	2.9	1.8	2.8	3.0	1.6	1.7	**4.2**
F3P16_RS05790	0.1	0.1	1.2	0.1	0.2	0.2	2.4	2.8	**1.0**
F3P16_RS05795	0.5	0.7	1.5	0.8	1.0	1.6	1.3	2.0	**1.7**
F3P16_RS05800	0.9	2.1	2.4	1.5	1.8	2.0	1.2	1.4	**1.7**
F3P16_RS05805	1.5	2.7	1.8	2.3	3.9	5.2	1.7	2.2	**1.6**
F3P16_RS05810	0.0	0.0	3.6	0.0	0.0	0.0	3.9	3.7	**1.5**
F3P16_RS05815	0.1	0.2	1.1	0.2	0.4	0.5	2.0	2.2	**1.4**
metF	0.7	1.3	1.8	1.5	3.2	3.9	2.1	2.6	**2.1**
ahcY	3.3	4.2	1.3	5.4	10.0	17.6	1.9	3.3	**1.6**
F3P16_RS05830	0.1	0.2	1.9	0.2	0.3	0.1	1.5	0.7	**1.4**
F3P16_RS05835	1.1	1.8	1.7	1.3	1.8	1.3	1.4	1.0	**1.2**
F3P16_RS05840	3.7	9.9	2.7	6.3	7.4	6.7	1.2	1.1	**1.7**
lipA	1.8	2.0	1.1	2.6	2.9	4.5	1.1	1.8	**1.4**

sthA	0.9	1.7	1.9	2.2	2.8	3.4	1.3	1.6	**2.4**
F3P16_RS05855	0.1	0.1	1.0	0.1	0.1	0.1	2.0	2.0	**1.1**
F3P16_RS05860	0.0	0.1	5.7	0.0	0.1	0.2	1.1	3.4	**2.6**
F3P16_RS05865	0.0	0.2	28.8	0.2	0.2	0.1	0.9	0.8	**32.0**
map]	0.5	0.8	1.6	0.8	1.0	1.2	1.3	1.4	**1.6**
rpsB	12.3	17.3	1.4	33.8	48.0	98.1	1.4	2.9	**2.7**
tsf]	5.0	7.1	1.4	23.1	35.2	77.0	1.5	3.3	**4.6**
F3P16_RS05885	0.2	0.3	1.3	0.3	0.5	0.4	1.9	1.4	**1.2**
F3P16_RS05890	0.0	0.0	1.2	0.0	0.1	0.1	1.7	3.0	**1.8**
F3P16_RS05895	0.0	0.0	0.7	0.0	0.0	0.0	4.5	10.3	**0.3**
F3P16_RS05900	0.0	0.0	UC	0.0	0.0	0.0	UC	UC	**UC**
F3P16_RS05905	0.0	0.0	UC	0.0	0.0	0.1	1.0	2.4	**UC**
F3P16_RS05910	0.7	1.5	2.1	1.9	1.8	2.8	0.9	1.4	**2.7**
mltB	0.6	2.7	4.9	1.1	1.4	1.7	1.3	1.6	**1.9**
rodA	0.2	0.2	1.0	0.1	0.2	0.3	1.3	2.1	**0.7**
F3P16_RS05925	0.1	0.1	1.0	0.1	0.2	0.2	1.5	1.9	**1.0**
queF	0.4	0.6	1.5	0.5	0.8	1.0	1.5	1.8	**1.3**
F3P16_RS05935	0.1	0.2	1.8	0.2	0.3	0.3	1.5	1.4	**2.1**
F3P16_RS05940	0.3	0.4	1.3	0.3	0.5	0.5	1.5	1.7	**1.2**
F3P16_RS05945	0.0	0.0	UC	0.0	0.0	0.0	UC	UC	**UC**
F3P16_RS05950	0.0	0.0	UC	0.0	0.0	0.0	UC	UC	**UC**
F3P16_RS05955	0.0	0.0	UC	0.0	0.0	0.0	UC	UC	**UC**
F3P16_RS18945	0.0	0.0	UC	0.0	0.0	0.0	UC	UC	**UC**
F3P16_RS05965	0.0	0.0	UC	0.0	0.0	0.0	UC	UC	**UC**
F3P16_RS05970	0.0	0.0	UC	0.0	0.0	0.0	UC	UC	**UC**
F3P16_RS05975	0.0	0.0	UC	0.0	0.0	0.0	UC	UC	**UC**
F3P16_RS05980	0.0	0.0	UC	0.0	0.0	0.0	UC	UC	**UC**
F3P16_RS05985	0.0	0.0	UC	0.0	0.0	0.0	UC	UC	**UC**
F3P16_RS19020	0.0	0.0	UC	0.0	0.0	0.0	UC	UC	**UC**
F3P16_RS05995	0.0	0.0	UC	0.0	0.0	0.0	UC	UC	**UC**

F3P16_RS19025	0.0	0.0	UC	0.0	0.0	0.0	UC	UC	**UC**
F3P16_RS06005	0.0	0.0	UC	0.0	0.0	0.0	UC	UC	**UC**
F3P16_RS06010	0.0	0.0	UC	0.0	0.0	0.0	UC	UC	**UC**
F3P16_RS06015	0.0	0.0	UC	0.0	0.0	0.0	UC	UC	**UC**
F3P16_RS06020	0.0	0.0	UC	0.0	0.0	0.0	UC	UC	**UC**
F3P16_RS06025	0.0	0.0	UC	0.0	0.0	0.0	UC	UC	**UC**
F3P16_RS06030	0.0	0.0	UC	0.0	0.0	0.0	UC	UC	**UC**
F3P16_RS06035	0.0	0.0	UC	0.0	0.0	0.0	UC	UC	**UC**
F3P16_RS06040	0.0	0.0	UC	0.0	0.0	0.0	UC	UC	**UC**
F3P16_RS06045	0.0	0.0	UC	0.0	0.0	0.0	UC	UC	**UC**
F3P16_RS06050	0.0	0.0	UC	0.0	0.0	0.0	UC	UC	**UC**
F3P16_RS06055	0.0	0.0	UC	0.0	0.0	0.0	UC	UC	**UC**
F3P16_RS06060	0.0	0.0	UC	0.0	0.0	0.0	UC	UC	**UC**
F3P16_RS06065	0.0	0.0	UC	0.0	0.0	0.0	UC	UC	**UC**
F3P16_RS06075	0.0	0.0	UC	0.0	0.0	0.0	UC	UC	**UC**
F3P16_RS06080	0.0	0.0	UC	0.0	0.0	0.0	UC	UC	**UC**
F3P16_RS06085	0.0	0.0	UC	0.0	0.0	0.0	UC	UC	**UC**
F3P16_RS06090	0.0	0.0	UC	0.0	0.0	0.0	UC	UC	**UC**
F3P16_RS06095	0.0	0.0	UC	0.0	0.0	0.0	UC	UC	**UC**
F3P16_RS06100	0.0	0.0	UC	0.0	0.0	0.0	UC	UC	**UC**
F3P16_RS06105	0.0	0.0	UC	0.0	0.0	0.0	UC	UC	**UC**
F3P16_RS06110	0.0	0.0	UC	0.0	0.0	0.0	UC	UC	**UC**
F3P16_RS06115	0.0	0.0	UC	0.0	0.0	0.0	UC	UC	**UC**
F3P16_RS06120	0.0	0.0	UC	0.0	0.0	0.0	UC	UC	**UC**
F3P16_RS06125	0.0	0.0	UC	0.0	0.0	0.0	UC	UC	**UC**
F3P16_RS06130	0.0	0.0	UC	0.0	0.0	0.0	UC	UC	**UC**
F3P16_RS06135	0.0	0.0	UC	0.0	0.0	0.0	UC	UC	**UC**
F3P16_RS06140	0.0	0.0	UC	0.0	0.0	0.0	UC	UC	**UC**
F3P16_RS06145	0.0	0.0	UC	0.0	0.0	0.0	UC	UC	**UC**
terL	0.0	0.0	UC	0.0	0.0	0.0	UC	UC	**UC**

F3P16_RS06155	0.0	0.0	UC	0.0	0.0	0.0	UC	UC	**UC**
F3P16_RS06160	0.0	0.0	UC	0.0	0.0	0.0	UC	UC	**UC**
F3P16_RS06165	0.0	0.0	UC	0.0	0.0	0.0	UC	UC	**UC**
F3P16_RS06170	0.0	0.0	UC	0.0	0.0	0.0	UC	UC	**UC**
F3P16_RS06175	0.0	0.0	UC	0.0	0.0	0.0	UC	UC	**UC**
F3P16_RS06180	0.0	0.0	UC	0.0	0.0	0.0	UC	UC	**UC**
F3P16_RS06185	0.0	0.0	UC	0.0	0.0	0.0	UC	UC	**UC**
F3P16_RS06190	0.0	0.0	UC	0.0	0.0	0.0	UC	UC	**UC**
F3P16_RS06195	0.0	0.0	0.0	0.0	0.0	0.0	2.8	2.6	**1.0**
F3P16_RS06200	0.1	0.1	1.8	0.1	0.1	0.2	1.8	2.6	**1.0**
F3P16_RS06205	0.0	0.1	2.3	0.0	0.0	0.1	1.4	2.9	**1.1**
F3P16_RS19030	0.0	0.0	UC	0.0	0.0	0.0	UC	UC	**UC**
F3P16_RS06210	3.7	4.0	1.1	5.8	8.5	14.6	1.4	2.5	**1.6**
adeH	0.1	0.1	1.6	0.1	0.2	0.2	2.0	2.6	**1.3**
adeG	0.0	0.1	1.8	0.1	0.1	0.1	1.0	1.6	**1.9**
adeF	0.0	0.0	2.6	0.0	0.1	0.1	2.0	1.6	**2.6**
adeL	0.0	0.0	1.4	0.1	0.1	0.1	0.8	1.4	**3.7**
F3P16_RS06235	0.0	0.0	1.5	0.0	0.0	0.1	1.7	4.1	**0.8**
hisQ	0.1	0.1	0.9	0.0	0.1	0.1	2.8	3.0	**0.2**
F3P16_RS06245	0.1	0.0	0.8	0.0	0.0	0.1	1.3	3.2	**0.5**
F3P16_RS06250	0.0	0.0	0.9	0.0	0.0	0.1	0.5	1.5	**2.6**
abeS	0.0	0.0	UC	0.0	0.0	0.1	0.4	2.3	**UC**
F3P16_RS06260	0.5	0.4	0.9	0.3	0.3	0.5	0.9	1.8	**0.7**
yegQ	0.7	1.0	1.3	0.7	1.0	1.3	1.4	1.8	**0.9**
F3P16_RS06270	0.1	0.2	2.4	0.1	0.2	0.1	1.6	1.1	**2.1**
F3P16_RS06275	0.0	0.0	0.0	0.0	0.0	0.0	UC	UC	**0.0**
F3P16_RS06280	0.4	0.4	1.0	0.5	1.0	1.5	2.0	3.0	**1.2**
tsaA	0.1	0.1	1.7	0.1	0.1	0.2	0.8	1.8	**2.2**
F3P16_RS06290	0.1	0.1	2.1	0.1	0.1	0.1	1.0	1.3	**1.4**
tadA	0.0	0.0	1.5	0.0	0.0	0.1	1.2	2.8	**1.0**

F3P16_RS06300	0.0	0.0	1.4	0.0	0.2	0.1	8.5	2.9	**1.3**
F3P16_RS06305	0.0	0.1	1.7	0.2	0.2	0.1	1.3	0.7	**3.8**
F3P16_RS06310	0.1	0.1	1.1	0.1	0.1	0.2	1.8	1.9	**0.8**
F3P16_RS06315	0.1	0.1	0.9	0.1	0.0	0.1	0.0	1.9	**1.3**
F3P16_RS06320	0.1	0.1	1.9	0.2	0.3	0.3	1.8	1.7	**3.0**
F3P16_RS06325	1.3	5.6	4.2	5.4	7.3	5.9	1.3	1.1	**4.1**
mrcB	1.3	5.2	4.0	5.2	6.9	4.6	1.3	0.9	**4.0**
F3P16_RS06335	0.8	1.3	1.5	1.5	1.8	2.7	1.2	1.8	**1.8**
F3P16_RS06340	0.1	0.3	2.6	0.3	0.5	0.9	1.4	2.7	**3.0**
F3P16_RS06345	0.1	0.3	2.8	0.3	0.3	0.7	1.1	2.7	**2.2**
F3P16_RS06355	1.0	1.5	1.6	1.8	2.8	3.8	1.5	2.1	**1.9**
F3P16_RS06360	0.1	0.2	2.0	0.2	0.3	0.2	1.2	1.0	**2.3**
F3P16_RS06365	0.0	0.0	0.8	0.0	0.0	0.0	UC	UC	**0.0**
pheA	0.8	1.6	2.0	1.3	2.1	1.9	1.7	1.5	**1.5**
F3P16_RS06375	1.2	2.1	1.7	2.2	2.6	2.8	1.2	1.3	**1.8**
F3P16_RS06380	0.0	0.1	3.4	0.1	0.2	0.1	1.5	1.0	**3.4**
F3P16_RS06385	0.2	0.6	3.3	0.5	0.4	0.9	0.9	2.0	**2.3**
F3P16_RS06390	0.1	0.1	1.4	0.0	0.1	0.1	2.8	3.0	**0.6**
F3P16_RS06395	0.1	0.2	1.5	0.1	0.2	0.3	1.3	1.9	**1.3**
yaaA	0.1	0.2	1.5	0.1	0.3	0.2	1.9	1.6	**1.2**
F3P16_RS06405	0.1	0.1	1.2	0.1	0.2	0.1	3.0	1.9	**1.0**
F3P16_RS06410	0.6	2.0	3.1	1.6	2.4	2.0	1.5	1.3	**2.5**
thiS	3.0	7.2	2.4	7.1	9.2	8.0	1.3	1.1	**2.3**
F3P16_RS06420	1.2	5.4	4.3	3.5	4.7	3.7	1.3	1.1	**2.8**
rpoH	3.5	15.5	4.4	14.1	15.4	17.9	1.1	1.3	**4.0**
F3P16_RS06430	0.1	0.1	1.5	0.1	0.3	0.2	1.8	1.2	**1.8**
F3P16_RS19035	0.1	0.0	0.0	0.2	0.1	0.0	0.8	0.2	**2.0**
F3P16_RS06435	1.7	2.0	1.2	3.1	5.7	4.9	1.8	1.6	**1.8**
rhlB	5.6	10.8	1.9	12.7	21.4	26.3	1.7	2.1	**2.3**
F3P16_RS06445	0.1	1.6	30.5	2.5	3.7	1.4	1.5	0.6	**47.0**

F3P16_RS06450	0.6	2.3	3.8	1.3	2.0	1.7	1.5	1.3	**2.2**
F3P16_RS06455	0.6	3.2	5.6	1.8	2.3	1.4	1.3	0.8	**3.2**
F3P16_RS06460	0.2	1.2	5.8	0.9	1.1	0.9	1.2	1.0	**4.4**
gspL	0.5	1.1	2.2	0.7	1.5	1.1	2.0	1.4	**1.5**
F3P16_RS06470	0.1	0.2	1.6	0.2	0.5	0.3	2.2	1.2	**1.6**
F3P16_RS06475	0.4	0.8	2.0	0.7	1.3	1.2	1.8	1.7	**1.8**
F3P16_RS06480	0.6	0.8	1.3	0.9	1.5	1.5	1.5	1.6	**1.6**
purF	0.8	1.6	2.0	1.8	2.3	3.1	1.3	1.7	**2.2**
F3P16_RS06490	0.0	0.1	1.1	0.1	0.1	0.2	1.8	2.7	**1.3**
F3P16_RS06495	0.0	0.1	2.1	0.1	0.1	0.2	1.1	1.4	**2.8**
F3P16_RS06500	0.1	0.2	1.4	0.2	0.4	0.3	1.6	1.2	**2.0**
F3P16_RS06505	0.1	1.1	17.3	0.7	0.8	0.7	1.3	1.0	**10.4**
F3P16_RS06510	1.4	1.7	1.3	2.5	3.2	4.5	1.3	1.8	**1.8**
rpsU	2.4	2.3	1.0	3.5	4.1	7.3	1.2	2.1	**1.5**
tsaD	0.3	0.3	1.1	0.5	0.5	0.9	1.0	1.6	**1.9**
F3P16_RS06525	0.0	0.0	1.2	0.0	0.1	0.1	3.2	5.1	**1.1**
F3P16_RS06530	0.1	0.2	1.7	0.1	0.2	0.3	1.6	2.2	**1.2**
F3P16_RS06535	0.3	0.6	2.1	0.4	0.4	0.5	1.1	1.3	**1.5**
F3P16_RS06540	0.0	0.1	4.2	0.1	0.1	0.1	1.0	1.3	**3.2**
F3P16_RS06545	0.2	0.2	0.9	0.2	0.4	0.8	1.8	3.4	**1.1**
F3P16_RS06550	0.2	0.1	0.7	0.4	0.1	1.0	0.2	2.9	**2.0**
F3P16_RS06555	0.2	0.2	0.9	0.2	0.4	0.8	1.8	3.4	**1.1**
F3P16_RS06560	0.2	0.1	0.7	0.4	0.1	1.0	0.2	2.9	**2.0**
F3P16_RS06565	0.0	0.0	UC	0.0	0.0	0.0	UC	UC	**UC**
F3P16_RS06570	0.0	0.0	UC	0.0	0.0	0.0	UC	UC	**UC**
F3P16_RS06575	0.0	0.0	UC	0.0	0.0	0.0	UC	UC	**UC**
F3P16_RS18860	0.0	0.0	UC	0.0	0.0	0.0	UC	UC	**UC**
F3P16_RS06590	0.0	0.0	UC	0.0	0.0	0.0	UC	UC	**UC**
F3P16_RS06595	0.0	0.0	UC	0.0	0.0	0.0	UC	UC	**UC**
F3P16_RS19040	0.0	0.0	UC	0.0	0.0	0.0	UC	UC	**UC**

F3P16_RS06605	0.0	0.0	UC	0.0	0.0	0.0	UC	UC	**UC**
F3P16_RS06610	0.0	0.0	UC	0.0	0.0	0.0	UC	UC	**UC**
F3P16_RS06615	0.0	0.0	UC	0.0	0.0	0.0	UC	UC	**UC**
F3P16_RS06620	0.0	0.0	UC	0.0	0.0	0.0	UC	UC	**UC**
F3P16_RS06625	0.0	0.0	UC	0.0	0.0	0.0	UC	UC	**UC**
F3P16_RS06630	0.0	0.0	UC	0.0	0.0	0.0	UC	UC	**UC**
F3P16_RS06635	0.0	0.0	UC	0.0	0.0	0.0	UC	UC	**UC**
F3P16_RS06640	0.0	0.0	UC	0.0	0.0	0.0	UC	UC	**UC**
F3P16_RS06645	0.0	0.0	0.8	0.0	0.0	0.1	0.6	3.0	**0.8**
F3P16_RS06650	0.1	0.0	0.7	0.0	0.2	0.1	5.7	3.7	**0.4**
F3P16_RS06655	0.1	0.2	1.2	0.1	0.3	0.2	2.5	1.5	**0.7**
F3P16_RS06660	0.0	0.0	1.1	0.0	0.0	0.0	0.9	1.0	**1.6**
F3P16_RS06665	0.1	0.3	3.6	0.0	0.0	0.1	0.8	2.7	**0.5**
F3P16_RS06670	0.0	0.3	8.4	0.0	0.0	0.1	1.2	3.4	**1.0**
F3P16_RS06675	0.1	0.4	7.7	0.1	0.0	0.1	0.6	1.9	**1.3**
F3P16_RS06680	0.0	0.3	8.2	0.1	0.1	0.1	0.5	1.4	**2.8**
F3P16_RS06685	0.0	0.1	UC	0.0	0.0	0.1	2.0	26.8	**UC**
F3P16_RS06690	0.0	0.0	3.7	0.0	0.0	0.0	2.0	2.3	**0.5**
F3P16_RS06695	0.0	0.0	UC	0.0	0.0	0.0	2.0	4.6	**UC**
F3P16_RS06700	0.0	0.0	1.4	0.0	0.0	0.1	UC	UC	**0.0**
F3P16_RS06705	0.0	0.0	UC	0.0	0.0	0.0	UC	UC	**UC**
F3P16_RS06710	0.0	0.1	7.2	0.0	0.1	0.1	1.2	2.4	**4.4**
F3P16_RS06715	0.0	0.0	1.5	0.0	0.0	0.1	1.8	3.8	**0.8**
gltS	0.4	0.7	1.9	0.5	0.8	2.5	1.6	4.7	**1.5**
F3P16_RS06725	0.0	0.1	1.6	0.1	0.1	0.2	1.2	3.0	**2.5**
F3P16_RS06730	0.0	0.1	1.5	0.1	0.1	0.3	1.1	2.9	**3.2**
F3P16_RS06735	0.1	0.0	0.5	0.2	0.1	0.3	0.7	1.3	**3.0**
F3P16_RS06740	0.0	0.0	UC	0.0	0.0	0.0	UC	UC	**UC**
csuA	2.1	1.9	0.9	1.5	2.5	3.6	1.7	2.4	**0.7**
csuA	0.1	0.1	1.3	0.1	0.1	0.1	1.2	1.1	**1.4**

csuB	0.1	0.0	0.6	0.1	0.1	0.1	2.4	1.2	**1.1**
csuC	0.4	0.3	0.8	0.2	0.4	0.3	2.1	1.8	**0.5**
csuD	0.6	0.6	1.0	0.4	0.9	0.7	2.5	2.0	**0.6**
csuE	0.3	0.5	1.5	0.3	0.8	0.7	2.3	2.0	**1.1**
F3P16_RS18950	0.0	0.0	0.0	0.0	0.0	0.0	2.0	2.3	**0.5**
proP	0.2	0.1	0.5	1.0	1.3	2.2	1.3	2.2	**5.7**
F3P16_RS06785	0.0	0.0	UC	0.0	0.0	0.0	UC	UC	**UC**
F3P16_RS06790	0.0	0.0	UC	0.0	0.0	0.0	UC	UC	**UC**
F3P16_RS06795	0.0	0.1	3.7	0.0	0.0	0.0	5.0	8.6	**0.2**
F3P16_RS06800	1.5	4.8	3.2	0.8	1.1	0.3	1.4	0.3	**0.5**
aspT	0.7	2.4	3.4	0.3	0.4	0.2	1.7	0.6	**0.4**
F3P16_RS06810	0.0	0.0	UC	0.0	0.0	0.0	UC	UC	**UC**
F3P16_RS06815	0.0	0.0	1.9	0.1	0.0	0.0	0.3	0.8	**4.4**
F3P16_RS06820	0.0	0.1	3.1	0.0	0.0	0.0	0.7	1.0	**2.2**
F3P16_RS06825	0.0	0.1	2.4	0.0	0.1	0.1	1.9	1.2	**1.0**
F3P16_RS06830	0.0	0.1	1.4	0.0	0.1	0.0	2.5	1.3	**0.7**
uraH	0.0	0.0	UC	0.0	0.0	0.0	UC	UC	**UC**
F3P16_RS06840	0.0	0.0	UC	0.0	0.0	0.0	UC	UC	**UC**
F3P16_RS06845	0.0	0.0	UC	0.0	0.0	0.0	UC	UC	**UC**
F3P16_RS06850	0.0	0.0	UC	0.0	0.0	0.0	UC	UC	**UC**
F3P16_RS06855	0.0	0.0	UC	0.0	0.0	0.0	UC	UC	**UC**
F3P16_RS06860	0.0	0.0	UC	0.0	0.0	0.0	UC	UC	**UC**
F3P16_RS06865	0.0	0.0	UC	0.0	0.0	0.0	UC	UC	**UC**
F3P16_RS06870	0.0	0.0	UC	0.0	0.0	0.0	UC	UC	**UC**
F3P16_RS06875	0.0	0.0	UC	0.0	0.0	0.0	UC	UC	**UC**
F3P16_RS06880	0.0	0.0	UC	0.0	0.0	0.0	UC	UC	**UC**
F3P16_RS06885	0.0	0.0	UC	0.0	0.0	0.0	UC	UC	**UC**
F3P16_RS06890	0.0	0.0	UC	0.0	0.0	0.0	UC	UC	**UC**
F3P16_RS06895	0.0	0.0	UC	0.0	0.0	0.0	UC	UC	**UC**
F3P16_RS06900	0.0	0.0	UC	0.0	0.0	0.0	UC	UC	**UC**

F3P16_RS06905	0.0	0.0	UC	0.0	0.0	0.0	UC	UC	**UC**
F3P16_RS06910	0.0	0.0	UC	0.0	0.0	0.0	UC	UC	**UC**
F3P16_RS06915	0.1	0.1	2.2	0.1	0.1	0.0	0.9	0.6	**1.4**
yddG	0.0	0.1	1.4	0.0	0.1	0.1	1.6	2.5	**0.9**
F3P16_RS06925	0.0	0.0	UC	0.0	0.0	0.0	UC	UC	**UC**
F3P16_RS06930	0.0	0.0	UC	0.0	0.0	0.0	UC	UC	**UC**
F3P16_RS06935	0.0	0.0	UC	0.0	0.0	0.0	UC	UC	**UC**
F3P16_RS06940	0.0	0.0	UC	0.0	0.0	0.0	UC	UC	**UC**
F3P16_RS06945	0.0	0.2	6.1	0.1	0.1	1.1	0.7	10.4	**2.7**
F3P16_RS06950	0.0	0.2	UC	0.1	0.1	0.6	1.0	7.9	**UC**
gloB	0.0	0.1	2.6	0.1	0.1	0.2	1.2	1.9	**2.7**
F3P16_RS06960	0.3	0.4	1.6	0.5	0.8	0.9	1.6	1.7	**2.1**
F3P16_RS06965	2.0	3.0	1.5	3.4	5.6	5.8	1.6	1.7	**1.7**
F3P16_RS06970	0.7	1.0	1.4	0.9	1.4	1.5	1.5	1.7	**1.3**
purD	0.9	1.7	2.0	2.1	4.0	3.8	1.9	1.8	**2.4**
purH	3.5	6.4	1.8	6.8	12.4	10.4	1.8	1.5	**1.9**
fis]	1.0	1.6	1.6	2.4	6.1	4.4	2.5	1.8	**2.4**
F3P16_RS06990	0.1	0.4	2.6	0.3	0.6	0.5	2.0	1.7	**2.1**
prmA	0.7	0.9	1.5	0.9	1.4	1.8	1.6	2.0	**1.4**
F3P16_RS07000	1.0	3.4	3.3	1.8	1.7	3.3	0.9	1.8	**1.8**
mnmG	5.3	9.1	1.7	10.0	14.3	9.5	1.4	1.0	**1.9**
F3P16_RS18955	0.1	0.2	3.3	0.1	0.2	0.4	1.3	2.9	**2.4**
F3P16_RS07015	0.1	0.2	1.1	0.2	0.2	0.1	1.6	0.9	**1.0**
F3P16_RS07020	0.2	0.2	1.3	0.2	0.3	0.2	1.8	1.0	**0.9**
F3P16_RS07025	0.2	0.2	1.0	0.2	0.3	0.3	1.3	1.2	**1.0**
F3P16_RS07030	0.1	0.2	1.6	0.2	0.3	0.4	1.5	1.8	**1.9**
alr]	0.2	0.2	1.2	0.3	0.5	0.6	1.4	1.8	**2.3**
dnaB	0.7	0.8	1.2	0.7	1.3	1.0	1.9	1.5	**1.0**
rplI	55.3	87.6	1.6	123.7	206.0	404.1	1.7	3.3	**2.2**
rpsR	126.7	204.6	1.6	311.2	525.2	1016.6	1.7	3.3	**2.5**

rpsF	37.7	58.5	1.6	75.3	135.2	238.8	1.8	3.2	**2.0**
cyoE	3.7	8.7	2.3	11.0	18.7	26.0	1.7	2.3	**3.0**
F3P16_RS07065	7.6	15.0	2.0	17.2	29.2	39.9	1.7	2.3	**2.3**
cyoC	8.7	16.0	1.8	18.3	31.2	52.7	1.7	2.9	**2.1**
cyoB	14.6	20.9	1.4	23.1	36.5	59.2	1.6	2.6	**1.6**
cyoA	13.1	19.7	1.5	18.7	31.9	43.8	1.7	2.3	**1.4**
F3P16_RS07085	0.2	0.3	1.8	0.4	0.6	0.6	1.4	1.5	**2.5**
ppsA	7.5	16.8	2.2	15.0	19.5	18.1	1.3	1.2	**2.0**
F3P16_RS07095	0.2	0.3	1.5	0.2	0.3	0.3	1.1	1.2	**1.5**
pgaA	0.0	0.3	24.6	0.1	0.1	0.2	1.8	2.6	**6.2**
pgaB	0.0	0.2	8.6	0.1	0.2	0.2	1.9	2.4	**3.3**
pgaC	0.0	0.2	6.8	0.1	0.1	0.2	1.0	1.4	**4.3**
pgaD	0.1	0.1	1.5	0.1	0.1	0.2	1.2	2.0	**1.7**
F3P16_RS07120	0.0	0.1	UC	0.0	0.1	0.2	2.0	6.8	**UC**
F3P16_RS07125	0.0	0.8	40.5	0.3	0.4	0.8	1.3	2.6	**16.3**
F3P16_RS07130	0.0	3.2	97.2	1.6	1.5	2.9	0.9	1.9	**46.9**
F3P16_RS07140	0.0	1.9	40.3	1.4	1.3	1.5	0.9	1.0	**29.7**
F3P16_RS07145	0.0	0.0	1.4	0.0	0.0	0.1	0.3	3.2	**1.6**
F3P16_RS07150	0.1	0.1	1.5	0.0	0.0	0.2	1.0	3.9	**0.8**
prfA	0.6	0.6	0.9	0.6	0.8	1.2	1.3	2.0	**1.0**
prmC	0.2	0.3	1.3	0.3	0.4	0.8	1.3	2.3	**1.6**
F3P16_RS07165	0.0	0.0	UC	0.0	0.0	0.1	5.0	13.7	**UC**
F3P16_RS07170	0.0	0.1	3.0	0.0	0.0	0.1	1.1	5.0	**1.1**
F3P16_RS07175	0.0	0.0	4.2	0.0	0.0	0.0	0.5	1.3	**12.9**
F3P16_RS07180	0.0	0.0	UC	0.0	0.0	0.0	1.8	2.9	**UC**
F3P16_RS07185	0.0	0.1	2.0	0.0	0.0	0.1	0.4	1.4	**1.9**
F3P16_RS07190	0.0	0.1	6.5	0.0	0.0	0.1	0.5	1.8	**4.3**
F3P16_RS07195	0.0	0.0	2.3	0.1	0.1	0.1	1.2	1.6	**3.1**
F3P16_RS07200	0.3	1.1	3.3	1.4	1.7	2.3	1.2	1.7	**4.0**
F3P16_RS07205	0.6	1.9	3.4	2.1	2.7	2.8	1.3	1.3	**3.8**

folB	1.8	3.3	1.9	3.3	3.7	4.2	1.1	1.3	**1.9**
F3P16_RS07215	0.1	0.1	2.3	0.2	0.2	0.2	1.3	1.1	**2.8**
kdpA	0.1	0.1	0.6	0.0	0.1	0.2	1.5	5.0	**0.4**
kdpB	0.1	0.1	0.8	0.1	0.1	0.1	1.3	2.1	**0.9**
kdpC	0.0	0.0	0.8	0.0	0.0	0.1	1.0	2.5	**0.7**
F3P16_RS07235	0.0	0.0	1.0	0.0	0.1	0.1	2.6	2.9	**0.7**
F3P16_RS07240	0.0	0.1	2.7	0.0	0.1	0.0	4.4	2.9	**0.8**
F3P16_RS07245	0.0	0.0	UC	0.0	0.0	0.0	1.3	5.3	**UC**
F3P16_RS07250	0.0	0.1	UC	0.0	0.0	0.1	5.0	12.5	**UC**
coq7	0.1	0.2	3.5	0.2	0.3	0.5	2.0	2.8	**3.0**
F3P16_RS07260	0.1	0.2	2.2	0.2	0.3	0.2	1.5	1.4	**1.6**
F3P16_RS07265	0.4	0.8	1.8	0.7	0.9	1.0	1.3	1.4	**1.5**
F3P16_RS07270	0.9	1.5	1.8	1.5	2.6	1.6	1.7	1.1	**1.7**
F3P16_RS07275	0.1	0.1	1.3	0.1	0.1	0.2	1.3	1.8	**1.8**
F3P16_RS07285	5.3	8.2	1.6	7.8	15.9	19.6	2.0	2.5	**1.5**
F3P16_RS07290	0.0	0.0	UC	0.0	0.0	0.0	UC	UC	**UC**
F3P16_RS07295	0.1	0.1	1.8	0.1	0.1	0.1	1.1	2.4	**1.0**
F3P16_RS07300	0.8	1.2	1.4	1.0	1.6	2.4	1.7	2.4	**1.2**
F3P16_RS07305	0.0	0.0	UC	0.0	0.0	0.0	UC	UC	**UC**
F3P16_RS07310	0.0	0.0	0.0	0.0	0.0	0.0	UC	UC	**0.0**
F3P16_RS07315	0.2	0.2	1.2	0.3	0.4	0.4	1.4	1.2	**1.8**
F3P16_RS07320	0.0	0.0	5.3	0.1	0.1	0.1	0.5	0.4	**15.9**
F3P16_RS07325	0.0	0.0	2.1	0.0	0.0	0.0	UC	UC	**0.0**
F3P16_RS07330	0.1	0.2	2.3	0.2	0.3	0.3	1.6	2.0	**1.9**
F3P16_RS07335	0.0	0.1	3.0	0.1	0.2	0.5	1.8	3.4	**3.0**
F3P16_RS07340	0.0	0.0	UC	0.0	0.0	0.2	1.0	3.7	**UC**
F3P16_RS07345	0.0	0.0	2.1	0.0	0.0	0.1	1.1	1.4	**6.0**
tsaE	0.1	0.2	1.5	0.1	0.3	0.4	2.2	3.6	**0.8**
mutL	0.3	0.7	2.1	0.6	1.0	1.0	1.5	1.6	**1.9**
miaA	0.2	0.5	2.0	0.9	1.3	1.1	1.4	1.2	**3.9**

hfq]	3.6	6.7	1.9	9.2	12.7	10.5	1.4	1.1	**2.6**
F3P16_RS07370	0.0	0.1	20.1	0.1	0.2	0.2	2.3	2.0	**14.9**
nfsB	0.0	0.1	5.5	0.1	0.1	0.1	1.4	2.4	**5.7**
F3P16_RS07380	0.0	0.0	UC	0.0	0.0	0.0	UC	UC	**UC**
F3P16_RS07385	0.1	0.1	2.4	0.3	0.3	0.3	1.1	1.0	**5.9**
F3P16_RS07390	0.2	0.5	2.4	0.9	0.9	2.0	1.1	2.3	**4.4**
F3P16_RS07395	1.4	1.3	1.0	2.0	3.3	3.7	1.6	1.8	**1.5**
F3P16_RS07400	0.0	0.2	4.0	0.1	0.2	0.3	1.6	2.3	**2.6**
eutC	0.0	0.1	2.7	0.1	0.1	0.1	1.1	1.7	**3.9**
F3P16_RS07410	0.0	0.1	4.8	0.1	0.0	0.1	0.5	0.9	**5.0**
eat]	0.0	0.1	2.7	0.0	0.0	0.1	1.0	2.8	**2.0**
F3P16_RS07420	1.2	3.5	2.9	3.3	2.6	2.7	0.8	0.8	**2.8**
F3P16_RS07425	0.1	0.6	4.5	0.6	0.5	1.4	0.9	2.3	**4.8**
F3P16_RS07430	0.2	0.4	2.0	0.2	0.3	0.5	1.2	2.2	**1.1**
F3P16_RS07435	1.0	2.2	2.2	1.3	0.8	1.2	0.6	0.9	**1.3**
F3P16_RS07440	0.4	0.4	1.2	0.4	0.6	0.7	1.3	1.7	**1.2**
F3P16_RS07445	0.0	0.1	8.4	0.1	0.2	0.2	3.4	3.2	**6.0**
thiM	0.1	0.2	2.3	0.2	0.3	0.2	1.5	1.4	**1.9**
F3P16_RS07455	0.1	0.2	1.3	0.2	0.3	0.2	1.2	0.9	**1.7**
F3P16_RS07460	0.1	0.1	2.2	0.1	0.1	0.3	0.7	1.8	**2.2**
pepN	3.1	10.0	3.2	6.4	7.2	12.7	1.1	2.0	**2.0**
F3P16_RS07470	0.0	0.0	UC	0.0	0.0	0.0	UC	UC	**UC**
F3P16_RS07475	0.0	0.0	UC	0.0	0.0	0.0	UC	UC	**UC**
F3P16_RS07480	0.0	0.0	UC	0.0	0.0	0.0	UC	UC	**UC**
F3P16_RS07485	0.0	0.0	UC	0.0	0.0	0.0	UC	UC	**UC**
F3P16_RS07490	0.0	0.1	5.1	0.1	0.0	0.1	0.7	1.0	**5.0**
F3P16_RS07495	0.0	0.1	2.3	0.1	0.1	0.1	1.3	1.0	**1.7**
F3P16_RS07500	0.0	0.0	UC	0.0	0.0	0.0	4.0	5.7	**UC**
F3P16_RS07505	0.1	0.0	0.5	0.0	0.0	0.1	1.6	2.5	**0.6**
F3P16_RS07510	0.1	0.1	1.9	0.1	0.1	0.2	1.4	3.3	**1.2**

F3P16_RS07520	0.0	0.0	UC	0.0	0.0	0.0	UC	UC	**UC**
F3P16_RS07525	0.0	0.0	UC	0.0	0.0	0.0	UC	UC	**UC**
F3P16_RS07530	0.0	0.0	UC	0.0	0.0	0.0	UC	UC	**UC**
F3P16_RS07535	0.0	0.1	1.4	0.0	0.1	0.1	1.9	3.6	**0.8**
F3P16_RS07540	0.1	0.3	3.3	0.3	0.3	0.6	1.2	2.5	**3.3**
F3P16_RS07545	0.0	0.1	6.3	0.1	0.1	0.8	0.8	5.4	**10.9**
F3P16_RS07550	0.0	0.0	UC	0.0	0.0	0.1	UC	UC	**UC**
F3P16_RS07555	0.0	0.1	UC	0.1	0.0	0.1	0.4	0.9	**UC**
F3P16_RS07560	0.0	0.0	UC	0.1	0.1	0.1	0.9	1.1	**UC**
F3P16_RS07565	0.0	0.0	1.1	0.0	0.1	0.1	1.7	4.0	**2.5**
F3P16_RS07570	0.0	0.0	1.1	0.0	0.0	0.0	13.5	20.5	**0.1**
F3P16_RS07575	0.0	0.0	3.4	0.0	0.0	0.0	1.1	1.4	**3.2**
F3P16_RS07580	0.0	0.0	UC	0.1	0.0	0.3	0.0	5.4	**UC**
F3P16_RS07590	0.0	0.0	UC	0.0	0.0	0.0	UC	UC	**UC**
F3P16_RS07595	0.1	0.1	1.5	0.1	0.2	0.1	1.6	0.8	**1.7**
F3P16_RS07600	0.4	0.3	0.7	0.2	0.2	0.2	1.6	1.4	**0.3**
F3P16_RS07605	0.0	0.0	UC	0.0	0.0	0.0	UC	UC	**UC**
F3P16_RS18875	0.0	0.0	UC	0.0	0.0	0.0	UC	UC	**UC**
F3P16_RS07610	0.0	0.0	UC	0.0	0.0	0.0	UC	UC	**UC**
F3P16_RS18880	0.0	0.0	UC	0.0	0.0	0.0	0.0	0.6	**UC**
mgtA	0.0	0.0	2.1	0.0	0.0	0.1	1.5	3.0	**1.8**
F3P16_RS07620	0.0	0.1	8.2	0.0	0.0	0.1	1.4	4.9	**2.6**
F3P16_RS07625	0.0	0.0	0.9	0.0	0.0	0.1	1.3	4.8	**0.5**
F3P16_RS07630	0.0	0.0	2.1	0.0	0.0	0.1	3.5	6.6	**2.0**
leuE	0.0	0.1	8.2	0.1	0.2	0.1	1.5	0.9	**11.7**
tal]	1.2	3.7	3.1	4.5	3.8	7.7	0.8	1.7	**3.7**
F3P16_RS07645	0.1	0.1	2.8	0.1	0.1	0.2	1.5	2.0	**1.8**
aceI	0.0	0.0	UC	0.0	0.0	0.0	2.3	3.1	**UC**
F3P16_RS07655	0.4	0.9	2.2	0.8	0.9	1.0	1.1	1.2	**2.1**
F3P16_RS07660	0.2	1.2	5.1	1.1	1.2	1.4	1.1	1.3	**4.6**

F3P16_RS07665	0.6	2.0	3.6	1.7	1.9	1.1	1.1	0.7	**2.9**
F3P16_RS07670	0.2	0.8	3.3	0.9	0.8	0.5	0.9	0.6	**3.6**
F3P16_RS07675	0.0	0.1	2.4	0.1	0.1	0.1	1.7	2.1	**1.8**
amvA	0.8	1.9	2.4	1.1	1.8	1.0	1.6	0.9	**1.4**
F3P16_RS07685	0.6	0.8	1.3	1.0	1.3	1.5	1.2	1.4	**1.8**
F3P16_RS07690	0.1	0.1	0.9	0.0	0.1	0.1	1.9	2.3	**0.9**
F3P16_RS07695	0.1	0.0	0.8	0.1	0.2	0.1	1.4	1.4	**1.7**
F3P16_RS07700	0.2	0.2	1.2	0.1	0.2	0.2	1.1	1.5	**0.8**
F3P16_RS07705	0.1	0.3	3.7	0.4	0.5	0.8	1.2	1.8	**5.0**
F3P16_RS07710	0.1	0.2	2.2	0.2	0.4	0.4	1.5	1.6	**2.2**
murB	0.2	0.5	2.2	0.3	0.7	0.7	2.1	1.9	**1.7**
F3P16_RS07720	0.2	0.3	1.8	0.3	0.4	0.4	1.5	1.6	**1.6**
F3P16_RS07725	0.2	0.3	1.6	0.2	0.4	0.4	1.5	1.8	**1.3**
F3P16_RS07730	0.0	0.0	UC	0.0	0.0	0.0	UC	UC	**UC**
F3P16_RS07735	0.0	0.0	UC	0.0	0.0	0.0	UC	UC	**UC**
F3P16_RS07740	0.2	0.9	5.2	0.6	0.8	1.0	1.3	1.6	**3.7**
F3P16_RS07745	0.0	0.0	2.2	0.0	0.0	0.1	0.4	2.0	**2.3**
F3P16_RS07750	0.0	0.0	0.8	0.1	0.1	0.1	1.1	1.9	**1.2**
F3P16_RS07755	0.2	0.4	1.5	0.4	0.4	0.5	1.2	1.6	**1.5**
F3P16_RS07760	0.1	0.0	0.7	0.0	0.1	0.1	3.4	2.7	**0.4**
F3P16_RS07765	0.1	0.3	2.9	0.5	0.8	1.0	1.7	2.1	**4.2**
accC	9.1	19.8	2.2	18.2	34.5	47.8	1.9	2.6	**2.0**
accB	7.0	9.4	1.3	7.5	14.2	19.8	1.9	2.7	**1.1**
aroQ	0.7	1.2	1.8	0.7	1.0	1.6	1.4	2.2	**1.1**
F3P16_RS07785	0.0	0.0	3.4	0.0	0.0	0.1	1.2	2.2	**1.7**
F3P16_RS07790	0.0	0.0	UC	0.0	0.0	0.0	UC	UC	**UC**
F3P16_RS07795	0.0	0.0	1.1	0.0	0.0	0.0	6.0	9.1	**1.0**
F3P16_RS07800	0.0	0.0	0.0	0.0	0.0	0.0	UC	UC	**0.0**
F3P16_RS07805	0.0	0.0	UC	0.0	0.0	0.0	UC	UC	**UC**
nirB	0.0	0.1	1.6	0.0	0.0	0.1	1.3	4.3	**0.6**

nirD	0.0	0.0	1.5	0.1	0.0	0.0	0.8	0.8	**1.6**
F3P16_RS07820	0.0	0.1	2.6	0.0	0.1	0.1	1.6	2.3	**2.0**
F3P16_RS07825	0.0	0.0	UC	0.0	0.0	0.0	0.8	2.3	**UC**
F3P16_RS07830	0.0	0.0	2.0	0.0	0.0	0.1	1.4	5.4	**1.2**
moaA	0.1	0.1	2.7	0.2	0.3	0.3	1.6	1.7	**3.7**
F3P16_RS07840	0.0	0.4	9.0	0.6	0.7	0.4	1.1	0.7	**15.2**
F3P16_RS07845	0.5	1.3	2.8	1.2	1.4	1.4	1.2	1.2	**2.6**
moaC	0.4	1.6	3.7	1.6	1.6	1.3	1.0	0.8	**3.7**
F3P16_RS07855	0.2	0.5	3.3	0.5	0.5	0.3	1.1	0.7	**3.1**
F3P16_RS07860	0.7	0.8	1.1	1.1	1.1	2.1	1.0	2.0	**1.5**
F3P16_RS07865	0.1	0.2	2.0	0.1	0.2	0.4	1.4	4.1	**1.4**
F3P16_RS07870	0.0	0.0	1.1	0.0	0.0	0.1	0.6	1.8	**1.6**
F3P16_RS07875	0.1	0.1	1.2	0.1	0.2	0.3	1.6	1.9	**1.3**
F3P16_RS07880	0.0	0.1	9.5	0.2	0.2	0.3	1.4	2.0	**14.4**
F3P16_RS07885	0.2	0.3	1.4	0.3	0.4	0.8	1.4	3.0	**1.2**
tusD	1.1	4.0	3.5	3.8	5.0	5.7	1.3	1.5	**3.4**
galE	1.4	9.7	7.0	9.4	10.6	8.4	1.1	0.9	**6.8**
fumC	0.9	4.0	4.5	3.5	4.0	3.5	1.1	1.0	**3.9**
F3P16_RS07905	0.0	0.0	UC	0.0	0.0	0.2	0.5	7.4	**UC**
F3P16_RS07910	0.0	0.0	1.1	0.0	0.0	0.0	UC	UC	**0.0**
F3P16_RS07920	0.0	0.0	UC	0.0	0.1	0.1	7.3	4.8	**UC**
F3P16_RS07925	0.9	1.3	1.4	0.8	0.9	0.5	1.1	0.6	**1.0**
F3P16_RS07930	0.0	0.1	14.3	0.1	0.0	0.1	0.5	1.0	**11.9**
ispF	0.0	0.1	4.2	0.0	0.1	0.1	2.4	2.4	**2.6**
F3P16_RS07940	0.0	0.0	2.1	0.1	0.0	0.1	0.2	1.5	**8.9**
argF	0.7	1.1	1.6	1.1	1.8	2.0	1.6	1.8	**1.7**
adeN	0.2	0.5	2.2	0.3	0.7	0.4	2.4	1.4	**1.5**
glnG	2.9	5.8	2.0	3.5	5.8	3.6	1.7	1.0	**1.2**
F3P16_RS07960	0.8	1.5	1.9	0.9	1.3	0.8	1.4	0.8	**1.2**
F3P16_RS07965	0.1	0.0	0.0	0.2	0.0	0.1	0.0	0.7	**1.7**

rimO	4.6	8.3	1.8	7.7	11.3	10.0	1.5	1.3	**1.7**
pyrH	1.7	3.6	2.1	4.2	7.3	6.0	1.7	1.4	**2.4**
frr]	1.6	2.5	1.6	2.9	5.5	5.4	1.9	1.9	**1.8**
uppS	3.5	4.9	1.4	4.5	8.9	8.8	2.0	2.0	**1.3**
F3P16_RS07990	2.8	3.9	1.4	3.5	6.8	6.2	1.9	1.8	**1.3**
ispC	2.9	4.1	1.4	4.1	8.1	7.4	2.0	1.8	**1.4**
rseP	1.6	2.6	1.7	4.0	6.2	6.3	1.5	1.6	**2.6**
bamA	2.1	6.7	3.1	8.5	12.1	10.1	1.4	1.2	**4.0**
F3P16_RS08010	0.6	1.9	3.1	2.7	5.3	3.6	2.0	1.4	**4.4**
lpxD	1.7	4.1	2.4	4.1	8.5	5.6	2.1	1.4	**2.5**
fabZ	5.9	13.6	2.3	9.5	21.3	16.7	2.2	1.8	**1.6**
lpxA	10.1	20.2	2.0	14.1	26.4	24.6	1.9	1.7	**1.4**
F3P16_RS08030	0.4	0.8	2.1	0.9	1.3	1.2	1.4	1.3	**2.4**
ffs]	0.2	0.1	0.8	0.1	0.3	0.2	4.0	2.9	**0.4**
F3P16_RS08040	0.1	0.1	0.5	0.1	0.2	0.1	1.8	0.7	**0.9**
recA	3.3	6.1	1.9	5.2	6.6	9.1	1.3	1.8	**1.6**
F3P16_RS08050	0.6	1.0	1.7	1.3	1.5	3.0	1.1	2.3	**2.1**
F3P16_RS08055	0.2	0.4	1.7	0.2	0.4	0.7	1.8	2.9	**1.1**
F3P16_RS08060	0.0	0.0	UC	0.0	0.0	0.0	UC	UC	**UC**
F3P16_RS08065	0.0	0.0	0.0	0.0	0.0	0.0	UC	UC	**0.0**
F3P16_RS08070	0.0	0.1	2.5	0.0	0.1	0.3	4.0	17.9	**0.5**
F3P16_RS08075	0.0	0.1	5.0	0.1	0.1	0.2	0.7	1.9	**8.7**
F3P16_RS08080	0.0	0.0	UC	0.0	0.0	0.0	0.5	0.6	**UC**
kynU	0.0	0.0	4.4	0.1	0.1	0.1	1.3	1.2	**11.1**
F3P16_RS08090	0.0	0.1	10.0	0.1	0.1	0.1	1.3	0.9	**6.1**
F3P16_RS08095	0.0	0.1	3.5	0.1	0.1	0.1	1.6	2.1	**4.0**
F3P16_RS19045	0.0	0.0	UC	0.0	0.0	0.0	UC	UC	**UC**
F3P16_RS08100	0.0	0.1	1.6	0.0	0.1	0.1	1.1	1.6	**1.2**
F3P16_RS08105	0.1	0.1	1.4	0.1	0.2	0.2	1.5	1.6	**1.9**
F3P16_RS08110	0.0	0.1	7.9	0.1	0.1	0.2	1.5	2.7	**6.2**

F3P16_RS08115	0.3	1.2	3.9	0.8	0.8	1.0	1.0	1.3	**2.4**
F3P16_RS08120	0.0	0.0	UC	0.1	0.0	0.1	0.7	1.1	**UC**
F3P16_RS08125	0.1	0.3	2.6	0.2	0.4	0.6	1.7	2.4	**2.0**
F3P16_RS08130	0.0	0.0	UC	0.0	0.1	0.1	7.0	9.1	**UC**
F3P16_RS08135	0.0	0.0	UC	0.0	0.0	0.1	0.2	2.5	**UC**
F3P16_RS08140	0.0	0.0	UC	0.0	0.0	0.0	0.0	3.4	**UC**
F3P16_RS08145	0.4	0.9	2.2	0.8	1.3	1.5	1.7	1.9	**1.8**
pfkB	0.3	0.8	2.5	0.6	1.2	1.2	2.0	2.0	**1.8**
ptsP	0.6	1.4	2.3	1.1	1.5	1.5	1.4	1.4	**1.8**
F3P16_RS08160	0.0	0.0	0.8	0.0	0.0	0.0	0.3	3.3	**0.5**
F3P16_RS08165	0.0	0.0	1.1	0.0	0.0	0.1	0.5	3.3	**5.0**
F3P16_RS08170	0.0	0.0	0.0	0.1	0.1	0.1	1.0	1.5	**4.5**
F3P16_RS08175	0.2	0.4	1.9	0.7	1.2	1.2	1.7	1.7	**3.3**
brnQ	0.2	0.6	2.6	0.6	1.0	1.0	1.7	1.7	**2.5**
map]	0.2	0.2	1.4	0.2	0.2	0.3	1.0	1.4	**1.6**
F3P16_RS08190	0.1	0.5	3.8	0.6	0.6	0.6	1.1	1.0	**4.6**
F3P16_RS08195	0.0	0.0	UC	0.0	0.1	0.1	1.5	1.7	**UC**
grxD	0.1	0.2	1.7	0.3	0.5	0.8	1.8	3.1	**2.2**
F3P16_RS08205	0.2	0.4	2.5	0.4	0.5	0.7	1.4	1.9	**2.3**
F3P16_RS08210	0.2	0.2	1.2	0.2	0.3	0.4	1.3	1.6	**1.3**
F3P16_RS08215	0.0	0.1	4.4	0.1	0.2	0.5	1.4	3.6	**5.6**
F3P16_RS08220	0.0	0.0	UC	0.0	0.1	0.0	1.3	0.6	**UC**
F3P16_RS08225	0.0	0.2	4.8	0.2	0.3	0.5	1.2	2.0	**6.2**
F3P16_RS08230	0.0	0.4	10.0	0.4	0.6	0.5	1.5	1.4	**10.2**
ftsY	0.9	1.6	1.9	2.0	2.5	3.2	1.2	1.6	**2.3**
F3P16_RS08240	0.0	0.0	UC	0.1	0.1	0.1	1.0	1.4	**UC**
F3P16_RS08245	0.0	0.0	1.2	0.1	0.1	0.1	1.0	1.6	**1.6**
F3P16_RS08250	3.3	1.5	0.4	1.3	2.0	4.0	1.5	3.0	**0.4**
cydX	5.7	3.9	0.7	3.2	4.1	7.4	1.3	2.3	**0.6**
cydB	16.0	10.2	0.6	5.6	8.1	10.7	1.4	1.9	**0.4**

F3P16_RS08265	12.0	10.3	0.9	4.8	8.0	9.6	1.7	2.0	**0.4**
F3P16_RS08270	0.8	0.8	1.0	0.4	0.8	1.1	1.8	2.4	**0.5**
F3P16_RS08275	0.0	0.1	2.0	0.1	0.1	0.1	2.1	2.4	**2.0**
F3P16_RS08280	0.2	0.4	2.1	0.4	0.7	0.5	1.7	1.3	**1.9**
F3P16_RS08285	0.4	0.7	1.7	0.7	1.0	1.0	1.5	1.6	**1.6**
F3P16_RS08290	0.0	0.1	1.6	0.0	0.1	0.3	3.1	10.3	**0.7**
F3P16_RS08295	1.5	3.0	2.0	2.4	4.0	3.2	1.7	1.3	**1.6**
F3P16_RS08300	0.5	0.8	1.6	0.9	1.3	1.3	1.4	1.4	**1.8**
F3P16_RS08305	0.1	0.4	4.3	0.3	0.4	0.4	1.4	1.4	**2.8**
F3P16_RS08310	0.1	0.2	2.4	0.4	0.4	0.4	1.1	1.1	**4.0**
ilvA	0.9	1.5	1.7	1.7	2.8	2.5	1.6	1.4	**1.9**
rpiA	0.9	1.6	1.8	2.1	3.6	2.3	1.7	1.1	**2.3**
F3P16_RS08325	0.4	0.7	1.7	1.0	2.2	1.2	2.3	1.2	**2.3**
F3P16_RS08330	0.2	0.3	1.6	0.3	0.6	0.4	1.9	1.2	**1.6**
argC	0.3	0.5	1.9	0.7	1.0	0.9	1.4	1.3	**2.7**
F3P16_RS08340	0.5	1.5	3.0	1.5	2.5	2.7	1.7	1.8	**2.8**
clpS	1.0	2.8	2.9	2.3	3.4	3.9	1.4	1.7	**2.4**
clpA	0.9	2.8	3.0	3.1	3.4	4.5	1.1	1.5	**3.3**
F3P16_RS08355	0.1	0.3	4.8	0.3	0.5	0.4	1.4	1.1	**5.3**
F3P16_RS08360	0.1	0.2	1.8	0.4	0.3	0.4	0.8	1.1	**3.0**
F3P16_RS08365	0.1	0.3	2.8	0.2	0.3	0.3	1.7	1.3	**2.1**
F3P16_RS08370	0.0	0.2	4.8	0.1	0.1	0.2	1.4	1.9	**2.1**
F3P16_RS08375	0.0	0.2	4.1	0.1	0.1	0.1	1.0	1.4	**1.6**
F3P16_RS08380	0.0	0.1	2.8	0.0	0.1	0.1	1.3	2.1	**1.7**
F3P16_RS08385	0.1	0.2	3.1	0.1	0.1	0.1	1.9	2.0	**1.3**
F3P16_RS08390	0.1	0.1	1.1	0.0	0.1	0.1	1.8	1.1	**0.9**
F3P16_RS08395	0.0	0.0	1.2	0.0	0.0	0.0	2.5	1.7	**0.7**
F3P16_RS08400	0.3	0.3	1.0	0.4	0.5	0.9	1.4	2.3	**1.3**
F3P16_RS08405	2.6	4.0	1.5	5.7	9.0	8.6	1.6	1.5	**2.1**
kdsA	2.5	5.2	2.1	5.6	13.4	12.3	2.4	2.2	**2.3**

eno]	0.9	2.2	2.3	2.2	4.8	5.7	2.1	2.5	**2.4**
F3P16_RS08420	0.0	0.0	1.5	0.1	0.1	0.1	1.0	1.3	**3.7**
ftsB	0.3	0.8	2.5	1.1	1.2	2.1	1.1	1.8	**3.4**
ispD	0.0	0.2	5.2	0.3	0.4	0.7	1.1	2.0	**7.5**
F3P16_RS08435	0.1	0.6	5.9	0.6	0.6	0.8	1.1	1.3	**5.4**
F3P16_RS08440	0.1	0.5	3.7	0.5	0.6	0.5	1.2	1.1	**3.7**
pcaF	0.2	0.6	3.4	0.5	0.6	0.6	1.2	1.2	**2.7**
F3P16_RS08450	0.0	0.2	5.2	0.2	0.2	0.2	1.5	1.2	**4.3**
pcaD	0.0	0.2	8.4	0.2	0.3	0.2	1.2	0.9	**10.0**
F3P16_RS08460	0.1	0.3	4.6	0.2	0.3	0.3	1.7	1.5	**2.9**
pcaC	0.1	0.3	5.8	0.2	0.4	0.2	1.5	1.0	**4.6**
pcaH	0.1	0.3	2.3	0.3	0.4	0.3	1.5	1.2	**2.4**
pcaG	0.1	0.3	2.9	0.3	0.3	0.3	1.1	1.2	**2.9**
aroD	0.0	0.2	5.3	0.1	0.2	0.2	1.4	1.0	**3.8**
F3P16_RS08485	0.0	0.1	5.2	0.1	0.2	0.1	1.6	1.3	**3.6**
F3P16_RS08490	0.0	0.1	20.3	0.0	0.1	0.1	2.3	2.3	**8.4**
F3P16_RS08495	0.2	0.6	2.5	0.4	0.5	0.6	1.3	1.5	**1.7**
F3P16_RS08500	0.0	0.0	0.5	0.0	0.0	0.0	UC	UC	**0.0**
F3P16_RS08505	0.0	0.0	1.1	0.0	0.0	0.0	0.8	0.6	**1.0**
F3P16_RS08510	0.0	0.0	1.1	0.0	0.0	0.1	2.5	4.3	**1.0**
F3P16_RS08515	0.0	0.0	2.1	0.0	0.0	0.0	0.7	1.0	**2.1**
pcaU	0.0	0.0	0.7	0.0	0.0	0.1	1.0	5.5	**0.5**
F3P16_RS08525	0.0	0.0	2.1	0.0	0.0	0.0	3.2	3.0	**1.7**
F3P16_RS08530	0.3	0.9	3.5	0.7	0.9	0.5	1.3	0.7	**2.6**
F3P16_RS08535	0.3	1.0	3.1	0.6	1.0	0.5	1.5	0.7	**2.0**
F3P16_RS08540	0.3	0.5	2.0	0.4	0.8	0.4	2.1	1.0	**1.4**
F3P16_RS08545	0.2	0.4	2.2	0.4	0.5	0.3	1.3	0.9	**2.2**
F3P16_RS08550	0.0	0.0	1.6	0.0	0.0	0.0	3.3	6.8	**1.0**
F3P16_RS08555	0.0	0.0	2.1	0.0	0.0	0.1	1.0	3.7	**13.9**
F3P16_RS08560	0.0	0.0	0.7	0.0	0.0	0.1	3.5	13.7	**0.2**

F3P16_RS08565	0.0	0.0	UC	0.0	0.0	0.0	0.1	0.0	**UC**
F3P16_RS08570	0.0	0.0	2.5	0.0	0.0	0.0	1.8	1.5	**1.8**
F3P16_RS08575	0.0	0.0	0.0	0.0	0.0	0.0	UC	UC	**0.0**
F3P16_RS08580	0.0	0.0	UC	0.0	0.0	0.0	1.0	1.1	**UC**
F3P16_RS08585	0.0	0.0	2.5	0.0	0.0	0.0	0.9	1.6	**1.8**
F3P16_RS08590	0.0	0.0	2.6	0.0	0.0	0.0	0.2	0.9	**3.0**
F3P16_RS08595	0.0	0.0	UC	0.0	0.0	0.0	1.0	6.3	**UC**
F3P16_RS08600	0.0	0.0	0.7	0.0	0.1	0.1	5.6	6.0	**0.3**
F3P16_RS08605	0.0	0.0	1.4	0.0	0.0	0.0	0.5	0.7	**2.3**
feaR	0.0	0.0	UC	0.0	0.0	0.0	0.0	1.5	**UC**
tynA	0.0	0.0	1.7	0.0	0.0	0.0	1.4	3.4	**0.4**
F3P16_RS08620	0.0	0.0	0.0	0.0	0.0	0.0	UC	UC	**0.0**
F3P16_RS08625	0.0	0.1	4.9	0.0	0.1	0.0	1.9	1.1	**3.4**
F3P16_RS08630	0.0	0.0	UC	0.0	0.0	0.0	0.7	1.2	**UC**
pcaD	0.0	0.1	2.7	0.1	0.2	0.2	1.9	2.1	**2.2**
pcaF	0.2	0.7	4.1	0.5	0.6	0.5	1.1	1.0	**3.3**
F3P16_RS08645	0.1	0.5	3.6	0.5	0.6	0.6	1.2	1.1	**3.6**
F3P16_RS08650	0.1	0.6	5.1	0.6	0.6	0.7	1.0	1.3	**4.6**
catA	0.1	0.2	3.7	0.3	0.2	0.2	0.9	0.6	**4.3**
catC	0.1	0.2	2.3	0.1	0.1	0.2	0.9	1.5	**1.7**
F3P16_RS08665	0.0	0.1	4.8	0.1	0.1	0.2	2.1	2.9	**3.8**
F3P16_RS08670	0.0	0.0	3.9	0.1	0.1	0.1	1.3	1.1	**6.3**
F3P16_RS08675	0.1	0.3	3.3	0.5	0.4	0.4	0.9	0.9	**5.6**
F3P16_RS08680	0.1	0.2	1.9	0.2	0.3	0.3	1.6	1.9	**2.0**
F3P16_RS08685	0.0	0.0	1.1	0.0	0.0	0.1	0.5	1.3	**1.9**
abaQ	0.0	0.0	0.9	0.0	0.0	0.0	2.8	5.7	**0.4**
F3P16_RS08695	0.0	0.1	2.9	0.0	0.1	0.1	2.0	1.7	**1.6**
F3P16_RS08700	0.1	0.2	1.9	0.1	0.2	0.3	1.7	2.3	**1.5**
F3P16_RS08705	0.0	0.0	UC	0.0	0.0	0.0	UC	UC	**UC**
F3P16_RS08710	0.0	0.2	5.7	0.1	0.1	0.1	1.2	1.3	**3.3**

F3P16_RS08715	0.0	0.0	1.1	0.0	0.0	0.0	2.8	4.6	**1.0**
F3P16_RS08720	0.0	0.1	3.1	0.1	0.1	0.4	0.8	3.3	**4.0**
F3P16_RS08725	0.0	0.0	UC	0.0	0.0	0.1	0.9	3.2	**UC**
F3P16_RS08730	0.0	0.0	UC	0.0	0.0	0.0	UC	UC	**UC**
F3P16_RS08735	0.0	0.0	0.0	0.0	0.0	0.0	0.0	0.9	**2.5**
F3P16_RS08740	0.0	0.1	3.2	0.1	0.0	0.1	0.4	1.1	**3.6**
F3P16_RS08745	0.1	0.1	1.3	0.2	0.2	0.4	0.8	1.8	**2.6**
F3P16_RS08750	0.1	0.1	1.2	0.1	0.1	0.2	0.8	1.7	**2.2**
F3P16_RS08755	0.0	0.1	5.6	0.1	0.1	0.2	1.2	1.4	**4.5**
F3P16_RS08760	0.0	0.0	2.7	0.1	0.1	0.0	1.9	0.5	**4.2**
F3P16_RS08765	0.0	0.0	UC	0.0	0.0	0.0	UC	UC	**UC**
F3P16_RS08770	0.0	0.1	UC	0.1	0.1	0.1	1.1	2.2	**UC**
F3P16_RS08775	0.0	0.0	UC	0.0	0.0	0.0	UC	UC	**UC**
F3P16_RS08780	0.0	0.0	UC	0.0	0.0	0.0	UC	UC	**UC**
F3P16_RS08785	0.0	0.0	UC	0.0	0.0	0.0	UC	UC	**UC**
F3P16_RS08790	0.0	0.0	UC	0.0	0.0	0.0	UC	UC	**UC**
F3P16_RS08795	0.0	0.0	UC	0.0	0.0	0.0	UC	UC	**UC**
F3P16_RS08800	0.0	0.0	UC	0.0	0.0	0.0	UC	UC	**UC**
F3P16_RS08805	0.0	0.0	UC	0.0	0.0	0.0	UC	UC	**UC**
F3P16_RS08815	0.0	0.0	UC	0.0	0.0	0.0	UC	UC	**UC**
F3P16_RS08820	0.0	0.0	UC	0.0	0.0	0.0	UC	UC	**UC**
F3P16_RS08830	0.0	0.0	UC	0.0	0.0	0.0	UC	UC	**UC**
F3P16_RS08835	0.0	0.0	UC	0.0	0.0	0.0	UC	UC	**UC**
F3P16_RS08840	0.0	0.0	UC	0.0	0.0	0.0	UC	UC	**UC**
F3P16_RS08845	0.0	0.0	1.1	0.0	0.0	0.0	2.5	3.4	**0.5**
F3P16_RS08850	0.0	0.0	UC	0.0	0.0	0.0	UC	UC	**UC**
F3P16_RS08855	0.0	0.0	1.1	0.1	0.0	0.1	0.0	1.9	**6.0**
F3P16_RS08860	0.0	0.0	UC	0.0	0.0	0.0	UC	UC	**UC**
F3P16_RS08865	0.0	0.0	UC	0.0	0.0	0.0	UC	UC	**UC**
F3P16_RS19050	0.0	0.0	UC	0.0	0.0	0.0	UC	UC	**UC**

F3P16_RS08875	0.0	0.0	UC	0.0	0.0	0.0	UC	UC	**UC**
F3P16_RS08880	0.0	0.0	UC	0.0	0.0	0.2	0.9	3.8	**UC**
F3P16_RS08885	0.1	0.1	1.1	0.1	0.1	0.1	0.6	1.2	**2.1**
F3P16_RS08890	0.0	0.0	2.0	0.0	0.1	0.1	1.7	2.7	**1.9**
F3P16_RS08895	0.0	0.0	0.8	0.1	0.0	0.1	0.7	2.0	**2.2**
F3P16_RS08900	0.0	0.0	UC	0.0	0.0	0.0	3.8	3.1	**UC**
F3P16_RS08905	0.0	0.0	1.4	0.0	0.0	0.0	0.9	4.7	**1.3**
F3P16_RS08910	0.0	0.1	2.7	0.0	0.1	0.1	1.2	2.8	**2.1**
F3P16_RS08915	0.0	0.0	0.0	0.0	0.0	0.1	UC	UC	**0.0**
F3P16_RS08920	0.0	0.0	0.5	0.0	0.0	0.0	0.8	0.3	**2.0**
F3P16_RS08925	0.0	0.1	2.0	0.1	0.1	0.1	1.6	1.7	**1.6**
F3P16_RS08930	0.0	0.0	6.5	0.0	0.0	0.1	1.3	3.5	**2.5**
F3P16_RS08935	0.0	0.0	5.6	0.0	0.0	0.0	0.5	1.1	**5.3**
F3P16_RS08940	0.0	0.0	UC	0.0	0.0	0.0	UC	UC	**UC**
F3P16_RS08945	0.0	0.0	1.1	0.0	0.0	0.0	UC	UC	**0.0**
F3P16_RS08950	0.0	0.0	UC	0.0	0.0	0.0	UC	UC	**UC**
F3P16_RS19055	0.7	0.7	1.1	0.2	0.2	0.6	1.3	3.7	**0.2**
F3P16_RS19060	0.0	0.0	0.0	0.0	0.0	0.0	UC	UC	**0.0**
F3P16_RS19065	0.0	0.0	UC	0.0	0.0	0.0	0.0	1.1	**UC**
F3P16_RS08960	0.0	0.3	6.2	0.2	0.3	0.5	1.7	2.6	**4.6**
F3P16_RS08965	0.0	0.5	12.4	0.2	0.4	0.5	1.9	2.2	**5.7**
F3P16_RS08970	0.0	0.0	UC	0.0	0.0	0.0	1.1	1.7	**UC**
F3P16_RS08975	0.1	0.2	1.8	0.1	0.2	0.3	1.4	2.2	**1.7**
F3P16_RS08980	0.1	0.2	3.0	0.2	0.3	0.3	1.6	2.0	**2.3**
F3P16_RS08985	0.0	0.0	UC	0.0	0.0	0.0	1.8	0.2	**UC**
F3P16_RS08990	0.0	0.0	0.9	0.1	0.1	0.1	1.4	1.6	**1.7**
F3P16_RS08995	0.0	0.0	1.3	0.0	0.0	0.0	6.0	8.6	**0.5**
F3P16_RS09000	0.0	0.0	5.3	0.1	0.0	0.1	0.4	1.0	**9.6**
F3P16_RS09005	0.0	0.0	UC	0.0	0.0	0.1	0.6	3.6	**UC**
F3P16_RS09010	0.0	0.0	7.4	0.0	0.0	0.1	1.4	3.0	**8.4**

F3P16_RS09015	0.1	0.0	0.6	0.0	0.1	0.1	1.9	3.0	**0.6**
F3P16_RS09020	0.2	0.5	2.1	0.3	0.7	0.8	2.0	2.3	**1.5**
F3P16_RS09025	0.0	0.1	4.3	0.1	0.1	0.1	1.4	1.7	**3.7**
F3P16_RS09030	0.0	0.0	1.5	0.0	0.0	0.1	0.8	4.1	**0.9**
F3P16_RS09035	0.1	0.1	0.6	0.1	0.1	0.1	0.9	0.8	**1.0**
F3P16_RS09040	0.0	0.0	UC	0.1	0.1	0.1	1.5	1.7	**UC**
F3P16_RS09045	0.0	0.0	0.0	0.0	0.0	0.0	8.0	4.6	**0.5**
F3P16_RS09050	0.0	0.0	1.1	0.0	0.0	0.1	1.0	6.1	**3.0**
F3P16_RS09055	0.0	0.0	UC	0.0	0.0	0.0	UC	UC	**UC**
F3P16_RS09060	0.5	0.6	1.3	0.6	1.2	1.0	2.1	1.8	**1.2**
F3P16_RS09065	0.7	0.9	1.3	0.8	1.2	1.2	1.5	1.5	**1.1**
F3P16_RS09070	0.3	0.3	0.9	0.2	0.5	0.4	2.1	1.8	**0.8**
F3P16_RS09075	0.1	0.1	2.2	0.0	0.1	0.1	1.8	2.9	**0.8**
F3P16_RS09080	0.0	0.0	UC	0.0	0.0	0.0	0.0	2.3	**UC**
F3P16_RS09085	0.0	0.0	0.5	0.0	0.0	0.0	1.0	2.1	**1.7**
F3P16_RS09090	0.0	0.0	2.1	0.0	0.0	0.1	0.8	5.0	**2.5**
F3P16_RS09095	0.0	0.0	0.4	0.0	0.0	0.0	1.0	2.0	**1.4**
F3P16_RS09100	0.0	0.1	5.3	0.0	0.0	0.1	0.9	2.0	**4.3**
F3P16_RS09105	0.0	0.0	1.1	0.0	0.0	0.0	0.7	0.5	**1.1**
F3P16_RS09110	0.0	0.0	UC	0.0	0.0	0.0	0.9	2.4	**UC**
F3P16_RS09115	0.0	0.1	7.7	0.0	0.1	0.1	4.8	5.5	**1.5**
F3P16_RS09120	0.0	0.0	1.1	0.0	0.1	0.1	5.0	7.4	**0.5**
F3P16_RS09125	0.0	0.0	UC	0.0	0.0	0.0	UC	UC	**UC**
F3P16_RS09130	0.0	0.0	UC	0.0	0.0	0.1	UC	UC	**UC**
F3P16_RS09135	0.0	0.0	UC	0.0	0.0	0.0	UC	UC	**UC**
F3P16_RS09140	0.0	0.0	UC	0.0	0.0	0.0	UC	UC	**UC**
F3P16_RS09145	0.0	0.0	UC	0.0	0.0	0.0	UC	UC	**UC**
F3P16_RS19070	0.0	0.0	UC	0.0	0.0	0.0	UC	UC	**UC**
F3P16_RS09155	0.0	0.0	UC	0.0	0.0	0.0	UC	UC	**UC**
F3P16_RS09160	0.0	0.0	UC	0.0	0.0	0.0	UC	UC	**UC**

F3P16_RS09165	0.0	0.0	UC	0.0	0.0	0.0	UC	UC	**UC**
F3P16_RS09170	0.0	0.0	UC	0.0	0.0	0.0	UC	UC	**UC**
F3P16_RS09175	0.0	0.0	0.9	0.0	0.0	0.1	1.3	2.1	**1.3**
F3P16_RS09180	0.0	0.0	3.0	0.0	0.0	0.0	1.1	1.1	**2.6**
F3P16_RS09185	0.0	0.0	4.9	0.0	0.0	0.0	4.5	5.7	**0.7**
F3P16_RS09190	0.0	0.0	0.5	0.0	0.0	0.0	UC	UC	**0.0**
adeT	0.0	0.0	UC	0.0	0.0	0.1	2.0	7.1	**UC**
adeS	0.0	0.0	1.2	0.0	0.0	0.1	1.7	8.5	**0.9**
adeR	0.0	0.0	2.1	0.0	0.0	0.1	2.3	4.6	**3.5**
adeA	0.4	1.4	3.6	0.3	2.1	7.7	7.0	25.6	**0.8**
adeB	0.4	1.6	3.8	0.6	2.6	9.2	4.3	15.4	**1.5**
F3P16_RS09220	0.0	0.0	UC	0.0	0.0	0.0	UC	UC	**UC**
F3P16_RS09225	0.0	0.0	UC	0.0	0.0	0.0	UC	UC	**UC**
F3P16_RS09230	0.7	0.4	0.6	0.1	0.2	0.3	1.5	2.1	**0.2**
F3P16_RS09235	0.0	0.0	0.6	0.0	0.1	0.1	2.2	4.1	**0.7**
F3P16_RS09240	0.1	0.1	2.4	0.1	0.2	0.2	1.3	1.4	**2.7**
F3P16_RS09245	0.0	0.0	2.1	0.0	0.0	0.0	UC	UC	**0.0**
F3P16_RS09250	0.0	0.0	UC	0.0	0.0	0.0	UC	UC	**UC**
F3P16_RS09255	0.3	0.5	1.5	0.3	0.4	0.6	1.3	2.1	**0.9**
F3P16_RS09260	0.1	0.2	1.8	0.1	0.1	0.1	1.2	2.2	**0.6**
F3P16_RS09265	0.0	0.1	2.1	0.0	0.1	0.1	2.6	4.1	**0.8**
F3P16_RS09270	0.0	0.0	UC	0.0	0.0	0.0	UC	UC	**UC**
F3P16_RS09275	0.0	0.0	0.0	0.1	0.1	0.0	1.1	0.6	**5.3**
F3P16_RS09280	0.2	0.5	3.2	0.3	0.4	0.5	1.3	1.6	**2.0**
F3P16_RS09285	0.0	0.1	2.5	0.2	0.1	0.3	0.6	1.7	**3.7**
F3P16_RS09290	0.0	0.0	UC	0.0	0.0	0.0	UC	UC	**UC**
F3P16_RS09300	0.1	0.4	3.8	0.5	0.7	0.5	1.6	1.1	**4.4**
pqqE	0.4	2.3	6.4	2.1	3.5	2.5	1.7	1.2	**6.0**
pqqD	0.5	3.3	6.8	3.2	5.4	3.9	1.7	1.2	**6.7**
pqqC	0.8	5.0	5.9	4.2	6.8	5.6	1.6	1.3	**5.0**

pqqB	0.4	2.2	5.3	2.3	3.2	2.7	1.3	1.1	**5.5**
pqqA	0.2	0.9	5.3	1.4	1.4	1.3	1.0	1.0	**7.7**
ppk1	0.1	0.2	2.4	0.3	0.4	0.2	1.3	0.8	**2.9**
F3P16_RS09335	0.0	0.1	6.7	0.1	0.0	0.0	0.8	0.9	**3.5**
F3P16_RS09340	0.0	0.0	UC	0.0	0.0	0.0	UC	UC	**UC**
F3P16_RS09345	0.0	0.0	6.3	0.0	0.1	0.0	1.4	0.6	**8.9**
F3P16_RS09350	0.0	0.0	0.0	0.0	0.0	0.0	UC	UC	**0.0**
F3P16_RS09355	0.0	0.0	UC	0.0	0.0	0.1	1.1	1.5	**UC**
modB	0.1	0.2	3.0	0.1	0.1	0.1	1.0	1.4	**1.7**
modA	0.0	0.0	1.7	0.1	0.1	0.1	1.0	1.8	**2.3**
F3P16_RS09370	0.0	0.0	UC	0.0	0.0	0.0	UC	UC	**UC**
antA	0.0	0.0	5.3	0.0	0.0	0.2	1.3	22.1	**3.0**
antB	0.0	0.0	11.6	0.0	0.0	0.1	0.7	1.6	**13.9**
antC	0.0	0.0	0.9	0.0	0.0	0.1	1.1	4.8	**1.1**
F3P16_RS09390	0.0	0.0	0.0	0.0	0.0	0.1	UC	UC	**0.0**
F3P16_RS09395	0.0	0.0	0.5	0.0	0.0	0.0	0.3	0.5	**2.2**
F3P16_RS09400	0.0	0.0	0.5	0.0	0.0	0.0	UC	UC	**0.0**
F3P16_RS09405	0.0	0.0	6.3	0.0	0.0	0.0	1.4	0.8	**6.9**
F3P16_RS09410	0.0	0.0	4.8	0.0	0.0	0.0	1.5	1.1	**1.0**
F3P16_RS09415	0.0	0.0	9.0	0.0	0.0	0.0	1.5	0.6	**2.0**
F3P16_RS09420	0.0	0.0	UC	0.0	0.0	0.0	UC	UC	**UC**
F3P16_RS09425	0.0	0.0	2.6	0.0	0.0	0.1	2.3	4.0	**2.5**
F3P16_RS09430	0.1	0.2	1.7	0.2	0.3	0.3	1.1	1.0	**1.9**
F3P16_RS09435	0.1	0.3	2.0	0.1	0.1	0.2	1.2	1.4	**0.9**
F3P16_RS09440	0.0	0.0	UC	0.0	0.0	0.0	UC	UC	**UC**
F3P16_RS09445	0.0	0.0	UC	0.0	0.0	0.0	UC	UC	**UC**
F3P16_RS09450	0.0	0.0	UC	0.0	0.0	0.0	UC	UC	**UC**
F3P16_RS09455	0.0	0.0	UC	0.0	0.0	0.0	UC	UC	**UC**
F3P16_RS18960	0.0	0.0	UC	0.0	0.0	0.0	UC	UC	**UC**
F3P16_RS09465	0.0	0.1	2.5	0.1	0.1	0.2	0.7	1.5	**3.4**

F3P16_RS09470	0.0	0.2	5.8	0.1	0.0	0.2	0.4	1.3	**4.0**
F3P16_RS09475	0.1	0.3	4.0	0.2	0.1	0.1	0.5	0.7	**2.6**
F3P16_RS09480	0.1	0.2	2.2	0.2	0.1	0.2	0.5	1.0	**2.1**
F3P16_RS09485	0.2	0.6	2.4	0.5	0.5	0.9	1.0	1.7	**2.2**
F3P16_RS09490	0.1	0.4	3.8	0.4	0.6	0.5	1.7	1.3	**3.8**
F3P16_RS09495	0.0	0.0	2.3	0.1	0.2	0.1	1.9	1.6	**4.2**
F3P16_RS09500	0.0	0.0	UC	0.0	0.0	0.1	UC	UC	**UC**
F3P16_RS09505	0.0	0.0	0.0	0.0	0.0	0.0	UC	UC	**0.0**
F3P16_RS09510	0.5	0.7	1.5	1.1	1.5	1.8	1.3	1.6	**2.4**
F3P16_RS09515	0.0	0.1	1.6	0.0	0.1	0.1	3.3	2.7	**1.0**
F3P16_RS09520	0.1	0.2	2.8	0.2	0.2	0.4	1.2	2.2	**3.1**
F3P16_RS09525	0.0	0.0	1.4	0.0	0.0	0.1	0.5	8.0	**0.7**
F3P16_RS09530	0.0	0.1	1.3	0.1	0.1	0.0	1.2	0.9	**1.1**
F3P16_RS09535	0.0	0.0	1.3	0.0	0.0	0.0	1.8	1.1	**0.8**
F3P16_RS09540	0.0	0.0	1.1	0.0	0.0	0.0	0.9	1.0	**1.5**
F3P16_RS09545	0.0	0.0	2.6	0.0	0.0	0.0	1.0	1.1	**3.4**
F3P16_RS09550	0.0	0.0	3.2	0.0	0.0	0.0	2.0	1.1	**2.0**
F3P16_RS09555	0.0	0.0	1.4	0.0	0.0	0.0	0.9	0.8	**1.6**
F3P16_RS09560	0.0	0.0	2.5	0.0	0.0	0.0	UC	UC	**0.0**
chrA	0.0	0.0	1.1	0.0	0.1	0.1	1.9	1.2	**1.1**
F3P16_RS09570	0.0	0.0	0.6	0.0	0.0	0.1	0.9	1.8	**1.3**
F3P16_RS09575	0.1	0.1	1.8	0.2	0.2	0.5	0.9	2.6	**2.5**
F3P16_RS09580	0.0	0.1	2.6	0.1	0.1	0.2	1.2	2.4	**2.9**
F3P16_RS09585	0.0	0.0	4.2	0.0	0.0	0.0	0.7	0.1	**5.5**
F3P16_RS09590	0.0	0.0	6.3	0.0	0.0	0.0	2.5	6.0	**2.0**
F3P16_RS09595	0.0	0.0	1.5	0.0	0.0	0.1	8.5	29.7	**0.2**
F3P16_RS09600	0.1	0.2	2.5	0.1	0.1	0.2	1.6	2.1	**0.9**
F3P16_RS19075	0.1	0.2	2.8	0.0	0.0	0.1	1.4	2.8	**0.4**
F3P16_RS09605	0.0	0.0	0.1	0.0	0.0	0.1	0.0	2.1	**1.5**
F3P16_RS09610	0.0	0.1	2.3	0.0	0.1	0.1	1.6	3.6	**1.2**

F3P16_RS09615	0.0	0.1	1.7	0.1	0.2	0.1	2.9	2.5	**1.7**
F3P16_RS09620	0.1	0.2	2.5	0.2	0.1	0.1	0.8	0.8	**2.7**
F3P16_RS09625	0.1	0.2	2.2	0.1	0.1	0.1	1.3	0.8	**0.7**
lpdA	0.1	0.5	5.8	0.2	0.2	0.1	1.2	0.7	**2.0**
F3P16_RS09635	0.3	1.0	3.2	0.4	0.3	0.2	0.8	0.4	**1.4**
F3P16_RS09640	0.3	0.8	2.8	0.3	0.2	0.1	0.7	0.4	**1.3**
F3P16_RS09645	0.1	0.2	2.9	0.1	0.1	0.0	0.5	0.2	**1.3**
lipA	0.0	0.0	3.5	0.0	0.0	0.0	0.6	2.1	**2.6**
F3P16_RS09655	0.0	0.1	2.6	0.1	0.1	0.1	0.9	2.0	**2.3**
F3P16_RS09660	0.1	0.1	1.0	0.1	0.2	0.5	1.2	3.2	**1.3**
F3P16_RS09665	0.1	0.1	1.5	0.1	0.2	0.2	1.8	2.1	**1.1**
aroC	0.4	0.9	2.2	1.2	1.9	1.6	1.5	1.3	**3.0**
prmB	0.4	0.8	1.9	1.1	1.4	1.3	1.3	1.2	**2.7**
trpB	0.2	0.4	2.4	0.4	0.5	0.4	1.4	1.2	**2.4**
F3P16_RS09685	0.4	1.0	2.3	0.9	1.2	0.6	1.4	0.7	**2.1**
F3P16_RS09690	0.5	1.2	2.4	1.2	1.4	1.1	1.2	0.9	**2.2**
F3P16_RS09695	0.4	1.0	2.8	0.9	1.1	1.6	1.2	1.8	**2.4**
F3P16_RS09700	0.4	0.6	1.5	0.3	0.6	0.8	1.8	2.4	**0.9**
F3P16_RS09705	0.0	0.1	UC	0.1	0.2	0.4	1.4	3.0	**UC**
F3P16_RS09710	0.1	0.1	0.9	0.1	0.1	0.2	1.4	2.0	**0.8**
F3P16_RS09715	0.0	0.0	UC	0.0	0.1	0.0	2.0	1.4	**UC**
F3P16_RS09720	0.2	0.3	1.7	0.3	0.5	0.4	1.5	1.3	**1.9**
recR	1.3	2.2	1.7	2.8	3.6	5.7	1.3	2.0	**2.2**
F3P16_RS09730	0.6	1.1	2.0	1.1	1.6	1.8	1.5	1.6	**2.0**
F3P16_RS09735	0.0	0.0	UC	0.0	0.0	0.1	2.0	5.1	**UC**
F3P16_RS09740	0.4	0.9	1.9	0.9	1.2	1.2	1.4	1.4	**2.0**
F3P16_RS09745	0.0	0.0	2.9	0.0	0.0	0.1	3.3	7.5	**1.7**
F3P16_RS09750	0.0	0.0	UC	0.0	0.0	0.0	UC	UC	**UC**
F3P16_RS09755	0.0	0.0	3.2	0.1	0.1	0.1	1.6	1.8	**6.9**
F3P16_RS09760	0.0	0.0	UC	0.0	0.0	0.1	2.0	2.3	**UC**

F3P16_RS09765	0.2	0.1	0.8	0.2	0.0	0.1	0.2	0.6	**1.2**
F3P16_RS09770	0.0	0.0	2.1	0.1	0.0	0.1	0.1	1.1	**7.4**
F3P16_RS09775	0.6	1.1	2.0	1.5	1.2	2.1	0.8	1.4	**2.7**
F3P16_RS09780	0.1	0.2	1.9	0.3	0.3	1.0	1.0	3.1	**3.3**
F3P16_RS09785	0.0	0.0	UC	0.1	0.1	0.1	0.9	1.6	**UC**
F3P16_RS09790	0.1	0.3	2.9	0.2	0.2	0.5	1.4	3.2	**1.6**
F3P16_RS09795	0.2	0.3	1.4	0.4	0.4	0.6	1.0	1.5	**2.1**
F3P16_RS09800	0.4	0.7	1.8	1.3	1.5	2.2	1.1	1.7	**3.3**
F3P16_RS09805	0.0	0.1	2.7	0.0	0.1	0.1	2.0	1.8	**1.5**
F3P16_RS09810	0.1	0.1	0.7	0.1	0.1	0.0	1.3	0.8	**0.7**
F3P16_RS09815	0.0	0.0	2.7	0.0	0.0	0.0	3.6	3.5	**0.6**
F3P16_RS09820	0.0	0.0	1.1	0.0	0.0	0.1	1.4	4.4	**0.9**
F3P16_RS09825	0.1	0.1	1.5	0.1	0.1	0.2	1.7	1.9	**1.4**
lpxB	0.2	0.4	2.0	0.2	0.3	0.6	1.4	2.4	**1.3**
F3P16_RS09835	0.0	0.0	UC	0.0	0.0	0.0	0.9	1.2	**UC**
F3P16_RS09840	0.0	0.0	0.5	0.0	0.0	0.0	UC	UC	**0.0**
F3P16_RS09845	0.0	0.0	1.7	0.0	0.0	0.0	2.3	4.8	**0.7**
F3P16_RS09850	0.0	0.0	UC	0.0	0.0	0.0	UC	UC	**UC**
F3P16_RS09855	0.2	0.3	1.4	0.2	0.1	0.3	0.7	2.3	**0.7**
F3P16_RS09860	0.4	0.6	1.5	0.8	0.8	1.2	1.0	1.5	**1.9**
F3P16_RS09865	0.0	0.0	UC	0.0	0.0	0.0	0.8	1.4	**UC**
F3P16_RS09870	0.0	0.1	3.0	0.1	0.1	0.1	1.3	1.3	**3.2**
F3P16_RS09875	0.1	0.2	1.6	0.2	0.3	0.3	1.5	1.3	**1.7**
cobT	0.2	0.4	1.8	0.3	0.4	0.4	1.3	1.3	**1.5**
cobU	0.1	0.2	3.4	0.1	0.2	0.2	2.8	3.0	**1.1**
F3P16_RS09890	0.0	0.0	1.4	0.0	0.1	0.0	3.8	1.5	**1.0**
F3P16_RS09895	0.0	0.1	7.4	0.0	0.0	0.0	1.1	0.7	**2.1**
F3P16_RS09900	0.0	0.0	3.0	0.0	0.0	0.0	2.7	2.3	**1.2**
F3P16_RS09905	0.0	0.0	UC	0.0	0.0	0.1	UC	UC	**UC**
F3P16_RS09910	0.0	0.0	0.8	0.0	0.0	0.2	6.4	25.5	**0.2**

F3P16_RS09915	0.0	0.0	UC	0.0	0.0	0.0	UC	UC	**UC**
F3P16_RS09920	0.2	0.2	1.4	0.1	0.3	0.4	3.4	5.1	**0.5**
F3P16_RS09925	0.1	0.2	1.9	0.1	0.1	0.1	1.6	0.9	**0.9**
F3P16_RS09930	0.0	0.0	4.2	0.1	0.1	0.1	2.4	1.3	**5.4**
F3P16_RS09935	0.0	0.1	4.0	0.1	0.2	0.1	2.4	1.4	**4.5**
F3P16_RS09940	0.0	0.1	1.8	0.1	0.1	0.1	1.9	1.7	**1.9**
F3P16_RS09945	0.0	0.0	1.3	0.0	0.1	0.1	1.5	3.2	**1.2**
F3P16_RS09950	0.0	0.0	1.0	0.0	0.1	0.1	1.7	2.1	**2.6**
F3P16_RS09955	0.0	0.0	4.9	0.0	0.0	0.0	2.0	2.1	**2.9**
ybaK	0.0	0.0	UC	0.0	0.1	0.0	3.8	1.1	**UC**
F3P16_RS09965	0.0	0.1	9.5	0.2	0.2	0.4	0.7	1.6	**21.8**
F3P16_RS09970	0.5	1.8	3.9	1.7	1.7	3.0	1.0	1.8	**3.6**
F3P16_RS09975	0.0	0.1	6.7	0.1	0.1	0.1	1.4	1.7	**5.1**
F3P16_RS09980	0.0	0.1	3.7	0.1	0.1	0.1	0.5	1.1	**3.9**
F3P16_RS09985	0.0	0.0	4.0	0.0	0.1	0.0	1.7	1.0	**4.1**
F3P16_RS09990	0.0	0.0	2.3	0.0	0.0	0.1	0.6	2.4	**2.3**
F3P16_RS09995	1.7	3.4	2.0	2.8	5.0	3.8	1.8	1.4	**1.6**
F3P16_RS10000	0.2	0.4	2.5	0.4	0.7	1.1	1.8	2.9	**2.3**
F3P16_RS10005	0.0	0.0	2.8	0.0	0.1	0.1	2.5	1.7	**2.6**
F3P16_RS10010	0.1	0.1	0.8	0.2	0.2	0.4	1.1	2.3	**1.8**
F3P16_RS10015	2.3	3.6	1.5	2.4	3.3	4.0	1.4	1.7	**1.0**
F3P16_RS10020	2.4	4.1	1.7	3.2	5.6	4.8	1.7	1.5	**1.4**
iscU	3.0	5.9	1.9	5.9	9.3	6.8	1.6	1.2	**1.9**
iscA	2.7	6.2	2.3	6.7	11.9	8.2	1.8	1.2	**2.5**
hscB	1.3	3.3	2.5	3.8	7.0	4.9	1.9	1.3	**2.9**
hscA	1.8	3.3	1.8	3.1	5.7	4.3	1.8	1.4	**1.7**
fdx]	0.5	1.1	2.1	1.5	3.0	2.3	2.0	1.5	**2.8**
F3P16_RS10050	0.1	0.2	2.3	0.2	0.3	0.3	1.4	1.6	**2.1**
F3P16_RS10055	0.2	0.4	1.8	0.4	0.6	0.7	1.4	1.8	**1.7**
mfd]	0.1	0.3	2.6	0.3	0.5	0.5	1.4	1.5	**3.1**

F3P16_RS10065	0.0	0.1	5.8	0.0	0.2	0.0	3.2	0.9	**5.0**
F3P16_RS10070	0.1	0.2	1.9	0.2	0.2	0.3	1.5	1.7	**1.5**
F3P16_RS10075	0.0	0.1	3.1	0.2	0.1	0.1	0.6	0.8	**4.6**
rraA	0.0	0.2	4.1	0.2	0.2	0.3	0.9	1.0	**5.3**
F3P16_RS10085	0.0	0.0	1.2	0.0	0.0	0.0	1.2	1.3	**1.4**
rpsT	0.3	0.7	2.0	2.4	2.5	5.3	1.1	2.2	**7.2**
F3P16_RS10095	0.1	0.2	2.2	0.3	0.2	0.2	0.8	0.7	**3.5**
F3P16_RS10100	0.0	0.1	1.5	0.1	0.1	0.1	1.2	1.1	**2.3**
F3P16_RS10105	0.0	0.0	1.1	0.1	0.1	0.2	1.1	3.4	**11.9**
F3P16_RS10110	0.0	0.1	3.3	0.2	0.2	0.3	0.9	1.7	**4.8**
F3P16_RS10115	0.1	0.2	1.8	0.1	0.3	0.2	2.2	1.5	**1.4**
F3P16_RS10120	0.1	0.2	1.2	0.1	0.2	0.1	2.2	1.0	**0.8**
F3P16_RS10125	0.1	0.3	2.3	0.2	0.5	0.3	2.5	1.5	**1.3**
F3P16_RS10130	0.1	0.2	1.7	0.1	0.2	0.2	1.8	1.9	**0.9**
F3P16_RS10135	0.1	0.1	1.4	0.1	0.1	0.1	1.5	1.4	**1.0**
F3P16_RS10140	0.0	0.0	1.8	0.0	0.0	0.1	0.8	2.0	**1.2**
F3P16_RS10145	0.0	0.0	0.6	0.0	0.0	0.0	0.9	2.9	**0.7**
F3P16_RS10150	0.0	0.0	1.2	0.0	0.0	0.0	4.0	6.8	**0.2**
F3P16_RS10155	0.1	0.1	1.2	0.1	0.1	0.1	1.3	1.8	**0.6**
F3P16_RS10160	0.2	0.2	1.2	0.1	0.1	0.1	1.1	1.0	**0.7**
F3P16_RS10165	0.2	0.1	0.5	0.1	0.1	0.1	1.3	2.0	**0.3**
F3P16_RS10170	0.0	0.0	UC	0.0	0.1	0.0	UC	UC	**UC**
F3P16_RS10175	0.6	1.6	2.8	1.1	1.1	1.1	1.0	0.9	**2.0**
F3P16_RS10180	0.3	0.5	1.7	0.5	0.7	0.9	1.4	1.7	**1.9**
F3P16_RS10185	0.0	0.0	1.9	0.0	0.0	0.1	1.0	1.8	**1.4**
F3P16_RS10190	0.0	0.0	3.4	0.0	0.0	0.0	6.7	6.1	**0.7**
F3P16_RS10195	0.2	0.1	0.9	0.1	0.2	0.3	1.6	2.7	**0.8**
pyrF	0.2	0.4	2.1	0.3	0.6	0.6	1.6	1.9	**1.7**
F3P16_RS10205	0.2	0.4	2.5	0.6	0.9	1.0	1.4	1.6	**4.3**
F3P16_RS10210	1.5	2.6	1.7	3.0	3.6	4.2	1.2	1.4	**2.0**

rpsA	12.1	20.9	1.7	33.6	39.1	61.6	1.2	1.8	**2.8**
cmk]	1.7	3.1	1.8	4.4	5.8	5.5	1.3	1.3	**2.6**
F3P16_RS10225	0.0	0.3	9.5	0.3	0.7	0.3	2.4	0.9	**9.4**
tadA	0.2	0.6	2.9	0.5	0.7	0.8	1.4	1.6	**2.6**
F3P16_RS10235	1.3	4.8	3.6	4.1	4.9	5.7	1.2	1.4	**3.1**
ung]	0.5	2.0	4.0	1.4	2.3	2.2	1.7	1.6	**2.7**
F3P16_RS10245	0.3	1.4	4.6	1.2	1.9	1.8	1.6	1.5	**3.9**
gspK	0.0	0.1	1.4	0.1	0.1	0.2	1.4	2.3	**1.3**
gspJ	0.0	0.1	2.2	0.0	0.1	0.1	1.8	2.0	**1.6**
gspI	0.0	0.1	UC	0.0	0.0	0.2	1.8	8.0	**UC**
F3P16_RS10265	0.0	0.1	1.7	0.1	0.3	0.2	2.6	2.1	**2.5**
F3P16_RS10270	0.2	0.5	2.6	0.5	0.9	1.0	1.6	1.8	**3.1**
F3P16_RS10275	0.3	0.4	1.5	0.6	1.0	1.0	1.8	1.7	**1.9**
F3P16_RS10280	0.0	0.1	3.5	0.1	0.2	0.2	2.1	1.9	**2.6**
F3P16_RS10285	0.1	0.2	1.8	0.3	0.5	0.5	1.8	1.7	**2.2**
kdsB	0.5	0.9	2.0	1.0	1.8	1.8	1.7	1.7	**2.3**
lpxK	0.4	1.0	2.9	0.9	1.7	1.4	1.8	1.5	**2.7**
msbA	0.9	2.9	3.2	3.2	4.6	3.6	1.4	1.1	**3.5**
F3P16_RS10305	0.2	0.3	1.5	0.4	0.8	0.9	1.9	2.2	**1.9**
F3P16_RS10310	0.0	0.1	1.7	0.1	0.1	0.4	1.0	2.8	**3.2**
F3P16_RS10315	0.5	0.8	1.6	0.5	1.0	1.0	2.0	2.0	**1.0**
F3P16_RS10320	0.3	0.7	2.1	0.4	0.8	1.2	1.8	2.8	**1.3**
rsmG	0.4	1.0	2.7	1.0	1.4	1.7	1.4	1.7	**2.6**
F3P16_RS10330	0.5	1.4	2.7	1.2	2.0	2.2	1.6	1.8	**2.3**
F3P16_RS10335	0.5	0.9	1.8	0.9	1.2	1.3	1.4	1.4	**1.9**
F3P16_RS10340	0.7	2.3	3.2	1.4	2.3	2.5	1.7	1.8	**1.9**
F3P16_RS10345	0.7	2.2	3.0	2.3	3.1	4.2	1.4	1.9	**3.1**
fba]	0.5	0.9	1.8	1.2	1.6	4.0	1.3	3.2	**2.4**
F3P16_RS10355	0.1	0.2	2.4	0.4	0.5	0.5	1.3	1.4	**3.8**
F3P16_RS10360	0.2	0.3	1.6	0.6	0.9	1.5	1.5	2.6	**2.8**

F3P16_RS10365	0.3	0.4	1.4	0.5	0.4	0.9	0.9	1.9	**1.5**
F3P16_RS10370	0.5	0.6	1.3	0.6	1.3	1.1	2.0	1.7	**1.3**
F3P16_RS10375	0.6	1.1	1.7	1.2	1.8	1.7	1.5	1.4	**1.9**
F3P16_RS10380	0.1	0.0	0.7	0.1	0.1	0.2	1.7	2.8	**1.2**
F3P16_RS10385	0.0	0.0	4.2	0.1	0.0	0.0	0.7	0.8	**6.0**
sstT	0.9	1.3	1.5	1.2	2.1	2.5	1.8	2.1	**1.3**
F3P16_RS10395	0.2	0.4	1.7	0.4	0.7	0.8	1.7	2.0	**1.7**
F3P16_RS10400	0.0	0.1	3.6	0.1	0.1	0.2	1.0	1.5	**6.1**
F3P16_RS10405	0.3	0.7	2.1	0.6	0.8	0.5	1.4	0.8	**1.8**
F3P16_RS10410	0.5	1.0	2.2	0.9	1.1	0.7	1.3	0.8	**1.9**
F3P16_RS10415	0.1	0.2	2.3	0.3	0.3	0.3	1.1	1.3	**2.8**
gcvH	0.3	0.5	1.6	0.7	0.9	0.8	1.4	1.2	**2.1**
F3P16_RS10425	0.0	0.0	2.1	0.0	0.1	0.1	2.4	3.0	**4.2**
putP	1.3	1.4	1.0	2.1	3.0	3.7	1.4	1.7	**1.6**
F3P16_RS10435	0.0	0.0	0.4	0.0	0.0	0.0	1.3	0.9	**1.3**
putA	4.2	4.9	1.2	5.7	8.5	5.8	1.5	1.0	**1.4**
F3P16_RS10445	0.0	0.0	0.7	0.0	0.0	0.0	0.5	0.8	**1.8**
trmA	0.2	0.5	2.2	0.4	0.7	0.7	1.7	1.6	**1.9**
F3P16_RS10455	0.0	0.1	UC	0.0	0.0	0.0	0.5	1.1	**UC**
F3P16_RS10460	0.0	0.0	1.2	0.0	0.0	0.0	1.8	2.1	**1.0**
F3P16_RS10465	0.2	0.6	2.7	0.7	0.8	0.7	1.3	1.1	**3.3**
F3P16_RS10470	0.3	0.9	3.3	1.3	1.0	2.6	0.8	2.1	**4.4**
F3P16_RS10475	0.0	0.1	2.8	0.1	0.1	0.2	1.3	1.8	**3.0**
tkt]	2.0	3.1	1.6	4.3	5.8	6.3	1.4	1.5	**2.2**
metK	0.9	1.0	1.1	1.4	1.6	3.0	1.2	2.2	**1.5**
F3P16_RS18965	0.0	0.0	UC	0.1	0.1	0.2	1.4	3.9	**UC**
F3P16_RS10495	0.0	0.0	UC	0.0	0.0	0.0	UC	UC	**UC**
F3P16_RS10500	0.2	0.7	2.7	0.5	0.4	1.2	0.8	2.4	**2.1**
F3P16_RS10505	0.0	0.3	10.7	0.2	0.1	0.3	0.9	2.0	**5.4**
F3P16_RS10510	0.1	0.3	4.8	0.1	0.2	0.2	1.5	1.5	**2.2**

F3P16_RS10515	0.1	0.1	1.8	0.1	0.2	0.3	1.9	3.3	**1.7**
ruvC	0.3	0.1	0.5	0.2	0.4	0.8	1.9	3.6	**0.7**
F3P16_RS10525	0.0	0.1	2.2	0.1	0.1	0.2	1.1	2.0	**1.9**
queG	0.1	0.1	1.5	0.1	0.2	0.2	2.7	2.9	**0.9**
bioB	1.1	1.5	1.3	1.5	1.7	2.5	1.2	1.7	**1.3**
F3P16_RS10540	0.0	0.1	3.4	0.1	0.1	0.1	2.6	2.0	**1.7**
F3P16_RS10545	0.0	0.0	0.5	0.0	0.0	0.0	0.5	1.1	**1.0**
F3P16_RS10550	0.0	0.0	3.7	0.0	0.0	0.0	UC	UC	**0.0**
F3P16_RS10555	0.0	0.0	0.0	0.0	0.0	0.1	UC	UC	**0.0**
F3P16_RS10560	0.0	0.0	1.7	0.0	0.0	0.1	4.8	7.7	**0.5**
F3P16_RS10565	0.0	0.0	0.8	0.0	0.0	0.1	7.5	15.4	**0.1**
F3P16_RS10575	0.0	0.0	UC	0.0	0.0	0.0	UC	UC	**UC**
F3P16_RS10580	0.0	0.1	2.5	0.1	0.0	0.1	0.8	2.2	**2.9**
F3P16_RS10585	0.4	0.9	2.2	0.5	0.6	0.2	1.3	0.3	**1.2**
F3P16_RS10590	0.1	0.1	0.8	0.0	0.2	0.1	3.6	1.9	**0.6**
F3P16_RS10595	0.0	0.1	2.2	0.0	0.1	0.1	1.4	3.1	**1.4**
F3P16_RS10600	0.2	0.3	2.1	0.6	0.9	0.7	1.4	1.1	**3.8**
F3P16_RS10605	0.0	0.0	2.3	0.0	0.0	0.1	0.8	1.8	**2.6**
F3P16_RS10610	0.0	0.0	UC	0.0	0.0	0.0	UC	UC	**UC**
F3P16_RS10615	0.0	0.0	UC	0.0	0.0	0.0	UC	UC	**UC**
F3P16_RS10620	0.0	0.0	0.8	0.0	0.1	0.0	14.0	8.0	**0.1**
F3P16_RS10625	0.0	0.0	UC	0.0	0.0	0.0	UC	UC	**UC**
F3P16_RS10630	0.0	0.0	15.8	0.0	0.1	0.0	3.8	1.4	**9.9**
F3P16_RS10635	0.1	0.1	1.3	0.1	0.2	0.3	1.6	2.3	**1.9**
F3P16_RS10640	0.0	0.0	3.2	0.0	0.1	0.1	2.4	2.0	**6.9**
F3P16_RS10645	0.0	0.0	1.1	0.0	0.0	0.0	UC	UC	**0.0**
F3P16_RS10650	0.4	0.3	0.7	0.3	0.3	0.5	1.1	1.7	**0.9**
F3P16_RS10655	0.2	0.3	1.7	0.2	0.2	0.2	1.3	1.4	**0.9**
F3P16_RS10660	0.4	0.5	1.3	0.3	0.4	0.4	1.3	1.3	**0.8**
F3P16_RS10665	0.3	0.6	2.3	0.3	0.3	0.4	1.1	1.4	**1.2**

F3P16_RS10670	0.1	0.2	1.3	0.2	0.3	0.4	1.3	1.7	**1.8**
F3P16_RS10675	0.0	0.0	0.9	0.0	0.0	0.0	4.0	6.1	**0.1**
F3P16_RS10680	0.1	0.1	1.2	0.0	0.1	0.1	1.3	1.7	**0.6**
F3P16_RS10685	0.0	0.0	2.0	0.0	0.0	0.0	4.7	4.6	**0.4**
F3P16_RS10690	0.0	0.0	1.0	0.0	0.0	0.0	3.8	1.1	**0.3**
F3P16_RS10695	0.0	0.1	1.2	0.1	0.0	0.1	0.8	0.9	**1.3**
F3P16_RS10700	0.0	0.0	0.8	0.0	0.1	0.1	2.1	1.7	**0.8**
F3P16_RS10705	0.0	0.0	1.4	0.0	0.1	0.0	2.7	2.0	**0.9**
ychF	1.3	1.7	1.3	1.4	2.3	2.8	1.6	2.0	**1.1**
F3P16_RS10715	0.0	0.0	1.6	0.0	0.0	0.0	2.3	4.2	**0.7**
F3P16_RS10720	0.0	0.0	UC	0.0	0.0	0.1	UC	UC	**UC**
F3P16_RS10725	0.1	0.2	2.1	0.2	0.2	0.3	1.0	1.8	**2.1**
F3P16_RS10730	0.0	0.0	5.3	0.0	0.0	0.0	0.4	1.3	**5.0**
F3P16_RS10735	0.0	0.1	2.7	0.1	0.1	0.2	0.7	1.8	**3.5**
ygaH	0.0	0.0	3.7	0.0	0.0	0.1	UC	UC	**0.0**
F3P16_RS10745	0.1	0.1	0.7	0.1	0.2	0.1	2.4	1.4	**1.0**
purT	0.6	1.6	2.6	1.2	1.5	1.6	1.3	1.3	**1.9**
glnD	0.5	1.2	2.3	1.0	1.4	1.1	1.4	1.2	**1.9**
dapC	0.8	1.5	1.9	1.1	1.7	1.3	1.6	1.2	**1.4**
F3P16_RS10765	0.0	0.0	UC	0.0	0.0	0.1	0.9	1.3	**UC**
F3P16_RS10770	0.1	0.8	5.8	0.5	0.6	0.8	1.1	1.6	**3.8**
F3P16_RS10775	0.2	1.2	6.3	0.8	1.0	1.4	1.2	1.6	**4.5**
msrB	0.1	0.6	5.4	0.6	0.6	0.8	1.0	1.2	**5.4**
F3P16_RS10785	0.2	0.2	0.9	0.1	0.2	0.6	1.8	4.5	**0.7**
F3P16_RS10790	0.1	0.2	2.5	0.3	0.1	0.2	0.4	0.9	**4.0**
F3P16_RS10795	0.6	0.8	1.3	0.9	1.0	3.6	1.1	4.1	**1.4**
dnaE	0.7	1.1	1.5	1.3	1.7	1.5	1.4	1.1	**1.9**
F3P16_RS10805	1.0	1.9	1.8	1.9	3.6	2.7	1.9	1.4	**1.9**
cysE	1.0	1.7	1.7	2.2	3.0	2.8	1.4	1.3	**2.2**
F3P16_RS10815	0.3	0.7	2.3	0.9	1.0	1.5	1.1	1.7	**2.8**

F3P16_RS10820	0.0	0.0	1.1	0.1	0.1	0.1	0.7	1.4	**2.3**
ahpF	0.3	0.5	2.1	0.5	0.6	1.1	1.3	2.2	**1.9**
F3P16_RS10830	0.1	0.1	1.5	0.0	0.0	0.1	0.8	4.4	**0.3**
F3P16_RS10835	0.0	0.0	1.0	0.0	0.0	0.1	1.0	8.0	**0.3**
F3P16_RS10840	0.0	0.1	10.0	0.0	0.1	0.1	4.7	9.1	**1.5**
arsB	0.0	0.1	10.6	0.0	0.0	0.0	0.7	1.1	**7.2**
F3P16_RS10850	0.0	0.0	UC	0.0	0.0	0.0	UC	UC	**UC**
arsC	0.0	0.0	UC	0.0	0.0	0.0	UC	UC	**UC**
F3P16_RS10860	0.0	0.1	UC	0.0	0.1	0.1	4.0	4.6	**UC**
F3P16_RS10865	0.1	0.2	2.7	0.2	0.2	0.2	1.1	1.4	**2.0**
F3P16_RS10870	0.0	0.1	1.7	0.1	0.1	0.1	1.0	2.0	**1.7**
F3P16_RS10875	0.0	0.0	UC	0.0	0.0	0.0	0.8	0.7	**UC**
F3P16_RS10880	0.0	0.1	4.9	0.0	0.0	0.1	5.5	11.4	**0.7**
F3P16_RS10885	0.0	0.0	0.8	0.0	0.0	0.1	1.5	3.3	**0.7**
F3P16_RS10890	0.1	0.1	0.5	0.1	0.1	0.0	1.4	0.3	**0.5**
tauD	2.0	1.2	0.6	0.6	1.1	0.8	1.7	1.2	**0.3**
tauC	1.9	1.4	0.8	0.7	1.0	0.7	1.3	1.0	**0.4**
F3P16_RS10905	0.9	0.7	0.8	0.4	0.4	0.5	0.9	1.0	**0.5**
tauA	0.6	0.4	0.7	0.2	0.2	0.2	0.8	1.2	**0.4**
F3P16_RS10915	0.0	0.1	2.5	0.1	0.1	0.1	0.9	0.9	**4.2**
F3P16_RS10920	0.0	0.0	0.4	0.0	0.0	0.0	0.3	2.4	**1.6**
F3P16_RS10925	0.0	0.0	1.0	0.0	0.1	0.1	3.1	4.0	**0.7**
F3P16_RS10930	0.1	0.1	1.9	0.1	0.1	0.2	1.3	2.0	**1.4**
F3P16_RS10935	0.0	0.0	3.5	0.1	0.2	0.2	1.3	1.3	**8.2**
F3P16_RS10945	1.2	2.6	2.1	1.8	2.4	4.1	1.3	2.3	**1.5**
cydB	1.6	3.1	2.0	2.4	3.1	4.8	1.3	2.0	**1.5**
F3P16_RS10955	0.8	0.7	0.9	0.4	1.0	0.9	2.3	2.2	**0.6**
F3P16_RS10960	0.1	0.2	2.3	0.1	0.2	0.2	1.4	1.8	**1.6**
F3P16_RS10965	0.0	0.1	3.1	0.1	0.1	0.1	1.4	1.1	**3.6**
madM	0.0	0.0	UC	0.0	0.0	0.0	UC	UC	**UC**

madL	0.0	0.0	UC	0.0	0.0	0.0	UC	UC	**UC**
mdcH	0.0	0.1	3.2	0.0	0.1	0.0	1.3	0.6	**2.4**
F3P16_RS10985	0.0	0.1	1.3	0.1	0.2	0.1	1.8	1.1	**1.9**
mdcE	0.1	0.1	1.6	0.1	0.2	0.2	1.9	1.4	**2.0**
F3P16_RS10995	0.0	0.1	6.2	0.1	0.1	0.0	0.8	0.5	**4.6**
F3P16_RS11000	0.0	0.1	9.5	0.1	0.1	0.1	0.8	0.9	**16.9**
F3P16_RS11005	0.0	0.1	4.1	0.1	0.1	0.1	1.3	0.7	**5.8**
mdcA	0.0	0.1	7.8	0.1	0.1	0.1	0.8	1.3	**14.6**
gcvA	0.0	0.0	UC	0.0	0.0	0.0	UC	UC	**UC**
F3P16_RS11020	0.0	0.0	UC	0.0	0.0	0.0	UC	UC	**UC**
F3P16_RS11025	0.0	0.0	0.8	0.0	0.0	0.1	1.3	5.4	**0.6**
F3P16_RS11030	0.0	0.0	1.1	0.0	0.0	0.1	2.3	8.0	**0.5**
F3P16_RS11035	0.0	0.0	0.5	0.0	0.0	0.1	7.5	11.4	**1.0**
F3P16_RS11040	0.0	0.0	1.5	0.0	0.0	0.1	2.7	9.3	**0.3**
F3P16_RS11045	0.0	0.0	2.1	0.0	0.0	0.1	1.4	8.2	**0.8**
F3P16_RS11050	0.0	0.0	UC	0.0	0.0	0.0	UC	UC	**UC**
F3P16_RS11055	0.0	0.0	2.6	0.0	0.0	0.0	2.9	2.6	**1.7**
yfcF	0.0	0.3	6.2	0.3	0.2	0.2	0.9	0.6	**5.5**
yghU	0.2	1.1	5.2	0.8	1.0	0.8	1.3	1.0	**3.8**
F3P16_RS11070	0.1	0.2	2.8	0.2	0.3	0.4	1.5	2.0	**2.3**
F3P16_RS11075	0.0	0.1	UC	0.0	0.0	0.0	1.8	3.2	**UC**
F3P16_RS11080	0.0	0.1	1.3	0.0	0.0	0.1	0.6	1.1	**1.1**
F3P16_RS11085	0.1	0.0	0.8	0.0	0.0	0.1	1.8	3.6	**0.4**
F3P16_RS11090	0.1	0.2	2.3	0.1	0.3	0.2	1.8	1.5	**1.7**
F3P16_RS11095	0.1	0.1	1.2	0.1	0.1	0.1	2.3	1.7	**0.9**
F3P16_RS11100	0.0	0.0	0.0	0.0	0.0	0.0	UC	UC	**0.0**
F3P16_RS11105	0.0	0.0	1.2	0.0	0.0	0.1	1.3	3.1	**1.2**
F3P16_RS11110	0.2	0.1	0.7	0.2	0.3	0.2	1.8	1.2	**0.9**
F3P16_RS11115	0.2	0.1	0.7	0.1	0.3	0.1	2.7	0.9	**0.6**
F3P16_RS11120	0.2	0.2	0.7	0.1	0.2	0.2	2.5	1.7	**0.4**

F3P16_RS11125	0.1	0.2	1.3	0.1	0.2	0.1	2.0	1.0	**0.7**
F3P16_RS11130	0.1	0.1	1.4	0.1	0.2	0.1	1.8	0.9	**1.1**
F3P16_RS11135	0.0	0.0	UC	0.0	0.0	0.1	UC	UC	**UC**
F3P16_RS11140	0.0	0.0	2.6	0.1	0.2	0.1	1.7	1.4	**8.9**
F3P16_RS11145	0.0	0.0	2.8	0.0	0.0	0.1	8.0	22.8	**0.7**
F3P16_RS11150	0.0	0.0	1.6	0.0	0.0	0.1	UC	UC	**0.0**
F3P16_RS11155	0.0	0.0	2.0	0.0	0.0	0.1	0.8	3.5	**1.0**
F3P16_RS11160	0.0	0.0	2.6	0.0	0.0	0.1	1.0	2.0	**2.2**
F3P16_RS11165	0.1	0.1	1.4	0.1	0.1	0.3	1.2	5.2	**0.6**
umuD	0.1	0.2	2.1	0.2	0.2	0.4	0.9	2.0	**2.3**
F3P16_RS11175	0.1	0.2	2.2	0.1	0.2	0.4	1.3	3.7	**1.7**
F3P16_RS19080	0.1	0.2	1.8	0.1	0.1	0.8	0.8	8.8	**1.0**
F3P16_RS19085	0.0	0.0	UC	0.0	0.0	0.0	UC	UC	**UC**
F3P16_RS11185	0.0	0.0	UC	0.0	0.0	0.0	UC	UC	**UC**
F3P16_RS11190	0.1	0.3	5.6	0.1	0.1	0.1	1.5	1.8	**1.0**
katE	0.7	4.4	6.0	1.5	2.0	1.7	1.4	1.2	**2.0**
F3P16_RS11200	0.0	0.5	19.1	0.2	0.2	0.3	0.9	1.8	**6.2**
F3P16_RS11205	0.0	0.0	UC	0.0	0.0	0.0	UC	UC	**UC**
F3P16_RS11210	0.0	0.6	UC	0.0	0.0	0.1	0.2	1.7	**UC**
F3P16_RS11215	0.1	0.5	9.4	0.2	0.1	0.1	0.7	0.7	**3.3**
F3P16_RS11220	0.0	0.0	UC	0.0	0.0	0.0	0.3	0.6	**UC**
F3P16_RS11225	0.0	0.0	UC	0.0	0.0	0.0	UC	UC	**UC**
F3P16_RS18895	0.0	0.0	UC	0.0	0.0	0.0	UC	UC	**UC**
F3P16_RS11230	0.0	0.0	UC	0.0	0.0	0.0	UC	UC	**UC**
F3P16_RS11235	0.0	0.0	0.4	0.1	0.2	0.1	2.1	1.1	**3.8**
F3P16_RS11240	0.1	0.0	0.8	0.1	0.1	0.1	1.1	0.6	**1.9**
F3P16_RS11245	0.0	0.0	0.7	0.0	0.0	0.0	1.2	3.2	**1.0**
F3P16_RS11250	0.0	0.0	2.0	0.0	0.0	0.0	0.5	0.8	**1.5**
F3P16_RS11255	0.0	0.1	5.5	0.0	0.0	0.0	0.4	1.0	**3.6**
F3P16_RS11260	0.0	0.2	3.4	0.1	0.0	0.1	0.4	0.9	**1.8**

F3P16_RS11265	0.1	0.8	8.5	0.2	0.1	0.3	0.3	1.1	**2.7**
F3P16_RS11270	0.3	3.2	10.5	0.9	0.1	0.4	0.1	0.4	**3.0**
F3P16_RS11275	0.5	5.3	10.3	1.5	0.4	0.3	0.3	0.2	**2.9**
F3P16_RS11280	0.3	2.0	7.1	0.4	0.1	0.1	0.4	0.2	**1.4**
F3P16_RS11285	0.2	1.6	8.8	0.5	0.2	0.1	0.4	0.3	**2.6**
F3P16_RS11290	0.2	1.4	7.2	0.4	0.2	0.2	0.6	0.5	**1.9**
F3P16_RS11295	0.2	0.6	2.3	0.6	0.4	0.6	0.8	1.0	**2.3**
F3P16_RS11300	0.2	0.8	4.2	0.7	0.5	0.4	0.7	0.6	**3.9**
F3P16_RS11305	0.4	0.9	2.6	0.6	0.5	0.9	0.9	1.6	**1.6**
F3P16_RS11310	0.1	0.3	3.6	0.2	0.3	0.2	1.6	1.4	**2.1**
F3P16_RS11315	0.0	0.0	1.1	0.0	0.0	0.2	0.2	3.8	**5.5**
F3P16_RS11320	0.0	0.0	6.3	0.0	0.0	0.0	6.0	13.1	**2.0**
F3P16_RS11325	0.0	0.0	UC	0.0	0.0	0.0	5.0	10.3	**UC**
F3P16_RS11330	0.0	0.0	0.4	0.0	0.0	0.0	0.5	0.4	**0.9**
F3P16_RS11335	0.0	0.1	10.6	0.0	0.0	0.0	5.0	7.4	**1.0**
F3P16_RS11340	0.0	0.0	5.5	0.0	0.0	0.1	1.1	4.0	**1.7**
F3P16_RS11345	0.0	0.1	5.5	0.0	0.0	0.1	1.6	4.0	**1.2**
F3P16_RS11350	0.0	0.1	3.5	0.0	0.0	0.1	1.9	3.7	**0.8**
F3P16_RS11355	0.0	0.0	8.4	0.0	0.0	0.0	0.5	1.3	**7.9**
F3P16_RS11360	0.0	0.1	2.1	0.0	0.0	0.1	0.9	1.8	**1.4**
F3P16_RS11365	0.0	0.1	2.1	0.0	0.1	0.1	1.1	1.6	**1.3**
F3P16_RS11370	0.2	0.2	1.3	0.2	0.3	0.4	1.3	1.6	**1.3**
F3P16_RS11375	0.0	0.1	3.7	0.0	0.1	0.1	1.9	2.8	**2.3**
F3P16_RS11380	0.0	0.1	2.8	0.1	0.1	0.1	1.4	1.8	**2.9**
F3P16_RS11385	0.0	0.0	3.4	0.0	0.0	0.0	0.7	2.7	**1.5**
F3P16_RS11390	0.1	0.1	1.5	0.1	0.1	0.3	1.2	2.6	**1.6**
F3P16_RS11395	0.0	0.1	22.2	0.1	0.0	0.2	0.6	2.4	**16.5**
F3P16_RS11400	0.0	0.1	3.0	0.0	0.0	0.0	1.6	3.2	**0.7**
F3P16_RS11405	0.0	0.1	3.2	0.0	0.0	0.1	1.2	2.0	**1.1**
F3P16_RS11410	0.0	0.1	2.3	0.1	0.1	0.1	1.4	1.5	**1.9**

phnW	0.1	0.2	2.1	0.1	0.2	0.3	2.0	3.1	**1.1**
F3P16_RS11420	0.0	0.0	UC	0.0	0.0	0.0	0.0	4.8	**UC**
F3P16_RS11425	0.0	0.0	6.9	0.0	0.0	0.0	3.3	5.1	**3.5**
F3P16_RS11430	0.0	0.0	0.8	0.0	0.0	0.0	6.5	12.5	**0.2**
F3P16_RS11435	0.0	0.0	0.8	0.0	0.0	0.0	2.0	9.9	**0.6**
F3P16_RS11440	0.0	0.1	3.0	0.1	0.0	0.1	0.6	1.1	**2.4**
pobA	0.0	0.1	5.5	0.1	0.0	0.1	0.2	0.7	**6.8**
pobR	0.0	0.1	2.6	0.1	0.1	0.1	1.0	1.4	**2.3**
F3P16_RS11455	0.0	0.0	UC	0.0	0.0	0.0	1.5	1.4	**UC**
F3P16_RS11460	0.1	0.1	1.9	0.1	0.2	0.3	2.0	3.6	**1.3**
F3P16_RS11465	0.0	0.0	0.0	0.0	0.1	0.1	3.7	4.6	**3.5**
F3P16_RS11470	0.0	0.0	1.6	0.1	0.0	0.1	0.8	1.2	**2.9**
F3P16_RS11475	0.0	0.0	6.3	0.0	0.0	0.0	0.3	2.2	**5.0**
F3P16_RS11480	0.0	0.0	2.6	0.0	0.0	0.0	0.9	1.7	**1.0**
F3P16_RS11485	0.0	0.0	UC	0.0	0.0	0.0	0.0	0.0	**UC**
F3P16_RS11490	0.1	0.2	4.1	0.1	0.1	0.1	1.3	2.0	**1.3**
F3P16_RS11495	0.1	0.2	2.4	0.1	0.2	0.2	2.4	2.8	**1.2**
F3P16_RS11500	0.0	0.1	4.2	0.0	0.1	0.3	1.7	6.4	**1.6**
F3P16_RS11505	0.0	0.2	3.5	0.1	0.1	0.6	0.9	4.5	**2.8**
F3P16_RS11510	0.0	0.2	5.0	0.1	0.1	1.2	1.0	9.7	**3.4**
F3P16_RS11515	0.1	0.3	2.4	0.3	0.2	0.4	0.9	1.6	**2.3**
F3P16_RS11520	0.1	0.2	2.2	0.2	0.2	0.3	0.9	1.1	**3.0**
F3P16_RS11525	0.1	0.2	2.0	0.1	0.1	0.2	1.1	1.5	**1.5**
F3P16_RS11530	0.1	0.4	5.7	0.2	0.2	0.3	0.9	1.5	**3.1**
F3P16_RS11535	0.0	0.0	UC	0.0	0.0	0.0	UC	UC	**UC**
F3P16_RS11540	0.0	0.0	UC	0.0	0.0	0.0	UC	UC	**UC**
F3P16_RS11545	0.0	0.0	UC	0.0	0.0	0.0	UC	UC	**UC**
F3P16_RS11550	0.0	0.0	UC	0.0	0.0	0.0	UC	UC	**UC**
F3P16_RS11555	0.0	0.0	UC	0.0	0.0	0.0	UC	UC	**UC**
F3P16_RS11560	0.0	0.0	UC	0.0	0.0	0.0	UC	UC	**UC**

F3P16_RS11565	0.0	0.0	UC	0.0	0.0	0.0	0.9	0.7	**UC**
F3P16_RS11570	0.0	0.0	UC	0.0	0.0	0.0	0.0	3.4	**UC**
F3P16_RS11580	0.0	0.0	UC	0.0	0.0	0.0	UC	UC	**UC**
F3P16_RS11585	0.0	0.0	UC	0.0	0.0	0.0	UC	UC	**UC**
F3P16_RS11590	0.0	0.0	UC	0.0	0.0	0.0	UC	UC	**UC**
F3P16_RS11595	0.0	0.0	5.3	0.0	0.0	0.0	0.0	2.9	**1.0**
F3P16_RS11600	0.1	0.3	1.9	0.2	0.2	0.3	1.1	1.7	**1.3**
paaX	0.2	0.7	3.1	0.5	0.6	0.7	1.1	1.4	**2.2**
paaF	0.2	0.4	2.9	0.3	0.2	0.5	0.5	1.5	**2.1**
pcaF	0.2	0.7	3.4	0.5	0.2	0.3	0.4	0.5	**2.7**
F3P16_RS11620	0.1	0.4	3.5	0.2	0.1	0.0	0.3	0.2	**2.0**
paaG	0.1	0.5	4.0	0.4	0.2	0.1	0.4	0.2	**3.0**
F3P16_RS11630	0.1	0.3	2.5	0.1	0.1	0.0	0.5	0.2	**1.3**
paaK	0.1	0.2	3.2	0.2	0.0	0.0	0.2	0.1	**3.5**
paaJ	0.3	1.2	3.6	1.1	0.3	0.1	0.3	0.1	**3.3**
paaC	0.2	0.5	2.9	0.5	0.1	0.0	0.3	0.1	**3.0**
paaB	0.1	0.2	2.3	0.3	0.1	0.0	0.4	0.0	**3.1**
paaA	0.1	0.4	3.8	0.3	0.2	0.1	0.7	0.2	**2.6**
paaZ	0.1	0.6	4.8	0.5	0.2	0.1	0.3	0.2	**4.6**
F3P16_RS11665	0.0	0.2	7.1	0.2	0.3	0.3	1.4	1.5	**5.6**
F3P16_RS11670	0.0	0.1	9.9	0.0	0.0	0.2	1.8	22.4	**1.3**
F3P16_RS11675	0.0	0.1	2.8	0.0	0.0	0.1	0.8	2.4	**1.3**
abaF	0.0	0.0	UC	0.0	0.0	0.1	1.1	4.7	**UC**
F3P16_RS11685	0.0	0.0	5.8	0.0	0.1	0.1	4.9	2.7	**4.5**
F3P16_RS11690	0.0	0.0	1.3	0.0	0.0	0.0	0.5	1.3	**0.9**
F3P16_RS11695	0.0	0.0	UC	0.0	0.0	0.1	UC	UC	**UC**
F3P16_RS11700	0.0	0.0	1.6	0.0	0.0	0.0	UC	UC	**0.0**
F3P16_RS11705	0.0	0.0	4.2	0.0	0.0	0.1	1.3	3.5	**2.5**
F3P16_RS11710	0.0	0.1	3.0	0.0	0.1	0.2	1.4	5.3	**2.0**
F3P16_RS11715	0.0	0.0	UC	0.0	0.0	0.0	1.3	6.8	**UC**

F3P16_RS11720	0.0	0.1	19.5	0.1	0.1	0.6	0.7	6.1	**29.3**
F3P16_RS11730	0.0	0.1	9.5	0.1	0.2	0.3	3.2	5.4	**6.9**
soxR	0.0	0.1	1.3	0.1	0.2	0.2	2.0	2.6	**1.9**
F3P16_RS11740	0.0	0.0	UC	0.0	0.0	0.0	UC	UC	**UC**
F3P16_RS11745	0.0	0.0	UC	0.0	0.0	0.0	UC	UC	**UC**
F3P16_RS11750	0.0	0.0	UC	0.0	0.0	0.0	UC	UC	**UC**
F3P16_RS11755	0.0	0.0	UC	0.0	0.0	0.0	UC	UC	**UC**
F3P16_RS11760	0.0	0.0	UC	0.0	0.0	0.0	UC	UC	**UC**
F3P16_RS11765	0.0	0.0	UC	0.0	0.0	0.0	UC	UC	**UC**
F3P16_RS11770	0.0	0.0	UC	0.0	0.0	0.0	UC	UC	**UC**
F3P16_RS11775	0.0	0.0	UC	0.0	0.0	0.0	UC	UC	**UC**
F3P16_RS11780	0.0	0.0	UC	0.0	0.0	0.0	UC	UC	**UC**
F3P16_RS11785	0.0	0.0	UC	0.0	0.0	0.0	UC	UC	**UC**
icmH	0.0	0.0	UC	0.0	0.0	0.0	UC	UC	**UC**
tssK	0.0	0.0	UC	0.0	0.0	0.0	UC	UC	**UC**
tssA	0.0	0.0	UC	0.0	0.0	0.0	UC	UC	**UC**
tssH	0.0	0.0	UC	0.0	0.0	0.0	UC	UC	**UC**
F3P16_RS11810	0.0	0.0	UC	0.0	0.0	0.0	UC	UC	**UC**
F3P16_RS19090	0.0	0.0	UC	0.0	0.0	0.0	UC	UC	**UC**
F3P16_RS11815	0.0	0.0	UC	0.0	0.0	0.0	UC	UC	**UC**
tagF	0.0	0.0	UC	0.0	0.0	0.0	UC	UC	**UC**
tssM	0.0	0.0	UC	0.0	0.0	0.0	UC	UC	**UC**
F3P16_RS11830	0.0	0.0	UC	0.0	0.0	0.0	UC	UC	**UC**
tssG	0.0	0.0	UC	0.0	0.0	0.0	UC	UC	**UC**
tssF	0.0	0.0	UC	0.0	0.0	0.0	UC	UC	**UC**
tssE	0.0	0.0	UC	0.0	0.0	0.0	UC	UC	**UC**
F3P16_RS11850	0.0	0.0	UC	0.0	0.0	0.0	UC	UC	**UC**
tssC	0.0	0.0	UC	0.0	0.0	0.0	UC	UC	**UC**
tssB	0.0	0.0	UC	0.0	0.0	0.0	UC	UC	**UC**
F3P16_RS11865	0.0	0.0	UC	0.0	0.0	0.0	UC	UC	**UC**

F3P16_RS11880	0.1	0.2	1.9	0.2	0.6	0.4	2.6	1.6	**2.1**
F3P16_RS11885	0.1	0.2	2.1	0.2	0.3	0.3	1.5	1.4	**2.4**
F3P16_RS11890	0.0	0.1	5.3	0.1	0.1	0.1	1.0	0.9	**5.0**
F3P16_RS11895	0.0	0.0	1.3	0.0	0.0	0.0	2.6	2.6	**0.6**
atzF	0.0	0.0	2.5	0.0	0.0	0.0	1.7	2.2	**1.1**
F3P16_RS11905	0.0	0.0	0.0	0.0	0.0	0.0	UC	UC	**0.0**
F3P16_RS11910	0.0	0.0	UC	0.0	0.0	0.0	UC	UC	**UC**
F3P16_RS11915	0.0	0.0	0.0	0.0	0.0	0.0	0.0	0.0	**1.2**
F3P16_RS11920	0.0	0.0	0.7	0.0	0.0	0.0	0.0	0.7	**2.6**
uca]	0.0	0.0	2.5	0.0	0.0	0.1	2.9	11.0	**0.7**
F3P16_RS11930	0.0	0.0	UC	0.0	0.0	0.0	0.5	1.1	**UC**
F3P16_RS11935	0.0	0.0	1.1	0.0	0.0	0.0	UC	UC	**0.0**
F3P16_RS11940	0.0	0.1	18.0	0.0	0.0	0.1	1.0	5.8	**4.0**
F3P16_RS11945	0.0	0.0	2.1	0.1	0.0	0.1	0.1	0.9	**3.8**
F3P16_RS11950	0.0	0.0	0.0	0.0	0.0	0.0	UC	UC	**0.0**
F3P16_RS11955	0.0	0.0	UC	0.0	0.0	0.0	UC	UC	**UC**
F3P16_RS11960	0.0	0.0	UC	0.0	0.0	0.0	UC	UC	**UC**
F3P16_RS11965	0.1	0.0	0.2	0.0	0.0	0.0	8.0	10.8	**0.0**
F3P16_RS11970	0.0	0.0	UC	0.0	0.0	0.0	UC	UC	**UC**
F3P16_RS11975	0.1	0.2	1.5	0.2	0.3	0.3	1.7	1.6	**1.2**
F3P16_RS11980	0.0	0.1	2.6	0.1	0.1	0.1	1.6	1.6	**1.4**
F3P16_RS11985	0.0	0.2	3.3	0.1	0.1	0.1	0.8	1.0	**1.9**
F3P16_RS11990	0.2	0.6	3.0	0.3	0.3	0.2	0.8	0.7	**1.7**
F3P16_RS11995	0.2	0.6	3.9	0.4	0.3	0.3	0.6	0.7	**2.8**
F3P16_RS12000	0.1	0.3	2.8	0.1	0.0	0.1	0.5	0.9	**0.8**
mumR	0.0	0.0	4.6	0.0	0.0	0.1	1.0	2.0	**2.8**
F3P16_RS12010	0.0	0.0	0.8	0.1	0.1	0.0	1.3	0.4	**1.7**
F3P16_RS12015	0.0	0.0	UC	0.0	0.0	0.0	UC	UC	**UC**
F3P16_RS12020	0.0	0.0	UC	0.0	0.0	0.0	UC	UC	**UC**
F3P16_RS12025	0.0	0.0	UC	0.0	0.0	0.0	UC	UC	**UC**

F3P16_RS12030	0.0	0.0	UC	0.0	0.0	0.0	UC	UC	**UC**
F3P16_RS12035	0.0	0.0	UC	0.0	0.0	0.0	2.0	1.6	**UC**
F3P16_RS12040	0.8	1.7	2.2	1.4	2.6	1.3	1.8	0.9	**1.8**
F3P16_RS12050	0.0	0.0	1.7	0.0	0.0	0.0	0.3	1.3	**1.4**
F3P16_RS12055	0.0	0.0	UC	0.0	0.0	0.0	1.0	0.6	**UC**
F3P16_RS12060	0.0	0.0	0.6	0.0	0.0	0.0	UC	UC	**0.0**
F3P16_RS12065	0.0	0.0	4.2	0.0	0.0	0.0	UC	UC	**0.0**
F3P16_RS12070	0.0	0.0	13.7	0.0	0.0	0.0	1.8	2.1	**11.9**
F3P16_RS12075	0.0	0.0	UC	0.0	0.0	0.0	1.0	1.1	**UC**
F3P16_RS12080	0.0	0.0	UC	0.0	0.0	0.0	UC	UC	**UC**
F3P16_RS12085	0.0	0.0	UC	0.0	0.0	0.0	UC	UC	**UC**
F3P16_RS12090	0.0	0.0	UC	0.0	0.0	0.0	UC	UC	**UC**
F3P16_RS12095	0.0	0.0	UC	0.0	0.0	0.0	UC	UC	**UC**
F3P16_RS12100	0.0	0.1	4.8	0.0	0.0	0.0	0.6	1.0	**4.3**
F3P16_RS12105	0.0	0.0	1.4	0.0	0.0	0.0	UC	UC	**0.0**
F3P16_RS12110	0.0	0.0	UC	0.0	0.0	0.0	UC	UC	**UC**
F3P16_RS12115	0.0	0.0	UC	0.0	0.0	0.0	UC	UC	**UC**
F3P16_RS12120	0.0	0.0	UC	0.0	0.0	0.0	UC	UC	**UC**
F3P16_RS12125	0.0	0.0	UC	0.0	0.0	0.0	UC	UC	**UC**
F3P16_RS12130	0.0	0.0	UC	0.0	0.0	0.0	UC	UC	**UC**
F3P16_RS12135	0.0	0.0	0.1	0.0	0.0	0.1	UC	UC	**0.0**
F3P16_RS12140	0.0	0.0	UC	0.0	0.0	0.0	UC	UC	**UC**
F3P16_RS12145	0.0	0.0	UC	0.0	0.0	0.0	UC	UC	**UC**
F3P16_RS12150	0.0	0.0	UC	0.0	0.0	0.0	UC	UC	**UC**
F3P16_RS12155	0.3	1.3	3.8	1.6	1.6	2.0	1.0	1.3	**4.5**
F3P16_RS12160	0.1	0.1	1.9	0.0	0.1	0.1	1.7	1.8	**0.7**
F3P16_RS12165	0.0	0.0	0.0	0.0	0.0	0.0	UC	UC	**0.0**
F3P16_RS12170	0.0	0.0	UC	0.0	0.0	0.0	6.0	0.0	**UC**
mutS	0.6	1.1	1.8	0.9	1.6	1.4	1.7	1.5	**1.5**
F3P16_RS12180	0.1	0.2	2.4	0.1	0.5	0.3	4.1	2.7	**1.4**

F3P16_RS12185	0.0	0.0	UC	0.0	0.1	0.2	2.3	3.8	**UC**
F3P16_RS12190	0.3	5.4	21.1	3.4	4.3	2.7	1.3	0.8	**13.0**
F3P16_RS12195	0.1	0.4	4.6	0.3	0.5	0.3	1.9	1.2	**3.2**
F3P16_RS12200	0.3	1.2	3.6	1.0	1.0	1.3	0.9	1.2	**3.0**
F3P16_RS12205	0.3	0.6	1.9	0.3	0.5	0.4	1.4	1.3	**1.0**
F3P16_RS12210	0.2	0.2	0.8	0.2	0.3	0.4	1.6	2.3	**0.7**
F3P16_RS12215	0.8	1.0	1.3	0.5	1.1	1.0	2.3	2.1	**0.6**
F3P16_RS12220	0.7	1.0	1.4	0.4	0.7	0.9	1.6	2.0	**0.6**
F3P16_RS12225	0.4	0.8	1.8	0.3	0.5	0.4	1.9	1.6	**0.6**
lptB	0.4	1.3	3.3	0.9	1.1	1.9	1.3	2.1	**2.3**
lptA	1.1	3.1	2.8	2.1	2.7	4.4	1.3	2.1	**1.9**
lptC	0.7	2.1	3.0	1.9	2.8	3.8	1.4	2.0	**2.8**
F3P16_RS12245	0.7	1.7	2.4	1.4	1.9	2.4	1.4	1.7	**2.0**
F3P16_RS12250	1.9	5.0	2.6	3.4	4.6	5.8	1.3	1.7	**1.8**
cysS	0.9	0.8	0.9	1.0	1.2	2.4	1.2	2.5	**1.1**
F3P16_RS12260	0.0	0.1	UC	0.2	0.1	0.7	0.5	4.1	**UC**
F3P16_RS12265	0.0	0.0	UC	0.0	0.0	0.0	UC	UC	**UC**
F3P16_RS12270	0.1	0.2	3.1	0.2	0.3	0.3	2.0	1.8	**2.5**
F3P16_RS12275	0.0	0.1	UC	0.1	0.1	0.1	1.3	0.8	**UC**
F3P16_RS12285	0.1	0.2	3.0	0.5	0.8	0.7	1.4	1.2	**6.5**
F3P16_RS12290	0.0	0.0	UC	0.0	0.0	0.0	0.3	1.1	**UC**
F3P16_RS12295	0.0	0.1	7.2	0.0	0.0	0.1	2.1	5.6	**2.1**
F3P16_RS12300	0.0	0.0	3.6	0.0	0.0	0.1	0.5	1.3	**4.0**
F3P16_RS12305	0.0	0.0	UC	0.0	0.0	0.0	UC	UC	**UC**
F3P16_RS12310	0.0	0.0	UC	0.0	0.0	0.0	0.0	3.4	**UC**
F3P16_RS12320	0.0	0.1	UC	0.3	0.1	0.1	0.5	0.5	**UC**
F3P16_RS12325	0.1	0.5	4.1	0.4	0.7	0.8	1.9	2.2	**3.2**
F3P16_RS12330	0.1	0.2	1.6	0.2	1.1	0.3	5.2	1.2	**1.8**
F3P16_RS12335	0.4	1.0	2.8	1.4	4.1	6.1	2.8	4.2	**4.1**
F3P16_RS12340	0.0	0.0	UC	0.0	0.0	0.0	1.0	2.3	**UC**

F3P16_RS12345	0.0	0.1	2.3	0.1	0.1	0.1	1.3	1.7	**1.3**
F3P16_RS12350	0.1	0.2	3.5	0.1	0.1	0.3	0.9	4.3	**1.0**
F3P16_RS12355	0.1	0.1	1.2	0.1	0.2	0.5	1.8	5.1	**1.8**
F3P16_RS12360	0.1	0.3	2.6	1.0	2.2	1.5	2.1	1.5	**7.9**
F3P16_RS12365	3.7	6.3	1.7	9.8	14.9	18.3	1.5	1.9	**2.7**
xseA	0.1	0.3	3.8	0.2	0.3	0.3	1.4	1.3	**2.8**
F3P16_RS12375	0.0	0.1	UC	0.1	0.1	0.1	1.2	0.7	**UC**
F3P16_RS12380	0.0	0.1	5.3	0.0	0.0	0.0	UC	UC	**0.0**
F3P16_RS12385	0.0	0.0	2.1	0.0	0.0	0.1	0.2	3.1	**2.7**
F3P16_RS12395	0.0	0.2	7.4	0.2	0.1	0.8	0.6	4.4	**5.8**
F3P16_RS12400	0.0	0.1	2.8	0.0	0.0	0.3	2.0	12.5	**0.8**
F3P16_RS12405	0.0	0.1	1.6	0.0	0.1	0.1	2.7	3.2	**1.2**
cueR	0.2	0.3	1.8	0.2	0.2	0.2	1.2	1.1	**1.0**
F3P16_RS12415	0.7	0.7	0.9	0.6	0.8	0.3	1.3	0.6	**0.8**
F3P16_RS12420	0.5	0.5	1.0	0.7	0.7	0.3	1.0	0.4	**1.5**
F3P16_RS12425	0.0	0.0	UC	0.0	0.0	0.0	UC	UC	**UC**
F3P16_RS19095	0.0	0.0	UC	0.0	0.0	0.0	UC	UC	**UC**
F3P16_RS12430	0.0	0.0	UC	0.0	0.0	0.0	UC	UC	**UC**
F3P16_RS12435	0.0	0.0	UC	0.0	0.0	0.0	UC	UC	**UC**
F3P16_RS12440	0.0	0.0	UC	0.0	0.0	0.0	UC	UC	**UC**
F3P16_RS12445	0.0	0.0	UC	0.0	0.0	0.0	UC	UC	**UC**
F3P16_RS12450	0.0	0.0	UC	0.0	0.0	0.0	UC	UC	**UC**
F3P16_RS12455	0.0	0.0	UC	0.0	0.0	0.0	UC	UC	**UC**
F3P16_RS12460	0.0	0.0	UC	0.0	0.0	0.0	UC	UC	**UC**
F3P16_RS12465	0.2	0.2	1.3	0.2	0.4	0.2	1.7	0.9	**1.2**
benA	0.0	0.2	6.1	0.1	0.1	0.1	0.8	0.6	**4.0**
benB	0.0	0.1	3.5	0.1	0.0	0.1	0.3	0.4	**3.3**
F3P16_RS12480	0.0	0.1	13.7	0.1	0.1	0.1	0.5	0.6	**18.4**
F3P16_RS12485	0.0	0.1	3.8	0.1	0.1	0.1	1.6	1.2	**3.0**
benE	0.0	0.1	2.0	0.0	0.0	0.1	1.5	1.5	**0.9**

F3P16_RS12495	0.0	0.0	1.8	0.0	0.0	0.0	0.9	2.9	**1.7**
F3P16_RS12500	0.0	0.0	1.2	0.0	0.0	0.1	2.9	7.5	**1.2**
F3P16_RS12505	0.7	1.0	1.4	1.1	1.9	1.8	1.7	1.7	**1.5**
hrpA	2.4	3.0	1.3	3.3	5.4	3.8	1.6	1.1	**1.4**
ahpC	1.4	2.5	1.8	3.6	5.0	8.5	1.4	2.4	**2.6**
F3P16_RS12520	0.0	0.1	9.5	0.1	0.1	0.2	1.0	2.3	**12.9**
F3P16_RS12525	0.1	0.2	2.1	0.1	0.3	0.5	2.6	4.8	**0.9**
F3P16_RS12530	0.1	0.1	1.7	0.2	0.2	0.4	1.3	2.2	**1.9**
ahpF	0.3	0.5	1.9	0.5	0.7	1.1	1.3	2.3	**1.8**
F3P16_RS12540	0.9	2.0	2.1	2.4	2.8	3.9	1.2	1.6	**2.5**
F3P16_RS12545	0.1	0.2	2.4	0.3	0.6	0.6	1.8	1.8	**3.6**
F3P16_RS12550	0.0	0.0	7.1	0.0	0.0	0.0	4.2	4.3	**1.2**
trmB	0.1	0.2	1.6	0.2	0.2	0.4	1.1	1.6	**1.5**
F3P16_RS12560	0.0	0.0	UC	0.0	0.0	0.0	UC	UC	**UC**
F3P16_RS12565	0.0	0.0	UC	0.0	0.0	0.0	UC	UC	**UC**
F3P16_RS12570	0.0	0.0	UC	0.0	0.0	0.0	UC	UC	**UC**
F3P16_RS12575	0.0	0.0	UC	0.0	0.0	0.0	UC	UC	**UC**
F3P16_RS12580	0.0	0.1	3.9	0.1	0.1	0.1	1.1	1.0	**3.0**
F3P16_RS12585	0.0	0.1	3.3	0.1	0.1	0.1	0.7	1.1	**3.2**
F3P16_RS12590	0.2	1.0	4.2	0.5	0.7	0.7	1.3	1.3	**2.1**
F3P16_RS12595	0.8	1.3	1.5	1.5	2.4	2.9	1.6	1.9	**1.8**
F3P16_RS12600	0.8	1.2	1.5	1.1	1.9	1.7	1.7	1.5	**1.4**
F3P16_RS12605	0.0	0.0	2.1	0.0	0.0	0.1	0.7	2.1	**6.0**
rlmK	1.2	2.2	1.9	1.8	3.2	2.8	1.8	1.6	**1.5**
F3P16_RS12615	0.0	0.1	1.2	0.1	0.1	0.2	1.4	1.9	**2.3**
F3P16_RS12620	0.0	0.1	4.2	0.1	0.1	0.1	1.4	1.4	**5.0**
clpB	1.9	3.2	1.7	2.4	2.6	10.4	1.1	4.2	**1.3**
F3P16_RS12630	0.0	0.1	3.3	0.1	0.1	0.3	1.1	3.8	**3.7**
F3P16_RS12635	0.0	0.1	UC	0.0	0.0	0.2	1.4	5.7	**UC**
F3P16_RS12640	0.2	0.2	1.3	0.3	0.5	0.6	1.7	2.1	**1.8**

F3P16_RS18970	0.1	0.4	6.3	0.2	0.3	0.9	1.8	4.5	**3.5**
crp]	0.3	0.4	1.3	0.4	0.7	0.9	1.7	2.0	**1.5**
F3P16_RS12650	0.2	1.0	5.1	0.8	0.8	1.5	0.9	1.7	**4.4**
F3P16_RS12655	0.4	6.6	18.2	3.4	4.3	4.9	1.3	1.4	**9.4**
F3P16_RS12660	0.0	0.0	2.6	0.0	0.1	0.1	4.0	2.1	**3.0**
F3P16_RS12665	4.9	8.8	1.8	11.2	21.0	26.5	1.9	2.4	**2.3**
F3P16_RS12670	2.0	4.2	2.1	4.1	7.5	5.2	1.8	1.3	**2.0**
F3P16_RS12675	0.0	0.0	UC	0.0	0.0	0.0	0.8	0.7	**UC**
F3P16_RS12680	0.1	0.2	1.6	0.1	0.1	0.3	0.8	2.0	**1.4**
alaS	3.2	3.6	1.1	3.1	5.3	5.9	1.7	1.9	**1.0**
F3P16_RS12690	6.9	9.3	1.3	10.0	15.2	24.5	1.5	2.5	**1.4**
csrA	0.0	0.2	8.7	0.3	0.6	0.9	1.9	2.8	**12.7**
rnhB	0.1	0.4	3.8	0.6	0.9	1.7	1.5	3.0	**6.1**
F3P16_RS12705	0.3	0.7	2.7	0.8	1.2	2.3	1.4	2.7	**3.3**
F3P16_RS12710	0.0	0.1	4.4	0.1	0.2	0.4	1.3	3.0	**5.8**
F3P16_RS12715	1.1	1.1	1.0	1.2	1.8	2.5	1.6	2.2	**1.1**
F3P16_RS12720	1.4	1.8	1.3	1.2	2.2	2.5	1.9	2.1	**0.9**
F3P16_RS12725	0.0	0.0	0.0	0.0	0.0	0.0	UC	UC	**0.0**
F3P16_RS12730	0.0	0.1	1.6	0.1	0.1	0.1	1.4	1.4	**1.7**
F3P16_RS12735	0.0	0.0	1.2	0.1	0.1	0.1	1.2	1.2	**1.4**
F3P16_RS12740	0.0	0.0	0.6	0.0	0.0	0.0	3.0	6.8	**0.8**
F3P16_RS12745	0.0	0.0	1.6	0.0	0.1	0.1	10.8	12.0	**0.5**
F3P16_RS12750	0.0	0.0	0.0	0.0	0.0	0.0	3.0	9.1	**0.5**
F3P16_RS12755	0.0	0.0	0.6	0.0	0.0	0.0	1.0	3.6	**0.3**
F3P16_RS12760	0.0	0.1	1.3	0.0	0.1	0.1	3.5	3.5	**0.6**
F3P16_RS12765	0.0	0.0	1.3	0.0	0.1	0.1	2.0	2.1	**1.2**
F3P16_RS12770	0.0	0.0	UC	0.0	0.0	0.0	UC	UC	**UC**
F3P16_RS12775	0.0	0.0	UC	0.0	0.0	0.0	UC	UC	**UC**
F3P16_RS12780	0.0	0.0	UC	0.0	0.0	0.0	UC	UC	**UC**
F3P16_RS12785	0.0	0.0	UC	0.0	0.0	0.0	UC	UC	**UC**

F3P16_RS12790	0.0	0.0	UC	0.0	0.0	0.0	UC	UC	**UC**
F3P16_RS12795	0.0	0.0	UC	0.0	0.0	0.0	UC	UC	**UC**
F3P16_RS12800	0.0	0.0	UC	0.0	0.0	0.0	UC	UC	**UC**
F3P16_RS12805	0.0	0.0	UC	0.0	0.0	0.0	UC	UC	**UC**
F3P16_RS12810	0.0	0.0	UC	0.0	0.0	0.0	UC	UC	**UC**
F3P16_RS12815	0.0	0.0	UC	0.0	0.0	0.0	UC	UC	**UC**
F3P16_RS12820	0.0	0.0	UC	0.0	0.0	0.0	UC	UC	**UC**
F3P16_RS12825	0.0	0.0	UC	0.0	0.0	0.0	UC	UC	**UC**
F3P16_RS12830	0.0	0.0	UC	0.0	0.0	0.0	UC	UC	**UC**
F3P16_RS12835	0.0	0.0	UC	0.0	0.0	0.0	UC	UC	**UC**
F3P16_RS12840	0.0	0.0	UC	0.0	0.0	0.0	UC	UC	**UC**
mhpT	0.0	0.0	UC	0.0	0.0	0.0	UC	UC	**UC**
F3P16_RS12850	0.0	0.0	UC	0.0	0.0	0.0	UC	UC	**UC**
F3P16_RS12855	0.0	0.0	UC	0.0	0.0	0.0	UC	UC	**UC**
F3P16_RS12860	0.0	0.0	UC	0.0	0.0	0.0	UC	UC	**UC**
F3P16_RS12865	0.0	0.0	2.1	0.0	0.0	0.0	0.0	0.6	**2.0**
F3P16_RS12870	0.0	0.0	UC	0.0	0.0	0.0	UC	UC	**UC**
F3P16_RS12875	0.0	0.0	UC	0.0	0.0	0.0	UC	UC	**UC**
F3P16_RS12880	0.0	0.0	UC	0.0	0.0	0.0	UC	UC	**UC**
F3P16_RS12885	0.0	0.0	UC	0.0	0.0	0.0	UC	UC	**UC**
F3P16_RS12890	0.0	0.0	UC	0.0	0.0	0.0	UC	UC	**UC**
F3P16_RS12895	0.0	0.0	1.1	0.0	0.0	0.0	UC	UC	**0.0**
kdgD	0.0	0.0	UC	0.0	0.0	0.0	UC	UC	**UC**
gudD	0.0	0.0	UC	0.0	0.0	0.0	UC	UC	**UC**
F3P16_RS12910	0.0	0.0	UC	0.0	0.0	0.0	UC	UC	**UC**
garD	0.0	0.0	UC	0.0	0.0	0.0	UC	UC	**UC**
F3P16_RS12920	0.0	0.0	UC	0.0	0.0	0.0	UC	UC	**UC**
F3P16_RS12925	0.0	0.0	UC	0.0	0.0	0.0	UC	UC	**UC**
F3P16_RS12930	0.0	0.0	UC	0.0	0.0	0.0	UC	UC	**UC**
F3P16_RS12935	0.0	0.0	UC	0.0	0.0	0.0	UC	UC	**UC**

F3P16_RS12940	0.0	0.0	UC	0.0	0.0	0.0	UC	UC	**UC**
F3P16_RS12945	0.0	0.0	UC	0.0	0.0	0.0	UC	UC	**UC**
F3P16_RS12950	0.0	0.0	UC	0.0	0.0	0.0	UC	UC	**UC**
F3P16_RS12955	0.0	0.0	UC	0.0	0.0	0.0	UC	UC	**UC**
astA	0.0	0.0	UC	0.0	0.0	0.0	UC	UC	**UC**
F3P16_RS12965	0.7	3.0	4.2	2.3	3.5	3.1	1.5	1.4	**3.2**
F3P16_RS12970	0.0	0.0	UC	0.0	0.0	0.0	UC	UC	**UC**
F3P16_RS12975	0.0	0.0	UC	0.0	0.0	0.0	UC	UC	**UC**
F3P16_RS12980	0.0	0.0	UC	0.0	0.0	0.0	UC	UC	**UC**
F3P16_RS12985	0.0	0.0	UC	0.0	0.0	0.0	UC	UC	**UC**
F3P16_RS12990	0.0	0.0	UC	0.0	0.0	0.0	UC	UC	**UC**
F3P16_RS12995	0.0	0.0	UC	0.0	0.0	0.0	UC	UC	**UC**
F3P16_RS13000	0.0	0.0	UC	0.0	0.0	0.0	UC	UC	**UC**
F3P16_RS13005	0.0	0.0	1.8	0.0	0.0	0.0	0.7	2.4	**1.3**
F3P16_RS13010	0.0	0.0	UC	0.0	0.0	0.0	UC	UC	**UC**
F3P16_RS13015	0.0	0.0	UC	0.0	0.0	0.0	UC	UC	**UC**
F3P16_RS13020	0.0	0.0	UC	0.0	0.0	0.0	UC	UC	**UC**
F3P16_RS13035	0.0	0.0	UC	0.0	0.0	0.0	UC	UC	**UC**
F3P16_RS13040	0.0	0.0	UC	0.0	0.0	0.0	UC	UC	**UC**
F3P16_RS13045	0.3	0.6	1.9	0.4	1.1	0.6	2.5	1.3	**1.3**
F3P16_RS13050	1.4	2.5	1.8	1.5	3.5	1.6	2.3	1.1	**1.1**
F3P16_RS13055	0.0	0.0	UC	0.0	0.0	0.1	1.3	5.7	**UC**
F3P16_RS18905	0.0	0.0	0.0	0.1	0.1	0.1	1.5	1.9	**1.5**
F3P16_RS13060	0.0	0.0	UC	0.0	0.0	0.0	UC	UC	**UC**
F3P16_RS13065	0.4	0.5	1.3	0.5	0.7	1.0	1.4	2.1	**1.2**
F3P16_RS13070	0.1	0.1	1.2	0.1	0.1	0.2	1.5	2.6	**1.3**
F3P16_RS13075	0.0	0.0	2.0	0.0	0.1	0.1	2.3	2.9	**0.9**
F3P16_RS13080	0.0	0.0	UC	0.0	0.0	0.1	UC	UC	**UC**
F3P16_RS13085	0.0	0.1	6.2	0.1	0.1	0.2	0.9	1.9	**4.0**
F3P16_RS13090	0.0	0.0	UC	0.0	0.0	0.0	UC	UC	**UC**

argG	0.8	1.0	1.2	1.7	2.1	3.4	1.2	2.0	**2.1**
F3P16_RS13100	0.0	0.0	1.6	0.0	0.0	0.0	2.0	1.9	**1.1**
pyrC	1.1	1.7	1.5	1.5	2.4	3.5	1.7	2.4	**1.3**
rnt]	1.4	2.1	1.4	2.2	2.8	4.2	1.3	1.9	**1.5**
F3P16_RS13115	0.4	0.2	0.6	0.3	0.4	1.1	1.5	3.7	**0.8**
F3P16_RS13120	0.1	0.1	1.1	0.0	0.1	0.2	2.9	4.5	**0.5**
F3P16_RS13125	0.3	0.9	2.8	0.6	0.4	0.9	0.7	1.4	**1.9**
F3P16_RS13130	0.0	0.0	1.9	0.0	0.1	0.1	1.7	1.8	**2.0**
F3P16_RS13135	0.1	0.2	1.6	0.1	0.2	0.3	1.7	2.1	**1.1**
yejB	0.0	0.1	2.5	0.1	0.1	0.1	1.9	1.6	**2.2**
F3P16_RS13145	0.1	0.2	2.5	0.2	0.4	0.3	1.9	1.8	**2.3**
F3P16_RS13150	1.3	3.7	2.8	3.0	4.1	3.9	1.4	1.3	**2.3**
dnaQ	0.1	0.2	1.3	0.2	0.4	0.3	1.5	1.4	**1.7**
F3P16_RS13160	0.1	0.0	0.5	0.1	0.0	0.1	0.9	1.9	**0.8**
nudC	0.1	0.4	2.8	0.2	0.4	0.6	1.7	3.0	**1.7**
F3P16_RS13170	0.3	1.1	4.1	1.4	1.6	3.1	1.2	2.3	**5.0**
F3P16_RS13175	0.3	1.6	6.0	1.5	2.0	4.3	1.3	2.9	**5.7**
F3P16_RS13180	0.0	0.0	0.0	0.0	0.0	0.0	2.0	4.6	**1.0**
rsmI	0.1	0.2	1.7	0.2	0.2	0.3	1.3	1.4	**1.3**
F3P16_RS13190	0.0	0.0	3.2	0.0	0.0	0.1	1.5	3.4	**4.0**
F3P16_RS13195	0.0	0.0	0.7	0.0	0.0	0.0	2.8	6.6	**0.3**
F3P16_RS13200	0.0	0.0	1.4	0.1	0.0	0.1	0.6	2.0	**2.0**
F3P16_RS13205	0.1	0.1	0.8	0.1	0.2	0.3	1.5	2.6	**1.1**
F3P16_RS13210	0.0	0.1	2.0	0.0	0.0	0.2	1.5	12.8	**0.4**
F3P16_RS13215	0.2	0.2	0.8	0.1	0.3	1.0	2.1	7.4	**0.6**
F3P16_RS13220	1.1	1.8	1.7	1.7	2.7	1.8	1.6	1.1	**1.6**
F3P16_RS13225	1.7	3.0	1.7	2.5	3.5	3.0	1.4	1.2	**1.4**
pepP	0.7	1.2	1.9	1.0	1.6	1.1	1.6	1.1	**1.6**
F3P16_RS13235	0.1	0.3	2.4	0.4	0.6	0.4	1.8	1.0	**2.8**
F3P16_RS13240	0.2	0.6	3.0	0.6	0.6	1.2	1.1	2.2	**2.7**

F3P16_RS13245	0.1	0.3	2.3	0.3	0.5	1.0	2.0	3.7	**1.9**
ssrS	0.1	0.2	1.8	0.4	0.2	1.0	0.4	2.3	**3.7**
F3P16_RS13255	0.0	0.1	3.9	0.0	0.1	0.5	1.4	12.5	**2.2**
F3P16_RS13260	0.0	0.0	UC	0.0	0.0	0.0	UC	UC	**UC**
F3P16_RS13265	0.0	0.1	2.0	0.1	0.2	0.2	2.2	2.2	**1.9**
F3P16_RS13270	0.0	0.0	UC	0.0	0.0	0.0	UC	UC	**UC**
F3P16_RS13275	0.0	0.0	UC	0.0	0.0	0.0	1.0	0.0	**UC**
umuD	0.0	0.0	UC	0.0	0.0	0.0	UC	UC	**UC**
F3P16_RS13285	0.0	0.0	UC	0.0	0.0	0.0	UC	UC	**UC**
F3P16_RS13290	0.0	0.0	UC	0.0	0.0	0.0	UC	UC	**UC**
F3P16_RS13295	0.0	0.0	UC	0.0	0.0	0.0	0.0	1.7	**UC**
F3P16_RS13300	0.0	0.0	3.2	0.0	0.0	0.1	0.7	3.4	**3.0**
F3P16_RS13305	0.0	0.1	1.7	0.1	0.1	0.2	1.5	3.3	**1.6**
F3P16_RS13310	0.0	0.0	1.3	0.1	0.1	0.1	1.6	2.3	**3.5**
F3P16_RS13315	0.0	0.0	0.6	0.0	0.0	0.1	1.2	1.9	**1.2**
F3P16_RS13320	0.0	0.0	2.6	0.0	0.0	0.0	UC	UC	**0.0**
F3P16_RS13325	0.0	0.0	UC	0.0	0.0	0.0	UC	UC	**UC**
F3P16_RS13330	0.1	0.1	1.8	0.1	0.1	0.2	1.5	1.9	**1.6**
F3P16_RS13335	0.0	0.0	UC	0.0	0.0	0.0	0.0	0.9	**UC**
F3P16_RS13340	0.0	0.0	UC	0.0	0.0	0.0	UC	UC	**UC**
F3P16_RS13345	0.0	0.0	UC	0.0	0.0	0.0	UC	UC	**UC**
F3P16_RS13350	0.0	0.0	UC	0.0	0.0	0.0	UC	UC	**UC**
F3P16_RS13360	0.0	0.0	UC	0.0	0.0	0.0	UC	UC	**UC**
F3P16_RS13365	0.0	0.0	UC	0.0	0.0	0.0	UC	UC	**UC**
F3P16_RS13370	0.0	0.0	UC	0.0	0.0	0.0	UC	UC	**UC**
F3P16_RS13380	0.0	0.1	1.5	0.0	0.0	0.1	8.0	44.5	**0.1**
F3P16_RS13385	0.0	0.0	2.4	0.0	0.0	0.1	3.0	10.8	**0.6**
F3P16_RS13390	0.0	0.0	0.4	0.0	0.0	0.0	0.5	3.2	**0.5**
F3P16_RS13395	0.0	0.0	UC	0.0	0.0	0.2	3.5	17.1	**UC**
F3P16_RS13400	0.0	0.0	UC	0.0	0.0	0.0	UC	UC	**UC**

F3P16_RS13405	0.0	0.0	UC	0.0	0.0	0.0	1.0	3.4	**UC**
F3P16_RS13410	0.0	0.0	4.2	0.0	0.0	0.1	1.3	2.0	**4.0**
F3P16_RS13415	0.0	0.0	3.5	0.0	0.0	0.1	UC	UC	**0.0**
F3P16_RS13420	0.0	0.0	UC	0.0	0.0	0.0	UC	UC	**UC**
F3P16_RS13425	0.0	0.0	3.7	0.0	0.0	0.1	0.3	1.7	**4.0**
F3P16_RS13430	0.0	0.0	2.1	0.0	0.0	0.0	UC	UC	**0.0**
F3P16_RS13435	0.0	0.0	UC	0.0	0.0	0.1	0.0	2.1	**UC**
F3P16_RS13440	0.0	0.0	1.6	0.0	0.0	0.0	1.9	2.6	**1.7**
F3P16_RS13445	0.0	0.0	1.3	0.0	0.0	0.1	0.9	3.2	**0.9**
F3P16_RS13450	0.0	0.0	1.1	0.0	0.0	0.1	6.0	12.5	**0.5**
F3P16_RS18910	0.0	0.1	UC	0.1	0.0	0.1	0.3	1.5	**UC**
F3P16_RS13455	0.0	0.0	UC	0.0	0.0	0.1	1.5	4.6	**UC**
F3P16_RS13460	0.0	0.0	1.9	0.0	0.0	0.1	2.3	3.9	**0.9**
F3P16_RS13465	0.0	0.0	24.3	0.0	0.0	0.1	0.9	3.5	**11.9**
F3P16_RS13470	0.0	0.0	1.8	0.0	0.0	0.1	3.2	7.5	**0.5**
F3P16_RS13475	0.0	0.0	UC	0.0	0.0	0.0	2.5	6.8	**UC**
F3P16_RS13480	0.0	0.0	UC	0.0	0.0	0.0	2.0	9.1	**UC**
F3P16_RS13485	0.0	0.0	2.1	0.0	0.0	0.0	0.3	2.1	**3.0**
F3P16_RS13490	0.0	0.0	1.1	0.0	0.0	0.0	4.0	1.1	**1.0**
F3P16_RS13495	0.0	0.0	UC	0.0	0.0	0.0	UC	UC	**UC**
F3P16_RS13500	0.0	0.0	UC	0.0	0.0	0.0	UC	UC	**UC**
F3P16_RS13505	0.0	0.0	UC	0.0	0.0	0.0	UC	UC	**UC**
F3P16_RS13510	0.0	0.0	UC	0.0	0.0	0.1	1.0	3.4	**UC**
F3P16_RS13515	0.0	0.0	UC	0.0	0.0	0.0	UC	UC	**UC**
F3P16_RS13520	0.0	0.0	UC	0.0	0.0	0.0	UC	UC	**UC**
F3P16_RS13525	0.0	0.0	0.0	0.1	0.0	0.0	0.3	0.2	**1.5**
F3P16_RS13530	0.0	0.0	0.7	0.0	0.0	0.1	1.1	2.0	**0.7**
F3P16_RS13535	0.0	0.0	UC	0.0	0.0	0.0	UC	UC	**UC**
F3P16_RS13540	0.0	0.0	UC	0.0	0.0	0.0	UC	UC	**UC**
F3P16_RS13545	0.1	0.0	0.4	0.0	0.0	0.0	0.4	0.7	**0.8**

F3P16_RS13550	0.0	0.0	UC	0.0	0.0	0.0	UC	UC	**UC**
F3P16_RS13555	0.0	0.0	UC	0.0	0.0	0.0	UC	UC	**UC**
F3P16_RS13560	0.0	0.0	UC	0.0	0.0	0.0	UC	UC	**UC**
F3P16_RS13565	0.0	0.0	UC	0.0	0.0	0.0	UC	UC	**UC**
F3P16_RS13570	0.0	0.0	UC	0.0	0.0	0.0	UC	UC	**UC**
F3P16_RS13575	0.0	0.0	UC	0.0	0.0	0.0	UC	UC	**UC**
F3P16_RS13580	0.0	0.0	UC	0.0	0.0	0.0	UC	UC	**UC**
F3P16_RS13585	0.0	0.0	UC	0.0	0.0	0.0	UC	UC	**UC**
F3P16_RS18915	0.0	0.0	UC	0.0	0.0	0.0	UC	UC	**UC**
F3P16_RS13590	0.0	0.0	UC	0.0	0.0	0.0	UC	UC	**UC**
F3P16_RS13595	0.0	0.0	UC	0.1	0.0	0.1	0.4	2.1	**UC**
F3P16_RS13600	0.0	0.0	UC	0.0	0.0	0.2	UC	UC	**UC**
F3P16_RS13605	0.0	0.0	2.1	0.0	0.0	0.1	4.5	8.6	**0.5**
F3P16_RS13610	0.0	0.0	0.5	0.0	0.0	0.1	2.1	4.1	**1.7**
F3P16_RS13615	0.0	0.0	0.8	0.0	0.0	0.1	3.2	4.9	**0.5**
ata]	0.0	0.1	2.7	0.0	0.1	0.3	2.7	10.7	**0.7**
lon]	5.5	14.3	2.6	14.1	13.8	30.1	1.0	2.1	**2.6**
rlmH	0.5	0.8	1.7	0.5	0.7	0.9	1.5	1.9	**1.0**
F3P16_RS13635	0.1	0.2	1.5	0.2	0.3	0.5	1.8	3.0	**1.3**
F3P16_RS13640	3.3	60.4	18.4	28.9	42.8	37.9	1.5	1.3	**8.8**
gdhA	0.2	0.3	1.3	0.4	0.5	0.5	1.4	1.2	**1.5**
F3P16_RS13650	0.1	0.1	1.6	0.2	0.3	0.3	1.8	2.1	**1.8**
nth]	0.1	0.2	1.5	0.2	0.5	0.5	2.4	2.5	**1.4**
F3P16_RS13660	0.2	0.3	1.4	0.3	0.9	0.8	2.5	2.4	**1.9**
adk]	0.6	0.9	1.5	0.9	1.4	2.0	1.5	2.1	**1.5**
F3P16_RS13670	0.4	0.5	1.3	0.5	0.7	0.9	1.6	1.8	**1.2**
F3P16_RS13675	0.0	0.1	1.8	0.1	0.1	0.2	1.8	2.6	**1.4**
mrdA	1.2	1.6	1.3	1.5	2.7	2.5	1.8	1.6	**1.3**
rsmD	0.3	0.6	1.7	0.6	0.8	0.9	1.5	1.6	**1.6**
F3P16_RS13690	0.5	0.6	1.3	0.5	0.8	0.7	1.6	1.5	**1.1**

ureG	0.5	0.6	1.2	0.5	0.9	0.6	1.9	1.3	**1.0**
F3P16_RS13700	0.5	0.7	1.4	0.4	0.8	0.5	1.9	1.2	**0.8**
ureE	0.1	0.2	1.7	0.3	0.4	0.3	1.6	1.3	**2.0**
ureC	0.2	0.2	1.1	0.1	0.2	0.2	2.0	2.7	**0.5**
F3P16_RS19100	0.3	0.3	1.2	0.1	0.1	0.2	1.2	2.3	**0.4**
ureA	0.1	0.1	1.1	0.0	0.1	0.1	3.3	2.4	**0.4**
F3P16_RS13725	0.1	0.1	1.0	0.0	0.0	0.1	1.9	6.5	**0.3**
F3P16_RS13730	0.0	0.0	0.0	0.0	0.0	0.1	UC	UC	**0.0**
F3P16_RS13735	0.3	1.5	5.2	2.1	3.0	2.8	1.4	1.3	**7.6**
F3P16_RS13740	0.8	2.0	2.4	1.3	1.3	1.3	1.0	1.0	**1.6**
F3P16_RS13745	1.0	1.3	1.3	1.6	2.0	2.4	1.2	1.5	**1.6**
F3P16_RS13750	0.5	0.9	1.8	0.6	1.0	1.3	1.6	2.1	**1.2**
F3P16_RS13760	0.6	1.8	2.8	1.9	2.4	4.0	1.3	2.1	**2.9**
F3P16_RS13765	0.2	0.1	0.9	0.1	0.3	0.2	2.8	1.6	**0.7**
F3P16_RS13770	0.0	0.0	2.1	0.0	0.0	0.1	0.6	2.4	**5.0**
cysN	1.4	2.7	1.9	3.0	3.0	4.1	1.0	1.4	**2.1**
cysD	1.5	2.8	1.9	2.4	2.9	3.9	1.2	1.6	**1.6**
F3P16_RS13785	1.0	1.0	1.1	0.8	0.9	1.6	1.0	1.8	**0.9**
F3P16_RS13790	0.3	1.0	3.2	0.9	1.1	1.4	1.2	1.7	**2.9**
lysS	1.9	2.4	1.2	3.1	4.2	4.2	1.4	1.4	**1.6**
F3P16_RS13800	0.0	0.0	6.3	0.0	0.1	0.0	2.5	1.5	**4.5**
F3P16_RS13805	0.0	0.0	UC	0.0	0.0	0.0	1.0	0.0	**UC**
F3P16_RS13810	0.2	0.4	1.5	0.6	0.5	0.8	1.0	1.5	**2.3**
F3P16_RS13815	0.8	1.5	1.9	1.1	1.8	2.1	1.6	1.8	**1.4**
F3P16_RS13820	0.5	0.9	1.8	0.7	1.3	1.3	1.8	1.8	**1.5**
F3P16_RS13825	0.3	0.7	2.2	0.8	1.2	1.7	1.5	2.1	**2.5**
F3P16_RS13830	0.0	0.0	UC	0.0	0.0	0.0	UC	UC	**UC**
F3P16_RS13835	0.0	0.0	UC	0.0	0.0	0.0	UC	UC	**UC**
ppk1	0.5	1.1	2.3	0.9	1.4	1.1	1.6	1.2	**1.9**
mtgA	0.1	0.2	2.5	0.2	0.3	0.5	1.7	2.9	**1.9**

F3P16_RS13850	0.0	0.1	2.5	0.1	0.1	0.1	1.4	1.6	**1.8**
F3P16_RS13855	0.0	0.1	2.8	0.1	0.2	0.1	1.7	1.4	**2.1**
F3P16_RS13860	0.1	0.1	1.3	0.1	0.1	0.1	1.5	2.0	**0.8**
F3P16_RS13865	0.0	0.1	1.4	0.1	0.1	0.3	1.0	3.1	**1.9**
F3P16_RS13870	0.3	0.3	0.9	0.3	0.3	0.8	0.8	2.4	**1.1**
F3P16_RS13875	0.3	0.4	1.7	0.4	0.7	0.7	1.6	1.8	**1.6**
F3P16_RS13880	0.2	0.3	1.9	0.3	0.4	0.6	1.5	2.1	**1.7**
F3P16_RS13885	0.1	0.0	0.7	0.0	0.0	0.1	2.8	9.8	**0.3**
nfuA	0.7	0.9	1.3	1.1	1.7	3.5	1.6	3.2	**1.6**
F3P16_RS13895	0.0	0.0	1.1	0.0	0.0	0.1	3.5	10.3	**2.0**
F3P16_RS13900	0.0	0.0	7.9	0.0	0.0	0.0	1.5	4.6	**5.0**
F3P16_RS13905	0.0	0.0	1.5	0.0	0.0	0.1	0.9	1.6	**2.2**
F3P16_RS13910	0.0	0.0	1.1	0.2	0.1	0.2	0.4	1.0	**9.4**
F3P16_RS13915	0.3	0.5	1.7	0.5	0.5	0.8	1.0	1.6	**1.9**
F3P16_RS13920	0.0	0.1	3.4	0.0	0.1	0.2	2.7	4.7	**0.9**
F3P16_RS13925	0.7	1.2	1.9	1.2	1.9	2.3	1.5	1.8	**1.9**
metH	0.9	1.3	1.5	1.2	2.0	1.6	1.7	1.4	**1.3**
F3P16_RS13935	0.0	0.1	1.6	0.0	0.1	0.1	2.9	2.5	**0.7**
F3P16_RS13940	0.1	0.3	3.7	0.2	0.2	0.3	1.3	1.6	**2.4**
F3P16_RS13945	0.1	0.1	1.2	0.0	0.0	0.2	1.0	3.3	**0.9**
F3P16_RS13950	0.0	0.1	5.7	0.1	0.0	0.1	0.3	2.3	**3.2**
F3P16_RS13955	0.0	0.0	5.3	0.0	0.0	0.1	0.5	2.0	**6.5**
F3P16_RS13960	0.0	0.0	0.5	0.0	0.0	0.1	0.0	3.7	**1.0**
F3P16_RS13970	0.2	0.7	3.7	0.4	0.5	0.6	1.4	1.7	**2.0**
F3P16_RS13975	0.1	0.1	1.3	0.1	0.1	0.1	0.9	1.0	**1.8**
F3P16_RS13980	0.1	0.1	0.9	0.1	0.2	0.3	1.4	2.7	**0.8**
F3P16_RS13985	0.5	0.7	1.5	0.6	0.9	1.6	1.6	2.9	**1.2**
def]	0.0	0.0	1.6	0.0	0.0	0.1	1.3	5.1	**2.0**
F3P16_RS13995	0.1	0.2	3.0	0.1	0.1	0.1	1.0	1.7	**1.1**
F3P16_RS14000	0.0	0.0	0.7	0.0	0.0	0.2	0.7	14.1	**0.5**

F3P16_RS14005	0.0	0.1	2.3	0.1	0.1	0.1	1.6	1.1	**1.3**
F3P16_RS14010	0.0	0.1	2.4	0.1	0.1	0.1	1.4	1.1	**2.0**
F3P16_RS14015	0.0	0.1	2.5	0.1	0.1	0.1	1.6	0.8	**3.1**
F3P16_RS14020	0.0	0.1	3.6	0.1	0.1	0.1	0.9	0.7	**3.4**
F3P16_RS14025	0.1	0.1	1.9	0.1	0.1	0.1	1.0	1.0	**1.4**
F3P16_RS14030	0.1	0.2	1.5	0.1	0.2	0.1	1.2	0.9	**1.0**
F3P16_RS14035	0.0	0.1	2.9	0.0	0.1	0.1	3.5	2.7	**0.8**
F3P16_RS14040	0.0	0.0	2.3	0.0	0.0	0.0	0.4	1.4	**3.4**
F3P16_RS14045	0.0	0.0	3.7	0.0	0.0	0.1	0.3	2.1	**2.6**
F3P16_RS14050	0.0	0.1	6.1	0.0	0.0	0.1	0.6	2.1	**5.0**
F3P16_RS14055	0.0	0.0	1.6	0.0	0.0	0.2	0.9	4.6	**1.8**
F3P16_RS14060	0.0	0.0	UC	0.0	0.0	0.0	UC	UC	**UC**
F3P16_RS14065	0.0	0.0	UC	0.0	0.0	0.4	UC	UC	**UC**
F3P16_RS14070	0.0	0.1	3.2	0.1	0.1	0.2	1.2	2.5	**2.8**
F3P16_RS14075	0.0	0.0	0.0	0.0	0.0	0.1	UC	UC	**0.0**
pnuC	0.0	0.0	3.5	0.0	0.0	0.0	0.3	1.4	**4.0**
F3P16_RS14085	0.0	0.1	6.9	0.1	0.0	0.3	0.4	3.4	**4.8**
pgaD	0.4	0.4	0.9	0.5	0.7	0.8	1.4	1.6	**1.2**
pgaC	0.7	1.1	1.6	1.3	1.5	1.7	1.2	1.4	**1.8**
pgaB	0.1	0.4	3.0	0.3	0.5	0.5	1.5	1.7	**2.4**
F3P16_RS14105	0.1	0.1	2.7	0.3	0.3	0.5	1.0	1.5	**5.6**
F3P16_RS14110	0.1	0.1	1.4	0.1	0.1	0.1	1.4	1.6	**1.1**
dctA	0.0	0.0	1.8	0.0	0.0	0.1	2.3	7.3	**1.1**
F3P16_RS14120	0.1	0.1	0.9	0.1	0.3	0.4	2.6	3.9	**0.9**
F3P16_RS14125	0.4	0.4	1.1	2.1	3.5	3.8	1.7	1.8	**5.9**
F3P16_RS14130	0.1	0.3	1.8	0.3	0.4	0.6	1.3	2.2	**2.0**
F3P16_RS14135	0.2	0.7	3.0	1.2	1.8	2.3	1.5	1.9	**5.1**
F3P16_RS14140	3.1	9.5	3.0	11.6	15.5	11.9	1.3	1.0	**3.7**
F3P16_RS14145	1.4	2.5	1.7	3.8	2.9	3.4	0.8	0.9	**2.7**
betI	10.5	21.1	2.0	25.5	25.2	22.2	1.0	0.9	**2.4**

betB	13.3	31.1	2.3	31.9	36.8	33.2	1.2	1.0	**2.4**
betA	9.9	24.3	2.4	26.3	30.9	30.0	1.2	1.1	**2.7**
mqo]	3.1	2.5	0.8	2.8	5.4	8.1	1.9	2.9	**0.9**
F3P16_RS14170	0.1	0.0	0.5	0.0	0.1	0.1	1.9	2.1	**0.7**
F3P16_RS14175	0.2	0.1	0.6	0.1	0.2	0.3	2.9	5.5	**0.2**
F3P16_RS14180	0.3	0.3	0.8	0.1	0.3	0.5	2.6	3.7	**0.4**
F3P16_RS14185	0.1	0.1	1.2	0.1	0.1	0.1	1.8	1.9	**1.0**
F3P16_RS14190	0.0	0.0	1.6	0.0	0.0	0.0	1.7	3.4	**1.5**
F3P16_RS14195	0.0	0.0	UC	0.0	0.0	0.0	0.5	0.0	**UC**
F3P16_RS14200	0.0	0.0	UC	0.0	0.0	0.1	1.3	2.7	**UC**
F3P16_RS14205	0.0	0.0	1.4	0.0	0.0	0.0	0.3	0.8	**2.5**
F3P16_RS14210	0.0	0.0	3.2	0.0	0.0	0.0	3.0	8.6	**0.5**
F3P16_RS14215	0.0	0.0	1.2	0.0	0.0	0.0	0.0	12.5	**0.2**
F3P16_RS14220	0.0	0.0	1.1	0.0	0.0	0.0	0.3	3.4	**2.0**
F3P16_RS14225	0.0	0.0	1.0	0.0	0.0	0.1	0.4	9.3	**0.6**
F3P16_RS14230	0.0	0.0	UC	0.0	0.0	0.0	0.0	1.6	**UC**
F3P16_RS14235	0.0	0.0	4.2	0.0	0.0	0.0	UC	UC	**0.0**
F3P16_RS14240	0.1	0.1	1.5	0.1	0.1	0.2	1.2	3.0	**1.1**
F3P16_RS14245	0.0	0.0	UC	0.0	0.0	0.0	UC	UC	**UC**
F3P16_RS14250	0.0	0.0	4.5	0.0	0.0	0.1	0.9	4.5	**3.2**
F3P16_RS14255	0.0	0.0	UC	0.0	0.0	0.0	0.7	4.4	**UC**
F3P16_RS14260	0.0	0.2	5.5	0.0	0.0	0.4	0.3	12.4	**1.0**
F3P16_RS14265	0.0	0.0	UC	0.0	0.0	0.0	UC	UC	**UC**
F3P16_RS14270	0.0	0.0	UC	0.0	0.0	0.0	UC	UC	**UC**
F3P16_RS14275	0.0	0.0	UC	0.0	0.0	0.0	UC	UC	**UC**
F3P16_RS14280	0.0	0.0	UC	0.0	0.0	0.0	UC	UC	**UC**
F3P16_RS14285	0.0	0.0	UC	0.0	0.0	0.0	UC	UC	**UC**
F3P16_RS14290	0.0	0.0	UC	0.0	0.0	0.0	UC	UC	**UC**
F3P16_RS14295	0.0	0.0	UC	0.0	0.0	0.0	UC	UC	**UC**
F3P16_RS14300	0.0	0.0	UC	0.0	0.0	0.0	UC	UC	**UC**

ssrA	36.7	44.2	1.2	65.3	51.4	130.3	0.8	2.0	**1.8**
ggt]	0.6	1.8	3.1	0.8	0.9	1.2	1.1	1.4	**1.5**
F3P16_RS14315	0.3	0.9	3.2	0.8	1.0	1.2	1.2	1.5	**2.7**
F3P16_RS14320	0.5	1.6	3.4	0.9	1.2	1.5	1.4	1.7	**1.8**
F3P16_RS14325	0.2	0.2	1.3	0.2	0.1	0.4	0.6	2.0	**1.2**
hemB	1.6	3.0	1.9	2.7	3.3	4.2	1.2	1.6	**1.7**
F3P16_RS14335	1.0	2.0	2.0	1.5	2.4	2.5	1.5	1.6	**1.6**
F3P16_RS14340	0.2	0.3	2.2	0.1	0.3	0.3	2.2	2.3	**1.0**
F3P16_RS14345	0.0	0.0	2.1	0.0	0.1	0.1	5.4	6.8	**1.0**
F3P16_RS14350	0.1	0.2	3.0	0.2	0.2	0.3	0.7	1.3	**3.6**
F3P16_RS14355	0.0	0.1	4.9	0.1	0.1	0.1	0.5	1.5	**9.3**
F3P16_RS14360	0.1	0.2	1.2	0.1	0.2	0.3	2.3	3.4	**0.7**
F3P16_RS14365	0.1	0.1	1.9	0.1	0.2	0.2	1.6	1.8	**2.1**
F3P16_RS14370	0.1	0.1	2.7	0.1	0.2	0.3	1.2	2.0	**2.7**
F3P16_RS14375	0.0	0.0	2.9	0.0	0.1	0.1	2.1	3.4	**2.0**
F3P16_RS14380	0.0	0.1	1.3	0.0	0.1	0.1	1.5	1.8	**1.1**
fur]	0.1	0.2	1.6	0.2	0.3	0.7	1.2	2.9	**2.5**
bamE	0.9	2.2	2.5	1.8	2.3	2.9	1.3	1.6	**2.0**
F3P16_RS14395	0.2	0.4	1.7	0.3	0.6	0.9	1.6	2.5	**1.5**
F3P16_RS14400	0.5	0.8	1.8	0.9	1.0	1.7	1.2	2.0	**1.9**
F3P16_RS14410	0.6	0.3	0.5	0.4	0.4	1.0	1.0	2.7	**0.6**
F3P16_RS14415	0.0	0.0	4.0	0.0	0.0	0.1	0.9	3.1	**2.6**
F3P16_RS14420	0.0	0.0	5.3	0.0	0.1	0.1	2.0	2.4	**7.4**
argB	0.7	1.9	2.5	1.7	2.1	2.6	1.2	1.5	**2.4**
F3P16_RS14430	1.4	4.3	3.2	3.1	3.7	4.2	1.2	1.3	**2.3**
dut]	0.2	0.4	2.4	0.3	0.9	0.9	2.7	2.6	**1.9**
rep]	0.3	1.1	3.8	1.1	1.7	1.5	1.6	1.4	**3.9**
F3P16_RS14445	0.7	4.7	6.4	3.3	4.3	8.1	1.3	2.4	**4.6**
F3P16_RS14450	0.0	0.1	2.1	0.1	0.1	0.4	1.1	4.0	**3.1**
F3P16_RS14455	0.0	0.1	2.5	0.1	0.2	0.3	1.4	2.3	**3.2**

minC	0.8	1.8	2.3	2.0	3.5	4.0	1.8	2.0	**2.6**
minD	3.7	11.3	3.0	17.0	21.8	25.7	1.3	1.5	**4.6**
minE	0.8	3.1	4.2	4.5	7.0	8.7	1.6	1.9	**6.0**
F3P16_RS14475	0.2	0.8	3.6	1.3	2.1	2.3	1.6	1.7	**5.8**
rhtC	0.0	0.0	0.4	0.0	0.1	0.1	2.0	2.3	**0.9**
F3P16_RS14485	2.1	4.8	2.3	5.6	7.4	5.8	1.3	1.0	**2.6**
trxB	0.3	0.8	2.6	0.7	0.9	1.3	1.2	1.8	**2.3**
aat]	0.1	0.3	2.0	0.2	0.5	0.3	2.0	1.2	**1.8**
F3P16_RS14500	0.1	0.2	2.8	0.1	0.2	0.2	1.3	2.0	**1.4**
rimI	0.0	0.0	1.6	0.1	0.1	0.1	1.2	0.9	**2.5**
F3P16_RS14510	0.0	0.0	1.3	0.0	0.0	0.0	UC	UC	**0.0**
F3P16_RS14515	0.1	0.1	1.4	0.0	0.1	0.1	2.7	3.3	**0.7**
tuf]	59.9	95.9	1.6	104.9	189.9	305.6	1.8	2.9	**1.8**
fusA	48.3	100.9	2.1	104.5	211.6	238.0	2.0	2.3	**2.2**
rpsG	37.4	87.2	2.3	83.7	195.7	212.5	2.3	2.5	**2.2**
rpsL	17.2	31.5	1.8	30.8	66.6	77.7	2.2	2.5	**1.8**
F3P16_RS14540	0.3	1.4	4.3	1.4	1.7	1.7	1.2	1.2	**4.4**
F3P16_RS14545	2.8	3.2	1.1	3.6	4.7	9.0	1.3	2.5	**1.3**
hemJ	0.5	0.6	1.4	0.8	1.0	2.1	1.2	2.7	**1.6**
F3P16_RS14555	0.1	0.1	1.0	0.2	0.2	0.2	1.2	1.3	**1.3**
F3P16_RS14560	0.0	0.0	0.3	0.0	0.0	0.0	2.0	1.1	**1.5**
F3P16_RS14565	0.7	2.0	3.0	2.0	2.9	3.4	1.5	1.7	**2.9**
F3P16_RS14570	0.1	0.2	3.8	0.2	0.4	0.5	2.1	2.2	**3.6**
F3P16_RS14575	0.2	0.4	1.7	0.6	0.7	1.0	1.2	1.7	**2.4**
F3P16_RS14580	0.2	0.7	4.1	0.6	0.5	0.4	0.8	0.6	**4.0**
F3P16_RS14585	0.4	1.3	2.9	1.4	1.3	0.5	0.9	0.4	**3.2**
F3P16_RS14590	0.3	1.0	3.3	1.0	0.7	0.4	0.7	0.4	**3.3**
F3P16_RS14595	0.7	2.0	2.9	2.0	1.6	0.6	0.8	0.3	**2.9**
F3P16_RS14600	0.9	2.3	2.5	2.0	1.6	0.6	0.8	0.3	**2.2**
F3P16_RS14605	0.9	2.0	2.2	1.9	1.0	0.5	0.5	0.3	**2.1**

F3P16_RS14610	0.1	0.2	3.7	0.1	0.1	0.2	1.1	1.7	**1.8**
F3P16_RS14615	0.0	0.0	2.5	0.0	0.0	0.0	2.5	1.8	**1.2**
F3P16_RS14620	0.2	0.4	1.7	0.6	0.7	1.0	1.2	1.7	**2.4**
rnpB	3.4	3.0	0.9	4.5	3.8	12.4	0.8	2.7	**1.3**
F3P16_RS14630	1.2	1.9	1.6	1.5	2.1	3.4	1.4	2.2	**1.2**
coaD	0.4	0.6	1.6	0.5	0.8	1.0	1.5	1.8	**1.4**
smpB	0.1	0.1	2.6	0.2	0.2	0.7	1.0	3.0	**4.1**
F3P16_RS14645	0.1	0.2	2.8	0.2	0.3	0.3	1.2	1.1	**3.5**
pgeF	1.1	1.9	1.8	1.3	2.1	2.7	1.6	2.0	**1.3**
rluD	0.7	1.1	1.5	1.2	1.6	1.9	1.3	1.6	**1.6**
F3P16_RS14660	1.3	4.6	3.5	4.8	6.6	9.5	1.4	2.0	**3.7**
F3P16_RS14665	0.3	0.6	2.2	0.6	1.0	0.8	1.6	1.3	**2.3**
hemA	0.6	1.0	1.6	1.2	1.7	2.0	1.4	1.7	**1.9**
F3P16_RS14675	0.1	0.5	4.0	0.5	0.7	0.8	1.5	1.9	**3.8**
lolB	0.1	4.3	38.4	2.6	3.3	3.0	1.2	1.1	**23.3**
ispE	0.4	4.6	11.1	3.7	4.9	5.0	1.3	1.4	**8.9**
F3P16_RS14690	9.8	21.8	2.2	11.9	19.3	34.4	1.6	2.9	**1.2**
F3P16_RS14695	9.8	21.8	2.2	11.9	19.3	34.4	1.6	2.9	**1.2**
F3P16_RS14700	9.8	21.8	2.2	11.9	19.3	34.4	1.6	2.9	**1.2**
F3P16_RS14705	9.8	21.8	2.2	11.9	19.3	34.4	1.6	2.9	**1.2**
F3P16_RS14710	6.7	10.7	1.6	11.8	23.3	34.9	2.0	3.0	**1.8**
rplY	6.1	11.5	1.9	15.6	31.3	27.2	2.0	1.7	**2.6**
pth]	7.6	12.1	1.6	14.6	31.6	25.4	2.2	1.7	**1.9**
F3P16_RS14725	1.3	2.0	1.5	2.2	4.4	4.0	2.0	1.8	**1.7**
F3P16_RS14730	0.0	0.1	2.2	0.1	0.1	0.1	1.2	1.2	**2.4**
F3P16_RS14735	0.0	0.1	2.6	0.1	0.1	0.1	2.1	1.5	**2.2**
ribB	1.6	2.3	1.5	1.7	1.9	1.2	1.1	0.7	**1.1**
F3P16_RS14745	0.3	0.7	2.2	0.7	0.8	0.8	1.2	1.1	**2.1**
F3P16_RS14750	0.3	0.6	1.9	0.3	0.9	1.1	2.5	3.1	**1.2**
lysM	0.1	0.4	3.9	0.5	1.2	1.8	2.3	3.3	**5.3**

acpP	0.6	0.5	0.9	1.4	3.0	4.3	2.1	3.1	**2.5**
fabG	9.0	11.7	1.3	12.4	19.5	31.6	1.6	2.6	**1.4**
fabD	6.1	8.8	1.4	8.6	13.3	16.9	1.5	2.0	**1.4**
rpmF	2.9	4.3	1.5	6.3	9.7	19.7	1.5	3.1	**2.2**
F3P16_RS14780	8.1	10.6	1.3	17.3	24.9	54.6	1.4	3.2	**2.1**
F3P16_RS14785	6.4	7.6	1.2	8.9	10.6	13.9	1.2	1.6	**1.4**
F3P16_RS14790	0.7	1.0	1.5	0.8	1.0	2.0	1.3	2.7	**1.1**
F3P16_RS14795	0.2	0.3	1.7	0.2	0.4	0.5	2.2	3.2	**1.1**
scpB	0.3	0.4	1.1	0.3	0.5	0.9	1.9	3.2	**0.8**
rluB	0.7	0.7	0.9	1.0	1.2	2.3	1.3	2.3	**1.3**
bioD	0.2	0.4	1.7	0.9	2.1	1.9	2.3	2.1	**3.6**
bioC	0.3	0.4	1.4	1.2	3.1	2.5	2.6	2.0	**4.1**
F3P16_RS14820	0.4	0.5	1.3	1.5	3.1	2.4	2.1	1.6	**3.6**
bioA	0.7	1.0	1.6	2.5	4.2	4.1	1.7	1.6	**3.9**
F3P16_RS14830	0.0	0.1	21.1	0.1	0.1	0.1	1.4	1.6	**12.4**
otsB	0.0	0.0	12.7	0.0	0.0	0.0	1.0	0.2	**19.9**
F3P16_RS14840	0.1	0.7	6.8	0.6	0.6	0.3	1.0	0.5	**5.6**
F3P16_RS14845	0.4	1.5	3.6	1.3	2.1	1.0	1.6	0.7	**3.3**
bfr]	0.5	1.5	2.9	2.2	1.7	1.8	0.8	0.8	**4.2**
ligA	0.3	0.6	1.9	0.6	0.9	1.1	1.4	1.7	**2.0**
F3P16_RS14860	0.5	1.4	2.7	1.4	1.4	2.3	1.0	1.7	**2.7**
smc]	0.1	0.3	2.7	0.3	0.4	0.6	1.4	2.3	**2.4**
F3P16_RS14870	0.0	0.0	4.8	0.1	0.0	0.1	0.6	2.2	**10.9**
F3P16_RS14875	0.1	0.2	1.8	0.2	0.2	0.4	1.2	2.3	**2.1**
F3P16_RS14880	0.2	0.6	3.4	0.4	0.6	1.2	1.7	3.1	**2.2**
F3P16_RS14885	0.2	0.4	2.7	0.5	0.5	1.2	1.0	2.5	**2.9**
ffh]	9.0	16.0	1.8	15.7	20.5	28.3	1.3	1.8	**1.7**
ccsA	0.1	0.1	0.7	0.1	0.1	0.2	1.1	1.7	**1.3**
F3P16_RS14900	0.1	0.1	1.5	0.1	0.1	0.1	1.1	1.5	**1.5**
F3P16_RS14905	0.0	0.0	8.4	0.0	0.0	0.0	0.9	1.6	**6.9**

F3P16_RS14910	0.1	0.2	3.9	0.1	0.2	0.2	1.5	1.4	**2.7**
F3P16_RS14915	0.0	0.1	2.0	0.1	0.2	0.1	1.7	1.0	**2.6**
F3P16_RS14920	0.0	0.0	2.3	0.0	0.0	0.1	1.1	1.8	**2.5**
F3P16_RS14925	0.0	0.0	1.6	0.0	0.0	0.0	0.9	0.4	**4.0**
dcd]	0.3	0.6	1.9	0.5	0.8	1.3	1.5	2.6	**1.6**
F3P16_RS14940	0.0	0.1	3.6	0.1	0.1	0.1	1.3	1.2	**3.0**
F3P16_RS14945	0.0	0.0	3.2	0.0	0.1	0.0	3.3	1.9	**3.0**
F3P16_RS14950	0.0	0.1	7.7	0.1	0.1	0.2	0.9	3.2	**9.4**
apbC	0.8	2.5	3.3	2.3	3.2	2.3	1.4	1.0	**2.9**
F3P16_RS14965	0.5	3.3	7.1	4.6	5.0	3.4	1.1	0.7	**9.9**
metG	1.2	2.3	1.9	3.0	3.9	4.6	1.3	1.5	**2.4**
F3P16_RS14975	0.0	0.0	UC	0.0	0.0	0.1	UC	UC	**UC**
F3P16_RS14980	0.0	0.1	10.6	0.0	0.1	0.2	2.7	5.7	**5.0**
F3P16_RS14985	0.0	0.1	13.4	0.1	0.1	0.2	0.5	2.2	**11.9**
F3P16_RS14990	0.1	0.5	6.0	0.4	0.4	0.7	0.9	1.6	**4.7**
F3P16_RS14995	0.1	0.5	3.4	0.3	0.3	0.5	1.1	1.9	**1.9**
F3P16_RS15000	0.0	0.2	8.4	0.1	0.1	0.2	1.1	2.8	**3.5**
F3P16_RS15005	0.2	0.2	1.3	0.2	0.4	0.5	1.8	2.4	**1.2**
F3P16_RS15010	0.0	0.2	5.8	0.2	0.5	0.4	2.4	2.1	**5.2**
F3P16_RS15015	0.9	2.0	2.3	1.6	1.9	1.2	1.2	0.8	**1.9**
F3P16_RS15020	0.1	0.1	1.6	0.3	0.3	1.0	0.8	3.2	**4.4**
F3P16_RS15025	0.4	0.6	1.7	0.4	1.1	1.5	2.7	3.6	**1.1**
F3P16_RS15030	0.1	0.2	2.5	0.2	0.5	0.7	2.5	3.5	**2.1**
gigC	1.0	1.3	1.3	1.1	1.6	2.3	1.4	2.1	**1.1**
F3P16_RS15040	0.0	0.0	UC	0.0	0.0	0.0	UC	UC	**UC**
F3P16_RS15045	0.7	1.3	1.8	1.8	2.5	3.4	1.4	1.8	**2.6**
upp]	1.1	1.4	1.3	1.6	2.2	2.5	1.3	1.5	**1.5**
nuoN	2.1	3.0	1.4	3.9	6.2	9.3	1.6	2.4	**1.8**
nuoM	7.0	9.6	1.4	10.7	19.8	19.7	1.8	1.8	**1.5**
nuoL	8.7	14.2	1.6	15.7	28.4	26.2	1.8	1.7	**1.8**

nuoK	11.4	13.9	1.2	15.0	23.2	25.3	1.5	1.7	**1.3**
nuoJ	14.6	17.2	1.2	15.7	28.1	28.9	1.8	1.8	**1.1**
nuoI	8.2	9.8	1.2	9.5	15.6	16.6	1.6	1.8	**1.2**
nuoH	7.0	10.1	1.4	9.7	17.1	17.1	1.8	1.8	**1.4**
nuoG	5.8	8.2	1.4	7.2	12.5	15.3	1.7	2.1	**1.2**
nuoF	12.7	18.2	1.4	16.3	26.7	32.7	1.6	2.0	**1.3**
nuoE	7.5	14.6	1.9	12.1	24.1	24.2	2.0	2.0	**1.6**
nuoC	12.6	18.7	1.5	15.3	28.7	33.8	1.9	2.2	**1.2**
F3P16_RS15110	8.5	15.1	1.8	11.7	22.5	26.5	1.9	2.3	**1.4**
ndhC	6.0	8.8	1.5	6.8	11.8	15.0	1.7	2.2	**1.1**
F3P16_RS15120	0.0	0.0	0.9	0.0	0.1	0.1	2.9	3.6	**0.6**
F3P16_RS15125	0.1	0.6	4.3	0.5	0.8	0.7	1.4	1.4	**3.7**
bfmS	1.4	3.7	2.7	3.4	4.6	10.7	1.3	3.1	**2.5**
bfmR	1.0	2.8	3.0	2.1	2.9	11.7	1.4	5.5	**2.2**
F3P16_RS15140	0.2	0.3	2.1	0.2	0.2	0.5	1.1	2.8	**1.2**
F3P16_RS15145	3.7	5.3	1.4	6.0	11.0	15.3	1.8	2.5	**1.6**
F3P16_RS15150	0.1	0.3	1.8	0.4	1.2	1.4	3.5	4.0	**2.4**
F3P16_RS15155	5.9	9.2	1.6	9.9	17.0	22.9	1.7	2.3	**1.7**
F3P16_RS15160	0.0	0.0	UC	0.0	0.0	0.0	UC	UC	**UC**
F3P16_RS15165	0.0	0.0	UC	0.0	0.0	0.0	UC	UC	**UC**
F3P16_RS15170	0.0	0.0	UC	0.0	0.0	0.0	UC	UC	**UC**
F3P16_RS15175	0.0	0.0	UC	0.0	0.0	0.0	UC	UC	**UC**
F3P16_RS15180	0.0	0.0	UC	0.0	0.0	0.0	UC	UC	**UC**
F3P16_RS15185	0.0	0.0	UC	0.0	0.0	0.0	UC	UC	**UC**
F3P16_RS15190	0.0	0.0	UC	0.0	0.0	0.0	UC	UC	**UC**
F3P16_RS15195	0.0	0.0	UC	0.0	0.0	0.0	UC	UC	**UC**
F3P16_RS15200	0.0	0.0	UC	0.0	0.0	0.0	0.0	0.0	**UC**
F3P16_RS15205	0.0	0.0	UC	0.0	0.0	0.0	UC	UC	**UC**
F3P16_RS15210	0.0	0.0	UC	0.0	0.0	0.0	UC	UC	**UC**
F3P16_RS15215	0.1	0.3	6.5	0.4	0.3	0.6	0.8	1.6	**7.2**

F3P16_RS15220	0.0	0.0	UC	0.0	0.0	0.0	6.0	1.1	**UC**
F3P16_RS15225	0.0	0.0	UC	0.0	0.0	0.0	UC	UC	**UC**
hxsA	0.0	0.0	UC	0.0	0.0	0.0	UC	UC	**UC**
hxsC	0.0	0.0	UC	0.0	0.0	0.0	UC	UC	**UC**
hxsB	0.0	0.0	UC	0.0	0.0	0.0	UC	UC	**UC**
F3P16_RS15245	0.0	0.0	UC	0.0	0.0	0.0	UC	UC	**UC**
F3P16_RS15250	0.0	0.0	1.6	0.1	0.1	0.2	0.9	3.0	**4.5**
F3P16_RS15260	0.7	1.2	1.6	1.6	3.4	5.5	2.1	3.4	**2.3**
F3P16_RS15265	0.9	1.9	2.1	2.7	5.3	6.5	2.0	2.4	**3.0**
F3P16_RS15270	0.9	1.2	1.3	2.6	5.0	5.9	1.9	2.2	**2.9**
F3P16_RS15275	0.0	0.0	0.0	0.0	0.0	0.0	1.3	5.1	**2.0**
F3P16_RS15280	0.0	0.0	UC	0.0	0.0	0.0	UC	UC	**UC**
F3P16_RS15285	0.0	0.0	4.2	0.0	0.0	0.1	0.7	6.1	**3.0**
F3P16_RS15290	0.0	0.0	1.2	0.0	0.0	0.1	15.0	20.5	**0.2**
F3P16_RS15295	0.2	0.2	1.4	0.1	0.2	0.2	1.9	2.6	**0.5**
F3P16_RS15300	0.0	0.0	0.9	0.0	0.0	0.0	1.3	1.9	**3.3**
F3P16_RS15305	0.0	0.1	2.7	0.1	0.1	0.2	1.6	3.5	**2.0**
F3P16_RS15315	0.0	0.0	8.4	0.0	0.0	0.1	0.9	2.5	**7.9**
F3P16_RS15320	0.0	0.0	2.1	0.0	0.0	0.0	1.3	3.7	**0.8**
F3P16_RS15325	0.0	0.0	0.9	0.1	0.1	0.1	1.6	2.3	**1.2**
tcuA	0.0	0.1	2.9	0.1	0.1	0.2	1.0	3.1	**1.2**
tcuB	0.0	0.0	1.8	0.0	0.0	0.0	2.1	2.4	**0.8**
F3P16_RS15340	0.0	0.1	1.4	0.0	0.0	0.1	1.5	3.6	**0.7**
F3P16_RS15345	0.0	0.0	7.4	0.0	0.0	0.0	0.0	5.7	**2.0**
F3P16_RS15350	0.0	0.1	2.5	0.1	0.1	0.1	1.2	2.2	**1.2**
F3P16_RS15355	0.0	0.0	UC	0.0	0.0	0.0	1.9	1.4	**UC**
F3P16_RS15360	0.0	0.2	5.0	0.1	0.1	0.3	1.1	2.4	**3.3**
add]	0.0	0.3	6.3	0.2	0.1	0.4	0.8	2.6	**3.5**
F3P16_RS15370	0.7	0.6	0.9	0.9	1.2	2.7	1.3	2.9	**1.3**
F3P16_RS15375	1.3	1.2	0.9	1.7	2.2	4.3	1.3	2.5	**1.3**

F3P16_RS15380	2.3	2.5	1.1	3.0	2.7	8.0	0.9	2.6	**1.3**
F3P16_RS15385	0.7	0.6	0.9	0.9	1.2	2.7	1.3	2.9	**1.3**
nadA	0.4	0.7	1.5	0.7	0.8	1.2	1.2	1.7	**1.6**
argJ	1.4	1.9	1.3	1.5	2.0	3.8	1.3	2.5	**1.0**
F3P16_RS15400	0.0	0.1	3.0	0.1	0.1	0.3	0.9	3.4	**1.8**
F3P16_RS15405	0.0	0.1	10.0	0.1	0.1	0.1	1.0	1.2	**6.0**
F3P16_RS15410	0.0	0.1	4.2	0.1	0.1	0.2	1.2	2.6	**2.8**
F3P16_RS15415	0.2	0.2	1.1	0.1	0.2	0.2	1.5	1.4	**0.8**
F3P16_RS15420	0.7	0.7	1.0	0.5	0.8	0.6	1.5	1.2	**0.7**
F3P16_RS15425	2.6	3.9	1.5	2.6	3.8	3.9	1.5	1.5	**1.0**
F3P16_RS15430	0.3	0.6	1.7	0.7	0.9	1.0	1.4	1.5	**1.9**
rpe]	0.2	0.3	1.4	0.5	0.8	0.8	1.6	1.7	**2.1**
F3P16_RS15440	0.1	0.3	2.0	0.3	0.3	0.5	1.2	2.1	**1.8**
fghA	0.5	0.9	1.8	0.8	1.2	0.9	1.5	1.1	**1.5**
F3P16_RS15450	1.2	2.8	2.4	2.4	3.2	3.0	1.3	1.3	**2.1**
F3P16_RS15455	0.2	0.3	1.3	0.5	0.7	0.7	1.5	1.4	**2.0**
tsaB	0.4	0.7	1.8	0.7	1.0	1.2	1.5	1.8	**1.7**
F3P16_RS15465	0.1	0.3	2.9	0.4	0.4	0.5	0.8	1.1	**4.4**
F3P16_RS15470	0.1	0.3	2.8	0.4	0.3	0.7	0.8	1.7	**4.1**
F3P16_RS15475	0.1	0.2	2.3	0.3	0.4	0.4	1.2	1.3	**4.3**
F3P16_RS15480	0.0	0.0	UC	0.0	0.0	0.0	1.5	0.8	**UC**
filE	0.0	0.0	1.1	0.1	0.1	0.1	0.7	0.8	**2.1**
F3P16_RS15490	0.0	0.1	2.3	0.1	0.2	0.2	1.6	1.7	**2.9**
F3P16_RS15495	0.0	0.0	1.3	0.0	0.0	0.1	0.7	1.3	**2.6**
F3P16_RS15500	0.0	0.1	1.2	0.0	0.1	0.1	1.6	2.0	**0.8**
F3P16_RS15505	0.0	0.0	UC	0.0	0.0	0.0	11.0	6.8	**UC**
F3P16_RS15510	0.0	0.0	7.4	0.0	0.1	0.0	2.0	0.7	**9.6**
hisC	0.5	0.8	1.5	0.7	1.4	1.3	2.1	1.9	**1.3**
hisD	0.7	1.0	1.5	0.8	1.5	1.2	1.9	1.6	**1.2**
hisG	1.1	2.1	1.9	2.7	4.6	4.5	1.7	1.7	**2.4**

murA	2.9	6.1	2.1	6.2	9.3	9.2	1.5	1.5	**2.2**
ibaG	2.7	3.9	1.5	4.2	6.3	7.7	1.5	1.8	**1.5**
raiA	0.1	0.0	0.2	0.1	0.1	0.0	0.8	0.4	**1.3**
F3P16_RS15545	0.1	0.2	3.0	0.2	0.2	0.4	1.5	2.3	**2.5**
rrf]	1.4	1.5	1.0	2.6	2.6	5.2	1.0	2.0	**1.9**
F3P16_RS15555	13854.4	12854.9	0.9	12100.3	10115.1	11101.5	0.8	0.9	**0.9**
F3P16_RS15560	0.5	1.0	2.1	1.0	0.8	5.2	0.8	5.3	**2.1**
F3P16_RS15565	2.0	1.5	0.8	1.8	2.4	7.6	1.3	4.3	**0.9**
F3P16_RS15570	10000.7	10000.2	1.0	10000.2	9998.7	10000.6	1.0	1.0	**1.0**
F3P16_RS15575	0.7	1.1	1.6	1.1	1.4	1.8	1.2	1.6	**1.6**
gigA	0.4	0.6	1.5	0.6	0.7	1.2	1.1	1.9	**1.7**
gigB	0.3	0.5	1.5	0.6	0.7	1.6	1.2	2.7	**1.8**
F3P16_RS15590	0.6	0.8	1.3	0.7	1.3	2.0	1.9	2.9	**1.1**
F3P16_RS15595	0.2	0.2	1.3	0.3	0.4	0.6	1.5	2.2	**1.7**
F3P16_RS15600	0.0	0.0	0.5	0.0	0.1	0.1	8.0	16.5	**1.0**
F3P16_RS15605	0.0	0.1	UC	0.2	0.1	0.5	0.8	3.0	**UC**
gspE	0.5	0.9	1.8	0.6	0.8	1.0	1.4	1.7	**1.1**
F3P16_RS15615	0.5	0.9	1.8	0.6	0.7	1.3	1.2	2.1	**1.2**
F3P16_RS15620	0.2	0.6	2.5	0.6	1.0	1.1	1.6	1.8	**2.6**
F3P16_RS15625	0.2	0.7	4.3	0.7	0.9	0.7	1.4	1.1	**4.1**
polA	0.5	1.3	2.4	1.0	2.1	2.0	2.0	1.9	**1.9**
F3P16_RS15635	0.2	0.3	1.8	0.2	0.7	1.1	3.1	5.1	**1.2**
proC	0.6	1.0	1.6	0.8	1.2	2.0	1.4	2.4	**1.4**
tilS	0.7	0.9	1.3	0.8	1.2	1.5	1.4	1.9	**1.2**
F3P16_RS15650	8.7	10.4	1.2	8.6	10.0	15.9	1.2	1.8	**1.0**
ppx]	0.8	0.9	1.1	0.8	1.2	2.1	1.5	2.7	**0.9**
trxA	0.2	0.5	2.1	0.5	0.7	1.9	1.3	3.5	**2.5**
rho]	2.4	3.7	1.5	4.7	6.9	6.0	1.5	1.3	**1.9**
F3P16_RS15670	0.0	0.0	UC	0.0	0.0	0.0	0.0	1.1	**UC**
F3P16_RS15675	0.3	0.3	1.2	0.6	1.1	2.6	2.0	4.6	**2.1**

F3P16_RS15680	0.5	1.7	3.5	2.3	2.4	4.0	1.1	1.8	**4.8**
pheT	3.0	5.2	1.7	4.3	7.6	8.9	1.8	2.1	**1.4**
pheS	1.0	1.5	1.6	1.2	1.9	2.1	1.6	1.8	**1.2**
F3P16_RS15695	0.1	0.1	1.7	0.1	0.1	0.1	2.0	2.3	**1.1**
F3P16_RS15700	0.1	0.1	1.0	0.1	0.2	0.1	1.6	0.8	**1.1**
F3P16_RS15705	0.1	0.3	1.8	0.2	0.4	0.2	2.2	1.0	**1.4**
rplT	6.9	9.1	1.3	16.3	28.8	53.7	1.8	3.3	**2.3**
rpmI	5.8	7.2	1.2	11.7	22.4	43.2	1.9	3.7	**2.0**
F3P16_RS19105	1.2	1.3	1.1	2.0	3.2	6.1	1.6	3.0	**1.7**
nreB	0.0	0.2	3.3	0.1	0.1	0.2	1.4	2.2	**1.6**
F3P16_RS15725	0.0	0.1	2.3	0.0	0.1	0.1	3.8	4.2	**0.5**
F3P16_RS15730	0.2	0.7	2.7	0.6	0.7	1.0	1.1	1.8	**2.4**
infC	5.0	5.5	1.1	10.1	15.2	28.8	1.5	2.8	**2.0**
thrS	7.1	8.5	1.2	9.6	15.1	17.5	1.6	1.8	**1.4**
F3P16_RS15745	0.0	0.3	6.7	0.2	0.1	0.2	0.8	1.2	**3.8**
F3P16_RS15750	1.3	2.1	1.7	1.9	3.0	4.3	1.6	2.3	**1.5**
F3P16_RS15755	1.3	2.7	2.1	2.4	3.5	5.0	1.5	2.1	**1.9**
rapZ	0.8	1.8	2.2	1.4	2.3	3.2	1.6	2.3	**1.7**
panC	0.8	1.8	2.2	1.2	2.4	3.0	1.9	2.4	**1.5**
panB	1.2	1.8	1.5	1.0	2.3	3.4	2.4	3.5	**0.8**
folK	0.7	1.0	1.5	0.9	1.3	1.5	1.5	1.8	**1.3**
pcnB	1.8	2.4	1.3	2.1	3.2	4.3	1.5	2.0	**1.2**
F3P16_RS15785	0.0	0.0	0.9	0.0	0.1	0.0	3.8	1.6	**0.8**
mazG	0.0	0.0	0.3	0.0	0.1	0.1	2.4	3.0	**1.3**
F3P16_RS15795	0.0	0.1	2.6	0.1	0.1	0.2	1.0	1.6	**4.1**
F3P16_RS15800	1.5	2.4	1.6	1.9	3.2	4.0	1.7	2.1	**1.2**
rlmD	1.4	1.8	1.3	1.8	3.5	3.9	1.9	2.1	**1.3**
F3P16_RS15810	0.4	0.7	1.8	0.7	1.9	1.6	2.7	2.2	**1.8**
cysM	0.9	1.0	1.1	1.0	1.7	1.6	1.6	1.5	**1.2**
F3P16_RS15820	0.1	0.2	1.9	0.1	0.2	0.4	1.8	2.9	**1.1**

F3P16_RS15825	0.2	0.6	3.7	0.2	0.2	0.3	1.3	1.8	**1.2**
F3P16_RS15830	0.4	1.6	3.7	0.4	0.6	0.6	1.5	1.3	**1.0**
F3P16_RS15835	0.3	1.0	3.7	0.7	0.6	1.8	1.0	2.7	**2.4**
rsfS	3.4	4.2	1.3	5.3	6.2	9.2	1.2	1.7	**1.6**
F3P16_RS15845	0.1	0.1	1.7	0.1	0.3	0.3	2.2	2.2	**1.6**
F3P16_RS15850	0.1	0.2	2.0	0.1	0.1	0.2	1.3	1.9	**1.0**
F3P16_RS15855	0.0	0.2	5.0	0.1	0.1	0.1	2.2	1.4	**1.2**
F3P16_RS15860	0.1	0.2	1.8	0.1	0.1	0.1	1.4	1.4	**0.9**
F3P16_RS15865	0.0	0.0	1.1	0.0	0.0	0.0	1.9	2.8	**3.5**
F3P16_RS15870	0.0	0.1	UC	0.0	0.0	0.1	2.5	3.4	**UC**
F3P16_RS15875	0.1	0.2	1.3	0.1	0.2	0.4	1.7	3.2	**0.8**
F3P16_RS15880	0.0	0.0	UC	0.0	0.0	0.0	UC	UC	**UC**
F3P16_RS15885	0.0	0.0	UC	0.0	0.0	0.0	UC	UC	**UC**
F3P16_RS15890	0.0	0.0	UC	0.0	0.0	0.0	UC	UC	**UC**
F3P16_RS15895	0.0	0.0	UC	0.0	0.0	0.0	UC	UC	**UC**
F3P16_RS15900	0.0	0.0	UC	0.0	0.0	0.0	UC	UC	**UC**
F3P16_RS15905	0.0	0.0	UC	0.0	0.0	0.0	UC	UC	**UC**
F3P16_RS15910	0.0	0.0	UC	0.0	0.0	0.0	UC	UC	**UC**
F3P16_RS15915	0.0	0.0	UC	0.0	0.0	0.0	UC	UC	**UC**
F3P16_RS15920	0.0	0.0	UC	0.0	0.0	0.0	UC	UC	**UC**
F3P16_RS15925	0.0	0.0	UC	0.0	0.0	0.0	UC	UC	**UC**
F3P16_RS15930	0.0	0.0	UC	0.0	0.0	0.0	UC	UC	**UC**
F3P16_RS15935	0.0	0.0	UC	0.0	0.0	0.0	UC	UC	**UC**
F3P16_RS15940	0.0	0.0	UC	0.0	0.0	0.0	UC	UC	**UC**
F3P16_RS15945	0.0	0.0	UC	0.0	0.0	0.0	UC	UC	**UC**
F3P16_RS15950	0.0	0.0	UC	0.0	0.0	0.0	UC	UC	**UC**
F3P16_RS15955	0.0	0.0	UC	0.0	0.0	0.0	UC	UC	**UC**
F3P16_RS18920	0.0	0.0	UC	0.0	0.0	0.0	UC	UC	**UC**
F3P16_RS15960	0.0	0.0	UC	0.0	0.0	0.0	UC	UC	**UC**
F3P16_RS15970	0.0	0.0	UC	0.0	0.0	0.0	UC	UC	**UC**

F3P16_RS15975	0.0	0.0	UC	0.0	0.0	0.0	UC	UC	**UC**
F3P16_RS15980	0.0	0.0	UC	0.0	0.0	0.0	UC	UC	**UC**
F3P16_RS15985	0.0	0.0	UC	0.0	0.0	0.0	UC	UC	**UC**
F3P16_RS15990	0.0	0.0	UC	0.0	0.0	0.0	UC	UC	**UC**
F3P16_RS15995	0.0	0.0	UC	0.0	0.0	0.0	UC	UC	**UC**
dusA	0.9	1.5	1.7	1.3	1.7	1.6	1.3	1.2	**1.5**
F3P16_RS16005	0.0	0.6	24.3	0.2	0.1	0.3	0.6	1.7	**7.4**
F3P16_RS16010	0.0	0.1	3.8	0.0	0.0	0.2	1.5	5.0	**1.9**
F3P16_RS16015	0.0	0.0	UC	0.0	0.0	0.0	UC	UC	**UC**
F3P16_RS16020	0.1	0.2	2.1	0.1	0.3	0.4	2.1	2.9	**1.9**
acnA	2.8	9.8	3.5	5.9	7.2	5.4	1.2	0.9	**2.1**
F3P16_RS16030	0.2	0.6	2.8	0.4	0.4	0.9	1.2	2.3	**1.7**
F3P16_RS16035	0.2	0.5	2.4	0.5	0.5	1.1	1.0	2.4	**2.2**
F3P16_RS16040	0.1	0.2	1.7	0.3	0.5	0.3	1.7	0.9	**2.9**
F3P16_RS16045	1.3	1.5	1.1	1.8	2.7	3.0	1.5	1.6	**1.4**
F3P16_RS16050	0.1	0.1	0.9	0.1	0.2	0.6	1.8	6.1	**0.7**
F3P16_RS16055	0.0	0.0	UC	0.0	0.0	0.0	0.7	2.3	**UC**
F3P16_RS16060	0.0	0.0	0.5	0.0	0.0	0.1	0.0	12.0	**1.0**
F3P16_RS16070	0.1	0.1	2.0	0.1	0.1	0.2	1.0	3.0	**1.3**
ilvC	2.4	3.1	1.3	3.6	5.8	9.7	1.6	2.7	**1.5**
ilvN	5.0	9.9	2.0	9.4	15.1	17.4	1.6	1.9	**1.9**
F3P16_RS16085	2.9	5.3	1.9	4.0	6.4	10.0	1.6	2.5	**1.4**
F3P16_RS16090	0.0	0.0	0.7	0.0	0.0	0.1	UC	UC	**0.0**
leuS	1.6	2.7	1.7	2.7	4.5	5.9	1.7	2.2	**1.7**
F3P16_RS16100	2.3	5.0	2.2	5.4	7.7	10.9	1.4	2.0	**2.3**
holA	0.3	0.7	2.5	0.7	1.5	1.5	2.0	2.0	**2.5**
F3P16_RS16110	0.2	23.0	112.3	7.8	12.7	5.7	1.6	0.7	**38.2**
F3P16_RS16115	0.4	46.5	117.6	17.4	27.0	11.9	1.6	0.7	**44.0**
F3P16_RS16120	0.4	31.9	84.2	13.4	24.1	11.0	1.8	0.8	**35.4**
F3P16_RS16125	1.5	1.0	0.7	1.7	2.9	5.6	1.7	3.2	**1.1**

F3P16_RS16130	0.4	0.6	1.7	0.8	1.0	1.5	1.3	2.0	**2.1**
orn]	0.1	0.1	1.5	0.1	0.1	0.2	1.3	2.1	**1.5**
rsgA	0.5	0.5	1.0	0.4	0.5	0.8	1.2	1.9	**0.8**
F3P16_RS16145	3.2	4.6	1.5	6.5	7.2	18.6	1.1	2.8	**2.0**
grxC	2.7	4.2	1.6	6.0	7.5	20.9	1.3	3.5	**2.2**
secB	0.4	0.7	1.8	1.6	1.9	5.5	1.2	3.5	**4.1**
F3P16_RS16160	0.0	0.1	1.6	0.0	0.1	0.1	2.9	3.0	**1.3**
der]	3.2	4.6	1.4	4.7	6.7	6.9	1.4	1.5	**1.5**
bamB	2.1	4.4	2.0	5.2	7.7	8.2	1.5	1.6	**2.4**
F3P16_RS16175	0.7	1.2	1.6	1.6	3.1	2.8	1.9	1.7	**2.3**
hisS	1.8	2.7	1.5	2.9	5.2	4.5	1.8	1.5	**1.6**
ispG	1.7	2.5	1.4	2.7	5.0	4.8	1.8	1.8	**1.6**
F3P16_RS16190	2.0	2.5	1.2	2.1	4.0	4.4	1.9	2.1	**1.0**
pilW	0.8	0.9	1.2	0.9	1.4	1.9	1.5	2.0	**1.2**
rlmN	3.0	4.6	1.5	4.6	6.8	6.5	1.5	1.4	**1.6**
ndk]	1.5	2.3	1.5	2.5	3.5	5.7	1.4	2.3	**1.7**
iscX	0.1	0.4	3.7	0.2	0.3	1.0	1.3	4.3	**2.4**
F3P16_RS16215	0.0	0.3	6.0	0.2	0.1	0.4	0.8	2.3	**3.3**
F3P16_RS16220	0.1	0.4	5.1	0.4	0.4	0.5	1.1	1.3	**5.2**
F3P16_RS16225	0.7	4.4	6.7	3.8	3.8	5.5	1.0	1.4	**5.8**
nagZ	0.7	2.0	3.0	1.7	1.8	2.9	1.1	1.7	**2.5**
F3P16_RS16235	0.1	0.2	2.0	0.2	0.2	0.3	1.3	1.6	**2.0**
F3P16_RS16240	0.2	0.2	0.9	0.1	0.2	0.3	1.1	1.8	**0.8**
F3P16_RS16245	3.4	6.0	1.8	4.0	4.7	7.1	1.2	1.8	**1.2**
F3P16_RS16250	1.9	4.4	2.4	3.1	4.9	4.1	1.6	1.3	**1.7**
F3P16_RS16255	2.1	6.3	3.0	5.6	9.7	10.2	1.7	1.8	**2.6**
F3P16_RS16260	2.1	6.4	3.1	5.1	8.7	9.4	1.7	1.8	**2.5**
eda]	2.5	8.9	3.5	7.7	12.0	16.9	1.6	2.2	**3.1**
edd]	2.4	8.1	3.4	6.2	10.1	15.3	1.6	2.5	**2.6**
F3P16_RS16275	0.2	1.1	5.8	0.7	0.7	1.1	1.0	1.4	**3.8**

pta]	0.5	2.1	4.0	1.6	1.9	1.7	1.2	1.0	**3.1**
F3P16_RS16285	3.9	5.3	1.4	4.5	6.8	9.9	1.5	2.2	**1.2**
F3P16_RS16290	0.0	0.1	1.5	0.2	0.2	0.4	1.4	2.6	**4.9**
F3P16_RS16295	0.0	0.1	1.6	0.1	0.1	0.2	1.2	2.8	**1.6**
clpX	1.0	1.9	2.0	2.0	2.1	4.8	1.1	2.5	**2.0**
clpP	2.4	4.5	1.8	3.9	4.8	8.5	1.2	2.2	**1.6**
tig]	2.8	3.4	1.2	4.2	5.9	12.9	1.4	3.1	**1.5**
F3P16_RS16315	0.5	0.2	0.4	0.1	0.4	1.2	3.6	10.0	**0.2**
F3P16_RS16320	0.1	0.2	3.0	0.2	0.2	0.4	1.2	2.3	**2.9**
leuA	1.5	4.0	2.6	3.6	3.3	4.3	0.9	1.2	**2.4**
F3P16_RS16330	2.5	6.3	2.5	4.6	6.2	5.6	1.4	1.2	**1.8**
metW	3.4	6.6	2.0	5.5	7.4	7.6	1.3	1.4	**1.6**
F3P16_RS16340	1.8	3.0	1.7	2.2	3.6	3.7	1.6	1.6	**1.3**
rdgB	0.3	0.3	1.2	0.3	0.4	0.5	1.5	1.7	**1.0**
F3P16_RS16350	0.0	0.0	1.2	0.0	0.1	0.1	1.3	2.8	**2.2**
F3P16_RS16355	0.0	0.0	UC	0.0	0.1	0.1	3.7	3.8	**UC**
tatB	0.0	0.0	2.1	0.1	0.2	0.1	3.9	1.5	**6.9**
tatC	0.0	0.0	UC	0.0	0.1	0.1	1.4	2.1	**UC**
F3P16_RS16370	0.0	0.1	2.7	0.1	0.1	0.1	2.3	2.3	**3.3**
F3P16_RS16375	0.0	0.0	1.5	0.0	0.0	0.1	1.8	3.4	**1.3**
F3P16_RS16380	0.2	0.2	1.2	0.2	0.2	0.4	1.1	2.1	**1.0**
lgt]	2.0	4.0	2.0	3.1	3.4	9.0	1.1	2.9	**1.5**
F3P16_RS16390	0.1	0.1	0.7	0.1	0.2	0.3	2.3	3.4	**1.1**
F3P16_RS16395	0.1	0.1	1.2	0.1	0.1	0.1	2.2	2.7	**0.5**
thyA	9.0	15.7	1.8	12.8	17.5	19.6	1.4	1.5	**1.4**
F3P16_RS16405	4.7	8.3	1.8	7.5	11.0	11.5	1.5	1.5	**1.6**
F3P16_RS16410	1.2	2.4	2.0	2.2	4.2	3.9	1.9	1.8	**1.8**
msrA	6.2	13.1	2.1	14.2	26.9	32.8	1.9	2.3	**2.3**
F3P16_RS16420	1.2	2.2	1.8	3.4	4.6	9.4	1.4	2.8	**2.8**
F3P16_RS16425	1.0	1.9	1.8	2.5	4.0	7.3	1.6	3.0	**2.4**

F3P16_RS16430	0.1	0.2	1.2	0.3	0.6	1.0	2.2	3.7	**1.7**
purU	0.4	0.6	1.6	0.7	0.8	1.4	1.1	1.9	**1.8**
F3P16_RS16440	0.2	0.4	2.2	0.4	0.5	0.9	1.2	2.2	**2.2**
F3P16_RS16445	5.0	6.6	1.3	6.4	4.6	5.1	0.7	0.8	**1.3**
rpmB	5.2	6.5	1.3	11.9	18.4	31.9	1.5	2.7	**2.3**
rpmG	1.1	2.5	2.2	5.5	10.7	15.8	2.0	2.9	**4.8**
F3P16_RS16460	0.5	1.0	1.9	1.1	1.6	3.0	1.4	2.7	**2.0**
F3P16_RS16465	0.5	2.3	4.1	2.1	1.9	7.3	0.9	3.5	**3.8**
F3P16_RS16470	0.9	1.9	2.2	1.7	2.1	3.5	1.2	2.0	**2.0**
F3P16_RS18980	0.4	0.7	1.8	0.6	1.1	1.6	1.7	2.4	**1.7**
F3P16_RS16475	0.1	0.5	3.9	0.3	0.6	0.5	2.1	1.7	**2.3**
F3P16_RS16480	0.3	0.6	1.9	0.6	0.8	1.2	1.3	1.9	**1.9**
F3P16_RS16485	0.0	0.2	10.7	0.2	0.1	0.8	0.6	3.2	**11.5**
F3P16_RS16490	0.0	0.1	2.9	0.0	0.1	0.1	3.3	4.9	**1.5**
topA	6.6	10.6	1.6	8.3	10.9	13.3	1.3	1.6	**1.3**
F3P16_RS16500	1.3	2.5	1.9	1.5	2.3	3.5	1.5	2.3	**1.2**
F3P16_RS16505	0.1	0.2	2.6	0.1	0.2	0.7	1.3	5.2	**2.1**
F3P16_RS16510	0.2	0.3	1.4	0.3	0.5	0.7	1.4	1.9	**1.7**
F3P16_RS16515	0.1	0.3	2.7	0.6	1.1	1.2	1.7	2.0	**5.3**
F3P16_RS16520	0.4	0.7	2.0	0.6	0.8	1.1	1.5	2.0	**1.5**
F3P16_RS16525	0.1	0.1	1.0	0.0	0.1	0.1	2.0	4.4	**0.6**
lpxL	0.2	0.3	2.1	0.2	0.3	0.4	1.6	1.8	**1.3**
F3P16_RS16535	0.4	0.5	1.3	0.5	0.6	0.8	1.1	1.5	**1.4**
gltP	0.9	1.3	1.5	2.4	3.0	5.1	1.2	2.1	**2.8**
F3P16_RS16545	0.1	0.2	1.9	0.1	0.1	0.7	1.1	5.8	**0.9**
asd]	0.5	0.5	1.0	0.5	0.6	1.4	1.2	3.1	**0.8**
F3P16_RS16555	0.1	0.1	1.0	0.1	0.2	0.2	2.2	1.9	**0.9**
F3P16_RS16560	0.1	0.1	1.2	0.2	0.3	0.2	1.8	1.3	**1.4**
F3P16_RS16565	0.5	0.8	1.5	0.6	1.0	1.6	1.7	2.5	**1.2**
truA	1.8	3.3	1.8	2.0	2.8	4.6	1.4	2.3	**1.1**

F3P16_RS16575	0.4	1.4	3.2	1.4	1.3	3.0	1.0	2.2	**3.0**
infA	0.5	0.6	1.3	0.7	0.9	2.1	1.1	2.9	**1.6**
F3P16_RS16585	0.0	0.0	1.3	0.1	0.1	0.1	1.2	1.6	**4.0**
F3P16_RS16590	0.0	0.0	UC	0.0	0.0	0.1	UC	UC	**UC**
leuB	0.5	0.7	1.3	0.7	0.9	1.3	1.3	1.9	**1.3**
F3P16_RS16600	1.3	2.4	1.8	2.1	2.7	4.5	1.3	2.2	**1.6**
leuD	0.3	0.8	2.8	0.5	0.9	1.0	2.0	2.1	**1.5**
leuC	0.4	1.0	2.6	0.6	0.9	0.9	1.5	1.5	**1.6**
F3P16_RS16615	0.0	0.0	UC	0.0	0.0	0.1	2.0	4.0	**UC**
F3P16_RS16620	0.2	0.3	1.2	0.2	0.3	0.5	1.1	2.3	**1.2**
F3P16_RS16625	0.1	0.1	1.2	0.1	0.1	0.2	1.9	3.0	**0.7**
F3P16_RS16630	0.0	0.1	7.9	0.1	0.1	0.2	1.2	2.5	**5.5**
F3P16_RS16635	0.0	0.1	2.0	0.1	0.1	0.2	1.8	2.3	**2.4**
F3P16_RS16640	0.8	1.6	2.0	1.3	1.6	2.0	1.2	1.6	**1.6**
ptsP	0.8	1.3	1.6	1.0	1.3	1.7	1.3	1.8	**1.2**
katG	0.9	3.6	3.8	2.7	4.2	4.4	1.6	1.6	**2.9**
F3P16_RS16655	0.1	0.2	3.1	0.2	0.2	0.5	1.2	3.3	**2.4**
hcaR	0.0	0.0	3.2	0.0	0.0	0.1	1.2	3.3	**3.6**
F3P16_RS16665	0.1	0.0	0.7	0.1	0.0	0.3	0.4	3.4	**1.2**
F3P16_RS16670	0.1	0.1	1.3	0.1	0.1	0.3	1.3	2.9	**1.1**
F3P16_RS16675	0.0	0.1	1.7	0.1	0.1	0.1	1.6	1.7	**2.1**
F3P16_RS16680	0.9	1.7	1.8	1.4	1.9	2.1	1.4	1.6	**1.4**
F3P16_RS16685	1.4	2.1	1.5	1.8	2.5	2.7	1.4	1.5	**1.3**
F3P16_RS16695	4.1	9.5	2.3	7.3	12.4	14.1	1.7	1.9	**1.8**
F3P16_RS16700	0.3	0.6	2.0	0.5	0.8	1.3	1.6	2.5	**1.8**
F3P16_RS16705	0.1	0.0	0.7	0.1	0.1	0.5	1.0	5.1	**1.3**
F3P16_RS16710	0.1	0.0	0.7	0.1	0.1	0.5	1.0	5.1	**1.3**
F3P16_RS16715	0.0	0.0	0.7	0.0	0.0	0.1	1.5	3.5	**0.6**
F3P16_RS16720	0.5	0.3	0.7	0.3	0.6	0.5	1.9	1.6	**0.6**
hemW	0.9	1.0	1.1	1.2	1.8	1.6	1.6	1.3	**1.3**

F3P16_RS16730	0.0	0.0	1.8	0.0	0.2	0.1	4.4	2.2	**1.4**
abeM	0.4	0.6	1.4	0.4	0.6	0.9	1.3	2.1	**1.0**
F3P16_RS16740	0.1	0.2	1.5	0.2	0.2	0.2	1.3	1.2	**1.3**
F3P16_RS16745	0.0	0.1	1.7	0.1	0.1	0.1	1.0	1.0	**1.3**
F3P16_RS16750	0.1	0.9	7.3	1.3	1.1	2.3	0.9	1.8	**10.2**
F3P16_RS16755	0.0	0.3	UC	0.7	1.2	1.4	1.7	1.9	**UC**
F3P16_RS16760	0.1	0.2	3.9	0.3	0.3	0.6	0.9	1.8	**5.7**
crcB	0.1	0.1	0.9	0.1	0.1	0.3	1.7	3.8	**0.9**
F3P16_RS16770	0.2	0.2	1.5	0.3	0.2	0.6	0.8	2.1	**1.9**
F3P16_RS16775	0.2	0.2	1.3	0.2	0.2	0.5	1.5	3.0	**1.0**
F3P16_RS16780	0.1	0.2	1.7	0.2	0.3	0.7	1.3	2.8	**1.8**
F3P16_RS16785	0.3	1.0	2.8	0.6	0.8	1.9	1.3	3.2	**1.7**
F3P16_RS16790	0.5	1.0	2.0	0.8	1.1	2.0	1.4	2.5	**1.5**
hemH	0.1	0.2	2.1	0.2	0.2	0.5	1.3	2.9	**2.1**
F3P16_RS16800	0.0	0.0	0.9	0.0	0.0	0.1	0.8	4.6	**0.7**
murI	0.4	0.8	1.7	0.6	0.7	1.9	1.2	3.4	**1.3**
F3P16_RS16810	0.2	0.4	2.0	0.4	0.6	0.8	1.4	2.1	**1.9**
F3P16_RS16815	2.5	5.3	2.1	4.4	5.8	8.5	1.3	2.0	**1.7**
F3P16_RS18985	0.0	0.0	UC	0.0	0.0	0.0	UC	UC	**UC**
tusA	0.0	0.0	3.2	0.1	0.0	0.1	0.5	1.5	**6.5**
F3P16_RS16830	0.5	1.0	2.0	0.8	1.1	2.0	1.4	2.4	**1.6**
lnt]	0.9	2.2	2.4	2.1	2.4	4.3	1.1	2.1	**2.3**
F3P16_RS16840	0.1	0.4	4.8	0.3	0.2	0.7	0.8	2.5	**3.4**
F3P16_RS16845	0.1	0.1	1.0	0.1	0.1	0.2	0.9	2.9	**1.1**
gspG	0.4	0.5	1.3	0.4	0.5	0.8	1.1	1.9	**1.1**
gspF	0.2	0.3	1.5	0.2	0.3	0.5	1.6	2.3	**1.0**
F3P16_RS16860	0.4	0.4	1.2	0.4	0.4	0.7	1.2	2.0	**1.0**
F3P16_RS16865	0.3	0.4	1.6	0.3	0.6	0.8	1.7	2.5	**1.3**
F3P16_RS16870	0.5	1.0	2.0	1.1	1.3	5.2	1.2	4.7	**2.1**
F3P16_RS16875	0.0	0.0	1.1	0.0	0.0	0.0	0.9	2.0	**2.6**

F3P16_RS16880	0.3	1.1	3.3	0.6	0.9	1.1	1.4	1.7	**2.0**
F3P16_RS16885	0.1	0.4	5.0	0.2	0.2	0.6	1.1	2.8	**2.8**
F3P16_RS16890	0.1	0.1	2.0	0.2	0.2	0.4	1.2	2.5	**2.1**
pnp]	4.9	9.3	1.9	9.8	15.7	24.2	1.6	2.5	**2.0**
rpsO	3.6	3.7	1.0	7.0	5.8	13.0	0.8	1.8	**1.9**
F3P16_RS16905	0.0	0.0	0.9	0.0	0.1	0.1	1.6	2.9	**1.0**
recD	0.2	0.2	1.1	0.2	0.4	0.5	2.2	2.7	**1.1**
F3P16_RS16915	0.5	0.6	1.3	0.6	1.2	1.3	2.0	2.1	**1.3**
F3P16_RS16920	0.4	0.6	1.7	0.5	0.9	0.9	1.9	1.9	**1.3**
F3P16_RS16925	0.1	0.7	4.9	0.7	0.6	1.1	0.9	1.6	**4.8**
F3P16_RS16930	0.2	0.8	3.7	0.7	0.6	0.9	0.8	1.3	**2.9**
F3P16_RS16935	0.1	0.1	1.1	0.1	0.1	0.2	1.6	2.5	**0.8**
ubiE	1.4	2.9	2.1	2.4	3.8	5.6	1.6	2.3	**1.8**
F3P16_RS16945	2.2	5.3	2.5	4.0	7.0	8.5	1.7	2.1	**1.9**
F3P16_RS16950	1.2	2.2	1.8	2.0	2.9	3.6	1.5	1.9	**1.6**
F3P16_RS16955	0.1	0.1	1.3	0.0	0.1	0.2	2.3	3.7	**0.4**
hisI	1.0	1.2	1.2	1.0	1.8	1.6	1.8	1.6	**1.0**
F3P16_RS16965	3.7	4.3	1.2	3.8	6.2	4.8	1.6	1.3	**1.0**
F3P16_RS16970	0.3	0.5	1.6	0.5	0.7	0.5	1.5	1.2	**1.5**
F3P16_RS16975	2.4	3.7	1.5	2.4	4.7	4.2	1.9	1.8	**1.0**
F3P16_RS16980	4.3	5.8	1.4	4.2	6.8	7.2	1.6	1.7	**1.0**
F3P16_RS16985	1.7	3.0	1.8	2.4	4.6	4.3	1.9	1.8	**1.5**
F3P16_RS16990	1.8	2.1	1.2	1.6	3.2	3.6	2.0	2.2	**0.9**
F3P16_RS16995	2.5	3.1	1.2	1.8	3.7	4.7	2.1	2.6	**0.7**
F3P16_RS17000	1.0	1.4	1.4	0.9	1.7	2.0	1.8	2.2	**0.9**
F3P16_RS17005	35.9	49.5	1.4	44.7	80.7	156.4	1.8	3.5	**1.2**
infB	30.5	59.8	2.0	52.1	123.4	169.3	2.4	3.2	**1.7**
nusA	10.5	17.9	1.7	18.9	39.2	41.3	2.1	2.2	**1.8**
rimP	7.8	9.4	1.2	10.8	18.7	15.2	1.7	1.4	**1.4**
F3P16_RS17025	5.4	7.5	1.4	11.9	18.9	22.3	1.6	1.9	**2.2**

F3P16_RS17030	5.4	7.5	1.4	11.9	18.9	22.3	1.6	1.9	**2.2**
F3P16_RS17035	0.9	1.5	1.6	1.7	2.7	4.9	1.6	2.9	**1.9**
secG	1.7	3.4	2.0	3.7	5.3	8.8	1.4	2.4	**2.1**
tpiA	2.6	3.8	1.5	4.3	7.1	10.1	1.6	2.3	**1.7**
pilB	0.0	0.0	1.1	0.0	0.0	0.1	1.1	5.1	**0.7**
F3P16_RS17055	0.1	0.1	1.5	0.1	0.1	0.2	1.2	3.2	**1.0**
F3P16_RS19110	0.2	0.2	1.3	0.2	0.3	0.8	1.5	3.5	**1.2**
F3P16_RS19115	0.1	0.2	1.7	0.1	0.1	0.3	2.2	4.2	**0.7**
coaE	0.2	0.2	1.2	0.2	0.3	0.9	1.6	4.1	**1.0**
F3P16_RS17070	0.5	0.8	1.6	0.8	1.3	1.3	1.6	1.7	**1.5**
rlmB	1.5	2.1	1.4	1.8	2.5	3.0	1.4	1.6	**1.2**
F3P16_RS17080	0.2	0.3	1.2	0.3	0.3	0.7	1.2	2.3	**1.2**
F3P16_RS17085	0.1	0.1	1.0	0.1	0.1	0.2	0.9	2.9	**1.2**
recN	0.2	0.2	1.5	0.2	0.2	0.6	1.4	3.7	**1.0**
F3P16_RS17095	5.5	11.0	2.0	8.8	9.8	20.9	1.1	2.4	**1.6**
ruvX	6.0	15.4	2.6	13.2	15.6	31.2	1.2	2.4	**2.2**
F3P16_RS17105	0.1	0.3	4.9	0.3	0.3	0.4	1.0	1.3	**5.8**
F3P16_RS17110	0.0	0.1	2.1	0.1	0.1	0.1	0.9	1.0	**4.5**
F3P16_RS17115	0.1	0.2	2.4	0.2	0.3	0.2	1.3	1.1	**2.6**
F3P16_RS17120	0.1	0.1	1.1	0.1	0.2	0.2	1.3	1.3	**1.8**
F3P16_RS17125	0.0	0.1	1.7	0.1	0.1	0.2	1.4	2.1	**1.7**
F3P16_RS17130	0.1	0.2	1.4	0.2	0.2	0.3	1.5	1.9	**1.4**
pgsA	0.4	0.6	1.7	0.7	0.7	1.8	1.0	2.5	**2.0**
F3P16_RS17140	0.1	0.2	3.3	0.2	0.1	0.5	0.7	3.4	**3.0**
uvrC	0.2	0.5	2.1	0.4	0.4	1.8	1.1	4.9	**1.5**
F3P16_RS17150	0.0	0.0	12.7	0.0	0.0	0.1	1.8	10.0	**4.0**
lpxO	0.3	0.3	1.2	0.3	0.4	0.9	1.4	3.0	**1.0**
fadB	3.1	17.0	5.4	7.5	5.9	5.8	0.8	0.8	**2.4**
fadA	4.5	24.0	5.4	10.0	8.1	8.1	0.8	0.8	**2.2**
F3P16_RS17175	0.0	0.0	0.4	0.0	0.0	0.0	UC	UC	**0.0**

F3P16_RS17180	0.0	0.0	0.5	0.0	0.0	0.0	0.0	1.9	**0.7**
F3P16_RS17185	0.0	0.0	UC	0.0	0.0	0.1	0.8	1.9	**UC**
F3P16_RS17190	0.0	0.0	UC	0.0	0.0	0.1	0.3	1.6	**UC**
F3P16_RS17195	0.0	0.0	UC	0.0	0.0	0.0	UC	UC	**UC**
F3P16_RS17200	0.2	0.5	2.3	0.6	0.5	2.2	0.9	3.5	**2.7**
F3P16_RS17205	0.2	0.4	2.1	0.2	0.2	0.7	0.9	2.8	**1.2**
F3P16_RS17210	0.0	0.0	UC	0.0	0.0	0.0	0.3	2.3	**UC**
htpG	0.3	0.7	2.2	0.7	0.8	2.4	1.2	3.6	**2.0**
F3P16_RS17220	0.2	0.5	3.1	0.9	0.7	1.3	0.8	1.5	**5.2**
F3P16_RS17225	7.3	8.9	1.2	9.5	13.2	13.9	1.4	1.5	**1.3**
F3P16_RS17230	0.5	0.7	1.5	0.8	1.0	1.6	1.2	2.0	**1.7**
F3P16_RS17235	0.1	0.5	7.8	0.3	0.3	0.4	1.0	1.5	**4.5**
F3P16_RS17240	0.0	0.2	UC	0.2	0.2	0.2	1.0	1.2	**UC**
F3P16_RS17245	0.0	2.5	266.7	1.0	0.9	1.0	0.8	1.0	**110.7**
F3P16_RS17250	0.0	5.0	107.9	3.7	4.4	3.9	1.2	1.1	**81.1**
rpoC	11.1	16.3	1.5	20.0	34.4	52.6	1.7	2.6	**1.8**
rpoB	11.5	18.0	1.6	20.3	33.7	34.4	1.7	1.7	**1.8**
rplL	2.9	4.2	1.5	8.3	14.0	28.2	1.7	3.4	**2.9**
rplJ	15.3	22.2	1.4	43.2	59.9	125.7	1.4	2.9	**2.8**
rplA	46.8	70.3	1.5	113.8	171.2	334.3	1.5	2.9	**2.4**
rplK	31.7	47.3	1.5	64.6	114.7	155.3	1.8	2.4	**2.0**
nusG	17.3	22.4	1.3	28.5	50.5	52.2	1.8	1.8	**1.6**
secE	3.3	4.7	1.4	5.0	9.6	12.6	1.9	2.5	**1.5**
F3P16_RS17295	5.3	6.0	1.1	6.5	11.7	22.1	1.8	3.4	**1.2**
tuf]	60.0	95.9	1.6	105.0	190.0	305.6	1.8	2.9	**1.8**
F3P16_RS17305	4.8	6.4	1.3	5.8	9.1	12.1	1.6	2.1	**1.2**
F3P16_RS17310	6.6	6.8	1.0	7.2	9.4	14.6	1.3	2.0	**1.1**
F3P16_RS17315	4.7	4.5	1.0	4.0	6.0	9.3	1.5	2.3	**0.9**
F3P16_RS17320	6.3	7.6	1.2	12.5	13.0	30.2	1.0	2.4	**2.0**
trpE	0.4	0.9	2.3	0.8	1.0	1.4	1.3	1.8	**1.9**

F3P16_RS17330	0.2	0.6	2.7	0.4	0.6	0.9	1.5	2.1	**1.9**
F3P16_RS17335	0.3	0.7	2.3	0.7	1.0	1.7	1.4	2.5	**2.2**
gspD	0.3	0.7	2.3	0.6	1.0	1.2	1.6	1.9	**2.0**
F3P16_RS17345	0.4	0.9	2.1	0.6	0.8	1.3	1.3	2.1	**1.5**
F3P16_RS17350	0.1	0.2	2.1	0.2	0.2	0.4	0.8	2.2	**1.8**
F3P16_RS17355	0.1	0.2	1.7	0.7	0.8	2.1	1.1	3.1	**6.8**
F3P16_RS17360	0.0	0.0	2.9	0.0	0.0	0.1	1.6	2.3	**2.0**
F3P16_RS17365	0.1	0.3	2.8	0.4	0.4	0.4	1.3	1.2	**3.1**
F3P16_RS17370	0.2	0.4	2.1	0.3	0.5	0.6	1.9	2.3	**1.7**
F3P16_RS17375	0.3	0.5	1.7	0.4	0.6	1.2	1.6	3.2	**1.1**
hemC	1.1	1.8	1.7	1.2	1.3	2.8	1.1	2.2	**1.1**
F3P16_RS17385	0.2	0.3	1.4	0.3	0.3	1.0	1.2	3.7	**1.2**
F3P16_RS17390	0.0	0.0	1.8	0.0	0.0	0.1	6.5	22.2	**0.2**
argH	1.3	2.2	1.8	2.0	3.0	4.1	1.5	2.0	**1.6**
F3P16_RS17400	0.3	0.5	2.1	0.9	2.0	3.0	2.4	3.5	**3.4**
phoU	0.1	0.3	2.0	0.5	0.6	0.9	1.3	2.1	**3.1**
F3P16_RS17410	0.1	0.1	1.4	0.1	0.1	0.4	1.2	3.1	**1.4**
F3P16_RS17415	0.0	0.0	5.3	0.0	0.0	0.2	4.6	18.9	**1.2**
F3P16_RS17420	0.0	0.0	4.2	0.0	0.0	0.2	2.5	33.7	**1.0**
F3P16_RS17425	0.0	0.1	1.6	0.1	0.1	0.1	1.0	1.8	**1.7**
thiC	1.7	3.5	2.0	3.3	3.8	5.1	1.2	1.6	**1.9**
F3P16_RS17435	0.3	0.7	2.5	0.5	0.9	1.0	1.9	2.0	**1.9**
cpdA	0.1	0.2	2.2	0.1	0.2	0.3	2.1	3.2	**1.1**
dksA	4.6	7.3	1.6	6.5	6.1	11.5	0.9	1.8	**1.4**
gluQ	1.2	1.6	1.4	1.1	1.6	2.6	1.5	2.4	**0.9**
ftsW	0.8	1.8	2.2	1.5	1.7	3.3	1.2	2.2	**1.9**
murD	0.5	0.6	1.3	0.5	0.5	1.0	1.1	2.2	**1.0**
F3P16_RS17465	0.2	0.2	1.0	0.3	0.2	0.8	0.6	2.7	**1.9**
F3P16_RS17470	1.1	0.8	0.8	1.0	1.8	3.5	1.8	3.6	**0.9**
F3P16_RS17475	0.5	0.3	0.6	0.5	0.9	2.0	1.9	4.2	**0.9**

xerD	0.1	0.2	1.4	0.1	0.1	0.3	1.1	2.1	**1.2**
F3P16_RS17485	0.5	1.2	2.3	1.0	1.1	2.9	1.1	2.8	**2.1**
F3P16_RS17490	1.4	2.5	1.8	2.0	3.1	4.6	1.6	2.3	**1.4**
thrC	4.1	6.4	1.5	6.4	10.4	14.2	1.6	2.2	**1.5**
pbpG	2.2	5.5	2.5	4.7	4.1	9.0	0.9	1.9	**2.2**
F3P16_RS17505	1.2	2.5	2.0	2.2	2.3	4.1	1.0	1.8	**1.8**
F3P16_RS17510	0.0	0.1	4.2	0.1	0.1	0.2	1.1	1.2	**7.7**
F3P16_RS17515	0.0	0.1	1.2	0.0	0.1	0.1	1.2	2.1	**1.1**
F3P16_RS17520	0.2	0.5	2.2	0.5	0.8	0.7	1.5	1.3	**2.3**
gpmI	2.9	5.3	1.8	5.0	6.7	7.1	1.3	1.4	**1.7**
lptG	0.7	1.7	2.6	1.4	2.0	2.1	1.4	1.5	**2.0**
lptF	1.4	2.9	2.2	2.7	3.0	4.1	1.1	1.5	**2.0**
F3P16_RS17540	1.4	3.1	2.2	2.7	3.2	3.3	1.2	1.2	**1.8**
F3P16_RS17545	0.7	1.6	2.5	1.4	1.8	1.5	1.3	1.0	**2.1**
F3P16_RS17550	0.2	0.6	2.8	0.8	0.8	1.0	1.0	1.2	**3.7**
F3P16_RS17555	0.1	0.2	1.7	0.1	0.5	0.3	3.2	2.1	**1.6**
F3P16_RS17560	1.8	3.2	1.8	5.0	5.9	7.6	1.2	1.5	**2.8**
F3P16_RS17565	2.5	3.6	1.4	4.1	5.7	6.7	1.4	1.6	**1.6**
ribD	1.1	1.5	1.4	1.5	1.9	2.5	1.3	1.7	**1.4**
nrdR	1.2	1.7	1.4	2.3	2.9	3.8	1.3	1.7	**1.9**
F3P16_RS17580	0.5	0.8	1.7	0.7	0.7	1.5	1.1	2.2	**1.3**
glnK	1.1	1.7	1.6	2.5	2.4	5.9	1.0	2.4	**2.3**
F3P16_RS17590	0.0	0.0	UC	0.0	0.1	0.1	2.0	3.1	**UC**
F3P16_RS17595	0.1	0.1	0.9	0.1	0.1	0.3	1.4	6.4	**0.8**
F3P16_RS17600	0.1	0.1	1.3	0.0	0.1	0.2	2.0	4.1	**0.5**
F3P16_RS17605	0.1	0.1	1.5	0.0	0.1	0.3	2.1	8.2	**0.6**
F3P16_RS17610	0.0	0.1	2.4	0.1	0.1	0.2	1.2	3.2	**2.1**
F3P16_RS17615	0.0	0.1	8.4	0.0	0.0	0.0	0.3	2.1	**3.3**
F3P16_RS19120	0.0	0.0	UC	0.0	0.0	0.0	UC	UC	**UC**
F3P16_RS17620	1.3	1.5	1.2	1.2	2.0	2.8	1.7	2.4	**0.9**

ppa]	0.8	1.6	1.9	1.7	2.4	4.0	1.4	2.3	**2.0**
F3P16_RS17630	0.1	0.1	1.9	0.1	0.3	0.4	1.7	2.4	**1.9**
F3P16_RS17635	0.0	0.0	1.8	0.1	0.1	0.1	0.9	1.7	**4.5**
F3P16_RS17640	0.7	1.4	2.0	1.0	1.0	1.6	1.0	1.6	**1.4**
parC	1.1	1.7	1.5	1.4	1.4	2.6	1.0	1.8	**1.3**
F3P16_RS17650	0.3	0.4	1.6	0.4	0.4	0.6	1.0	1.5	**1.5**
F3P16_RS17655	0.1	0.2	1.4	0.2	0.3	0.4	1.5	2.2	**1.4**
F3P16_RS17660	0.1	0.2	2.1	0.2	0.1	0.2	0.7	1.3	**2.1**
F3P16_RS17665	0.9	1.9	2.1	2.1	2.1	2.5	1.0	1.1	**2.5**
F3P16_RS17670	0.1	0.8	8.2	0.7	0.5	0.9	0.7	1.3	**7.6**
mdtD	0.1	0.5	4.2	0.4	0.4	0.7	1.0	1.6	**3.8**
F3P16_RS17680	0.0	0.1	3.8	0.2	0.1	0.2	0.9	1.4	**5.0**
dinB	0.1	0.1	2.0	0.2	0.3	0.3	1.5	1.7	**2.7**
F3P16_RS19125	0.0	0.0	UC	0.0	0.0	0.1	UC	UC	**UC**
F3P16_RS17690	0.0	0.1	2.3	0.1	0.1	0.3	0.9	2.7	**3.5**
F3P16_RS17695	0.0	0.0	0.8	0.0	0.1	0.2	2.3	6.8	**1.7**
F3P16_RS17700	1.7	2.3	1.4	2.4	2.8	2.1	1.2	0.9	**1.4**
F3P16_RS17705	0.0	0.0	1.1	0.0	0.0	0.1	0.6	2.8	**3.5**
F3P16_RS17710	0.0	0.0	0.0	0.0	0.0	0.0	UC	UC	**0.0**
F3P16_RS17715	0.0	0.1	2.8	0.1	0.2	0.1	1.5	1.0	**4.8**
F3P16_RS17720	0.0	0.1	7.4	0.1	0.2	0.3	1.3	1.8	**11.9**
F3P16_RS17725	0.0	0.1	2.6	0.2	0.2	0.3	0.9	1.7	**5.0**
cysK	0.7	1.2	1.6	0.9	0.9	1.1	1.1	1.3	**1.2**
F3P16_RS17735	0.0	0.0	2.1	0.0	0.0	0.0	0.3	2.7	**1.5**
rrf]	1.4	1.5	1.0	2.6	2.6	5.2	1.0	2.0	**1.9**
F3P16_RS17745	13854.5	12854.7	0.9	12098.9	10115.4	11101.1	0.8	0.9	**0.9**
F3P16_RS17750	0.5	1.0	2.1	1.0	0.8	5.2	0.8	5.3	**2.1**
F3P16_RS17755	2.0	1.5	0.8	1.7	2.4	7.6	1.4	4.4	**0.9**
F3P16_RS17760	9999.8	9999.5	1.0	9999.1	10000.8	9999.9	1.0	1.0	**1.0**
F3P16_RS17765	0.0	0.0	UC	0.0	0.0	0.0	0.0	5.1	**UC**

F3P16_RS17770	0.0	0.1	3.7	0.0	0.1	0.1	1.1	2.1	**2.9**
F3P16_RS17775	0.0	0.1	1.1	0.2	0.2	0.7	1.3	4.1	**3.3**
F3P16_RS17780	0.2	0.9	3.6	0.5	0.9	2.2	1.8	4.3	**2.1**
F3P16_RS17785	1.6	3.3	2.1	2.9	3.6	8.3	1.2	2.9	**1.8**
def]	1.1	2.1	1.9	1.7	2.0	4.7	1.2	2.7	**1.6**
F3P16_RS17795	0.1	0.1	1.2	0.1	0.1	0.3	1.9	4.4	**0.8**
dprA	0.1	0.0	0.7	0.0	0.1	0.2	2.2	5.5	**0.5**
F3P16_RS17805	0.1	0.2	1.6	0.1	0.3	0.5	2.3	4.0	**1.0**
F3P16_RS17810	0.1	0.1	1.0	0.1	0.1	0.2	0.7	2.2	**1.3**
F3P16_RS17815	0.0	0.0	1.4	0.0	0.1	0.3	1.1	5.9	**2.1**
F3P16_RS17820	0.1	0.2	2.4	0.1	0.1	0.2	1.1	1.8	**1.6**
F3P16_RS17825	0.0	0.0	0.5	0.0	0.0	0.1	0.8	1.7	**2.1**
F3P16_RS17830	0.3	0.7	2.3	0.6	0.5	1.2	0.8	2.0	**2.1**
F3P16_RS17835	0.1	2.8	20.5	2.6	3.0	2.4	1.1	0.9	**19.1**
F3P16_RS17840	6.2	6.6	1.1	6.9	9.9	27.5	1.4	4.0	**1.1**
atpD	18.4	17.9	1.0	22.7	34.9	88.4	1.5	3.9	**1.2**
atpG	24.9	24.0	1.0	27.3	51.1	108.1	1.9	4.0	**1.1**
atpA	25.6	25.6	1.0	28.5	51.3	107.2	1.8	3.8	**1.1**
F3P16_RS17860	35.9	36.8	1.0	43.5	67.2	128.9	1.5	3.0	**1.2**
F3P16_RS17865	13.7	14.2	1.0	12.1	24.4	46.7	2.0	3.8	**0.9**
atpE	5.7	7.5	1.3	7.1	15.7	25.0	2.2	3.5	**1.3**
atpB	13.3	14.0	1.1	11.7	20.6	43.6	1.8	3.7	**0.9**
F3P16_RS17880	1.5	1.9	1.3	1.7	3.0	6.1	1.7	3.5	**1.1**
F3P16_RS17885	0.3	0.7	2.0	1.1	1.5	1.4	1.3	1.3	**3.2**
F3P16_RS17890	1.0	1.0	0.9	0.9	1.2	1.4	1.3	1.5	**0.9**
znuC	0.9	1.2	1.3	0.9	1.1	1.6	1.2	1.6	**1.0**
znuB	0.9	1.2	1.4	0.9	1.1	1.9	1.2	2.1	**1.0**
F3P16_RS17905	0.1	0.2	1.4	0.1	0.2	0.2	1.7	2.5	**0.8**
F3P16_RS17910	0.7	0.9	1.3	1.1	1.1	1.5	1.0	1.3	**1.5**
F3P16_RS17915	1.0	1.6	1.7	2.4	2.7	4.6	1.1	1.9	**2.5**

argS	0.8	1.3	1.7	1.3	1.9	2.6	1.5	2.0	**1.7**
F3P16_RS17925	0.3	0.7	2.3	0.7	1.0	1.6	1.4	2.2	**2.3**
F3P16_RS17930	0.1	0.2	4.2	0.2	0.2	0.4	0.7	1.6	**4.3**
F3P16_RS17935	0.6	2.5	4.5	2.8	2.2	4.3	0.8	1.5	**5.0**
F3P16_RS17940	0.9	2.2	2.5	2.5	1.9	4.4	0.8	1.7	**2.9**
F3P16_RS17945	0.1	0.1	1.4	0.1	0.2	0.3	3.2	3.9	**1.3**
F3P16_RS17950	0.1	0.1	2.0	0.1	0.1	0.4	2.0	8.3	**0.8**
F3P16_RS17955	0.0	0.0	1.0	0.1	0.1	0.1	1.3	1.7	**2.3**
guaA	2.8	3.3	1.2	4.7	6.2	8.9	1.3	1.9	**1.7**
F3P16_RS17965	0.4	0.4	0.8	0.4	0.4	0.7	1.0	1.7	**0.9**
F3P16_RS17970	0.0	0.1	2.0	0.0	0.1	0.1	2.0	3.0	**1.1**
F3P16_RS17975	0.0	0.2	4.8	0.2	0.3	0.8	1.6	5.4	**4.2**
F3P16_RS17980	0.1	0.2	2.5	0.1	0.1	0.2	2.7	4.2	**0.6**
F3P16_RS17985	0.2	0.2	1.1	0.2	0.1	0.5	0.7	2.2	**1.3**
F3P16_RS17990	0.2	0.3	1.5	0.3	0.4	0.6	1.1	1.9	**1.5**
F3P16_RS17995	0.4	0.3	0.8	0.4	0.6	0.9	1.5	2.3	**1.1**
F3P16_RS18000	0.4	0.6	1.5	0.5	0.7	1.5	1.3	3.1	**1.2**
F3P16_RS18005	0.0	0.1	UC	0.2	0.1	0.7	0.5	4.1	**UC**
F3P16_RS18010	0.0	0.1	UC	0.2	0.1	0.7	0.5	4.1	**UC**
F3P16_RS18015	0.0	0.0	0.4	0.0	0.0	0.1	1.0	6.2	**0.6**
F3P16_RS18020	0.0	0.1	2.3	0.1	0.1	0.2	0.9	3.4	**1.8**
F3P16_RS18025	0.0	0.2	5.1	0.1	0.1	0.2	1.3	3.2	**2.0**
F3P16_RS18030	0.1	0.3	4.5	0.1	0.2	0.4	1.7	3.3	**1.7**
F3P16_RS18035	0.0	0.2	7.8	0.1	0.1	0.2	1.2	3.0	**2.8**
F3P16_RS18040	0.0	0.1	4.8	0.0	0.1	0.1	11.0	22.8	**0.2**
F3P16_RS18045	0.0	0.2	6.6	0.0	0.1	0.2	2.8	6.5	**1.3**
F3P16_RS18050	0.0	0.1	10.6	0.0	0.0	0.1	0.7	3.7	**4.3**
abaR	0.0	0.0	3.2	0.0	0.0	0.1	1.0	8.0	**1.5**
F3P16_RS18060	0.0	0.0	UC	0.0	0.0	0.0	0.0	5.7	**UC**
abaI	0.0	0.0	1.1	0.0	0.0	0.1	0.7	9.9	**1.5**

F3P16_RS18070	0.0	0.0	4.0	0.0	0.0	0.1	1.1	3.2	**2.1**
F3P16_RS18075	0.1	0.1	1.2	0.1	0.1	0.2	2.1	2.5	**0.8**
F3P16_RS18080	0.1	0.1	1.1	0.1	0.1	0.2	2.1	2.4	**0.6**
F3P16_RS18085	0.1	0.1	1.0	0.0	0.1	0.1	2.5	3.2	**0.4**
F3P16_RS18090	0.0	0.1	2.9	0.1	0.0	0.1	0.7	2.6	**1.9**
mmsB	0.1	0.1	1.5	0.1	0.1	0.1	1.1	2.1	**0.9**
F3P16_RS18100	0.0	0.1	2.5	0.1	0.1	0.1	1.0	1.0	**2.2**
F3P16_RS18105	0.0	0.1	3.8	0.1	0.0	0.1	0.5	1.5	**4.8**
F3P16_RS18110	0.6	0.9	1.6	0.9	1.2	2.5	1.3	2.8	**1.6**
F3P16_RS18115	1.4	2.1	1.5	1.6	2.1	2.0	1.4	1.3	**1.2**
F3P16_RS18120	1.7	2.8	1.6	1.7	2.4	1.7	1.4	1.0	**1.0**
alr]	2.2	3.7	1.7	1.4	1.8	2.5	1.2	1.7	**0.7**
F3P16_RS18130	1.7	3.2	1.8	1.2	1.6	3.1	1.4	2.7	**0.7**
F3P16_RS18135	0.0	0.1	2.9	0.1	0.1	0.7	0.8	5.1	**3.2**
F3P16_RS18140	0.0	0.0	6.1	0.1	0.2	0.3	2.0	3.4	**12.7**
F3P16_RS18145	0.1	0.4	4.7	0.9	1.4	1.4	1.5	1.5	**10.7**
F3P16_RS18150	0.0	0.0	0.6	0.1	0.1	0.3	1.3	2.8	**2.1**
F3P16_RS18155	0.0	0.2	6.7	0.2	0.2	0.5	0.8	2.6	**7.3**
F3P16_RS18160	0.1	0.2	2.0	0.1	0.1	0.3	0.9	3.8	**1.1**
F3P16_RS18165	0.0	0.0	5.8	0.0	0.0	0.2	0.0	5.4	**6.5**
F3P16_RS18170	0.1	0.0	0.5	0.1	0.1	0.3	1.5	4.7	**1.0**
F3P16_RS18180	0.0	0.0	UC	0.0	0.0	0.0	UC	UC	**UC**
F3P16_RS18185	0.0	0.0	19.0	0.0	0.0	0.1	0.5	4.4	**10.9**
F3P16_RS18190	0.0	0.1	5.8	0.0	0.0	0.2	0.0	4.9	**3.0**
F3P16_RS18195	0.0	0.0	UC	0.0	0.0	0.0	UC	UC	**UC**
F3P16_RS18200	0.1	0.1	1.1	0.0	0.0	0.2	0.9	5.8	**0.5**
F3P16_RS18990	0.0	0.0	UC	0.0	0.0	0.0	UC	UC	**UC**
F3P16_RS18210	0.0	0.0	UC	0.0	0.0	0.0	UC	UC	**UC**
F3P16_RS18215	0.0	0.0	UC	0.0	0.0	0.0	UC	UC	**UC**
F3P16_RS18220	0.0	0.0	UC	0.0	0.0	0.0	UC	UC	**UC**

acnD	0.7	2.0	3.1	1.7	1.4	1.6	0.8	1.0	**2.6**
prpC	0.7	2.6	3.8	2.3	1.4	2.0	0.6	0.8	**3.4**
prpB	0.5	2.1	3.8	1.8	1.0	1.8	0.6	1.0	**3.3**
F3P16_RS18240	0.0	0.2	5.3	0.1	0.1	0.4	0.6	3.4	**3.8**
F3P16_RS18245	0.0	0.0	UC	0.0	0.0	0.0	UC	UC	**UC**
F3P16_RS18250	3.6	6.9	1.9	8.2	7.5	16.4	0.9	2.0	**2.3**
dld]	0.4	1.0	2.4	1.1	1.3	0.7	1.2	0.7	**2.6**
lldD	0.6	1.3	2.2	1.5	1.7	1.2	1.1	0.8	**2.6**
lldR	0.8	2.1	2.7	2.4	2.5	1.5	1.1	0.6	**3.1**
lldP	0.3	1.1	3.2	1.2	1.1	0.8	1.0	0.7	**3.4**
F3P16_RS18275	0.3	0.9	2.8	1.1	1.1	2.7	1.0	2.4	**3.3**
galE	0.2	0.2	1.1	0.4	0.5	0.8	1.3	2.1	**2.4**
pgi]	0.7	1.3	1.8	1.3	1.7	2.0	1.3	1.5	**1.8**
F3P16_RS18290	0.1	0.2	3.1	0.2	0.2	0.2	1.1	1.2	**3.2**
galU	1.5	3.2	2.1	2.5	2.7	4.0	1.1	1.6	**1.6**
F3P16_RS18300	0.1	0.3	2.1	0.4	0.3	0.7	0.9	2.0	**2.9**
F3P16_RS18305	0.0	0.0	UC	0.0	0.0	0.0	UC	UC	**UC**
F3P16_RS18310	0.0	0.0	UC	0.0	0.0	0.0	UC	UC	**UC**
F3P16_RS18315	0.0	0.0	UC	0.0	0.0	0.0	UC	UC	**UC**
F3P16_RS18320	0.0	0.0	UC	0.0	0.0	0.0	UC	UC	**UC**
F3P16_RS18325	0.0	0.0	UC	0.0	0.0	0.0	UC	UC	**UC**
F3P16_RS18330	0.0	0.0	UC	0.0	0.0	0.0	UC	UC	**UC**
F3P16_RS18335	0.0	0.0	UC	0.0	0.0	0.0	UC	UC	**UC**
F3P16_RS18340	0.0	0.0	UC	0.0	0.0	0.0	UC	UC	**UC**
neuB	0.0	0.0	UC	0.0	0.0	0.0	UC	UC	**UC**
neuC	0.0	0.0	UC	0.0	0.0	0.0	UC	UC	**UC**
F3P16_RS18355	0.0	0.0	UC	0.0	0.0	0.0	UC	UC	**UC**
F3P16_RS18360	0.0	0.0	UC	0.0	0.0	0.0	UC	UC	**UC**
tviB	0.0	0.2	4.7	0.2	0.2	0.3	1.3	1.8	**3.9**
F3P16_RS18370	0.2	0.7	3.2	0.7	0.8	1.1	1.3	1.6	**3.0**

F3P16_RS18375	0.2	0.9	3.5	0.8	1.0	1.3	1.4	1.7	**3.0**
F3P16_RS18380	0.5	1.6	3.2	1.9	2.6	3.6	1.4	1.8	**3.9**
F3P16_RS18385	0.9	2.7	2.9	1.4	1.8	3.5	1.2	2.5	**1.6**
F3P16_RS18390	0.4	0.7	1.8	0.9	0.9	1.4	1.0	1.5	**2.4**
murJ	0.2	0.4	2.6	0.6	0.7	1.2	1.2	2.0	**3.5**
ampD	0.1	0.4	5.6	0.4	0.4	0.5	1.0	1.3	**4.5**
nadC	0.2	1.0	6.2	0.6	0.8	1.4	1.4	2.3	**3.7**
F3P16_RS18410	0.0	0.6	13.7	0.5	0.7	1.2	1.5	2.5	**11.9**
F3P16_RS18415	0.0	0.0	3.7	0.0	0.0	0.1	1.2	4.8	**1.2**
rph]	0.3	0.4	1.3	0.4	0.5	1.8	1.2	4.0	**1.4**
F3P16_RS18425	0.1	0.1	1.4	0.1	0.2	0.1	1.3	0.9	**1.8**
F3P16_RS18430	0.1	0.2	1.2	0.2	0.2	0.3	1.4	1.9	**1.3**
F3P16_RS18435	0.1	0.2	1.6	0.2	0.4	0.4	1.5	1.7	**2.0**
F3P16_RS18440	0.0	0.1	9.2	0.2	0.3	0.3	1.8	1.8	**15.4**
F3P16_RS18445	0.4	7.1	19.9	8.0	6.9	8.7	0.9	1.1	**22.2**
F3P16_RS18450	1.0	2.5	2.4	2.1	2.4	4.4	1.1	2.1	**2.1**
F3P16_RS18455	1.1	2.8	2.7	2.5	2.6	4.3	1.0	1.7	**2.4**
F3P16_RS18460	1.2	3.0	2.5	3.2	3.5	4.7	1.1	1.4	**2.8**
F3P16_RS18465	0.6	4.5	7.5	3.5	3.5	5.4	1.0	1.5	**5.8**
F3P16_RS18470	0.1	17.6	209.6	10.3	9.9	5.3	1.0	0.5	**123.3**
argA	0.2	5.3	28.5	2.3	3.2	1.7	1.4	0.7	**12.3**
F3P16_RS18480	0.0	0.0	0.7	0.0	0.0	0.1	1.7	2.7	**0.5**
F3P16_RS18485	0.1	0.1	0.9	0.1	0.1	0.1	1.4	1.6	**0.5**
ssuD	0.2	0.3	1.1	0.1	0.2	0.1	1.8	1.4	**0.4**
ssuC	0.2	0.2	1.0	0.1	0.3	0.1	2.7	1.2	**0.5**
F3P16_RS18500	0.1	0.1	0.6	0.0	0.1	0.1	2.5	1.8	**0.3**
F3P16_RS18505	0.2	0.2	0.7	0.2	0.4	0.4	1.8	2.1	**0.9**
F3P16_RS18510	0.0	0.0	2.6	0.1	0.1	0.1	1.3	1.8	**10.4**
F3P16_RS18520	0.0	0.0	0.8	0.0	0.0	0.1	1.4	2.8	**0.8**
ribF	0.4	0.5	1.4	0.6	0.8	1.2	1.4	2.2	**1.6**

ileS	2.1	3.0	1.4	3.6	4.8	5.6	1.3	1.6	**1.7**
lspA	1.9	4.4	2.3	5.6	9.1	11.6	1.6	2.1	**2.9**
F3P16_RS18540	1.3	2.5	1.9	4.2	5.8	7.5	1.4	1.8	**3.3**
F3P16_RS18545	0.0	0.2	3.8	0.2	0.2	0.4	1.1	1.8	**4.9**
F3P16_RS18550	0.0	0.1	1.1	0.1	0.1	0.1	0.7	1.4	**1.7**
rrf]	1.4	1.5	1.0	2.6	2.6	5.2	1.0	2.0	**1.9**
F3P16_RS18560	13853.1	12856.0	0.9	12100.1	10115.0	11101.4	0.8	0.9	**0.9**
F3P16_RS18565	0.5	1.0	2.1	1.0	0.8	5.2	0.8	5.3	**2.1**
F3P16_RS18570	2.0	1.5	0.7	1.8	2.4	7.6	1.3	4.3	**0.9**
F3P16_RS18575	9999.5	9999.8	1.0	10000.9	10000.9	10000.6	1.0	1.0	**1.0**
F3P16_RS18580	0.2	0.4	1.7	0.6	0.6	1.5	1.0	2.5	**2.9**
F3P16_RS18585	0.1	0.3	1.9	0.4	0.4	0.9	1.0	2.5	**2.9**
F3P16_RS18590	0.0	0.6	UC	0.7	0.5	1.2	0.7	1.8	**UC**
F3P16_RS18595	0.1	0.9	7.0	7.1	6.6	16.3	0.9	2.3	**54.3**
F3P16_RS18600	0.0	0.1	3.3	0.1	0.1	0.3	1.6	4.6	**1.8**
F3P16_RS18605	0.1	0.1	1.5	0.0	0.1	0.6	5.2	42.0	**0.2**
grpE	2.4	4.3	1.8	3.8	4.9	14.8	1.3	3.9	**1.6**
dnaK	11.8	20.1	1.7	21.3	26.0	62.9	1.2	2.9	**1.8**
F3P16_RS18620	0.0	0.0	0.0	0.0	0.0	0.0	UC	UC	**0.0**
F3P16_RS18625	0.0	0.0	1.3	0.1	0.0	0.1	0.6	0.9	**3.1**
F3P16_RS18630	0.0	0.1	4.2	0.2	0.3	0.2	1.3	0.7	**13.9**
F3P16_RS18635	1.7	15.2	9.0	12.1	11.5	8.2	0.9	0.7	**7.1**
F3P16_RS18640	0.7	0.7	1.0	1.0	1.3	1.7	1.4	1.7	**1.4**
purE	1.9	1.7	0.9	2.0	2.6	4.1	1.3	2.1	**1.1**
F3P16_RS18655	0.1	0.0	0.3	0.0	0.1	0.1	3.0	3.1	**0.2**
mpl]	1.6	4.8	3.0	6.6	6.8	11.2	1.0	1.7	**4.2**
F3P16_RS18665	0.1	0.2	1.6	0.2	0.2	0.4	0.9	1.8	**1.8**
F3P16_RS18670	0.0	0.0	UC	0.0	0.0	0.1	2.7	4.4	**UC**
F3P16_RS18675	0.0	0.1	1.5	0.0	0.1	0.3	3.3	6.1	**1.1**
F3P16_RS18680	0.2	0.1	0.8	0.2	0.1	0.2	0.7	0.9	**1.0**

F3P16_RS18685	0.2	0.2	0.8	0.1	0.1	0.2	1.6	1.9	**0.4**
F3P16_RS18690	0.0	0.0	1.1	0.0	0.0	0.0	0.7	2.3	**0.7**
guaD	0.7	0.9	1.3	0.9	1.1	2.0	1.3	2.3	**1.2**
hpt]	0.0	0.0	1.1	0.1	0.1	0.1	1.2	2.2	**1.5**
F3P16_RS18705	0.0	0.0	3.7	0.0	0.0	0.1	1.2	2.3	**3.0**
F3P16_RS18710	0.0	0.1	UC	0.1	0.1	0.2	0.8	2.1	**UC**
dmeF	0.0	0.0	3.7	0.0	0.0	0.1	3.3	10.1	**2.0**
F3P16_RS18720	0.0	0.1	UC	0.1	0.2	0.1	1.8	1.3	**UC**
mnmE	0.3	0.4	1.2	0.6	0.8	1.0	1.3	1.7	**1.7**
yidC	2.5	4.1	1.6	6.1	9.9	17.0	1.6	2.8	**2.4**
yidD	4.2	6.9	1.6	7.1	13.3	16.8	1.9	2.4	**1.7**
rnpA	3.9	6.6	1.7	7.5	13.8	16.9	1.8	2.2	**1.9**
rpmH	2.8	3.7	1.3	5.0	6.5	9.7	1.3	1.9	**1.8**
F3P16_RS18750	0.0	0.0	UC	0.0	0.0	0.0	UC	UC	**UC**
repM	0.0	0.0	UC	0.0	0.0	0.0	UC	UC	**UC**
F3P16_RS18765	0.0	0.0	UC	0.0	0.0	0.0	UC	UC	**UC**
F3P16_RS18775	0.0	0.0	UC	0.0	0.0	0.0	UC	UC	**UC**
F3P16_RS18930	0.0	0.0	UC	0.0	0.0	0.0	UC	UC	**UC**
F3P16_RS18935	0.0	0.0	UC	0.0	0.0	0.0	UC	UC	**UC**
F3P16_RS18780	0.0	0.0	UC	0.0	0.0	0.0	UC	UC	**UC**
F3P16_RS18785	0.0	0.0	UC	0.0	0.0	0.0	UC	UC	**UC**
F3P16_RS18790	0.0	0.0	UC	0.0	0.0	0.0	UC	UC	**UC**
F3P16_RS18795	0.0	0.0	UC	0.0	0.0	0.0	UC	UC	**UC**
F3P16_RS18800	0.0	0.0	UC	0.0	0.0	0.0	UC	UC	**UC**

UC, uncalculatable
